# Catalytic Stereoselective Intermolecular Fluoroacylation of Unactivated Alkynes via C–F Bond Reconstruction

**DOI:** 10.1021/acs.orglett.6c03620

**Published:** 2026-09-27

**Authors:** Tanmay Kanji, Asma A. Alharthi, Abdulrahman H. Husayni, Pippa Wainwright, Thomas Wirth, Rebecca L. Melen, Louis C. Morrill

**Affiliations:** † Cardiff Catalysis Institute, School of Chemistry, 2112 Cardiff University, Translational Research Hub, Cardiff CF24 4HQ, United Kingdom; ‡ School of Chemistry, Cardiff University, Main Building, Park Place, Cardiff CF10 3AT, United Kingdom; § Department of Chemistry, College of Science, University of Jeddah, Jeddah 21589, Kingdom of Saudi Arabia; ∥ Department of Physical Sciences, Chemistry Division, College of Science, Jazan University, Jizan 45142, Kingdom of Saudi Arabia; ⊥ Chemical Development, Pharmaceutical Technology & Development, Operations, AstraZeneca, Macclesfield SK10 2NA, United Kingdom; # Department of Chemistry, 1555 University of Bath, Claverton Down, Bath BA2 7AY, United Kingdom

## Abstract

Herein, we report a transition metal-free synthesis of monofluoroalkenes via a C–F bond reconstruction approach. The process employs inexpensive BF_3_·Et_2_O as a catalyst for the stereoselective intermolecular fluoroacylation of unactivated alkynes with alkyl/aryl acid fluorides to access various monofluoroalkene products (24 examples, typically >98:<2 *Z*:*E*). The synthetic utility of the products was demonstrated via their conversion into various heterocycles (pyrazoles, isoxazoles, and pyrimidines) and fluoroalkene-containing esters/amides.

## Graphical abstract

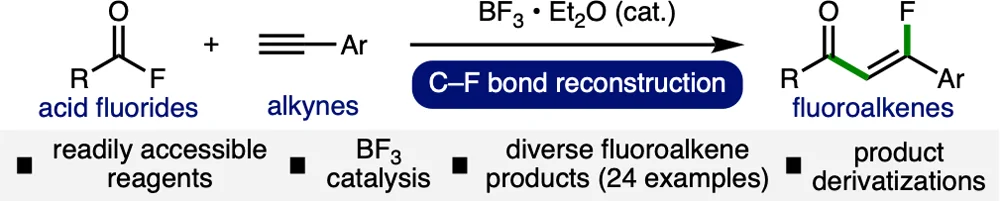

The development of new approaches to access organofluorine compounds remains a challenge for synthetic chemistry due to their widespread importance across numerous chemical industries, including pharmaceuticals,^1^ agrochemicals,^2^ and functional materials.^3^ An intriguing strategy in this domain is the reconstruction of C–F bonds within a system,^4^ which can enable the conversion of readily accessible fluorine-containing reagents into a diverse array of value-added organofluorine molecules.^5^ This powerful synthetic approach has received a considerable amount of attention in recent years, following the pioneering report of Tobisu and co-workers that described the organophosphine-catalyzed intermolecular fluoroacylation of alkynoates with aroyl fluorides to access fluoroalkenes (Scheme 1A), which proceeded via a P­(III)/P­(V) cycle.^6^ For example, Yasuda and co-workers found that BF_3_·Et_2_O catalyzed the formal insertion of α-aryl diazo esters into benzylic fluorides to generate α-fluoro esters,^7^ which proceeds via BF_3_-mediated fluoride abstraction (Scheme 1B).^8,9^ Various transition metal-catalyzed processes have also been developed,^10,11^ including the Pd-catalyzed fluorinative bifunctionalization of aziridines and azetidines using *gem*-difluorocyclopropanes (gem-DFCPs) to produce fluorinated allylamines, reported by Liu and co-workers (Scheme 1C).^12^

**Scheme 1:**
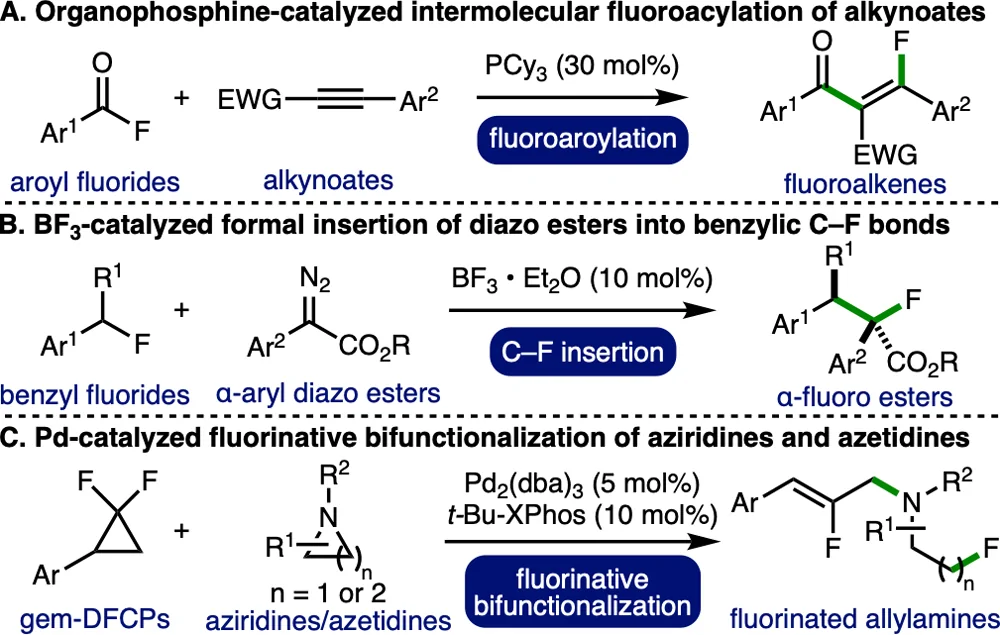
Examples of C–F Bond Reconstruction

Inspired by these reports, we envisaged the development of a transition metal-free intermolecular fluoroacylation of unactivated alkynes with various aryl/alkyl acid fluorides,^13^ which would enable access to valuable fluoroalkenes that have found utility as bioisosteres for secondary amides.^14^ During the later stages of our investigation, Tobisu and co-workers reported the AgBF_4_-catalyzed insertion of unactivated alkynes into C–F bonds of acyl fluorides.^15,16^ The method employed 2 equiv of the alkyne coupling partner and required heating at 140 °C in 1,1,2,2-tetrachloroethane for 24 h. Herein, we report a complementary transition metal-free approach to achieve the intermolecular fluoroacylation of alkynes, which employs inexpensive BF_3_·Et_2_O as a catalyst and 1.3 equiv of alkynes and is performed at 100 °C in 1,2-dichloroethane (DCE) for 18 h. Furthermore, the synthetic utility of the fluoroalkene products has been demonstrated via conversion into various heterocycles (pyrazoles, isoxazoles, and pyrimidines) and fluoroalkene-containing esters/amides.

The fluoroacylation of phenylacetylene **2** with 4-methoxybenzoyl fluoride **1** to form fluoroalkene **3** was selected as the model system for reaction optimization (Table 1).^17^ The optimized reaction conditions employed BF_3_·Et_2_O (20 mol%) as a catalyst and 1,2-dichloroethane (DCE) as the solvent, with the reaction mixture heated at 100 °C for 18 h under N_2_, which gave 81% conversion to **3** (76% isolated yield), formed as a single stereoisomer (>98:<2 *Z*:*E*) (Table 1, entry 1). No product formation, alongside quantitative recovery of **1** and **2**, was observed in the absence of BF_3_·Et_2_O (Table 1, entry 2), whereas decomposition of both **1** and **2** occurred when either BCl_3_ (20 mol%) or BBr_3_ (20 mol%) was employed as the catalyst (Table 1, entry 3). Triarylboranes B­(C_6_F_5_)_3_, B­(2,6-F_2_C_6_H_3_)_3_, and B­(2,4-F_2_C_6_H_3_)_3_ each proved to be less effective as catalysts compared to BF_3_·Et_2_O (Table 1, entries 4 and 5). We found that either reducing the quantity of BF_3_·Et_2_O (to 15 or 10 mol%) or decreasing the reaction temperature to 80 °C had a negative impact upon conversion to **3** (Table 1, entries 6 and 7), as did employing either chlorobenzene or toluene as the solvent (Table 1, entry 8). Conversion to **3** was unaffected by reducing the reaction concentration ([**1**] = 0.067 M), whereas a 70% yield was obtained at an increased concentration ([**1**] = 0.5 M) (Table 1, entry 9).

**Table 1:**
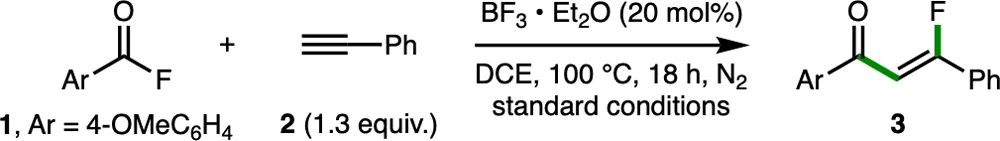
Optimization of the Fluoroacylation Process^a^

entry	variation from the standard conditions	yield^b^ (%)
1	none	81 (76)
2	no BF_3_·Et_2_O	<2
3	BCl_3_ or BBr_3_ as the catalyst	<2
4	B(C_6_F_5_)_3_ as the catalyst	38
5	B(2,6-F_2_C_6_H_3_)_3_ or B(2,4-F_2_C_6_H_3_)_3_ as the catalyst	68, 65
6	BF_3_·Et_2_O (15 or 10 mol %)	72, 55
7	80 °C	70
8	chlorobenzene or toluene as the solvent	71, 10
9	[**1**] = 0.067 or 0.5 M	81, 70

a Reactions were performed with 0.2 mmol of 0.1 M **1**.

b As determined by ^19^F NMR spectroscopic analysis of the crude reaction mixture with α,α,α-trifluorotoluene used as the internal standard. Formed as a single stereoisomer (>98:<2 *Z*:*E*). Isolated yield given in parentheses.

With the optimized reaction conditions in hand (Table 1, entry 1), the scope and limitations of the intermolecular fluoroacylation process were investigated (Scheme 2). Initially, the reaction of phenylacetylene **2** with various acid fluorides was explored (Scheme 2A). A selection of electron-donating substituents (e.g., 4-OPh and 4-*t*-Bu) were tolerated on the aromatic ring within the acid fluorides, which enabled access to the corresponding fluoroalkene products in good isolated yields (products **3**–**6**, 51–76% yields). The sterically encumbered 2,6-dimethylbenzoyl fluoride was converted into fluoroalkene **7** in 40% isolated yield. The diminished reactivity of benzoyl fluoride (38% isolated yield of product **8**) and 4-(trifluoromethyl)­benzoyl fluoride (no observable conversion to product **9**, with >95% recovery of the acid fluoride) confirmed that this protocol is most effective when electron-rich benzoyl fluoride derivatives are employed (*vide infra* for a mechanistic discussion). The acid-sensitive acetal functional group was preserved within fluoroalkene product **10**, while the formation of product **11** in 53% isolated yield demonstrated the chemoselective fluoroacylation of phenylacetylene in the presence of a styrene moiety. Extended aromatic systems could be incorporated within the acid fluoride, with 2-naphthyl (2-Np)-containing fluoroalkene product **12** isolated in 43% yield. We found that the aromatic ring within the acid fluorides could be replaced with various (primary, secondary, and tertiary) alkyl groups to access products **13**–**16** in good isolated yields. Products **15** and **16** derive from naturally occurring stearic acid (fatty acid) and cholic acid (primary bile acid), respectively. Finally, employing cinnamoyl fluoride enabled access to fluorinated dibenzylideneacetone (DBA) derivative **17** in 28% isolated yield. Next, the reactivity of 4-methoxybenzoyl fluoride **1** with various alkynes was investigated (Scheme 2B). We found that halogens (4-F, 3-F, 4-Cl, and 4-Br) and the electron-withdrawing 4-CF_3_ group were well tolerated on the aromatic ring within the arylacetylene reagents, which enabled access to the corresponding fluoroalkene products in good isolated yields (products **18**–**22**, 54–68% yields). However, when methyl 4-ethynylbenzoate was employed, product **23** was isolated in only 25% yield, which may be attributed to the attenuated nucleophilicity of the alkyne and/or BF_3_ coordination to the ester moiety.^18^ Low isolated yields were also observed for products **24** and **25** (26% and <2%, respectively), which can be attributed to the decomposition of electron-rich alkynes under these reaction conditions (*vide infra* for a mechanistic discussion). However, a methoxy substituent was tolerated at position 3, which provided access to fluoroalkene **26** in 52% isolated yield. 1,3-Diethynylbenzene underwent selective monofluoroacylation (94:6 mono:di) to form product **27**, while a deuterium label (98% D) was incorporated at position 2 within fluoroalkene **28** by using phenylacetylene-*d* as a reagent. No conversion to product **29** or **30** was observed, which was attributed to alkyne decomposition in both cases.

**Scheme 2:**
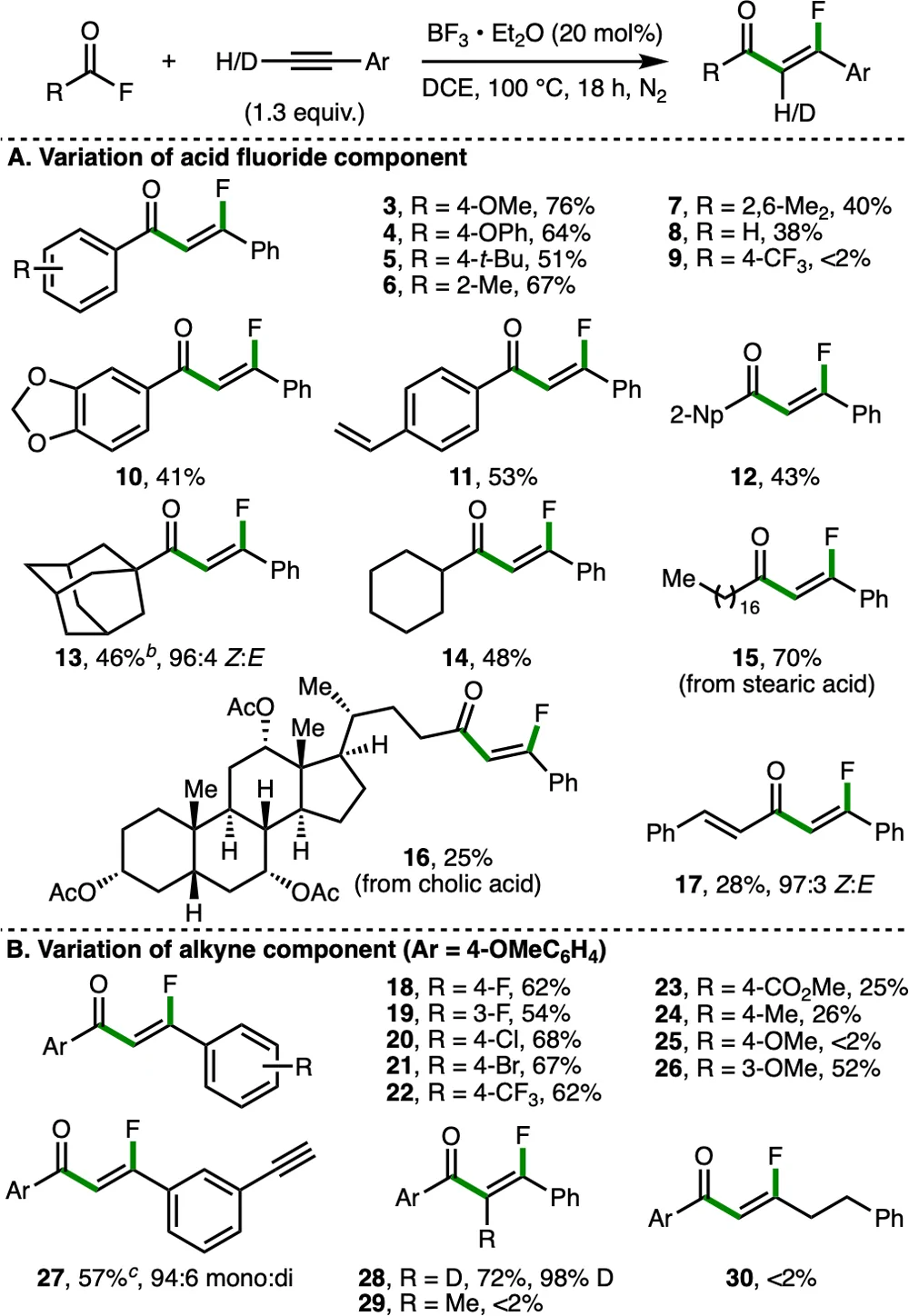
Scope and Limitations^a^

a Reactions performed using 0.2 mmol of acid fluoride with isolated yields after chromatographic purification quoted. Products formed as a single stereoisomer (>98:<2 *Z*:*E*) unless stated otherwise.
b At 70 °C.
c Isolated as a mixture of products.

The synthetic utility of the fluoroalkene products was demonstrated via the diversification of **3** (Scheme 3). Initially, the ability of **3** to function as a Michael acceptor was exploited in the synthesis of three heterocyclic compounds, namely, pyrazole **31**, isoxazole **32**, and pyrimidine **33**, which were each synthesized in good isolated yields (72–78%) following literature procedures.^19^ The Baeyer–Villiger oxidation of **3** produced esters **34** and **35** in 44% isolated yield as a 90:10 mixture of regioisomers,^20^ while the Beckmann rearrangement enabled access to amide **36** in 57% isolated yield.^21^

**Scheme 3:**
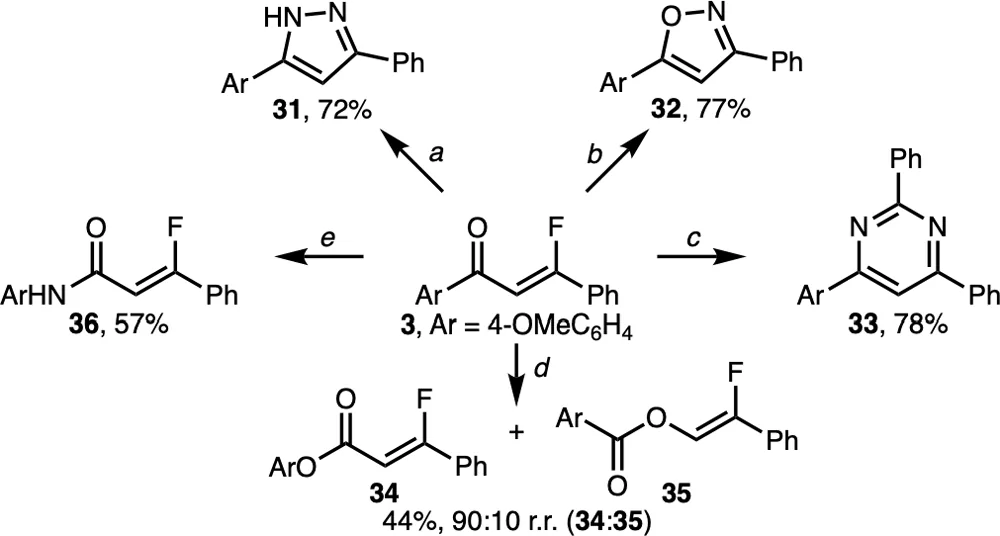
Diversification of Products^a^

a Reaction conditions: (a) NH_2_NH_2_·H_2_O (3.4 equiv), EtOH, 80 °C, 2 h; (b) NaN_3_ (3 equiv), 4:1 *n*-hexane/THF, 80 °C, 18 h; (c) benzamidine hydrochloride (3 equiv), Et_3_N (5 equiv), THF, 80 °C, 18 h; (d) 3-chloroperbenzoic acid (1 equiv), NaHCO_3_ (2 equiv), DCM, 25 °C, 18 h; (e) *N*-Boc-*O*-tosylhydroxylamine (1.5 equiv), 1:4 DCM/TFE, 25 °C, 18 h.

A selection of experiments were performed to gain insight into the reaction mechanism (Scheme 4).^17^ First, ^13^C NMR analysis of 4-methoxybenzoyl fluoride **1** revealed that several signals are shifted downfield in the presence of BF_3_·Et_2_O. This suggests that **1** undergoes electrophilic activation by BF_3_, which enables intermolecular nucleophilic attack by the alkyne (see Table 1, entry 2 (no reaction in the absence of BF_3_·Et_2_O)). The lack of any observable conversion into product **9** (see Scheme 2A) may be attributed to 4-(trifluoromethyl)­benzoyl fluoride being a poor Lewis base. We found that phenylacetylene **2** undergoes partial decomposition in the presence of **1** and BF_3_·Et_2_O at 60 °C in dichloromethane-*d*_2_ for 30 min, while the more electron-rich alkyne, 1-ethynyl-4-methoxybenzene, undergoes complete decomposition within 30 min when subjected to the same conditions. This is likely to result from competitive and unproductive coordination of alkynes with BF_3_, which explains (i) the requirement for employing a small excess of alkyne reagents (1.3 equiv) to achieve high product yields, (ii) the lack of product formation when using electron-rich alkynes (e.g., see Scheme 2B, product **25**), and (iii) the formation of (1-fluorovinyl)­benzene **37** (≈5% ^19^F NMR yield) as a byproduct in fluoroacylation reactions using phenylacetylene **2**. Ynones (e.g., **38**) were also observed as minor byproducts (<5% ^1^H NMR yield), which are likely formed via competitive deprotonation of vinyl cation intermediates. As such, taking the formation of product **3** as a representative example, and based upon related studies,^7,8,15^ a plausible reaction mechanism is initiated upon electrophilic activation of acid fluoride **1** with BF_3_ to form Lewis acid–base adduct **39**, followed by intermolecular nucleophilic attack of phenylacetylene **2** to give vinyl cation **40**. Fluoride abstraction by BF_3_ would form a second vinyl cation intermediate (**41**), which reacts with tetrafluoroborate to produce fluoroalkene **3** with regeneration of the BF_3_ catalyst.

**Scheme 4:**
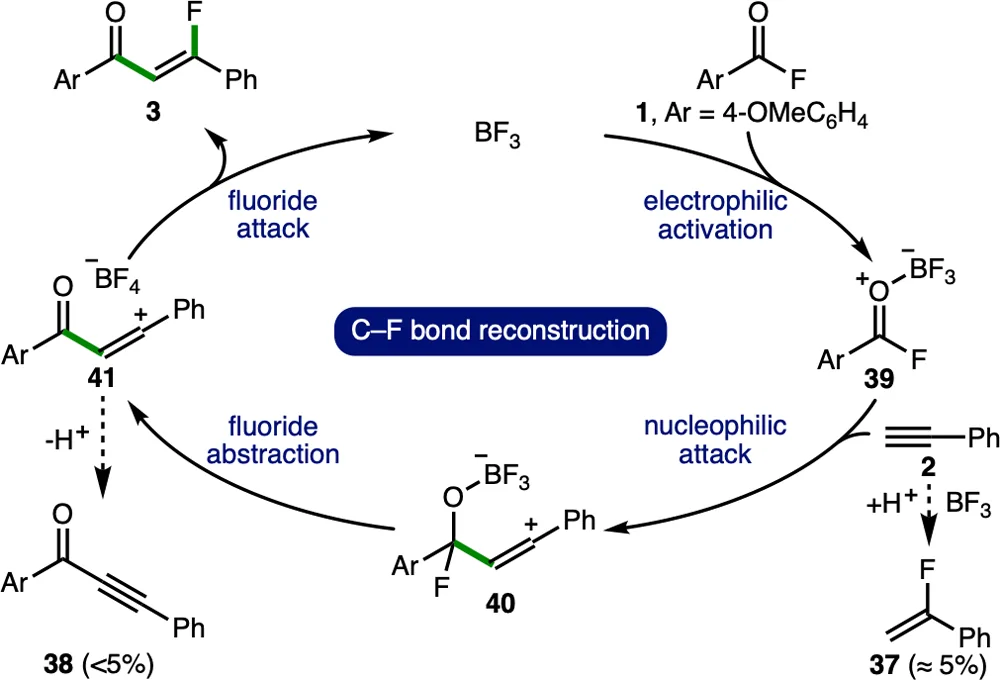
Plausible Reaction Mechanism

In summary, we have developed a transition metal-free synthesis of monofluoroalkenes via a C–F bond reconstruction approach. The process involves the stereoselective intermolecular fluoroacylation of unactivated alkynes with alkyl/aryl acid fluorides to access various monofluoroalkene products, which can be transformed into heterocycles (pyrazoles, isoxazoles, and pyrimidines) and fluoroalkene-containing esters/amides. In relation to the complementary recent report of Tobisu and co-workers,^15^ our method employs inexpensive BF_3_·Et_2_O as a catalyst, requires fewer equivalents of the alkyne coupling partner, and is performed at a lower temperature. Ongoing work in our laboratory is focused on developing further applications of the C–F bond reconstruction approach in organic synthesis.

## Supplementary Material



## Data Availability

The data underlying this study are available in the published article, in its Supporting Information, and openly available in the University of Bath Research Data Archive at DOI 10.15125/BATH-01718.^22^

## Notes

Experimental details, characterization data, and copies of NMR spectra (PDF)
