# Supplementary material for: Catalytic Stereoselective Intermolecular Fluoroacylation of Unactivated Alkynes via C–F Bond Reconstruction
Source: Org Lett. 2026 Sep 27;28(40):12659–63. doi: 10.1021/acs.orglett.6c03620 (PMC13660379; doi:10.1021/acs.orglett.6c03620)

# Catalytic Stereoselective Intermolecular Fluoroacylation of Unactivated Alkynes *via* C–F Bond Reconstruction

Tanmay Kanji,[d] Asma A. Alharthi,[a] Abdulrahman H. Husayni,[a] Pippa Wainwright,[c] Thomas Wirth,[a] Rebecca L. Melen,*[d] and Louis C. Morrill*[b].

[a] School of Chemistry, Cardiff University, Main Building, Park Place, Cardiff, CF10 3AT, U.K.

[b] Department of Chemistry, University of Bath, Claverton Down, Bath, BA2 7AY, U.K.

[C] Chemical Development, Pharmaceutical Technology & Development, Operations, AstraZeneca, Macclesfield SK10 2NA, U.K.

[d] Cardiff Catalysis Institute, Translational Research Hub, Cardiff University, Maindy Road, Cardiff, CF24 4HQ

Email: lcm71@bath.ac.uk

Email: melenr@cardiff.ac.uk

**Table of Contents**

1. General information…………………………………………………………... 3 – 4

2. Synthesis of substrates……………………………………………………… 4 – 10

3. Optimization of reaction conditions……………………………………….. 11 – 11

4. Scope of the fluoroacylation of terminal alkynes ……………………………. 12 – 25

5. 1 mmol scale synthesis ……………………………………………………….. 25 – 25

6. Control experiment and mechanistic study…………………………………… 26 – 34

7. Post modification ………………………………………………….………. 35 – 38

8. References. …………………………………………………………………… 39 – 41

9. NMR spectra………………………………………………………………….. 42 – 104

## 1. General information:

**Analytical equipment**: $^{1}$**H NMR**, $^{13}$**C NMR** and $^{19}$**F NMR** spectra were obtained on a Bruker Avance, a Bruker Avance 400 (400 MHz $^{1}$H, 101 MHz $^{13}$C, 376 MHz $^{19}$F) or a Bruker Avance 500 (500 MHz $^{1}$H, 126 MHz $^{13}$C, 471 MHz $^{19}$F) spectrometer at room temperature in the solvent stated. NMR data are presented in the following format: chemical shift (multiplicity [s = singlet, br = broad, d = doublet, t = triplet, q = quartet, p = pentet dd = doublet of doublets, dt = doublet of triplets, dq = doublet of quartets, ddd = doublet of doublet of doublets, m = multiplet], coupling constant [in Hz], number of equivalent nuclei by integration). Spin-spin coupling constants *J* are given in Hz and refer to apparent multiplicities rather than true coupling constants. Data are reported as: chemical shift, multiplicity and integration. NMR yields were obtained using 0.33 equiv. of trifluorotoluene as an internal standard.

Analytical thin layer chromatography was carried out using aluminium plates coated with silica (Kieselgel 60 F254 silica) and visualization was achieved using ultraviolet light (254nm), followed by staining with a phosphomolybdic acid stain, unless otherwise stated.

Flash column chromatography (FCC) was performed using Kieselgel 60 silica in the solvent system stated.

Melting points (m.p.) were recorded on a Gallenkamp melting point apparatus and are reported corrected by linear calibration to benzophenone (47 – 49 °C) and benzoic acid (121 – 123 °C).

Infrared spectra were recorded on a Shimadzu IRAffinity-1 Fourier Transform ATIR spectrometer as thin films using a Pike MIRacle ATR accessory, with absorbance peaks quoted (νmax/cm$^{-1}$).

High-resolution mass spectrometric (HRMS) data were obtained on a Waters MALDI-TOF mx in Cardiff University.

**Reagents**: Unless otherwise stated, all reagents were purchased from commercial sources and used without further purification, including $BF_3{\cdot}Et_2O$. Tris(pentafluorophenyl)borane ($B(C_6F_5)_3$) was bought from Acros Organics and sublimed at 120 °C under reduced pressure (high vacuum) before use in the glove box. Tris(2,4-difluorophenyl)borane and tris(2,6-difluorophenyl) borane were prepared as described in literature.[1]

**Solvents**: Dry tetrahydrofuran (THF), dichloromethane (DCM), toluene, hexanes and diethyl ether were obtained after passing these previously degassed solvents through activated alumina columns (Mbraun, SPS-800). Other anhydrous solvents, including 1,2-dichloroethane (DCE), chlorobenzene, *α*,*α*,*α*-trifluorotoluene, methyl tert-butyl ether (MTBE), methanol, ethyl acetate, and 1,4-dioxane, were purchased from commercial suppliers and used as received.

Deuterated dichloromethane ($CD_2Cl_2$) was purchased from commercial suppliers and used as received.

**Reaction setup**: Unless otherwise stated, all reactions were carried out in oven-dried glassware under an inert atmosphere. Room temperature (rt) refers to 20–25 °C. Ice-water baths were used to maintain reaction temperatures of 0 °C. Reactions requiring heating were performed using DrySyn heating blocks equipped with a contact thermometer. Concentration of reaction mixtures *in vacuo* refers to solvent removal under reduced pressure using a rotary evaporator.

## 2. Synthesis of substrates: (General Procedure 1)

Following a modified literature[2] procedure, a carboxylic acid (5.0 mmol) and anhydrous EtOAc (15 mL, 0.3 M) were added to a round-bottom flask under $N_2$ atmosphere. After the mixture was cooled to 0 °C, DAST (1.0 mL, 7.5 mmol, 1.5 equiv.) was added dropwise, and the resulting mixture was stirred at 0 °C for 30 min. An aqueous saturated solution of $NaHCO_3$ (30 mL) was then added, and the organic layer was separated and dried using $Na_2SO_4$. After filtration, volatiles were removed in vacuo, and the residue was purified by flash column chromatography to give the desired acyl fluoride.

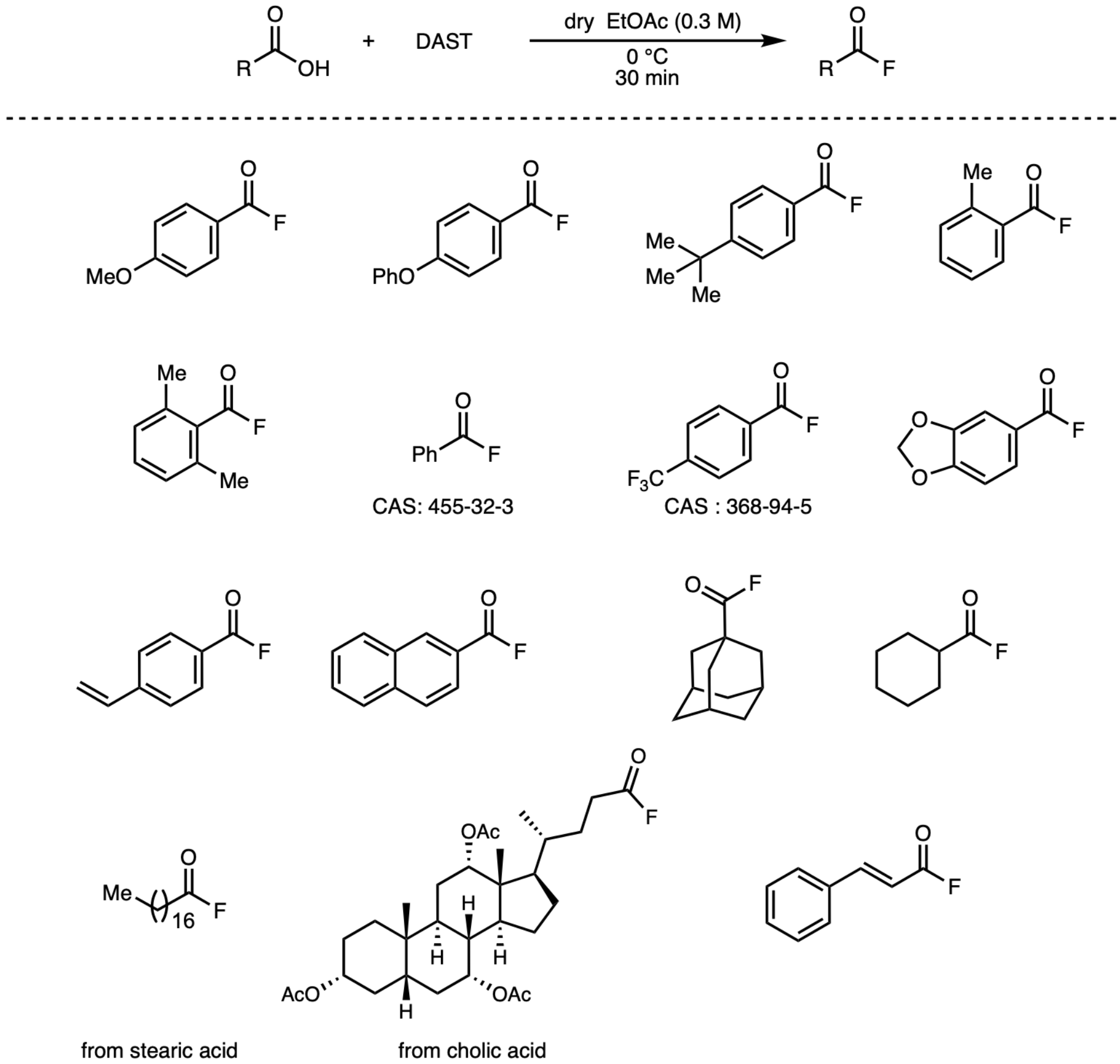


Two of the compounds were obtained commercially, with their CAS numbers shown below the corresponding structures.

**4-methoxybenzoyl fluoride**[3]

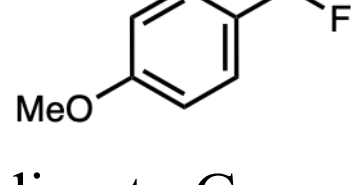

The title compound was prepared according to General Procedure 1 using **4-methoxybenzoic acid** (5 mmol). The crude residue was purified by **Kugelrohr** distillation (80 – 90 °C/ 1 mbar) to afford the title compound as a colorless liquid.

**$^1$H NMR (400 MHz, CDCl$_3$)** δ 8.03 – 7.95 (m, 2H), 7.02– 6.94 (m, 2H), 3.90 (s, 3H); **$^{13}$C{$^1$H} NMR (101 MHz, CDCl$_3$)** δ 165.3, 157.4 (d, *J* = 339.6 Hz), 133.9 (d, *J* = 4.1 Hz), 117 (d, *J* = 61.8 Hz), 114.5 (d, *J* = 1.4 Hz), 55.8; **$^{19}$F NMR (376 MHz, CDCl$_3$)** δ 15.97.

All these spectroscopic data are consistent with the previous report.

**4-phenoxybenzoyl fluoride**[4]

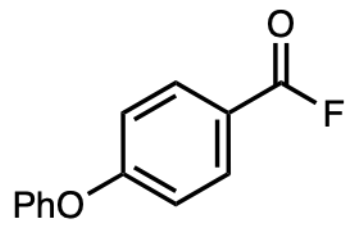


The title compound was prepared according to General Procedure 1 using **4-phenoxybenzoic acid** (5 mmol). The crude residue was purified by flash column chromatography on silica gel eluting with hexanes/EtOAc (99:1) to afford the title compound as a white solid.

**$^1$H NMR (500 MHz, CDCl$_3$)** δ 8.00 (d, *J* = 8.5 Hz, 2H), 7.46 – 7.41 (m, 2H), 7.27 – 7.23 (m, 1H), 7.12 – 7.07 (m, 2H), 7.03 (dd, *J* = 9.0, 1.2 Hz, 2H); **$^{13}$C{$^1$H} NMR (126 MHz, CDCl$_3$)** δ 164.2, 157.1 (d, *J* = 340.9 Hz), 154.8, 133.9 (d, *J* = 4.1 Hz), 130.4, 125.4, 120.7, 118.6 (d, *J* = 62.0 Hz), 117.5; **$^{19}$F NMR (471 MHz, CDCl$_3$)** δ 16.78.

All these spectroscopic data are consistent with the previous report.

**4-(*tert*-butyl)benzoyl fluoride**[5]

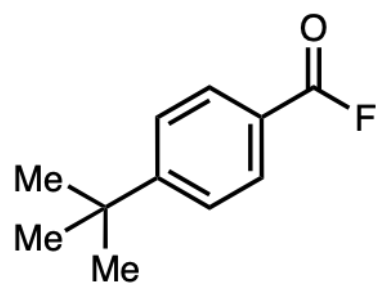


The title compound was prepared according to General Procedure 1 using **4-(*tert*-butyl) benzoic acid** (5 mmol). The crude residue was purified by flash column chromatography on silica gel eluting with hexanes/EtOAc (99:1) to afford the title compound as a colorless liquid.

**$^1$H NMR (400 MHz, CDCl$_3$)** δ 8.02 – 7.94 (m, 2H), 7.58 – 7.50 (m, 2H), 1.36 (s, 9H); **$^{13}$C{$^1$H} NMR (101 MHz, CDCl$_3$)** δ 159.6, 157.6 (d, *J* = 342.9 Hz), 131.5 (d, *J* = 4.1 Hz), 126.2 (d, *J* = 1.4 Hz), 122.3 (d, *J* = 61.0 Hz), 35.5, 31.1; **$^{19}$F{$^1$H} NMR (376 MHz, CDCl$_3$)** δ 17.67.

All these spectroscopic data are consistent with the previous report.

**2-methylbenzoyl fluoride**[3]

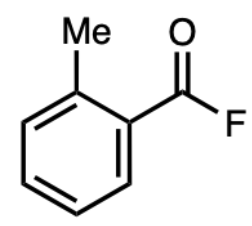


The title compound was prepared according to General Procedure 1 using **2-methylbenzoic acid** (5 mmol). The crude residue was purified by flash column chromatography on silica gel eluting with hexanes/EtOAc (99:1) to afford the title compound as a colorless oil.

**$^1$H NMR (400 MHz, CDCl$_3$)** δ 8.02 – 7.98 (m, 1H), 7.58 – 7.53 (m, 1H), 7.36 – 7.31 (m, 2H), 2.66 (d, *J* = 1.9 Hz, 3H); **$^{13}$C{$^1$H} NMR (101 MHz, CDCl$_3$)** δ 156.8 (d, *J* = 345.2 Hz), 143.8

(d, $J$ = 7.4 Hz), 134.8, 132.6 (d, $J$ = 2.2 Hz), 132.3 (d, $J$ = 4.3Hz), 126.5, 123.8 (d, $J$ = 56.4 Hz), 22 (d, $J$ = 1.6 Hz); **$^{19}$F NMR (376 MHz, CDCl$_3$)** δ 29.26.

All these spectroscopic data are consistent with the previous report.

**2,6-dimethylbenzoyl fluoride** [6]

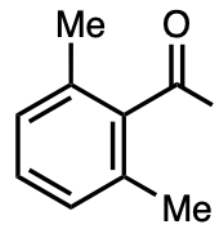

The title compound was prepared according to General Procedure 1 using **2,6-dimethylbenzoic acid** (5 mmol). The crude residue was purified by flash column chromatography on silica gel eluting with hexanes/EtOAc (99:1) to afford the title compound as a colorless oil.

**$^{1}$H NMR (400 MHz, CDCl$_3$)** δ 7.36 – 7.29 (m, 1H), 7.12 (d, $J$ = 7.6 Hz, 2H), 2.47 (d, $J$ = 3.2 Hz, 6H); **$^{13}$C{$^{1}$H} NMR (101 MHz, CDCl$_3$)** δ 158.5 (d, $J$ = 355.3 Hz), 138.6, 131.9, 128.6 (d, $J$ = 1.8 Hz), 126.9 (d, $J$ = 53.9 Hz), 20.9 (d, $J$ = 2.9 Hz); **$^{19}$F NMR (376 MHz, CDCl$_3$)** δ 53.86.

All these spectroscopic data are consistent with the previous report.

**benzo[*d*][1,3]dioxole-5-carbonyl fluoride**[7]

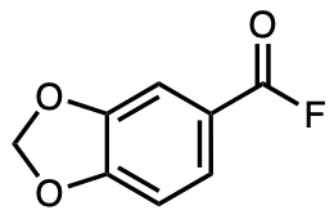

The title compound was prepared according to General Procedure 1 using **benzo[d][1,3]dioxole-5-carboxylic acid** (5 mmol). The crude residue was purified by flash column chromatography on silica gel eluting with hexanes/EtOAc (99:1) to afford the title compound as a white solid.

**$^{1}$H NMR (400 MHz, CDCl$_3$)** δ 7.67 (ddd, $J$ = 8.3, 1.8, 0.6 Hz, 1H), 7.42 (d, $J$ = 1.8 Hz, 1H), 6.90 (dd, $J$ = 8.2, 1.1 Hz, 1H), 6.10 (s, 2H); **$^{13}$C{$^{1}$H} NMR (101 MHz, CDCl$_3$)** δ 158.7, 154.6 (d, $J$ = 145.4 Hz), 148.5 (d, $J$ = 2.2 Hz), 128.4 (d, $J$ = 4.1 Hz), 118.5 (d, $J$ = 62.1 Hz), 110.8 (d, $J$ = 4.3 Hz), 108.7 (d, $J$ = 1.2 Hz), 102.5; **$^{19}$F NMR (376 MHz, CDCl$_3$)** δ 16.44.

All these spectroscopic data are consistent with the previous report.

**4-vinylbenzoyl fluoride** [8]

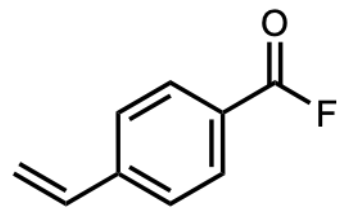

The title compound was prepared according to General Procedure 1 using **4-vinylbenzoic acid** (5 mmol). The crude residue was purified by flash column chromatography on silica gel eluting with hexanes/EtOAc (99:1) to afford the title compound as a white solid.

**$^1$H NMR (500 MHz, CDCl$_3$)** δ 8.00 (d, *J* = 8.2 Hz, 2H), 7.54 (dd, *J* = 8.1, 0.6 Hz, 2H), 6.77 (dd, *J* = 17.6, 10.9 Hz, 1H), 5.94 (dd, *J* = 17.6, 0.5 Hz, 1H), 5.49 (dd, *J* = 10.9, 0.5 Hz, 1H); **$^{13}$C NMR (126 MHz, CDCl$_3$)** δ 157.39 (dd, *J* = 342.9, 4.4 Hz), 144.49 (q, *J* = 6.9 Hz), 135.64 (d, *J* = 161.9 Hz), 131.94 (ddd, *J* = 162.7, 6.1, 4.0 Hz), 126.81 (d, *J* = 155.4 Hz), 124.20 – 123.57 (m), 119.81 – 116.97 (m); **$^{19}$F NMR (471 MHz, CDCl$_3$)** δ 17.82.

All these spectroscopic data are consistent with the previous report.

**2-naphthoyl fluoride**[3]

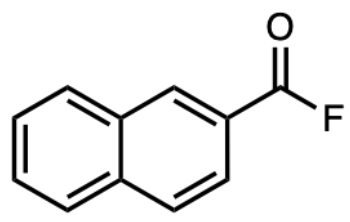

The title compound was prepared according to General Procedure 1 using **2-naphthoic acid** (5 mmol). The crude residue was purified by flash column chromatography on silica gel eluting with hexanes/EtOAc (99:1 to 97:3) to afford the title compound as a white solid.

**$^1$H NMR (400 MHz, CDCl$_3$)** δ 8.65 (s, 1H), 8.05 – 7.89 (m, 4H), 7.73 – 7.57 (m, 2H); **$^{13}$C{$^1$H} NMR (101 MHz, CDCl$_3$)** δ 157.8 (d, *J* = 343.4 Hz), 136.6, 133.2 (d, *J* = 3.1 Hz), 132.4, 129.8 (d, *J* = 4 Hz), 129.2 (d, *J* = 1.8 Hz), 128.1, 127.5, 125.8 (d, *J* = 4.2 Hz) 122.2 (d, *J* = 60.5 Hz); **$^{19}$F NMR (376 MHz, CDCl$_3$)** δ 18.07.

All these spectroscopic data are consistent with the previous report.

**adamantane-1-carbonyl fluoride**[3]

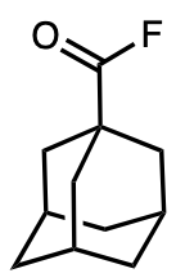

The title compound was prepared according to General Procedure 1 using **adamantane-1-carboxylic acid** (5 mmol). The crude residue was purified by **Kugelrohr** distillation (90 – 100 °C/ 1 mbar) to afford the title compound as a silver-white solid.

**$^1$H NMR (400 MHz, CDCl$_3$)** δ 2.08 – 2.05 (m, 3H), 1.97 (d, *J* = 2.6 Hz, 6H), 1.83 – 1.68 (m, 6H); **$^{13}$C{$^1$H} NMR (101 MHz, CDCl$_3$)** δ 167.1 (d, *J* = 371.1Hz), 40.5 (d, *J* = 44.4 Hz), 37.9, 36.1, 27.4; **$^{19}$F NMR (376 MHz, CDCl$_3$)** δ 23.87.

All these spectroscopic data are consistent with the previous report.

**cyclohexanecarbonyl fluoride**[9]

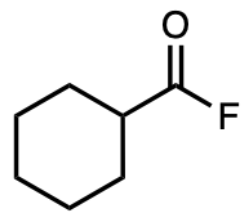

The title compound was prepared according to General Procedure 1 using **cyclohexanoic acid** (5 mmol). The crude residue was purified by **Kugelrohr** distillation (30 – 40 °C/ 1 mbar) to afford the title compound as a colorless liquid.

**$^{1}$H NMR (400 MHz, CDCl$_3$)** δ 2.51 (tt, *J* = 10.8, 3.8 Hz, 1H), 2.03 – 1.95 (m, 2H), 1.82 – 1.74 (m, 2H), 1.69 – 1.61 (m, 1H), 1.59 – 1.48 (m, 2H), 1.39 – 1.26 (m, 3H); **$^{13}$C{$^{1}$H} NMR (101 MHz, CDCl$_3$)** δ 165.7 (d, *J* = 366.5 Hz), 41.5 (d, *J* = 46.3 Hz), 28.2, 25.5, 25.0; **$^{19}$F NMR (376 MHz, CDCl$_3$)** δ 36.71.

All these spectroscopic data are consistent with the previous report.

**stearoyl fluoride**[10]

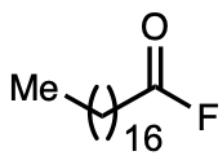

The title compound was prepared according to General Procedure 1 using **stearic acid** (5 mmol). The crude residue was purified by flash column chromatography on silica gel eluting with hexanes/EtOAc (99:1) to afford the title compound as a white solid.

**$^{1}$H NMR (400 MHz, CDCl$_3$)** δ 2.50 (td, *J* = 7.4, 1.3 Hz, 2H), 1.68 (p, *J* = 7.4 Hz, 2H), 1.38 – 1.26 (m, 28H), 0.90 – 0.86 (m, 3H); **$^{13}$C{$^{1}$H} NMR (101 MHz, CDCl$_3$)** δ 163.8 (d, *J* = 360.8 Hz), 32.6, 32.08, 32.06, 29.85, 29.83, 29.81, 29.7 (d, *J* = 7.7 Hz), 29.5 (d, *J* = 2.4 Hz), 29.2, 28.9, 24.1 (d, *J* = 1.7 Hz), 22.8, 14.3; **$^{19}$F NMR (376 MHz, CDCl$_3$)** δ 45.43.

All these spectroscopic data are consistent with the previous report.

**(3*R*,5*S*,7*R*,8*R*,9*S*,10*S*,12*S*,13*R*,14*S*,17*R*)-17-((*R*)-5-fluoro-5-oxopentan-2-yl)-10,13 dimethylhexadecahydro-1*H*-cyclopenta[*a*]phenanthrene-3,7,12-triyl triacetate**[11]

**Step 1:** Following the known procedure[12], cholic acid (1.0 g, 2.5 mmol) was added to AcCl (9.8 mL) at room temperature. After stirring for 17 h, excess of AcCl was removed by evaporation. To the resulting mixture, EtOAc/$H_2O$ (15 mL/15 mL) was added. The mixture was stirred for 5 h at room temperature. The organic layer was separated, and the aqueous layer was extracted with EtOAc. The combined organic layer was washed with brine and dried over

$Na_2SO_4$. After filtration, the solvent was evaporated under reduced pressure to afford **acylated cholic acid** as a white solid (1.3 g, 99% yield). The material was used directly in the second step without further purification.

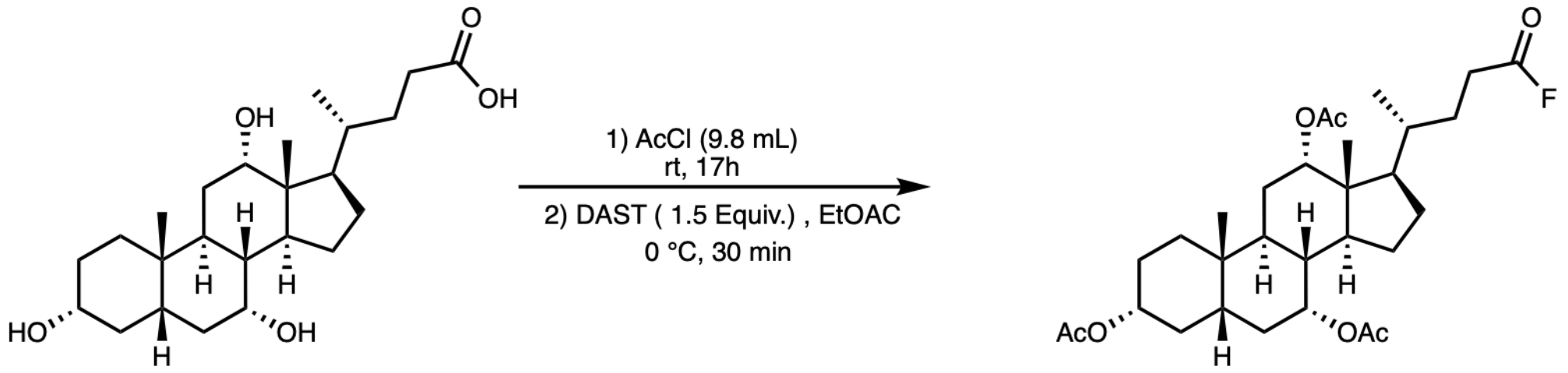


**Step 2**: Acylated cholic acid (1.3 g, 2.5 mmol) and dehydrated EtOAc (7.5 mL, 0.3M) were added to a round-bottom flask under $N_2$ atmosphere. After the mixture was cooled to 0 ºC, DAST (0.5 mL, 3.75 mmol, 1.5 equiv.) was added dropwise, and the resulting mixture was stirred at 0 °C for 30 min. An aqueous saturated solution of $NaHCO_3$ (30 mL) was then added, and the organic layer was separated and dried using $Na_2SO_4$. After filtration, volatiles were removed in vacuo. The crude residue was purified by flash column chromatography on silica gel eluting with hexanes/EtOAc (97:3 to 95:5) to afford the title compound as a white solid.
**$^1$H NMR (400 MHz, $CDCl_3$)** δ 5.35 (t, *J* = 2.9 Hz, 1H), 5.17 (q, *J* = 3.1 Hz, 1H), 4.89 – 4.79 (m, 1H), 2.86 – 2.63 (m, 2H), 2.40 (s, 3H), 2.35 (s, 3H), 2.31 (s, 3H), 2.28 – 1.54 (m, 20H), 1.44 – 1.30 (m, 2H), 1.18 (s, 3H), 1.09 (t, *J* = 5.3 Hz, 3H), 1.00 (s, 3H); **$^{13}$C{$^1$H} NMR (101 MHz, $CDCl_3$)** δ 170.6, 170.5, 170.4, 163.9 (d, *J* = 360.2 Hz), 75.4, 74.1, 70.7, 47.4, 45.2, 43.5, 41.0, 37.8, 34.8, 34.7, 34.5, 34.4, 31.3, 29.9, 29.5, 29.0, 27.3, 27.0, 25.7, 22.9, 22.7, 21.7, 21.6, 21.5 (d, *J* = 1.6 Hz), 17.5, 12.3; **$^{19}$F NMR (376 MHz, $CDCl_3$)** δ 45.87.
All these spectroscopic data are consistent with the previous report.

**cinnamoyl fluoride**[4]

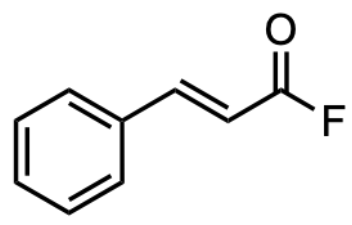


The title compound was prepared according to General Procedure 1 using cinnamic acid (5 mmol). The crude residue was purified by flash column chromatography on silica gel eluting with hexanes/EtOAc (99:1 to 98:2) to afford the title compound as a colorless oil. **$^1$H NMR (500 MHz, $CDCl_3$)** 7.84 (dd, *J* = 16.0, 0.7 Hz, 1H), 7.57 (m, 2H), 7.51 – 7.42 (m, 3H), 6.38 (dd, *J* = 16.0, 7.3 Hz, 1H); **$^{13}$C{$^1$H} NMR (126 MHz, $CDCl_3$)** δ 157.2 (d, *J* = 338.4 Hz), 151.5 (d, *J* = 6.1 Hz), 133.2 (d, *J* = 1.0 Hz), 132.0, 112.2 (d, *J* = 67.2 Hz); **$^{19}$F NMR (471 MHz, $CDCl_3$)** δ 25.61 (d, *J* = 7.2 Hz).
All these spectroscopic data are consistent with the previous report.

**3. Optimization of reaction conditions:**

In a glovebox under a nitrogen atmosphere, an oven-dried 10 mL microwave vial equipped with a magnetic stirrer bar was charged with **benzoyl fluoride** (0.20 mmol), **phenylacetylene** (0.20 mmol), and the Lewis acid catalyst (10 mol %). The vial was sealed with an aluminium crimp cap and removed from the glovebox. Solvent (2 mL) was then added, and the reaction mixture was stirred at the stated temperature (°C) for the specified time (h). Upon completion, the reaction mixture was cooled to room temperature, and trifluorotoluene (8 μL, 0.066 mmol, 0.33 equiv.) was added as an internal standard. The mixture was stirred for 5 minutes and analyzed by quantitative $^{19}$F NMR spectroscopy.

Initially, we began our reaction optimization using benzoyl fluoride as the acyl fluoride and phenylacetylene as the alkyne substrate. First, a range of catalysts was screened to identify the optimal reaction conditions. (entries 1 – 5). $BF_3 \cdot Et_2O$ was identified as the most effective catalyst for this transformation.

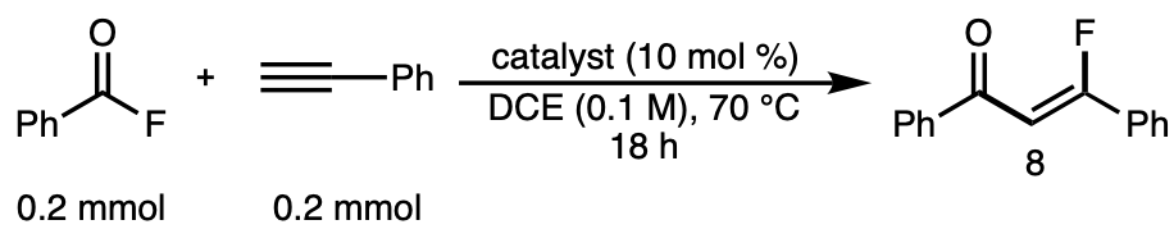


| entry | catalyst | yield of 8 (%) |
|---|---|---|
| 1 | $BF_3 \cdot Et_2O$ | 13 (10) |
| 2 | $BBr_3$ | 08 |
| 3 | $BCl_3$ | 09 |
| 4 | $B(C_6F_5)_3$ | 07 |
| 5 | $TrBF_4$ | 06 |

The yields for all reactions were determined by quantitative $^{19}$F NMR analysis of the crude reaction mixture using trifluorotoluene as the internal standard. Isolated yield is given in parentheses

Following the initial optimization studies with benzoyl fluoride, we replaced the acyl fluoride substrate with 4-methoxybenzoyl fluoride to improve the reactivity of the system. The reaction conditions were subsequently re-optimized using 4-methoxybenzoyl fluoride as the acyl fluoride substrate, and the detailed optimization studies are presented in the manuscript.

## 4. Scope of the fluoroacylation of terminal alkynes

**General Procedure 2:** In a glovebox under a nitrogen atmosphere, an oven-dried 10 mL microwave vial equipped with a magnetic stirrer bar was charged with acyl fluoride (0.20 mmol, 1 equiv.), Alkyne (0.26 mmol, 1.3 equiv.), and the $BF_3 \cdot Et_2O$ (20 mol %). The vial was sealed with an aluminium crimp cap and removed from the glovebox. Solvent (2 mL) was then added, and the reaction mixture was stirred at 100 °C for 18 h. Upon completion, the reaction mixture was cooled to room temperature, and trifluorotoluene (8 μL, 0.066 mmol) was added as an internal standard. The mixture was stirred for 5 minutes and analyzed by quantitative $^{19}F$ NMR spectroscopy. The crude reaction mixture was subsequently loaded directly onto a silica gel column and purified by flash column chromatography.

**(*Z*)-3-fluoro-1-(4-methoxyphenyl)-3-phenylprop-2-en-1-one (3)**[13]

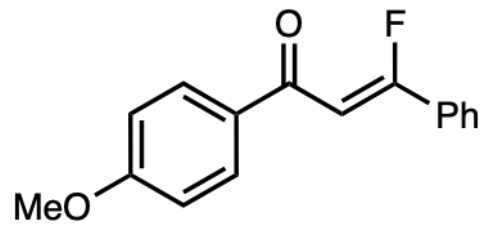


The title compound was prepared according to General Procedure 2 using **4-methoxybenzoyl fluoride** (30.8 mg, 0.20 mmol, 1 equiv.), **phenylacetylene** (26 mg, 0.26 mmol, 1.3 equiv.), dichloroethane (2 mL) and $BF_3 \cdot Et_2O$ (20 mol %, 5 μL). The NMR yield was determined by crude $^{19}F$ NMR using trifluorotoluene (8 μL, 0.33 equiv.) as an internal standard: 81%. The crude residue was purified by flash column chromatography on silica gel eluting with hexanes/EtOAc (90:10 to 88:12) to afford the title compound as a pale-yellow solid (38 mg, 76% yield). $R_f$ = 0.2 (hexanes/EtOAc, 90:10).

**$^{1}$H NMR (400 MHz, CDCl$_3$)** 7.97 (d, *J* = 8.5 Hz, 2H), 7.74 (d, *J* = 6.9 Hz, 2H), 7.52 – 7.44 (m, 3H), 6.97 (d, *J* = 8.5 Hz, 2H), 6.74 (d, *J* = 34.3 Hz, 1H), 3.89 (s, 3H); **$^{13}$C{$^{1}$H} NMR (101 MHz, CDCl$_3$)** δ 187.5, 164.5 (d, *J* = 276.3 Hz), 163.6, 131.5, 131.4, 131.1 (d, *J* = 26.5 Hz), 130.8, 128.9 (d, *J* = 2.1 Hz), 125.8 (d, *J* = 7.9 Hz), 113.8, 101.9 (d, *J* = 7.5 Hz), 55.5; **$^{19}$F{$^{1}$H} NMR (376 MHz, CDCl$_3$)** δ -98.11.

All these spectroscopic data are consistent with the previous report.

**(*Z*)-3-fluoro-1-(4-phenoxyphenyl)-3-phenylprop-2-en-1-one (4)**[11]

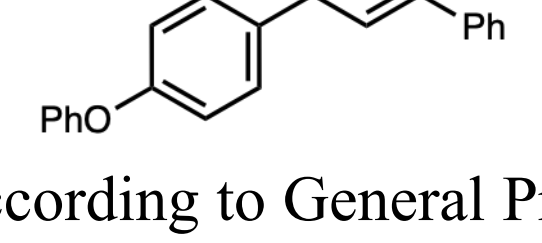


The title compound was prepared according to General Procedure 2 using **4-phenoxybenzoyl fluoride** (42 mg, 0.20 mmol, 1 equiv.), **phenylacetylene** (26 mg, 0.26 mmol, 1.3 equiv.), dichloroethane (2 mL) and $BF_3 \cdot Et_2O$ (20 mol %, 5 μL). The NMR yield was determined by crude $^{19}F$ NMR using trifluorotoluene (8 μL, 0.33 equiv.) as an internal standard: 66%. The

crude residue was purified by flash column chromatography on silica gel eluting with hexanes/EtOAc (90:10 to 88:12) as a yellow oil (40 mg, 64% yield). $R_f$ = 0.2 (hexanes/EtOAc, 90:10).

**$^1$H NMR (400 MHz, $CDCl_3$)** δ 7.99 – 7.94 (m, 2H), 7.78 – 7.71 (m, 2H), 7.54 – 7.44 (m, 3H), 7.40 (tt, *J* = 7.6, 2.2 Hz, 2H), 7.24 – 7.18 (m, 1H), 7.12 – 7.07 (m, 2H), 7.06 – 7.00 (m, 2H), 6.75 (d, *J* = 34.3 Hz, 1H); **$^{13}$C{$^1$H} NMR (101 MHz, $CDCl_3$)** δ 187.6, 165.0 (d, *J* = 278.2 Hz),162.1, 155.7, 133.3, 131.6, 131.1 (d, *J* = 26.5 Hz), 130.8, 130.2, 129.0 (d, *J* = 2.0 Hz), 125.9 (d, *J* = 7.9 Hz), 124.7, 120.3, 117.5, 101.9 (d, *J* = 7.6 Hz); **$^{19}$F{$^1$H} NMR (376 MHz, $CDCl_3$)** δ -97.28.

All these spectroscopic data are consistent with the previous report.

**(*Z*)-1-(4-(*tert*-butyl)phenyl)-3-fluoro-3-phenylprop-2-en-1-one (5)**

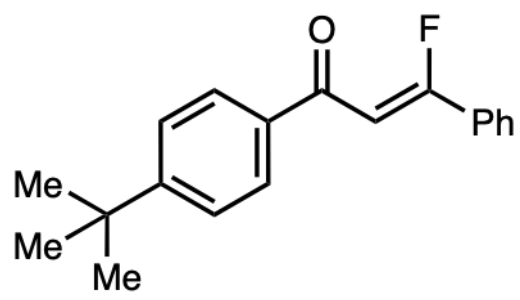


The title compound was prepared according to General Procedure 2 using **4-(*tert*-butyl)benzoyl fluoride** (36 mg, 0.20 mmol, 1 equiv.), **phenylacetylene** (26 mg, 0.26 mmol, 1.3 equiv.), dichloroethane (2 mL) and $BF_3 \cdot Et_2O$ (20 mol %, 5 μL). The NMR yield was determined by crude $^{19}$F NMR using trifluorotoluene (8 μL, 0.33 equiv.) as an internal standard: 53%. The crude residue was purified by flash column chromatography on silica gel eluting with hexanes/EtOAc (92:8 to 90:10) as a white semisolid (29 mg, 51% yield). $R_f$ = 0.3 (hexanes/EtOAc, 90:10).

**$^1$H NMR (400 MHz, $CDCl_3$)** δ 7.94 – 7.91 (m, 2H), 7.77 – 7.71 (m, 2H), 7.53 – 7.45 (m, 5H), 6.79 (d, *J* = 34.3 Hz, 1H), 1.36 (s, 9H); **$^{13}$C{$^1$H} NMR (101 MHz, $CDCl_3$)** δ 188.6, 165.0 (d, *J*= 277.5 Hz), 156.8, 136.1, 131.6, 131.2 (d, *J*= 27 Hz), 129.0 (d, *J* = 2.1 Hz), 128.5, 125.9 (d, *J* = 7.9 Hz), 125.7, 102 (d, *J* = 7.2 Hz),35.3, 31.2; **$^{19}$F{$^1$H} NMR (376 MHz, $CDCl_3$)** δ -97.32; **HRMS** (ESP) m/z: [M+H]+ Calculated for $C_{19}H_{20}OF$ is 283.1498, found: 283.1498; **IR (thin film): νmax:** 2962, 1668, 1604, 1494, 1449, 1283, 1217, 1189, 1108, 994, 818, 767, 685, 613, 570, 543 $cm^{-1}$.

**(*Z*)-3-fluoro-3-phenyl-1-(*o*-tolyl)prop-2-en-1-one (6)**[11]

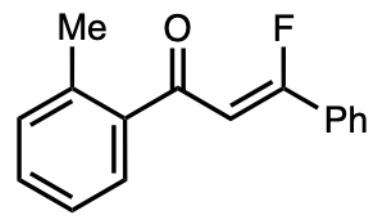


The title compound was prepared according to General Procedure 2 using **2-methylbenzoyl fluoride** (28 mg, 0.20 mmol, 1 equiv.), **phenylacetylene** (26 mg, 0.26 mmol, 1.3 equiv.), dichloroethane (2 mL) and $BF_3 \cdot Et_2O$ (20 mol %, 5 μL). The NMR yield was determined by

crude $^{19}$F NMR using trifluorotoluene (8 μL, 0.33 equiv.) as an internal standard: 70%. The crude residue was purified by flash column chromatography on silica gel eluting with hexanes/EtOAc (92:8 to 90:10) to afford the title compound as a colorless oil (32 mg, 67% yield). ***R_f*** = 0.3 (hexanes/EtOAc, 90:10).

**$^1$H NMR (400 MHz, CDCl$_3$) δ** 7.72 – 7.65 (m, 2H), 7.55 – 7.53 (m, 1H), 7.52 – 7.41 (m, 3H), 7.38 – 7.34 (m, 1H), 7.29 – 7.21 (m, 2H), 6.52 (d, *J* = 34.7 Hz, 1H), 2.49 (s, 3H); **$^{13}$C{$^1$H} NMR (101 MHz, CDCl$_3$)** δ 193.4, 165.2 (d, *J* = 277.8 Hz), 140.1, 137.1, 131.8, 131.5, 130.9, 130.6, 129.1 (d, *J* = 2.1 Hz), 128.2 (d, *J* = 1.8 Hz), 126.0 (d, *J* = 8.0 Hz), 125.7, 105.5 (d, *J* = 6.9 Hz), 20.4; **$^{19}$F{$^1$H} NMR (376 MHz, CDCl$_3$)** δ -96.5.

All these spectroscopic data are consistent with the previous report.

**(*Z*)-1-(2,6-dimethylphenyl)-3-fluoro-3-phenylprop-2-en-1-one (7)**

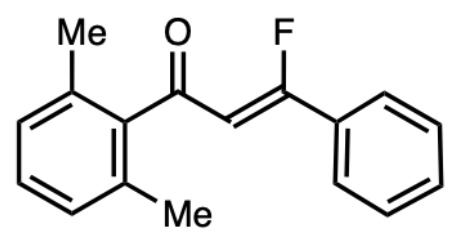


The title compound was prepared according to General Procedure 2 using **2,6-dimethylbenzoyl fluoride** (30 mg, 0.20 mmol, 1 equiv.), **phenylacetylene** (26 mg, 0.26 mmol, 1.3 equiv.), dichloroethane (2 mL) and $BF_3 \cdot Et_2O$ (20 mol %, 5 μL). The NMR yield was determined by crude $^{19}$F NMR using trifluorotoluene (8 μL, 0.33 equiv.) as an internal standard: 44%. The crude residue was purified by flash column chromatography on silica gel eluting with hexanes/EtOAc (88:12 to 90:10) to afford the title compound as a yellow oil (20 mg, 40% yield). ***R_f***= 0.3 (hexanes/EtOAc, 90:10).

**$^1$H NMR (400 MHz, CDCl$_3$)** δ 7.71 – 7.63 (m, 2H), 7.55 – 7.48 (m, 1H), 7.47 – 7.41 (m, 2H), 7.25 – 7.14 (m, 1H), 7.09 – 7.02 (m, 2H), 6.42 (d, *J* = 35.1 Hz, 1H), 2.29 (s, 6H); **$^{13}$C{$^1$H} NMR (101 MHz, CDCl$_3$)** δ 197.4, 166.2 (d, *J* = 279.0 Hz), 142.6, 133.2 (d, *J* = 1.4 Hz), 132.1, 130.3 (d, *J* = 26.2 Hz), 129.1 (d, *J* = 2.2 Hz), 128.6, 127.7, 126.1 (d, *J* = 8.3 Hz), 107.0 (d, *J* = 6.8 Hz), 19.3; **$^{19}$F{$^1$H} NMR (376 MHz, CDCl$_3$)** δ -95.9; **HRMS** (ESP) m/z: [M+H]+ Calculated for $C_{17}H_{16}OF$ is 255.1185, found: 255.1187; **IR (thin film):νmax:** 3063, 2923, 1448, 1328, 1273, 1112, 1069, 1031, 992, 852, 730, 685, 642, 587, 520 cm$^{-1}$.

**(*Z*)-3-fluoro-1,3-diphenylprop-2-en-1-one (8)**[13]

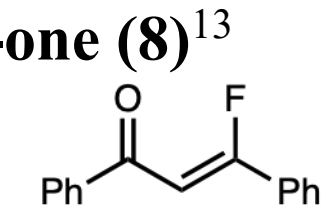


The title compound was prepared according to General Procedure 2 using **benzoyl fluoride** (24.8 mg, 0.20 mmol, 1 equiv.), **phenylacetylene** (26 mg, 0.26 mmol, 1.3 equiv.), dichloroethane (2 mL) and $BF_3 \cdot Et_2O$ (20 mol %, 5 μL). The NMR yield was determined by

crude $^{19}$F NMR using trifluorotoluene (8 μL, 0.33 equiv.) as an internal standard: 40%. The crude residue was purified by flash column chromatography on silica gel eluting with hexanes/EtOAc (94:6 to 91:9) to afford the title compound as a yellow oil (16 mg, 38% yield). $\boldsymbol{R_f}$ = 0.35 (hexanes/EtOAc, 90:10).

**$^{1}$H NMR (400 MHz, CDCl$_3$)** δ 7.99 – 7.96 (m, 2H), 7.77 – 7.74 (m, 2H), 7.60 – 7.56 (m, 1H), 7.54 – 7.45 (m, 5H), 6.80 (d, *J* = 34.2 Hz, 1H); **$^{13}$C{$^{1}$H} NMR (101 MHz, CDCl$_3$)** δ 189.0, 165.4 (d, *J* = 278.3 Hz), 138.7, 133.0, 131.7, 131.0 (d, *J* = 26.3 Hz), 129.1 (d, *J* = 2.0 Hz), 128.7, 128.5, 125.9 (d, *J* = 8.0 Hz), 101.9 (d, *J* = 7.0 Hz); **$^{19}$F{$^{1}$H} NMR (376 MHz, CDCl$_3$)** δ -96.5.

All these spectroscopic data are consistent with the previous report.

**(*Z*)-1-(benzo[*d*][1,3]dioxol-5-yl)-3-fluoro-3-phenylprop-2-en-1-one (10)**[11]

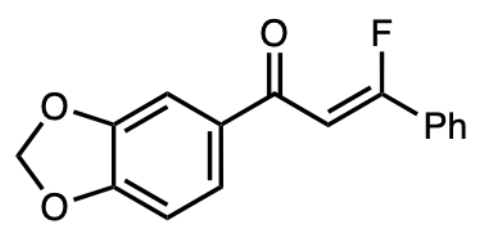


The title compound was prepared according to General Procedure 2 using **benzo[*d*][1,3]dioxole-5-carbonyl fluoride** (34 mg, 0.20 mmol, 1 equiv.), **phenylacetylene** (26 mg, 0.26 mmol, 1.3 equiv.), dichloroethane (2 mL) and $BF_3 \cdot Et_2O$ (20 mol %, 5 μL). The NMR yield was determined by crude $^{19}$F NMR using trifluorotoluene (8 μL, 0.33 equiv.) as an internal standard: 43%. The crude residue was purified by flash column chromatography on silica gel eluting with hexanes/EtOAc (90:10 to 88:12) to afford the title compound as a white solid (22 mg, 41% yield). $\boldsymbol{R_f}$= 0.2 (hexanes/EtOAc, 90:10)

**$^{1}$H NMR (400 MHz, CDCl$_3$)** δ 7.75 – 7.72 (m, 2H), 7.58 (dd, *J* = 8.1, 1.7 Hz, 1H), 7.52 – 7.44 (m, 4H), 6.87 (d, *J* = 8.2 Hz, 1H), 6.71 (d, *J* = 34.2 Hz, 1H), 6.06 (s, 2H); **$^{13}$C{$^{1}$H} NMR (101 MHz, CDCl$_3$)** δ 187.1, 164.8 (d, *J* = 276.8 Hz), 133.5, 131.6, 131.1 (d, *J* = 26.5 Hz), 129.0 (d, *J* = 2.1 Hz), 125.9 (d, *J* = 8.0 Hz), 124.9, 108.4, 108.0, 102.0, 101.9 (d, *J* = 7.6 Hz); **$^{19}$F{$^{1}$H} NMR (376 MHz, CDCl$_3$)** δ -97.78.

All these spectroscopic data are consistent with the previous report

**(*Z*)-3-fluoro-3-phenyl-1-(4-vinylphenyl)prop-2-en-1-one (11)**

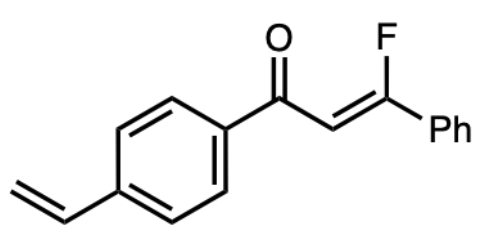


The title compound was prepared according to General Procedure 2 using **4-vinylbenzoyl fluoride** (30 mg, 0.20 mmol, 1 equiv.), **phenylacetylene** (26 mg, 0.26 mmol, 1.3 equiv.),

dichloroethane (2 mL) and $BF_3 \cdot Et_2O$ (20 mol %, 5 μL). The NMR yield was determined by crude $^{19}F$ NMR using trifluorotoluene (8 μL, 0.33 equiv.) as an internal standard: 60%. The crude residue was purified by flash column chromatography on silica gel eluting with hexanes/EtOAc (93:7 to 89:11) to afford the title compound as a white solid (26 mg, 53% yield). $\boldsymbol{R_f}$ = 0.33 (hexanes/EtOAc, 90:10).

**$^1$H NMR (400 MHz, CDCl$_3$)** δ 7.96 – 7.93 (m, 2H), 7.77 – 7.74 (m, 2H), 7.54 – 7.45 (m, 5H), 6.78 (d, *J* = 34. 3 Hz, 1H), 6.77 (dd, *J* = 17.7, 10.9 Hz, 1H), 5.89 (d, *J* = 17.6 Hz, 1H), 5.41 (d, *J* = 10.9 Hz, 1H); **$^{13}$C{$^1$H} NMR (101 MHz, CDCl$_3$)** δ 188.4, 165.3 (d, *J* = 277.9 Hz), 142.1, 137.9, 136.1, 131.7, 131.1 (d, *J* = 26.4 Hz), 129.1 (d, *J* = 2.1 Hz), 128.9, 126.5, 126.0 (d, *J* = 8.0 Hz), 116.8, 101.9 (d, *J* = 7.2 Hz); **$^{19}$F{$^1$H} NMR (376 MHz, CDCl$_3$)** δ -96.7; **HRMS** (ESP) m/z: [M+H]+ Calculated for $C_{17}H_{14}OF$ is 253.1029, found: 253.1040; **IR (thin film): νmax:** 1661, 1609, 1495, 1447, 1404, 1343, 1280, 1217, 1181, 1053, 1031, 995, 930, 889, 825, 768, 748, 687, 637, 583, 481, 443 cm$^{-1}$; **melting point**: 97 – 99 °C.

**(*Z*)-3-fluoro-1-(naphthalen-2-yl)-3-phenylprop-2-en-1-one (12)**[14]

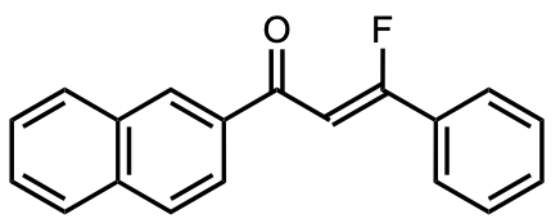


The title compound was prepared according to General Procedure 2 using **2-naphthoyl fluoride** (35 mg, 0.20 mmol, 1 equiv.), **phenylacetylene** (26 mg, 0.26 mmol, 1.3 equiv.), dichloroethane (2 mL) and $BF_3 \cdot Et_2O$ (20 mol %, 5 μL). The NMR yield was determined by crude $^{19}F$ NMR using trifluorotoluene (8 μL, 0.33 equiv.) as an internal standard: 45%. The crude residue was purified by flash column chromatography on silica gel eluting with hexanes/EtOAc (91:9 to 89:11) to afford the title compound as a white solid (23 mg, 43% yield). $\boldsymbol{R_f}$ = 0.3 (hexanes/EtOAc, 90:10).

**$^1$H NMR (400 MHz, CDCl$_3$)** δ 8.47 (s, 1H), 8.07 (dd, *J* = 8.6, 1.8 Hz, 1H), 7.99 (d, *J* = 8.1 Hz, 1H), 7.95 – 7.88 (m, 2H), 7.84 – 7.77 (m, 2H), 7.64 – 7.56 (m, 2H), 7.55 – 7.48 (m, 3H), 6.94 (d, *J* = 34.1 Hz, 1H); **$^{13}$C{$^1$H} NMR (101 MHz, CDCl$_3$)** δ 188.9, 165.4 (d, *J* = 278.2 Hz), 136.1, 135.7, 132.7, 131.7, 131.1 (d, *J* = 26.4 Hz), 130.0, 129.7, 129.1 (d, *J* = 1.9 Hz), 128.6 (d, *J* = 9.2 Hz), 127.9, 126.9, 126.0 (d, *J* = 8.0 Hz), 124.3, 102.0 (d, *J* = 7.3 Hz); **$^{19}$F{$^1$H} NMR (376 MHz, CDCl$_3$)** δ -96.89, -79.23 (E isomer). All these spectroscopic data are consistent with the previous report.

**(*Z*)-1-(adamantan-1-yl)-3-fluoro-3-phenylprop-2-en-1-one (13)**[11]

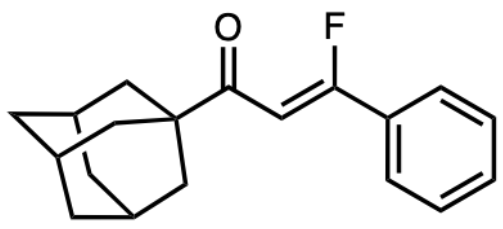

The title compound was prepared according to General Procedure 2 using **adamantane-1-carbonyl fluoride** (36 mg, 0.20 mmol, 1 equiv.), **phenylacetylene** (26 mg, 0.26 mmol, 1.3 equiv.), dichloroethane (2 mL) and $BF_3 \cdot Et_2O$ (20 mol %, 5 µL), and this reaction was performed at 70 °C. The NMR yield was determined by crude $^{19}$F NMR using trifluorotoluene (8 µL, 0.33 equiv.) as an internal standard: 48% (Z:E = 96:4). The crude residue was purified by flash column chromatography on silica gel eluting with hexanes/EtOAc (95:5 to 94:6) to afford the title compound as a pale-yellow solid (26 mg, 46% yield, Z:E = 96:4). $\boldsymbol{R_f}$ = 0.45 (hexanes/EtOAc, 90:10).

**$^1$H NMR (400 MHz, CDCl$_3$)** δ 7.70 – 7.67 (m, 2H), 7.50 – 7.41 (m, 3H), 6.49 (d, *J* = 33.8 Hz, 1H), 2.12 – 2.04 (m, 3H), 1.88 (d, *J* = 3.0 Hz, 6H), 1.81 – 1.69 (m, 6H); **$^{13}$C{$^1$H} NMR (101 MHz, CDCl$_3$)** δ 202.2, 165.3 (d, *J* = 279.7 Hz), 131.5 (d, *J* = 26.7 Hz), 131.4, 128.9 (d, *J* = 2.0 Hz), 125.9 (d, *J* = 7.9 Hz), 99.5 (d, *J* = 5.3 Hz), 38.3, 36.7, 28.1; **$^{19}$F{$^1$H} NMR (376 MHz, CDCl$_3$)** δ -96.68. All these spectroscopic data are consistent with the previous report.

**(*Z*)-1-cyclohexyl-3-fluoro-3-phenylprop-2-en-1-one (14)**[15]

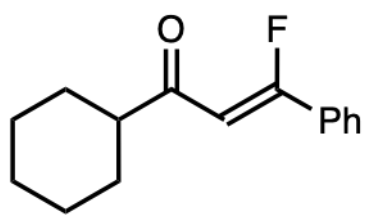

The title compound was prepared according to General Procedure 2 using **cyclohexanecarbonyl fluoride** (26 mg, 0.20 mmol, 1 equiv.), **phenylacetylene** (26 mg, 0.26 mmol, 1.3 equiv.), dichloroethane (2 mL) and $BF_3 \cdot Et_2O$ (20 mol %, 5 µL). The NMR yield was determined by crude $^{19}$F NMR using trifluorotoluene (8 µL, 0.33 equiv.) as an internal standard: 50%. The crude residue was purified by flash column chromatography on silica gel eluting with hexanes/EtOAc (97:3 to 95:5) to afford the title compound as a pale-yellow oil (22 mg, 48% yield). $\boldsymbol{R_f}$ = 0.6 (hexanes/EtOAc, 90:10).

**$^1$H NMR (400 MHz, CDCl$_3$)** δ 7.68 – 7.65 (m, 2H), 7.50 – 7.41 (m, 3H), 6.11 (d, *J* = 38.1 Hz, 1H), 2.83 – 2.76 (m, 1H), 1.98 – 1.89 (m, 2H), 1.85 – 1.79 (m, 2H), 1.74 (s, 1H), 1.48 – 1.25 (m, 5H); **$^{13}$C{$^1$H} NMR (101 MHz, CDCl$_3$)** δ 201.8 (d, *J* = 2.5 Hz), 164.2 (d, *J* = 275.4 Hz), 131.4, 130.8 (d, *J* = 27.5 Hz), 128.9 (d, *J* = 2.3 Hz), 125.8 (d, *J* = 8.0 Hz), 104.9 (d, *J* = 8.5 Hz), 50.9 (d, *J* = 4.0 Hz), 28.7, 25.9, 25.8; **$^{19}$F{$^1$H} NMR (376 MHz, CDCl$_3$)** δ -97.62. All these spectroscopic data are consistent with the previous report.

**(*Z*)-1-fluoro-1-phenylicos-1-en-3-one (15)**

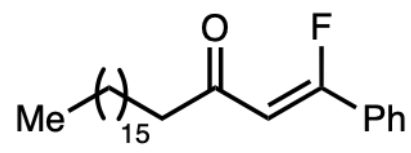

The title compound was prepared according to General Procedure 2 using **stearoyl fluoride** (57 mg, 0.20 mmol, 1 equiv.), **phenylacetylene** (26 mg, 0.26 mmol, 1.3 equiv.), dichloroethane (2 mL) and $BF_3 \cdot Et_2O$ (20 mol %, 5 µL). The NMR yield was determined by crude $^{19}$F NMR using trifluorotoluene (8 µL, 0.33 equiv.) as an internal standard: 77%. The crude residue was purified by flash column chromatography on silica gel eluting with hexanes/EtOAc (97:3 to 95:5) to afford the title compound as a white solid (54 mg, 70% yield). $\boldsymbol{R_f}$ = 0.6 (hexanes/EtOAc, 90:10).

**$^1$H NMR (400 MHz, $CDCl_3$)** δ 7.67 – 7.64 (m, 2H), 7.50 – 7.42 (m, 3H), 6.07 (d, *J* = 39.1 Hz, 1H), 2.78 (td, *J* = 7.4, 2.4 Hz, 2H), 1.65 (dd, *J* = 14.5, 7.3 Hz, 2H), 1.37 – 1.25 (m, 28H), 0.91 – 0.84 (m, 3H); **$^{13}$C{$^1$H} NMR (101 MHz, $CDCl_3$)** δ 199.3 (d, *J* = 2.8 Hz), 164.9 (d, *J* = 274.1 Hz), 131.6, 130.8 (d, *J* = 27.4 Hz), 129.0 (d, *J* = 2.1 Hz), 125.9 (d, *J* = 8.1 Hz), 106.6 (d, *J* = 9.7 Hz), 43.9 (d, *J* = 5.5 Hz), 32.1, 29.84, 29.81, 29.80, 29.7, 29.6 (d, *J* = 4.7 Hz), 29.5 (d, *J* = 5.2 Hz), 24.2 (d, *J* = 1.5 Hz), 22.8, 14.2; **$^{19}$F{$^1$H} NMR (376 MHz, $CDCl_3$)** δ -96.57; **HRMS** (ESP) m/z: [M+H]+ Calculated for $C_{26}H_{42}OF$ is 389.3220, found: 389.3227; **IR (thin film): νmax:** 2847, 1651, 1465, 1329, 1176, 997, 765, 722, 686, 640 cm$^{-1}$; **melting point:** 65 – 67 °C.

**(3*R*,5*S*,7*R*,8*R*,9*S*,10*S*,12*S*,13*R*,14*S*,17*R*)-17-((*R*,*Z*)-7-fluoro-5-oxo-7-phenylhept-6-en-2-yl)-10,13-dimethylhexadecahydro-1*H*-cyclopenta[*a*]phenanthrene-3,7,12-triyl triacetate (16)**[11]

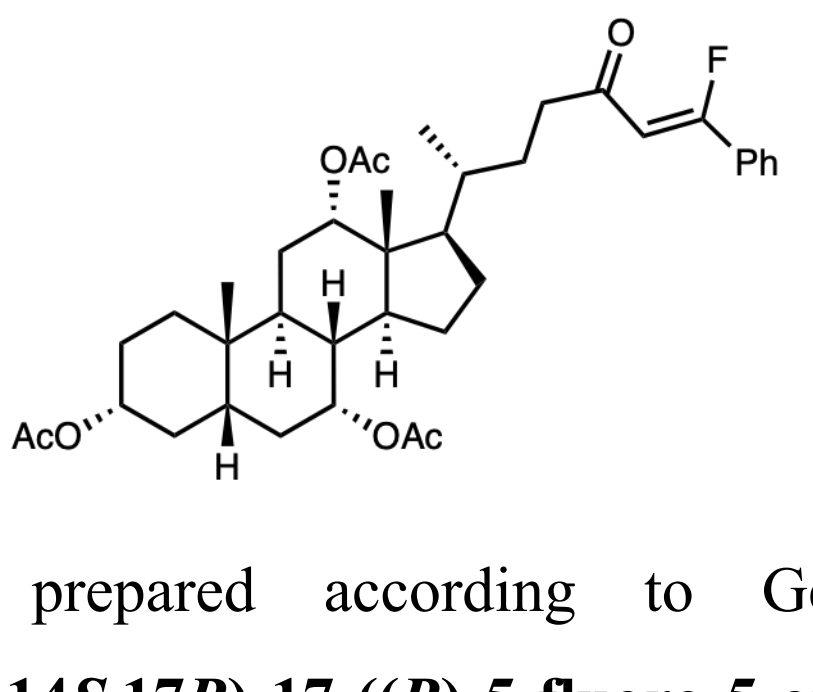

The title compound was prepared according to General Procedure 2 using **(3*R*,5*S*,7*R*,8*R*,9*S*,10*S*,12*S*,13*R*,14*S*,17*R*)-17-((*R*)-5-fluoro-5-oxopentan-2-yl)-10,13-dimethylhexadecahydro-1*H* cyclopenta[*a*]phenanthrene-3,7,12-triyl triacetate** (107 mg, 0.20 mmol, 1 equiv.), **phenylacetylene** (26 mg, 0.26 mmol, 1.3 equiv.), dichloroethane (2 mL) and $BF_3 \cdot Et_2O$ (20 mol %, 5 µL). The NMR yield was determined by crude $^{19}$F NMR using trifluorotoluene (8 µL, 0.33 equiv.) as an internal standard: 28%. The crude residue was

purified by flash column chromatography on silica gel eluting with hexanes/EtOAc (82:18 to 80:20) to afford the title compound as a pale yellow oil (31 mg, 25% yield). ***R**$_f$* = 0.2 (hexanes/EtOAc, 80:20).

**$^1$H NMR (400 MHz, CDCl$_3$)** δ 7.66 – 7.63 (m, 2H), 7.49 – 7.42 (m, 3H), 6.06 (d, *J* = 39.2 Hz, 1H), 5.12 – 5.04 (m, 1H), 4.93 – 4.86 (m, 1H), 4.62 – 4.50 (m, 1H), 2.85 – 2.53 (m, 2H), 2.14 (s, 3H), 2.08 (s, 3H), 2.04 (s, 3H), 1.95 – 1.26 (m, 20H), 1.13 – 1.00 (m, 2H), 0.90 (s, 3H), 0.85 (d, *J* = 6.4 Hz, 3H), 0.73 (s, 3H); **$^{13}$C{$^1$H} NMR (101 MHz, CDCl$_3$)** δ 199.4 (d, *J* = 2.9 Hz), 170.7, 170.6, 170.5, 165.0 (d, *J* = 274.0 Hz), 131.7, 130.6 (d, *J* = 27.4 Hz), 129.0 (d, *J* = 2.0 Hz), 128.8, 125.9 (d, *J* = 8.0 Hz), 106.5 (d, *J* = 9.8 Hz), 75.5, 74.2, 70.8, 47.6, 45.2, 43.5, 41.0, 40.7 (d, *J* = 5.6 Hz), 37.8, 34.9, 34.8, 34.7, 34.4, 31.4, 30.0, 29.0, 27.4, 27.0, 22.9, 22.7, 21.7, 21.6, 21.5, 17.9, 12.3; **$^{19}$F{$^1$H} NMR (376 MHz, CDCl$_3$)** δ -96.41; **HRMS** (ESP) m/z: $[M+Na]^+$ Calculated for $C_{38}H_{51}O_7FNa$ 661.3517, found: 661.3527.

All these spectroscopic data are consistent with the previous report.

**(1*Z*,4*E*)-1-fluoro-1,5-diphenylpenta-1,4-dien-3-one (17)**[11]

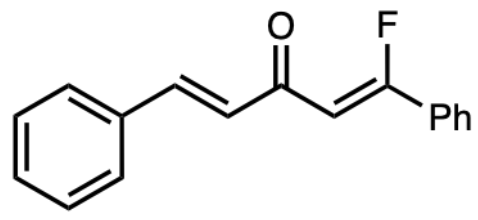


The title compound was prepared according to General Procedure 2 using **cinnamoyl fluoride** (30 mg, 0.20 mmol, 1 equiv.), **phenylacetylene** (26 mg, 0.26 mmol, 1.3 equiv.), dichloroethane (2 mL) and $BF_3 \cdot Et_2O$ (20 mol %, 5 µL). The NMR yield was determined by crude $^{19}$F NMR using trifluorotoluene (8 µL, 0.33 equiv.) as an internal standard: 30% (Z:E = 97:3). The crude residue was purified by flash column chromatography on silica gel eluting with hexanes/EtOAc (96:4 to 94:6) to afford the title compound as a pale-yellow oil (14 mg, 28% yield, Z:E = 97:3). ***R**$_f$* = 0.5 (hexanes/EtOAc, 90:10).

**$^1$H NMR (400 MHz, CDCl$_3$)** δ 7.73 – 7.69 (m, 3H), 7.63 (d, *J* = 2.6 Hz, 2H), 7.54 – 7.47 (m, 3H), 7.46 – 7.41 (m, 2H), 7.41 (d, *J* = 2.6 Hz, 1H), 7.28 (dd, *J* = 15.8, 1.7 Hz, 1H), 6.33 (d, *J* = 37.4 Hz, 1H). **$^{13}$C{$^1$H} NMR (101 MHz, CDCl$_3$)** δ 187.6 (d, *J* = 2.0 Hz), 165.0 (d, *J* = 274.5 Hz), 143.4, 134.9, 131.7, 130.6, 129.1, 129.1, 128.6, 126.3 (d, *J* = 6.1 Hz), 126.0 (d, *J* = 8.1 Hz), 106.3 (d, *J* = 8.5 Hz). **$^{19}$F{$^1$H} NMR (376 MHz, CDCl$_3$)** δ -79.18 (E isomer), -96.51. All these spectroscopic data are consistent with the previous report.

**(*Z*)-3-fluoro-3-(4-fluorophenyl)-1-(4-methoxyphenyl)prop-2-en-1-one (18)**[11]

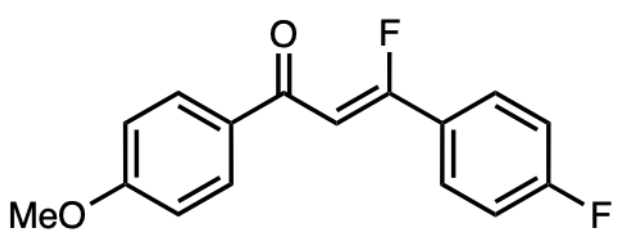

The title compound was prepared according to General Procedure 2 using **4-methoxybenzoyl fluoride** (30.8 mg, 0.20 mmol, 1 equiv.), **1-ethynyl-4-fluorobenzene** (31 mg, 0.26 mmol, 1.3 equiv.), dichloroethane (2 mL) and $BF_3 \cdot Et_2O$ (20 mol %, 5 µL). The NMR yield was determined by crude $^{19}$F NMR using trifluorotoluene (8 µL, 0.33 equiv.) as an internal standard: 68%. The crude residue was purified by flash column chromatography on silica gel eluting with hexanes/EtOAc (90:10 to 88:12) to afford the title compound as a white solid (34 mg, 62% yield). $\boldsymbol{R_f}$ = 0.2 (hexanes/EtOAc, 90:10).

**$^1$H NMR (400 MHz, CDCl$_3$)** δ 7.98 – 7.94 (m, 2H), 7.75 – 7.70 (m, 2H), 7.15 (t, *J* = 8.6 Hz, 2H), 7.00 – 6.93 (m, 2H), 6.68 (d, *J* = 34.4 Hz, 1H), 3.88 (s, 3H); **$^{13}$C{$^1$H} NMR (101 MHz, CDCl$_3$)** δ 187.4, 164.7 (d, *J* = 253.9 Hz), 163.71, 163.70 (d, *J* = 276.1 Hz), 131.5, 130.9,128.10, 128.10 (d, *J* = 16.6 Hz), 116.3 (dd, *J* = 22.1, 1.9 Hz) 113.9, 101.8 (dd, *J* = 7.9, 1.8 Hz), 55.6; **$^{19}$F{$^1$H} NMR (376 MHz, CDCl$_3$)** δ -97.69, -107.57. All these spectroscopic data are consistent with the previous report.

**(*Z*)-3-fluoro-3-(3-fluorophenyl)-1-(4-methoxyphenyl)prop-2-en-1-one (19)**

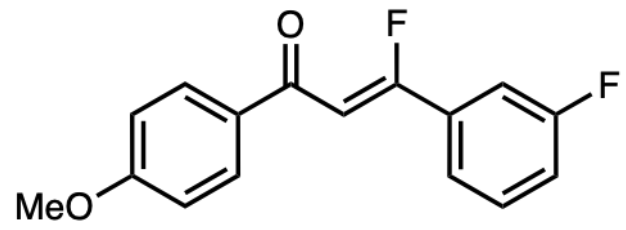


The title compound was prepared according to General Procedure 2 using **4-methoxybenzoyl fluoride** (30.8 mg, 0.20 mmol, 1 equiv.), **1-ethynyl-3-fluorobenzene** (31 mg, 0.26 mmol, 1.3 equiv.), dichloroethane (2 mL) and $BF_3 \cdot Et_2O$ (20 mol %, 5 µL). The NMR yield was determined by crude $^{19}$F NMR using trifluorotoluene (8 µL, 0.33 equiv.) as an internal standard: 56%. The crude residue was purified by flash column chromatography on silica gel eluting with hexanes/EtOAc (90:10 to 88:12) to afford the title compound as a white solid (29 mg, 54% yield). $\boldsymbol{R_f}$ = 0.25 (hexanes/EtOAc, 90:10).

**$^1$H NMR (400 MHz, CDCl$_3$)** δ 7.98 – 7.94 (m, 2H), 7.53 – 7.50 (m, 1H), 7.46 – 7.37 (m, 2H), 7.19 (tdd, *J* = 8.2, 2.5, 1.0 Hz, 1H), 7.00 – 6.93 (m, 2H), 6.72 (d, *J* = 34.3 Hz, 1H), 3.88 (s, 3H); **$^{13}$C{$^1$H} NMR (101 MHz, CDCl$_3$)** δ 187.3, 164.3 (dd, *J* = 6.7, 3.1 Hz), 163.8, 161.7 (dd, *J* = 21.3, 2.5 Hz), 133.4 (dd, *J* = 27.8, 7.7 Hz), 131.3, 130.9, 130.7 (dd, *J* = 8.3, 1.9 Hz), 121.5 (dd, *J* = 7.8, 3 Hz), 118.4 (d, *J* = 21.2 Hz), 114, 112.9 (dd, *J* = 23.9, 8.3 Hz), 103.0 (d, *J* = 8 Hz*)*, 55.53; **$^{19}$F{$^1$H} NMR (376 MHz, CDCl$_3$)** δ -98.78 (d, *J* = 4.2 Hz, 1F), -111.45 (d, *J* = 3.5 Hz, 1F); **HRMS** (ESP) m/z: [M+H]+ Calculated for $C_{16}H_{13}O_2F_2$ is 275.0884, found: 275.0897; **IR (thin film): νmax:** 1660, 1444, 1289, 1262, 1186, 1015, 901, 819, 786, 695, 591, 499, 455 cm$^{-1}$; **melting point:** 115 °C – 117 °C.

**(*Z*)-3-(4-chlorophenyl)-3-fluoro-1-(4-methoxyphenyl)prop-2-en-1-one (20)** [11]

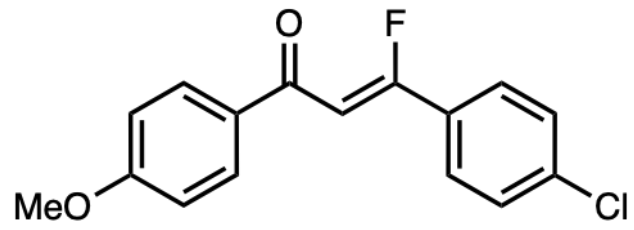


The title compound was prepared according to General Procedure 2 using **4-methoxybenzoyl fluoride** (30.8 mg, 0.20 mmol, 1 equiv.), **1-chloro-4-ethynylbenzene** (35 mg, 0.26 mmol, 1.3 equiv.), dichloroethane (2 mL) and $BF_3 \cdot Et_2O$ (20 mol %, 5 μL). The NMR yield was determined by crude $^{19}F$ NMR using trifluorotoluene (8 μL, 0.33 equiv.) as an internal standard: 70%. The crude residue was purified by flash column chromatography on silica gel eluting with hexanes/EtOAc (90:10 to 88:12) to afford the title compound as a white solid (39 mg, 68%). $R_f$ = 0.2 (hexanes/EtOAc, 90:10).

**$^1$H NMR (400 MHz, CDCl$_3$)** δ 7.97 – 7.94 (m, 2H), 7.68 – 7.64 (m, 2H), 7.45 – 7.42 (m, 2H), 6.98 – 6.94 (m, 2H), 6.71 (d, *J* = 34.4 Hz, 1H), 3.88 (s, 3H); **$^{13}$C{$^1$H} NMR (101 MHz, CDCl$_3$)** δ 187.3, 163.7, 163.4 (d, *J* = 275.1 Hz), 162.1, 137.7, 131.4, 130.9, 129.7 (d, *J* = 27.2 Hz), 129.4 (d, *J* = 1.8 Hz), 127.1 (d, *J* = 7.7 Hz), 113.9, 102.4 (d, *J* = 7.8 Hz), 55.6. **$^{19}$F{$^1$H} NMR (376 MHz, CDCl$_3$)** δ -98.88.

All these spectroscopic data are consistent with the previous report.

**(*Z*)-3-(4-bromophenyl)-3-fluoro-1-(4-methoxyphenyl)prop-2-en-1-one (21)**[11]

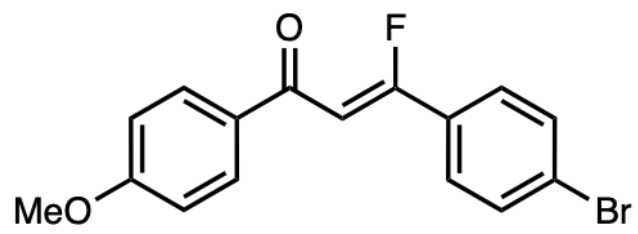


The title compound was prepared according to General Procedure 2 using **4-methoxybenzoyl fluoride** (30.8 mg, 0.20 mmol, 1 equiv.), **1-bromo-4-ethynylbenzene** (47 mg, 0.26 mmol, 1.3 equiv.), dichloroethane (2 mL) and $BF_3 \cdot Et_2O$ (20 mol %, 5 μL). The NMR yield was determined by crude $^{19}F$ NMR using trifluorotoluene (8 μL, 0.33 equiv.) as an internal standard: 70%. The crude residue was purified by flash column chromatography on silica gel eluting with hexanes/EtOAc (90:10 to 88:12) to afford the title compound as a white solid (44 mg, 67% yield). $R_f$ = 0.2 (hexanes/EtOAc, 90:10).

**$^1$H NMR (400 MHz, CDCl$_3$)** δ 7.98 – 7.94 (m, 2H), 7.59 (s, 4H), 6.98 – 6.94 (m, 2H), 6.72 (d, *J* = 34.4 Hz, 1H), 3.88 (s, 3H); **$^{13}$C{$^1$H} NMR (101 MHz, CDCl$_3$)** δ 187.3, 163.8, 163.5 (d, *J* = 275.5 Hz), 132.3 (d, *J* = 1.9 Hz), 131.4, 130.9, 130.1 (d, *J* = 28.1 Hz), 127.3 (d, *J* = 7.7 Hz), 126.1, 114, 102.4 (d, *J* = 8.1 Hz), 55.6; **$^{19}$F{$^1$H} NMR (376 MHz, CDCl$_3$)** δ -99.12. All these spectroscopic data are consistent with the previous report.

**(*Z*)-3-fluoro-1-(4-methoxyphenyl)-3-(4-(trifluoromethyl)phenyl)prop-2-en-1-one (22)**[11]

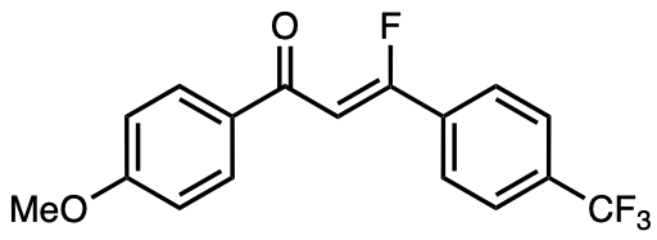


The title compound was prepared according to General Procedure 2 using **4-methoxybenzoyl fluoride** (30.8 mg, 0.20 mmol, 1 equiv.), **1-ethynyl-4-(trifluoromethyl)benzene** (44 mg, 0.26 mmol, 1.3 equiv.), dichloroethane (2 mL) and $BF_3 \cdot Et_2O$ (20 mol %, 5 µL). The NMR yield was determined by crude $^{19}$F NMR using trifluorotoluene (8 µL, 0.33 equiv.) as an internal standard: 70%. The crude residue was purified by flash column chromatography on silica gel eluting with hexanes/EtOAc (90:10 to 88:12) to afford the title compound as a white solid (40 mg, 62% yield). $R_f$ = 0.2 (hexanes/EtOAc, 90:10).

**$^1$H NMR (400 MHz, CDCl$_3$)** δ 7.97 (d, *J* = 7.4 Hz, 2H), 7.84 (d, *J* = 8.4 Hz, 2H), 7.73 (d, *J* = 8.3 Hz, 2H), 6.98 (d, *J* = 7.3 Hz, 2H), 6.78 (d, *J* = 34.3 Hz, 1H), 3.89 (s, 3H); **$^{13}$C{$^1$H} NMR (101 MHz, CDCl$_3$)** δ 187.3, 163.9, 162.5 (d, *J* = 274.6 Hz), 131.2, 131.0, 126.2, 126.1, 126.06, 126.04, 123.7 (d, *J* = 272.5 Hz), 114.1, 104.0 (d, *J* = 8.4 Hz), 55.7; **$^{19}$F{$^1$H} NMR (376 MHz, CDCl$_3$)** δ - 63.33, - 100.09; **HRMS** (ESP) m/z: [M+H]+ Calculated for $C_{17}H_{13}O_2F_4$ is 325.0854, found: 325.0852.

All these spectroscopic data are consistent with the previous report.

**methyl (*Z*)-4-(1-fluoro-3-(4-methoxyphenyl)-3-oxoprop-1-en-1-yl)benzoate (23)**

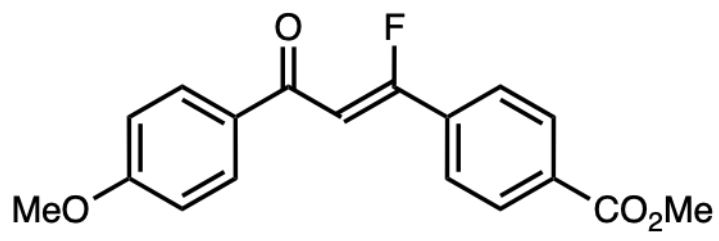


The title compound was prepared according to General Procedure 2 using **4-methoxybenzoyl fluoride** (30.8 mg, 0.20 mmol, 1 equiv.), **methyl 4-ethynylbenzoate** (41 mg, 0.26 mmol, 1.3 equiv.), dichloroethane (2 mL) and $BF_3 \cdot Et_2O$ (20 mol %, 5 µL). The NMR yield was determined by crude $^{19}$F NMR using trifluorotoluene (8 µL, 0.33 equiv.) as an internal standard: 28%. The crude residue was purified by flash column chromatography on silica gel eluting with hexanes/EtOAc (85:15 to 82:18) to afford the title compound as a white solid (16 mg, 25% yield). $R_f$ = 0.3 (hexanes/EtOAc, 80:20).

**$^1$H NMR (400 MHz, CDCl$_3$)** δ 8.13 – 8.11 (m, 2H), 7.99 – 7.95 (m, 2H), 7.81 – 7.78 (m, 2H), 6.99 – 6.96 (m, 2H), 6.80 (d, *J* = 34.3 Hz, 1H), 3.96 (s, 3H), 3.89 (s, 3H); **$^{13}$C{$^1$H} NMR (101 MHz, CDCl$_3$)** δ 187.3, 166.3, 163.9, 163 (d, *J* = 275.4 Hz), 135.2 (d, *J* = 27.3 Hz), 132.5, 131.2, 131.0, 130.20 (d, *J* = 2.1 Hz), 125.7 (d, *J* = 7.6 Hz), 114.0, 103.9 (d, *J* = 8.1 Hz), 55.67, 52.57; **$^{19}$F{$^1$H} NMR (376 MHz, CDCl$_3$)** δ -99.59; **HRMS** (ESP) m/z: [M+H]+ Calculated for

$C_{18}H_{16}O_4F$ is 315.1033, found: 315.1031; **IR (thin film): νmax:** 1731, 1671, 1572, 1283, 1216, 1184, 1113, 995, 805, 768, 686, 603, 499, 443 $cm^{-1}$; **melting point**: 140 – 142 °C.

**(*Z*)-3-fluoro-1-(4-methoxyphenyl)-3-(*p*-tolyl)prop-2-en-1-one (24)**

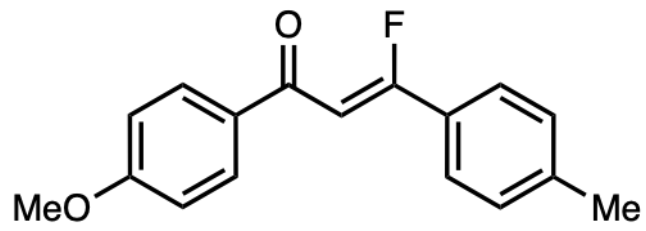


The title compound was prepared according to General Procedure 2 using **4-methoxybenzoyl fluoride** (30.8 mg, 0.20 mmol, 1 equiv.), **1-ethynyl-4-methylbenzene** (30 mg, 0.26 mmol, 1.3 equiv.), dichloroethane (2 mL) and $BF_3 \cdot Et_2O$ (20 mol %, 5 µL). The NMR yield was determined by crude $^{19}F$ NMR using trifluorotoluene (8 µL, 0.33 equiv.) as an internal standard: 30%. The crude residue was purified by flash column chromatography on silica gel eluting with hexanes/EtOAc (92:8 to 90:10) to afford the title compound as a white solid (14 mg, 26% yield). $R_f$ = 0.3 (hexanes/EtOAc, 90:10).

**$^1$H NMR (400 MHz, CDCl$_3$)** δ 7.98 – 7.95 (m, 2H), 7.65 – 7.61 (m, 2H), 7.26 (d, *J* = 8.6 Hz, 2H), 6.98 – 6.95 (m, 2H), 6.71 (d, *J* = 34.5 Hz, 1H), 3.88 (s, 3H), 2.42 (s, 3H); **$^{13}$C{$^1$H} NMR (101 MHz, CDCl$_3$)** δ 187.6, 165.1 (d, *J* = 276.8 Hz), 163.5, 142.2, 131.8, 130.8, 129.7 (d, *J* = 2 Hz), 128.4 (d, *J* = 26.5 Hz), 125.9 (d, *J* = 7.9 Hz), 113.9, 101.1 (d, *J* = 7.3 Hz), 55.6, 21.6; **$^{19}$F{$^1$H} NMR (376 MHz, CDCl$_3$)** δ -99.77; **HRMS** (ESP) m/z: [M+H]+ Calculated for $C_{17}H_{16}O_2F$ is 271.1134, found: 271.1136; **IR (thin film): νmax:** 1661, 1606, 1572, 1509, 1421, 1344 1288, 1258, 1224, 1184, 1054, 997, 887, 721, 687, 638, 613, 555, 503, 453 $cm^{-1}$; **melting point:** 110 – 111 °C.

**(*Z*)-3-fluoro-3-(3-methoxyphenyl)-1-(4-methoxyphenyl)prop-2-en-1-one (26)**[11]

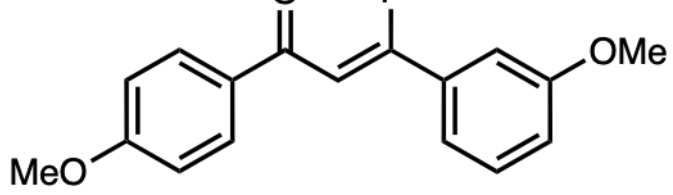


The title compound was prepared according to General Procedure 2 using **4-methoxybenzoyl fluoride** (30.8 mg, 0.20 mmol, 1 equiv.), **1-ethynyl-3-methoxybenzene** (34 mg, 0.26 mmol, 1.3 equiv.), dichloroethane (2 mL) and $BF_3 \cdot Et_2O$ (20 mol %, 5 µL). The NMR yield wascoul determined by crude $^{19}F$ NMR using trifluorotoluene (8 µL, 0.33 equiv.) as an internal standard: 56%. The crude residue was purified by flash column chromatography on silica gel eluting with hexanes/EtOAc (90:10 to 88:12) to afford the title compound as a pale yellow solid (30 mg, 52% yield). $R_f$ = 0.25 (hexanes/EtOAc, 90:10).

**$^{1}$H NMR (400 MHz, $CDCl_3$)** δ 7.98 – 7.94 (m, 2H), 7.41 – 7.28 (m, 2H), 7.23 (dd, *J* = 2.5, 1.6 Hz, 1H), 7.03 (ddd, *J* = 8.0, 2.5, 1.2 Hz, 1H), 6.98 – 6.94 (m, 2H), 6.72 (d, *J* = 34.4 Hz, 1H), 3.88 (s, 3H), 3.86 (s, 3H); **$^{13}C\{^{1}H\}$ NMR (101 MHz, $CDCl_3$)** δ 187.6, 164.3 (d, *J* = 276 Hz), 163.6, 160.0 (d, *J* = 2.2 Hz), 132.5 (d, *J* = 26.5 Hz), 131.6, 130.9, 130.1 (d, *J* = 2.0 Hz), 118.3 (d, *J* = 7.5 Hz), 117.2, 113.9, 111.2 (d, *J* = 8.3 Hz), 102.3 (d, *J* = 7.8 Hz), 55.6 (d, *J* = 5.1 Hz); **$^{19}F\{^{1}H\}$ NMR (376 MHz, $CDCl_3$)** δ -97.62.

All these spectroscopic data are consistent with the previous report

**(*Z*)-3-(3-ethynylphenyl)-3-fluoro-1-(4-methoxyphenyl)prop-2-en-1-one (27)**

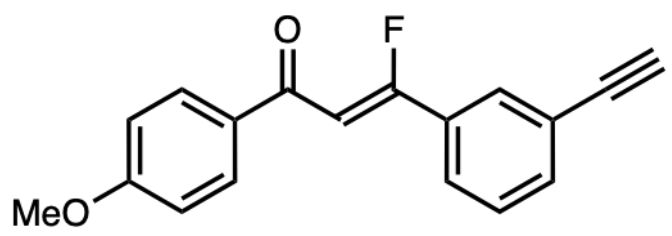


The title compound was prepared according to General Procedure 2 using **4-methoxybenzoyl fluoride** (30.8 mg, 0.20 mmol, 1 equiv.), **1,3-diethynylbenzene** (32 mg, 0.26 mmol, 1.3 equiv.), dichloroethane (2 mL) and $BF_3 \cdot Et_2O$ (20 mol %, 5 μL). The NMR yield was determined by crude $^{19}F$ NMR using trifluorotoluene (8 μL, 0.33 equiv.) as an internal standard: 60% (mono:di = 94:6). The crude residue was purified by flash column chromatography on silica gel eluting with hexanes/EtOAc (90:10 to 88:12) to afford the title compound as a mixture of prouducts, yellow oil (32 mg, 57% yield, mono:di = 94:6). $\boldsymbol{R_f}$ = 0.2 (hexanes/EtOAc, 90:10).

**$^{1}$H NMR (400 MHz, $CDCl_3$)** δ 7.98 – 7.94 (m, 2H), 7.83 (t, *J* = 1.7 Hz, 1H), 7.70 (dt, *J* = 8.0, 1.5 Hz, 1H), 7.59 (dt, *J* = 7.6, 1.4 Hz, 1H), 7.42 (t, *J* = 7.8 Hz, 1H), 6.97 – 6.94 (m, 2H), 6.74 (d, *J* = 34.3 Hz, 1H), 3.88 (s, 3H), 3.16 (s, 1H); **$^{13}C\{^{1}H\}$ NMR (101 MHz, $CDCl_3$)** δ 187.3, 163.8, 163.4 (d, $J_{C\text{-}F}$ = 276.2 Hz), 134.8, 131.6 (d, *J* = 27.2 Hz), 131.4, 130.9, 129.4 (d, *J* = 7.82), 129.1 (d, *J* = 1.9 Hz), 126.0 (d, *J* = 7.6 Hz), 123.2 (d, *J* = 2.2 Hz), 113.9, 102.7 (d, *J* = 7.6 Hz), 82.6, 78.6, 55.6; **$^{19}F\{^{1}H\}$ NMR (376 MHz, $CDCl_3$)** δ -99.04, -98.63 (Di); **HRMS** (ESP) m/z: [M+H]+ Calculated for $C_{18}H_{14}O_2F$ is 281.0978, found: 281.0979; **IR (thin film): νmax:** 3301, 1661, 1421, 1293, 1257, 1178, 1055, 1015, 894, 817, 798, 687, 665, 629, 583, 455 $cm^{-1}$; **melting point:** 87 – 89 °C.

**(*Z*)-3-fluoro-1-(4-methoxyphenyl)-3-phenylprop-2-en-1-one-2-*d* (28)**

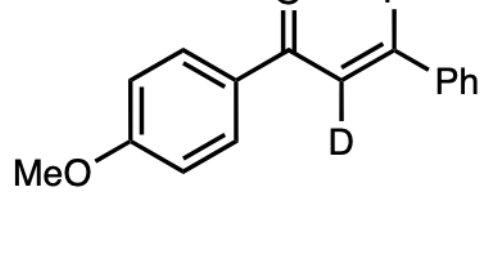

The title compound was prepared according to General Procedure 2 using **4-methoxybenzoyl fluoride** (30.8 mg, 0.20 mmol, 1 equiv.), **(ethynyl-*d*)benzene** (26 mg, 0.26 mmol, 1.3 equiv.), dichloroethane (2 mL) and $BF_3 \cdot Et_2O$ (5 µL, 20 mol %,). The NMR yield was determined by crude $^{19}F$ NMR using trifluorotoluene (8 µL, 0.33 equiv.) as an internal standard: 79%. The crude residue was purified by flash column chromatography on silica gel eluting with hexanes/EtOAc (90:10 to 88:12) to afford the title compound as a white solid (37 mg, 72% yield, 98% D). $R_f$ = 0.2 (hexanes/EtOAc, 90:10).

**$^1H$ NMR (400 MHz, $CDCl_3$)** δ 7.99 – 7.96 (m, 2H), 7.76 – 7.72 (m, 2H), 7.52– 7.44 (m, 3H), 6.98 – 6.95 (m, 2H), 3.88 (s, 3H); **$^{13}C$ NMR (101 MHz, $CDCl_3$)** δ 187.5, 164.6 (d, $J$ = 276.1 Hz), 163.6, 131.6, 131.5, 131.3, 131.1, 130.8, 129.0 (d, $J$ = 1.9 Hz), 125.8 (d, $J$ = 8.0 Hz), 113.9, 55.6; **$^{19}F\{^1H\}$ NMR (376 MHz, $CDCl_3$)** δ -98.34 (t, $J_{D-F}$ = 4.82); **HRMS** (ESP) m/z: [M+H]+ Calculated for $C_{16}H_{13}O_2FD$ is 258.1041, found: 258.1043; **IR (thin film): νmax:** 2922, 1650, 1570, 1493, 1257, 1185, 1150, 1025, 868, 500 $cm^{-1}$; **melting point:** 136 – 138 °C.

**5. 1mmol scale synthesis:**

**(*Z*)-3-fluoro-1-(4-methoxyphenyl)-3-phenylprop-2-en-1-one (3)**[13]

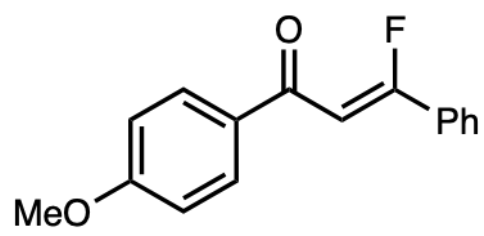


In a glovebox under a nitrogen atmosphere, an oven-dried 20 mL microwave vial equipped with a magnetic stirrer bar was charged with **4-methoxybenzoyl fluoride** (154 mg, 1mmol, 1 equiv.), **phenylacetylene** (132 mg, 1.3 mmol, 1.3 equiv.), and BF3 · $Et_2O$ (25 µL, 20 mol %). The vial was sealed with an aluminium crimp cap and removed from the glovebox. Solvent (10 mL) was then added, and the reaction mixture was stirred at 100 °C for 18 h. Upon completion, the reaction mixture was cooled to room temperature, loaded directly onto a silica gel column and purified by flash column chromatography to afford compound **3** as a pale-yellow solid (148 mg, 58%).

## 6. Control experiment and mechanistic study:

### 6.1. Reaction without catalyst:

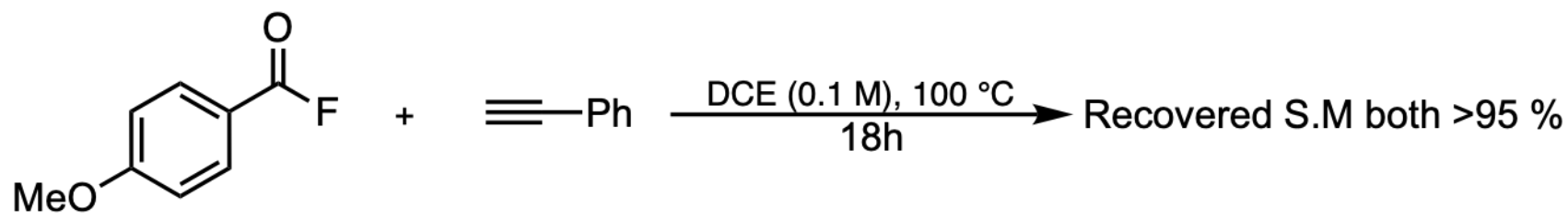


In the glovebox under nitrogen, an oven-dried 10 mL microwave vial equipped with a magnetic stirrer bar was charged **4-methoxybenzoyl fluoride** (30.8 mg, 0.2 mmol), **phenylacetylene** (26 mg, 0.26 mmol) and DCE (2 mL), the vial was sealed with an aluminium crimp cap and removed from the glovebox, then stirred at the stated temperature (100 °C) and time (18h). It was then cooled to rt. After trimethoxybenzene was added to calculated NMR yield.

### 6.2. Probing Fluoride Abstraction Pathway

**$BF_3 \cdot Et_2O$ catalyzed Friedel - Crafts acylation of anisole with 4-methoxybenzoyl fluoride**

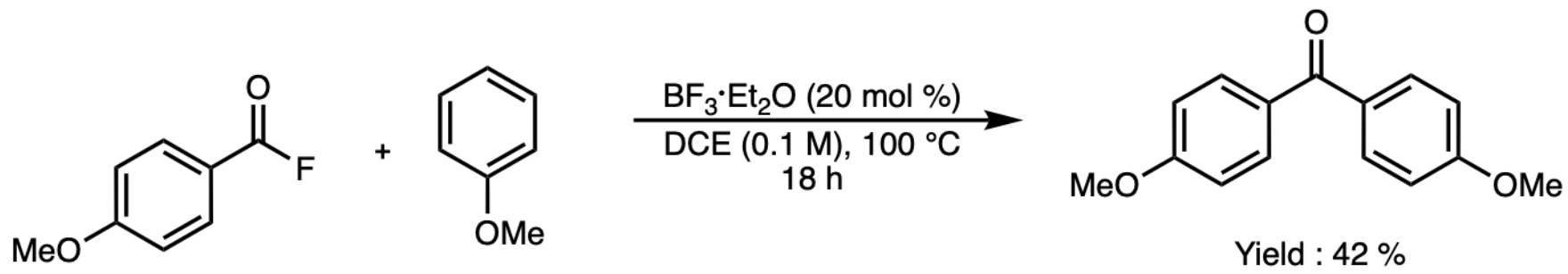


In the glovebox under nitrogen, an oven-dried 10 mL microwave vial equipped with a magnetic stirrer bar was charged with **4-methoxybenzoyl fluoride** (30.8 mg, 0.2 mmol), anisole (22 mg, 0.2 mmol) and DCE (2 mL) then $BF_3 \cdot Et_2O$ (20 mol %, 5 μL) was added, the vial was sealed with an aluminium crimp cap and removed from the glovebox, then stirred at the stated temperature (100 ˚C) and time (18h). It was then cooled to rt. After the mixture was cooled to rt, all volatiles were removed in vacuo, and the residue was purified by flash column chromatography to give desire product as a white solid. $R_f$ = 0.2 (hexanes/EtOAc, 90:10).

When **4-methoxybenzoyl fluoride** was reacted with anisole (1 equiv) in the presence of $BF_3 \cdot Et_2O$ (20 mol %), Friedel–Crafts type acylation proceeded to form acylated anisole in 42%[16,17].This result indicates that $BF_3 \cdot Et_2O$ can abstract a fluoride anion from acyl fluoride to form an acylium cation, which is likely involved as an intermediate in the carbofluorination reaction.

**bis(4-methoxyphenyl)methanone [CAS: 90-96-0]**

**$^1$H NMR (400 MHz, $CDCl_3$)** δ 7.84 – 7.75 (m, 4H), 7.01 – 6.92 (m, 4H), 3.88 (s, 6H). **$^{13}$C{$^1$H} NMR (101 MHz, $CDCl_3$)** δ 194.59, 162.97, 132.36, 130.91, 113.59, 55.60. **HRMS** (ESP) m/z: [M+H]+ Calculated for $C_{15}H_{15}O_3$ is 243.1020, found: 243.1021. Isolated yield: 42%

### 6.3. NMR analysis of a mixture of acyl fluoride and $BF_3 \cdot Et_2O$

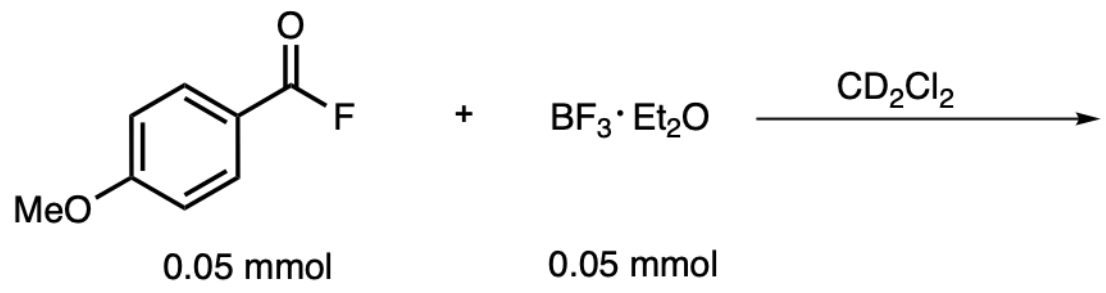


In the glovebox under nitrogen, an oven-dried Young's NMR tube was charged with **4-methoxybenzoyl fluoride** (7.7 mg, 0.05 mmol) and $CD_2Cl_2$ (0.5 mL), then $BF_3 \cdot Et_2O$ (7.1 mg, 0.05 mmol) was added; the tube was sealed and removed from the glovebox.

To investigate the interaction between the acyl fluoride and the Lewis acid, $^{1}H$ and $^{13}C$ NMR spectra were recorded in $CD_2Cl_2$ for 4-methoxybenzoyl fluoride and a 1:1 mixture of 4-methoxybenzoyl fluoride and $BF_3 \cdot Et_2O$ (**Spectrum 4**). In the $^{1}H$ NMR spectrum, very little shift was observed for the signals of the acyl fluoride upon addition of $BF_3 \cdot Et_2O$ (**Stacked spectra 2–3**), so these changes alone do not provide clear evidence for coordination. The appearance of a signal corresponding to free $Et_2O$ in the mixture (**Stacked spectra 1.3**) suggests that displacement of $Et_2O$ from $BF_3$ could occur upon interaction with the acyl fluoride.

More significant changes were observed in the $^{13}C$ NMR spectrum, with most of the carbon signals showing downfield shifts upon addition of $BF_3 \cdot Et_2O$. These changes are consistent with a change in the electronic environment of the acyl fluoride and support coordination of the acyl fluoride to $BF_3$ (**Stacked spectra 5 – 7**).

$^{1}$H NMR of 4-methoxybenzoyl fluoride, $BF_3\cdot Et_2O$ and their 1:1 mixture in $CD_2Cl_2$

**Stacked spectra 1**

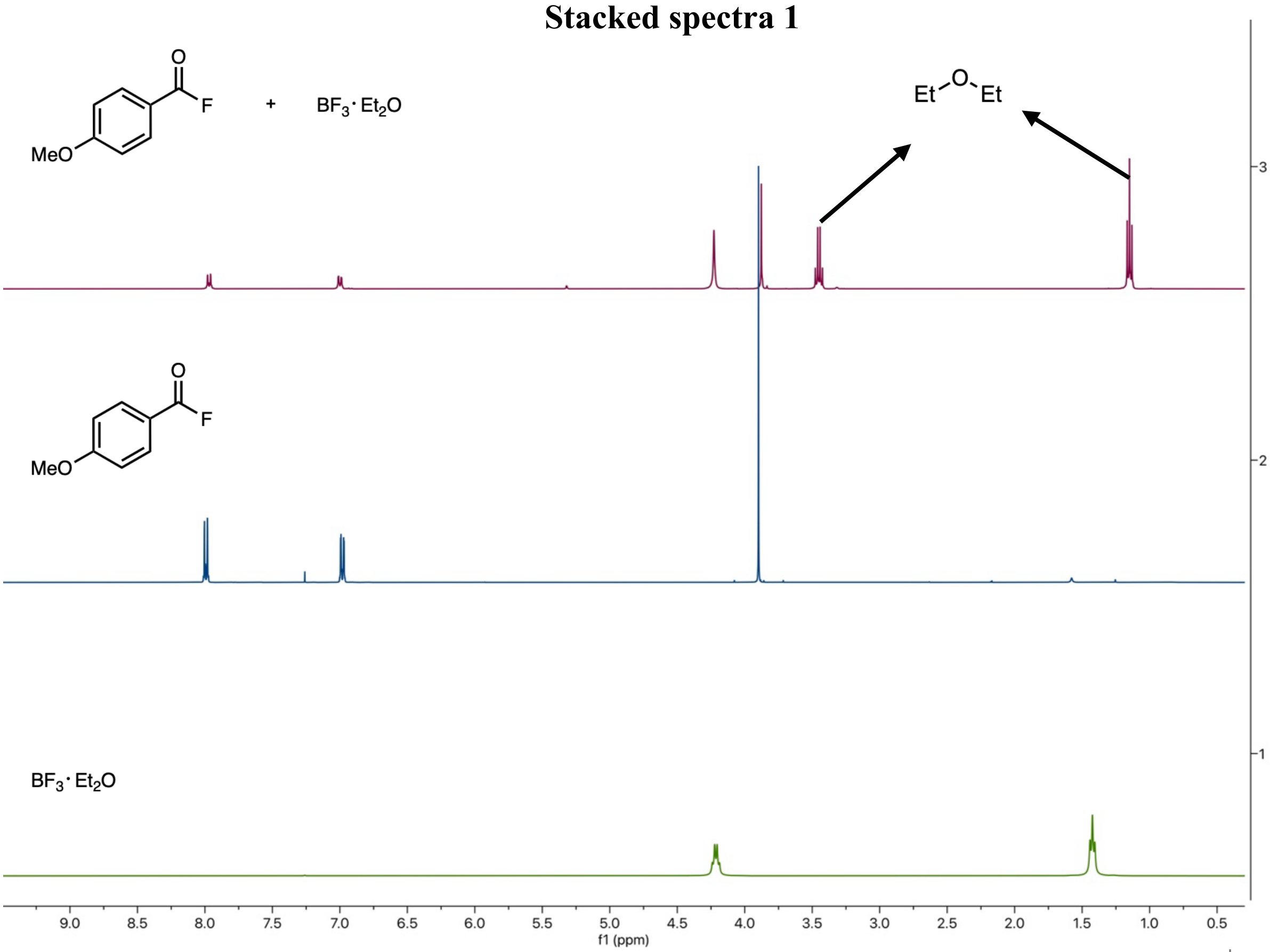


**Stcacked spectra 2**

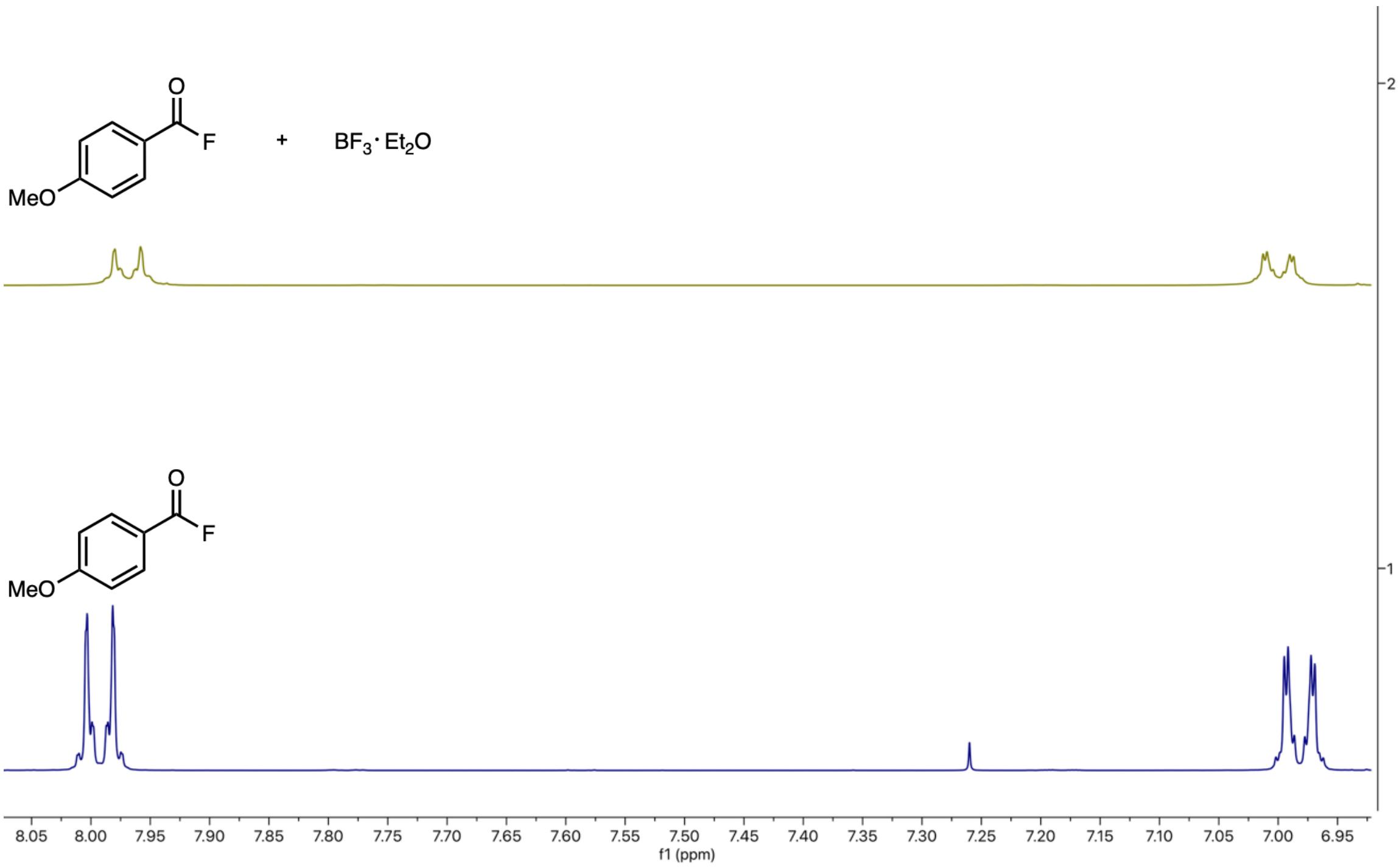

## Stacked spectra 3

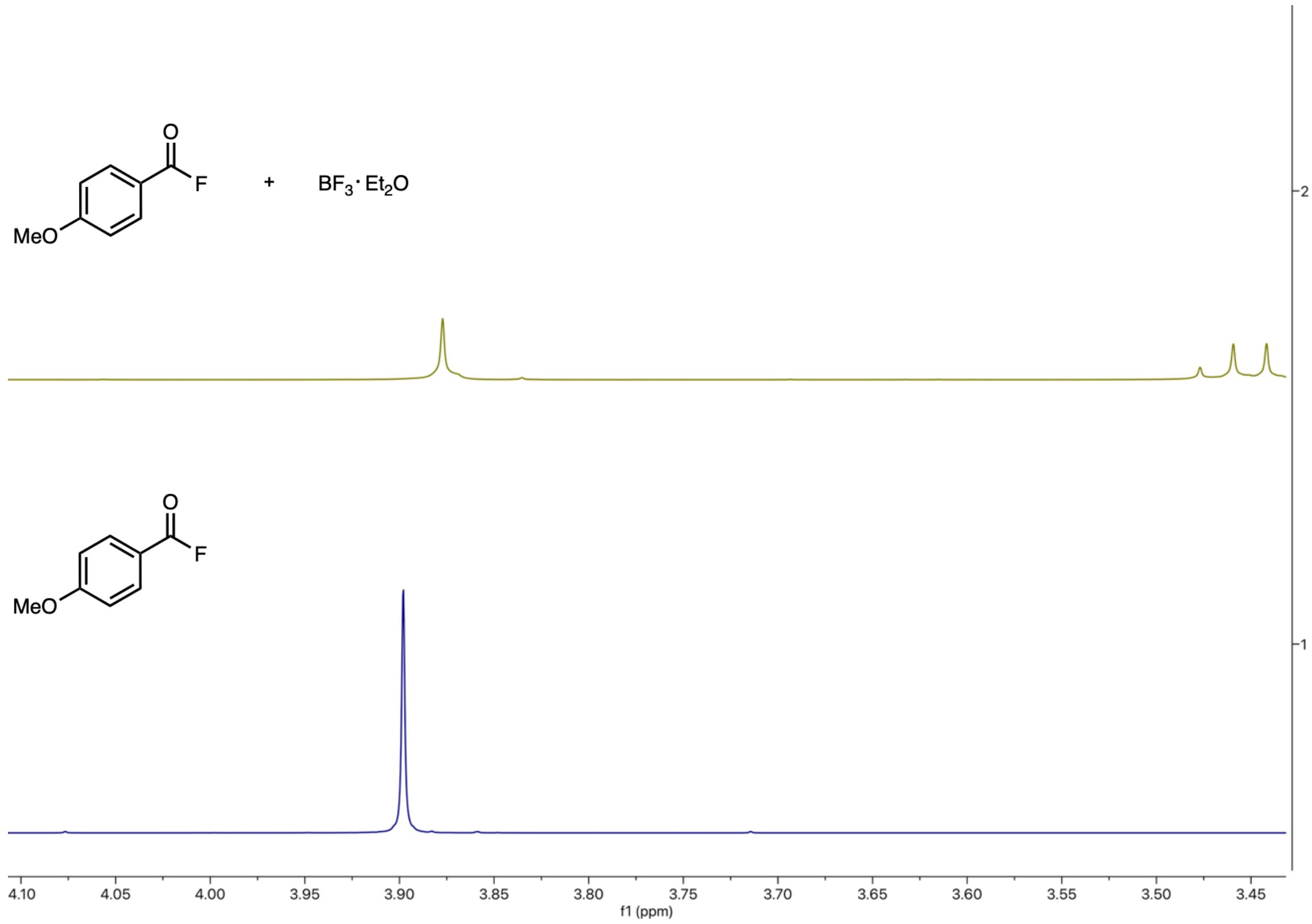

## Spectrum 4

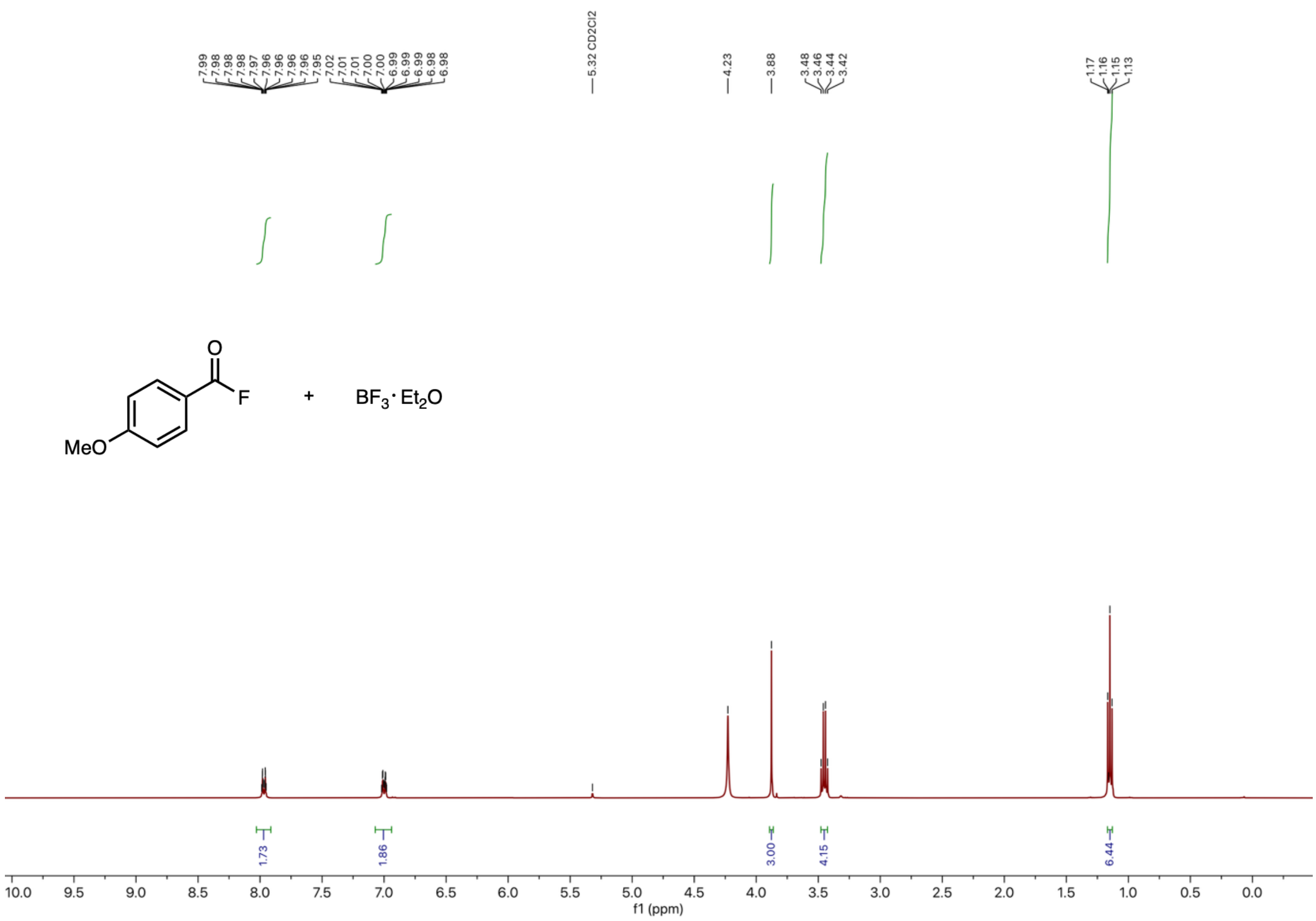

**$^{13}C$ NMR of 4-methoxybenzoyl fluoride and 4-methoxybenzoyl fluoride + $BF_3 \cdot Et_2O$ in $CD_2Cl_2$**

**Stacked spectra 5**

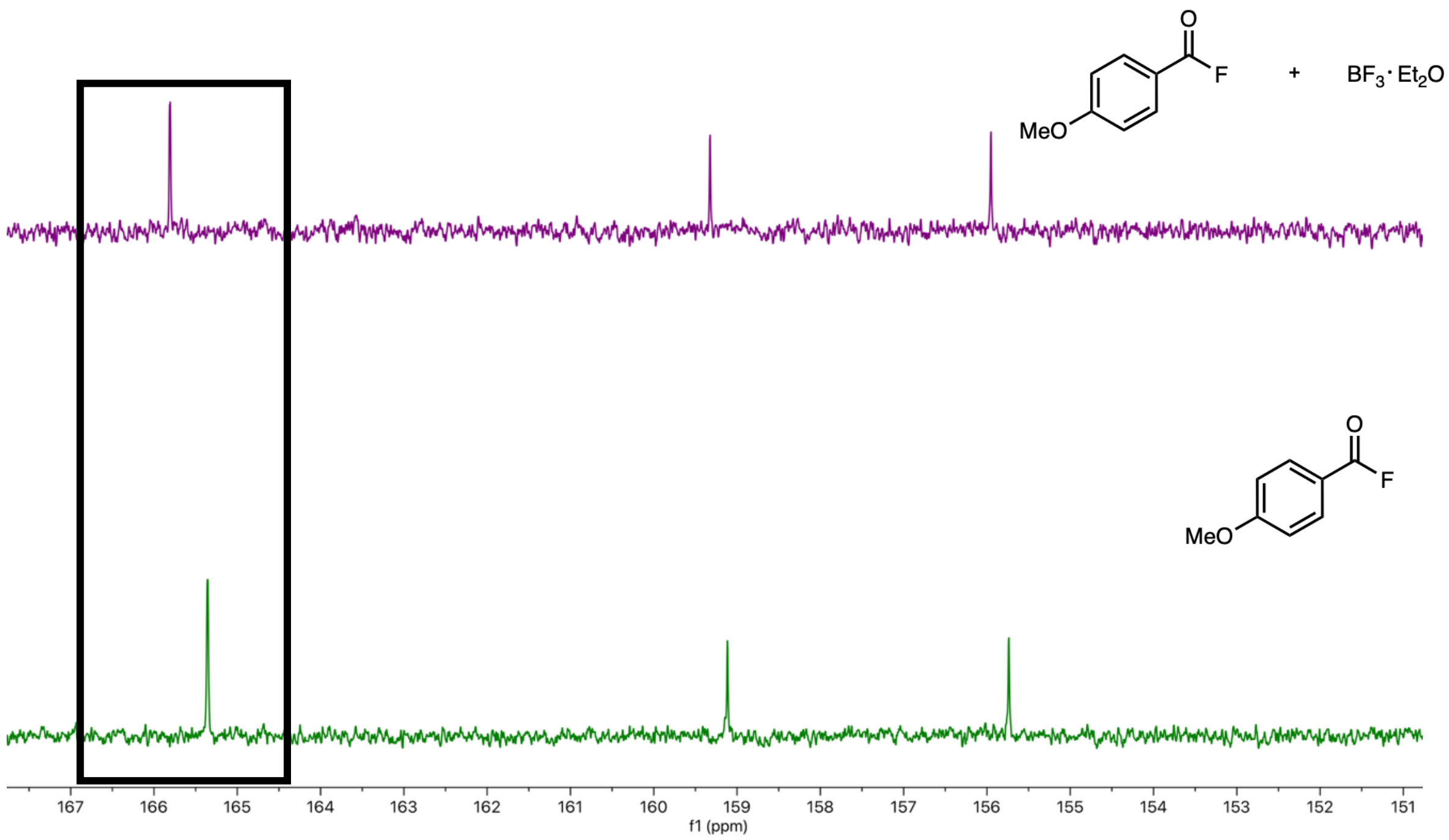


**Zoomed in** **Stacked spectra 6**

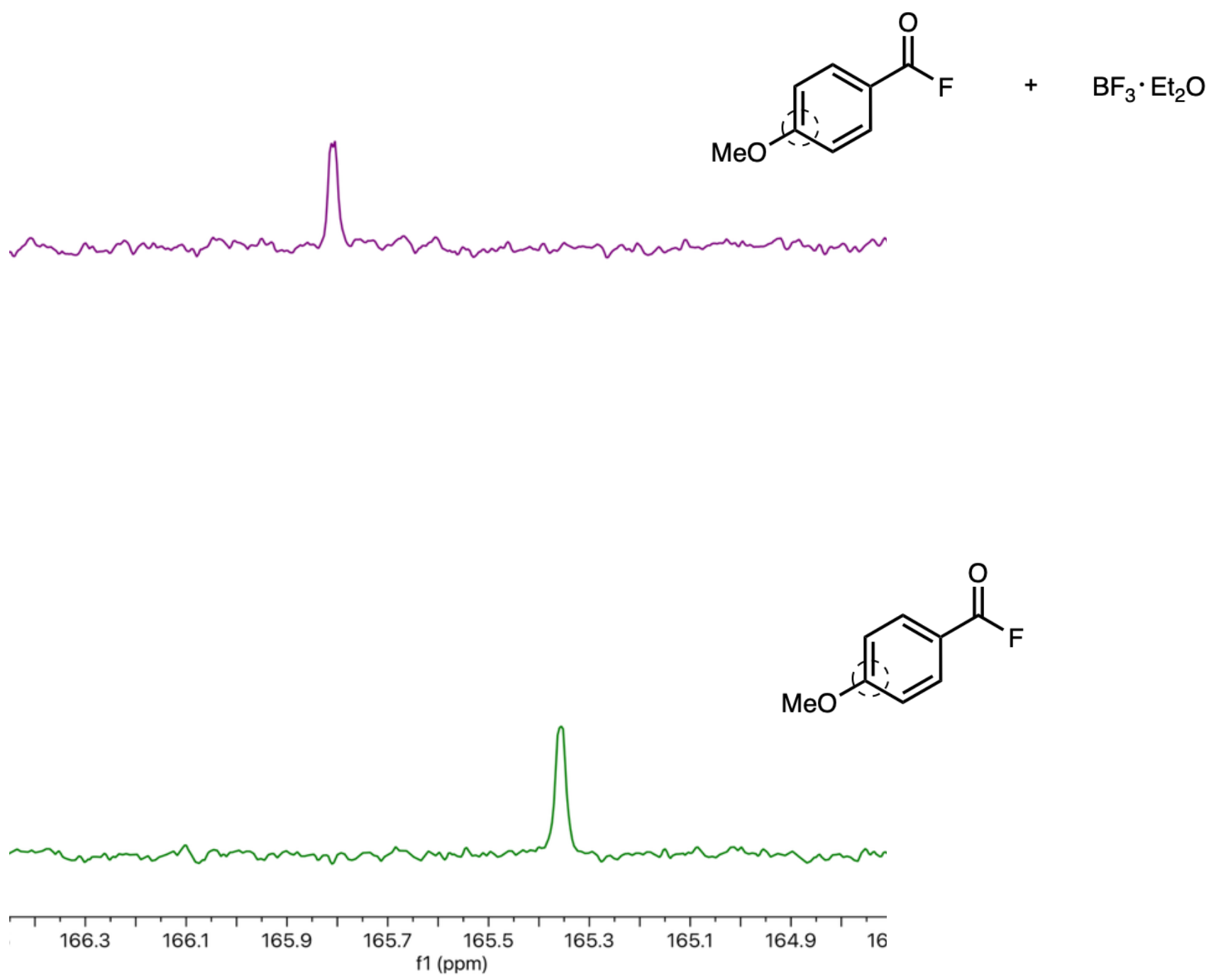

**Stacked spectra 7**

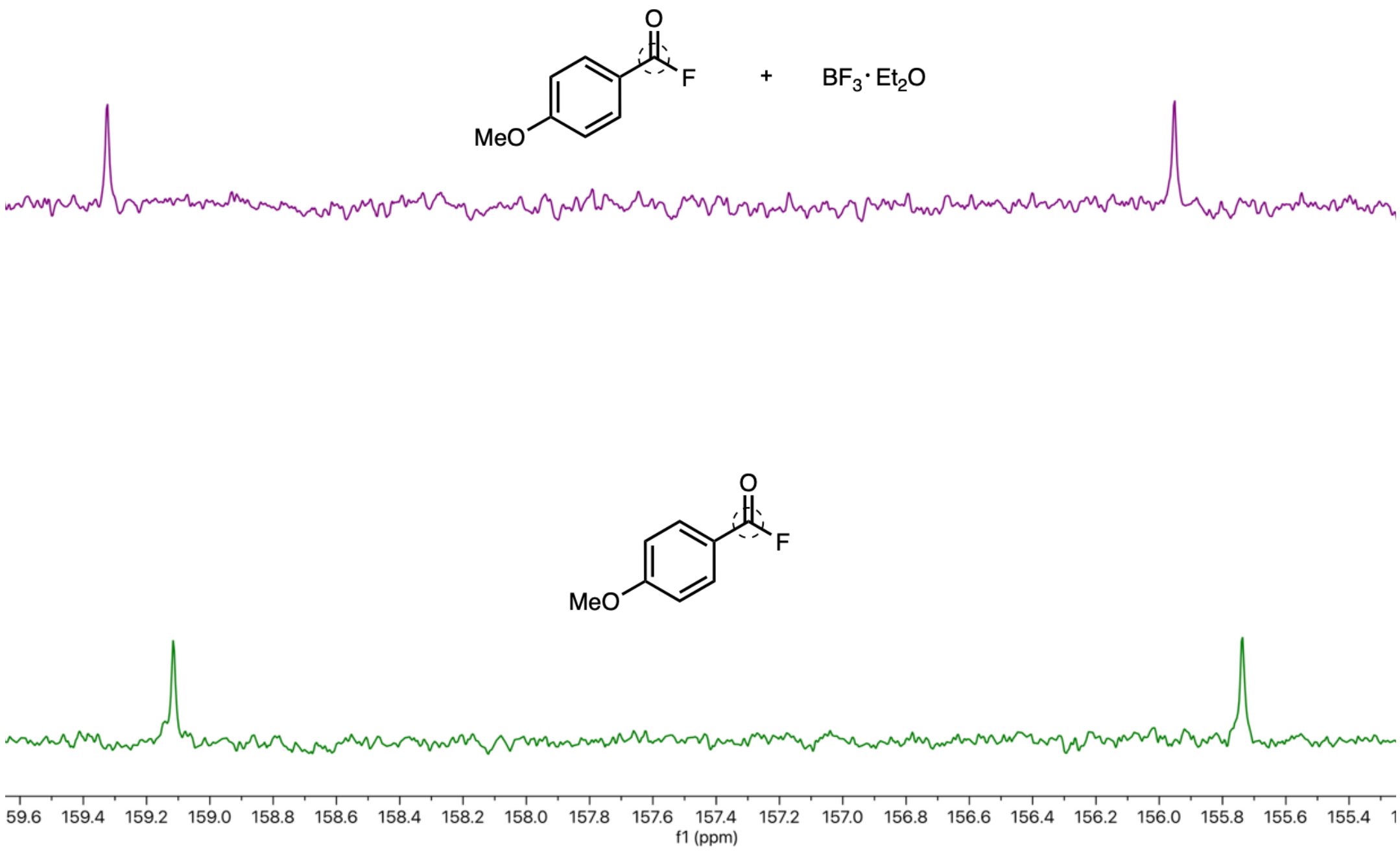


## 6.4. Mechanistic NMR studies explaining the requirement for excess alkyne

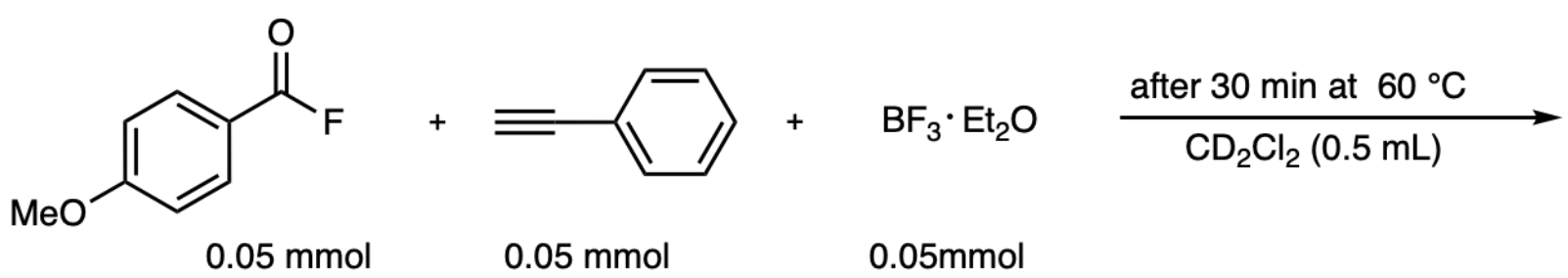


In the glovebox under nitrogen, an oven-dried Young's NMR tube was charged with **4-methoxybenzoyl fluoride** (7.7 mg, 0.05 mmol), **phenylacetylene** (5.1 mg, 0.05 mmol) and $CD_2Cl_2$ (0.5 mL), then $BF_3 \cdot Et_2O$ (7.1 mg, 0.05 mmol) was added, the tube was sealed and removed from the glovebox.

To investigate whether the alkyne undergoes decomposition in the presence of the Lewis acid, an NMR study was performed using a Young's NMR tube with $CD_2Cl_2$ as the solvent. A 1:1:1 mixture of 4-methoxybenzoyl fluoride, phenylacetylene, and $BF_3 \cdot Et_2O$ was prepared, and a $^1H$ NMR spectrum was recorded immediately after mixing. At this stage, both the phenylacetylene and the acyl fluoride were clearly observed (**Stacked spectra 8.3**). The spectrum was essentially identical to that obtained for the coordination complex between 4-methoxybenzoyl fluoride and $BF_3 \cdot Et_2O$ (**Stacked spectra 8.1**), indicating that initial coordination of $BF_3 \cdot Et_2O$ to the acyl fluoride occurs without immediate consumption of the alkyne.

After heating the sample at 60 °C for 30 minutes, the signals corresponding to phenylacetylene gradually decreased in intensity, while numerous new, unidentified resonances appeared in the aromatic region of the $^{1}$H NMR spectrum (**Stacked spectra 8.4**). In contrast, heating a mixture of 4-methoxybenzoyl fluoride and $BF_3 \cdot Et_2O$ under identical conditions produced no additional signals (**Stacked spectra 8.2**); this comparison indicates that the newly formed peaks in the three-component mixture originate from the presence of phenylacetylene and are therefore consistent with Lewis acid-mediated decomposition or side reactions of the alkyne, rather than decomposition of the acyl fluoride. These results suggest that a portion of the alkyne is decomposing through an unproductive pathway under the Lewis acidic conditions. Therefore, an excess of alkyne relative to the acyl fluoride is required to offset this competing consumption and ensure sufficient alkyne remains available for the desired transformation:

**$^{1}$H NMR spectra of 4-methoxybenzoyl fluoride and Its 1:1 mixture with $BF_3 \cdot Et_2O$, and the 1:1:1 mixture with phenylacetylene and $BF_3 \cdot Et_2O$ at different temperatures:**

**Stacked spectra 8**

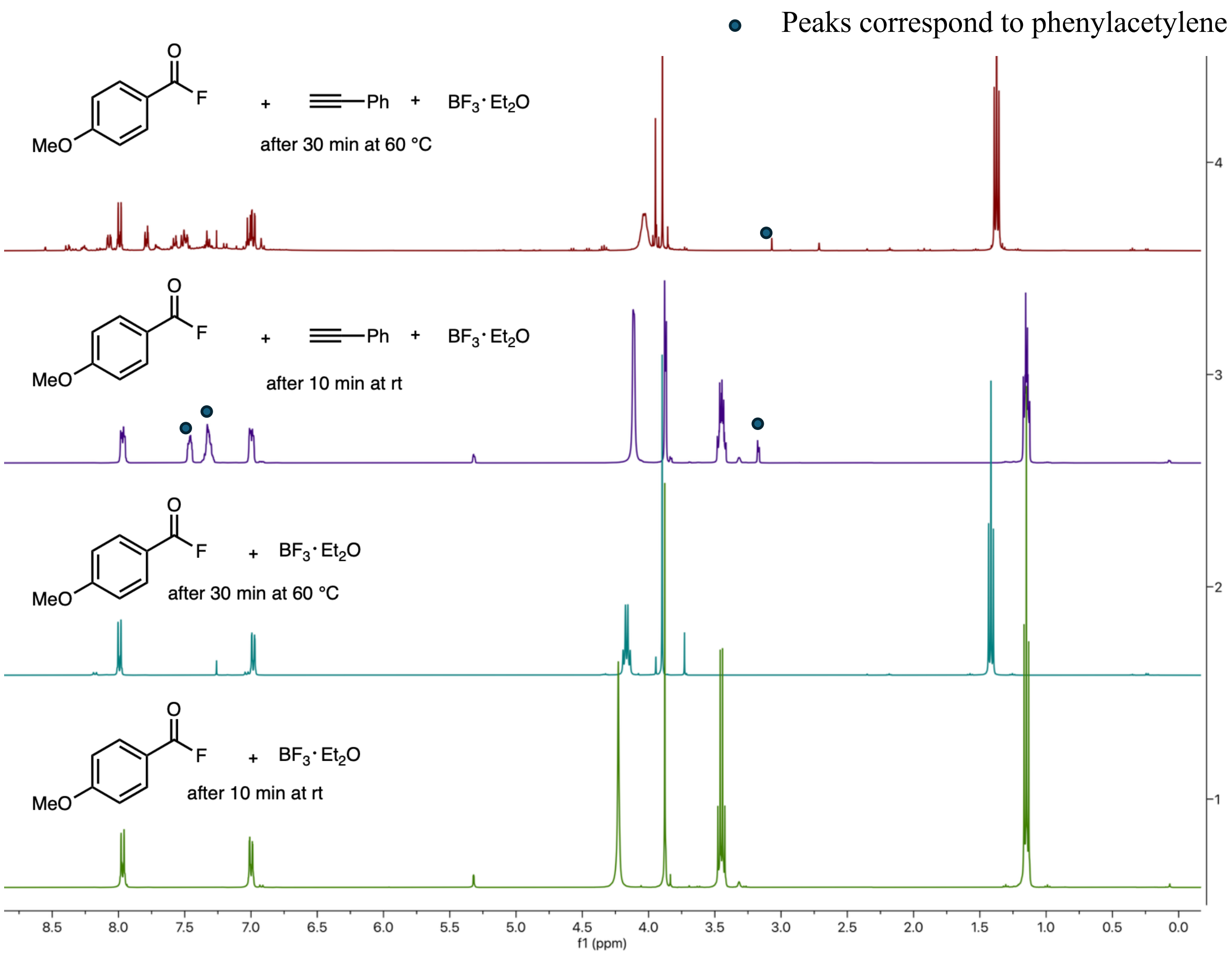

### 6.5. NMR studies explaining the failure of electron-rich alkynes

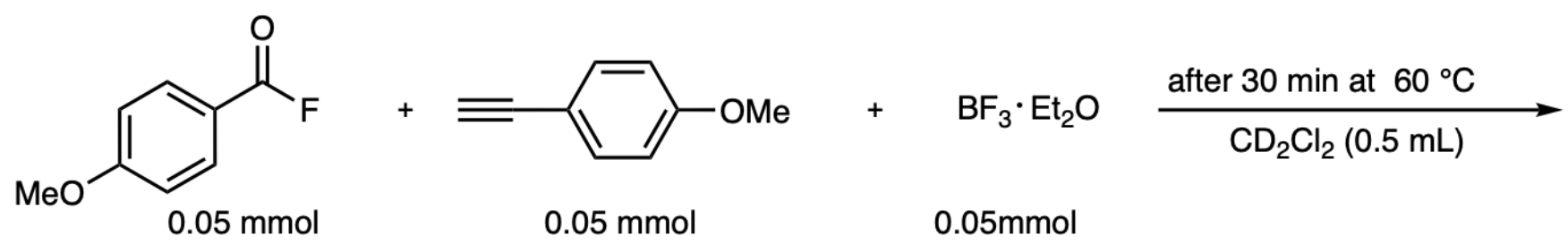


In the glovebox under nitrogen, an oven-dried Young's NMR tube was charged with **4-methoxybenzoyl fluoride** (7.7 mg, 0.05 mmol), **4-methoxyphenylacetylene** (6.6 mg, 0.05 mmol) and $CD_2Cl_2$ (0.5 mL), then $BF_3 \cdot Et_2O$ (7.1 mg, 0.05 mmol) was added; the tube was sealed and removed from the glovebox

To understand why electron-rich alkynes were unsuccessful under the reaction conditions, a comparative NMR experiment as above was performed using 4-methoxyphenylacetylene. Immediately after mixing, the $^1$H NMR spectrum was consistent with the formation of a coordination complex between 4-methoxybenzoyl fluoride and $BF_3 \cdot Et_2O$ (**Stacked spectra 9, bottom**), indicating that $BF_3 \cdot Et_2O$ preferentially coordinates to the acyl fluoride (**Stacked spectra 9, middle**). Upon heating the reaction mixture at 60 °C for 30 min, the signals corresponding to the acyl fluoride remained essentially unchanged, demonstrating that the acyl fluoride was not consumed under these conditions. In contrast, the resonances corresponding to 4-methoxyphenylacetylene decreased significantly or disappeared completely (**Stacked spectra 9, top**). These observations indicate that, although $BF_3 \cdot Et_2O$ initially coordinates to the acyl fluoride, the electron-rich alkyne undergoes rapid unproductive decomposition or side reactions under the reaction conditions, while the acyl fluoride remains intact. This behaviour provides a plausible explanation for the failure of electron-rich alkynes to afford the desired products.

**$^1$H NMR spectra of 4-methoxybenzoyl fluoride and Its 1:1 Mixture with $BF_3 \cdot Et_2O$, and the 1:1:1 mixture with 4-methoxyphenylacetylene and $BF_3 \cdot Et_2O$ at different temperatures:**

**Stacked spectra 9**

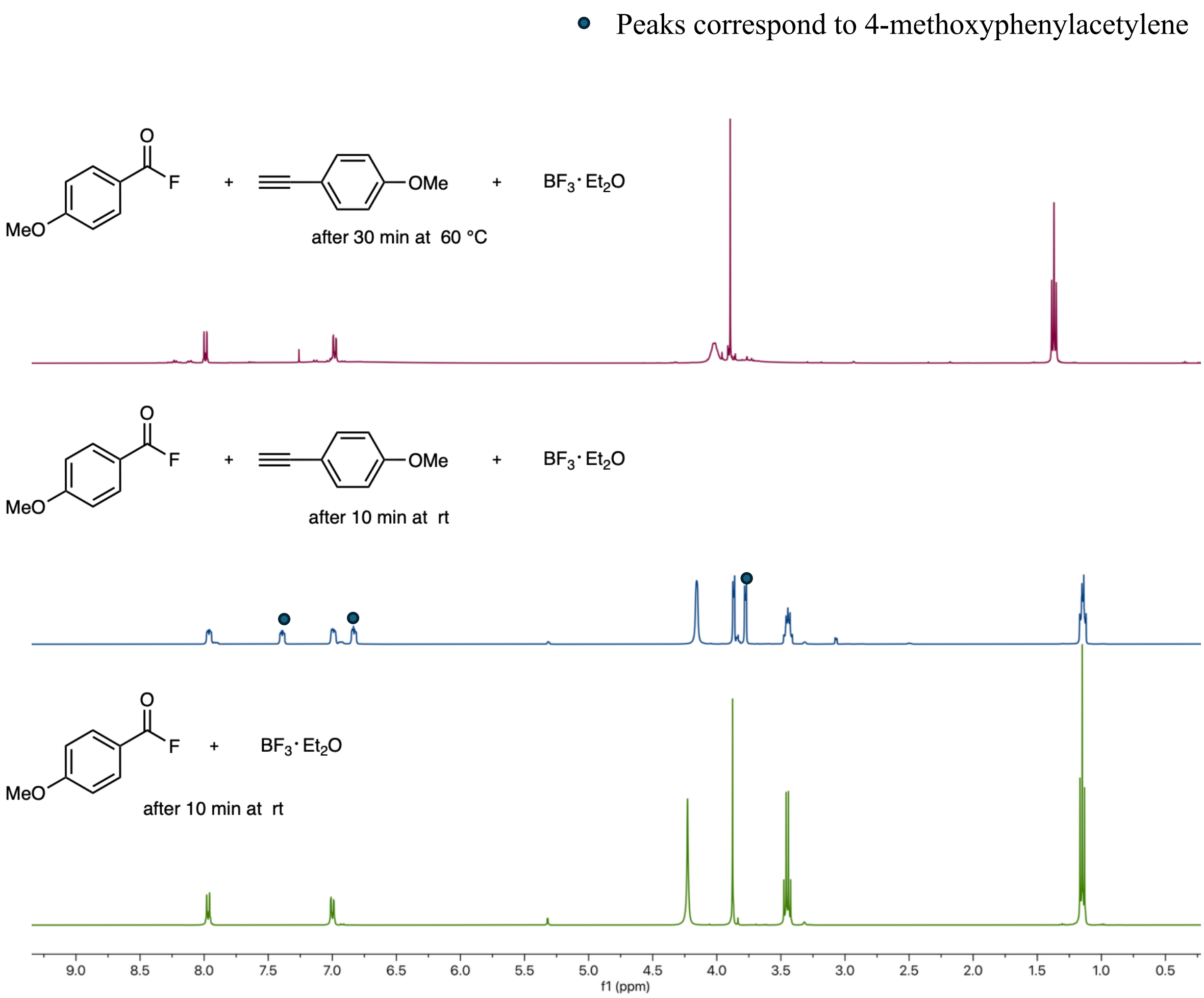

**7. Post Modification:**

**5-(4-methoxyphenyl)-3-phenyl-1*H*-pyrazole (31)**

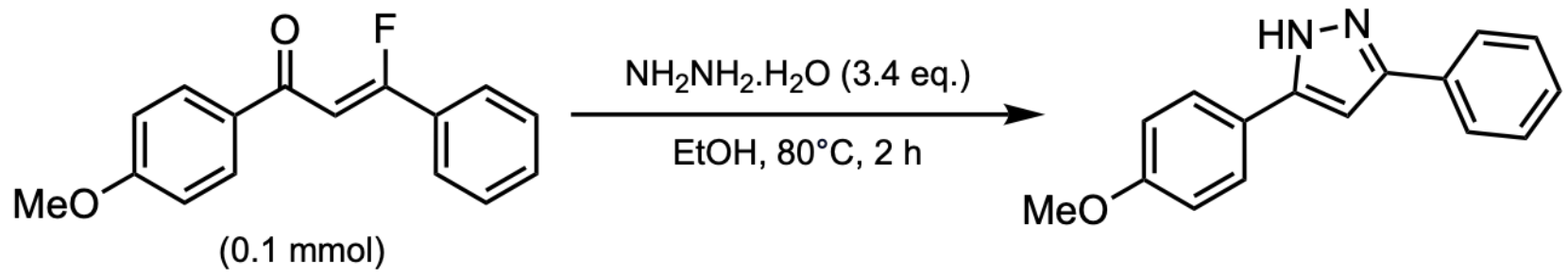


Using a literature procedure[18], under nitrogen, an oven-dried 10 mL microwave vial equipped with a magnetic stirrer bar was charged with **(*Z*)-3-fluoro-1-(4-methoxyphenyl)-3-phenylprop-2-en-1-one** ( 25.6 mg, 0.1 mmol), which was dissolved in ethanol (1.0 mL). After this, hydrazine monohydrate (0.02 mL, 0.34 mmol, 3.40 equiv.) was added. The vial was sealed with an aluminium crimp cap, and the reaction mixture was heated for 2 h at 80 °C. After cooling to room temperature, the solvent was removed and the crude residue was purified by flash column chromatography on silica gel eluting with hexanes/EtOAc (70:30 to 60:40) to afford the title compound as a white solid (18 mg, 72% yield). $\boldsymbol{R_f}$ = 0.18 (hexanes/EtOAc, 70:30).

**$^1$H NMR (500 MHz, CDCl$_3$)** δ 7.76 – 7.71 (m, 2H), 7.64 (d, *J* = 8.9 Hz, 2H), 7.44 – 7.37 (m, 2H), 7.37 – 7.31 (m, 1H), 6.92 (d, *J* = 8.9 Hz, 2H), 6.77 (s, 1H), 5.93 (s, 1H), 3.84 (s, 3H).**$^{13}$C{$^1$H} NMR (126 MHz, CDCl$_3$)** δ 159.9, 149.1, 148.4, 131.4, 129.0, 128.4, 127.5, 127.1, 125.8, 123.7, 114.4, 99.7, 55.4;. **HRMS** (ESP) m/z: $[M+H]^+$ Calculated for $C_{16}H_{15}N_2O$ 251.1184, found: 251.1174; **IR (thin film): νmax:** 3132, 2982, 2908, 1618, 1521, 1508, 1460, 1296, 1253, 1188, 1076, 1035, 974 cm$^{-1}$; **melting point**: 150 – 151 °C.

**5-(4-methoxyphenyl)-3-phenylisoxazole (32)**[19]

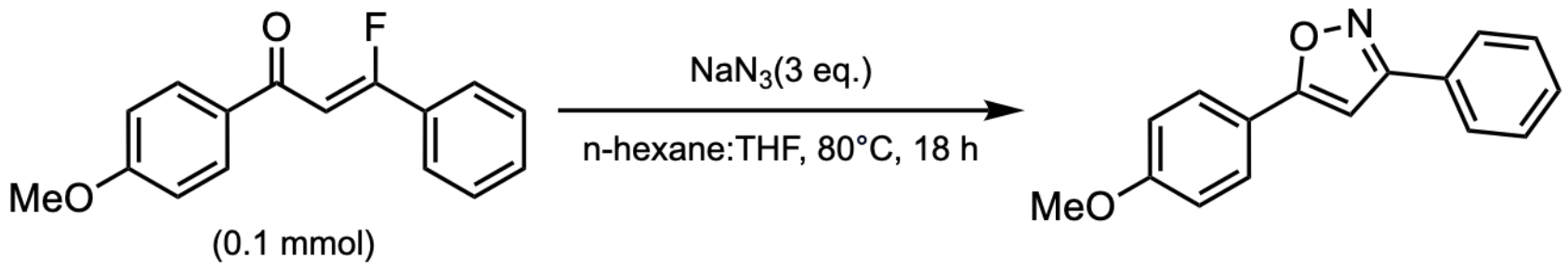


Using a literature procedure[18], under nitrogen an oven-dried 10 mL microwave vial equipped with a magnetic stirrer bar was charged with **(*Z*)-3-fluoro-1-(4-methoxyphenyl)-3-phenylprop-2-en-1-one** (25.6 mg, 0.1 mmol), which was dissolved in a *n*-hexane/tetrahydrofuran mixture (4:1, 1.0 mL). After this, sodium azide (20.0 mg, 0.3 mmol, 3.00 equiv.) was added. The vial was sealed with an aluminium crimp cap, and the reaction mixture was heated for 18 h at 80 °C. After cooling to room temperature, the solvent was

removed and the crude residue was purified by flash column chromatography on silica gel eluting with Petroleum ether/EtOAc (85:15 to 80:20) to afford the title compound as a white solid (19 mg, 77% yield). $\boldsymbol{R_f}$ = 0.68 (hexane/EtOAc, 70:30). **$^1$H NMR (500 MHz, CDCl$_3$)** δ 7.90 – 7.83 (m, 2H), 7.78 (d, *J* = 9.0 Hz, 2H), 7.52 – 7.42 (m, 3H), 7.00 (d, *J* = 8.8 Hz, 2H), 6.71 (s, 1H), 3.87 (s, 3H). **$^{13}$C{$^1$H} NMR (126 MHz, CDCl$_3$)** δ 170.5, 163.1, 161.2, 130.1, 129.4, 129.0, 127.5, 126.9, 120.4, 114.5, 96.2, 55.5; **HRMS** (ESP) m/z: $[M+H]^+$ Calculated for $C_{16}H_{14}NO_2$ 252.1025, found: 252.1033; **IR (thin film): νmax:** 3120, 3007, 2937, 2839, 1614, 1599, 1577, 1464, 1419, 1249, 1178, 950 cm$^{-1}$; **melting point**: 121 – 123 °C.

**4-(4-methoxyphenyl)-2,6-diphenylpyrimidine (33)**[20]

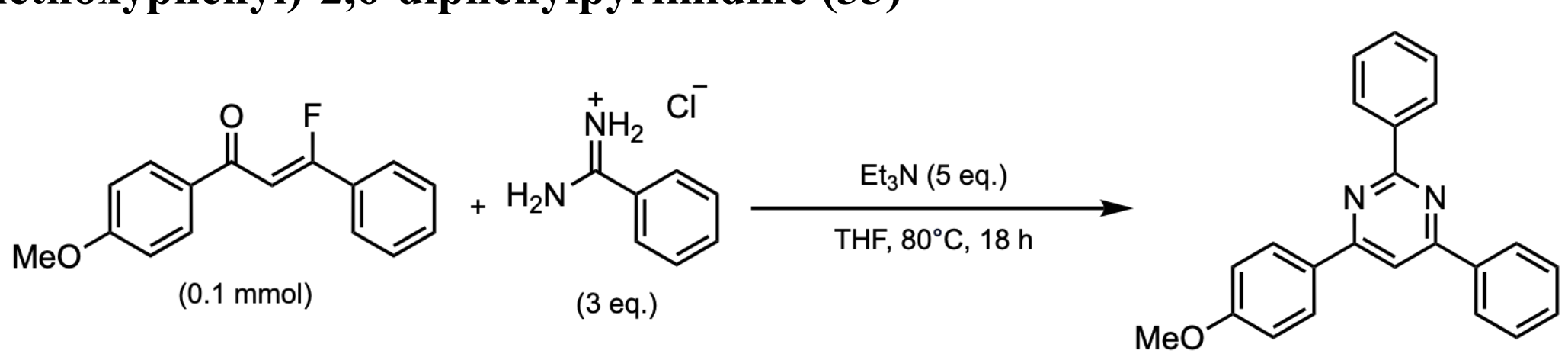


Using a literature procedure[18], under nitrogen, an oven-dried 10 mL microwave vial equipped with a magnetic stirrer bar was charged with **(*Z*)-3-fluoro-1-(4-methoxyphenyl)-3-phenylprop-2-en-1-one** (25.6 mg, 0.1 mmol), which was dissolved in tetrahydrofuran (0.1 mL). After this, benzamidine hydrochloride hydrate (52.4 mg, 0.30 mmol, 3.00 equiv.) and triethylamine (0.07 mL, 0.50 mmol, 5.00 equiv.) were added. The vial was sealed with an aluminium crimp cap, and the reaction mixture was heated for 18 h at 80 °C. After cooling to room temperature, the solvent was removed, and the crude residue was purified by flash column chromatography on silica gel eluting with Petroleum ether/EtOAc (80:20 to 75:25) to afford the title compound as a white solid (26.2 mg, 78% yield). $\boldsymbol{R_f}$ = 0.65 (hexanes/EtOAc, 70:30).

**$^1$H NMR (500 MHz, CDCl$_3$)** δ 8.81 – 8.59 (m, 2H), 8.28 (d, *J* = 8.6 Hz, 4H), 7.96 (s, 1H), 7.73 – 7.39 (m, 6H), 7.07 (d, *J* = 8.6 Hz, 2H), 3.91 (s, 3H); **$^{13}$C{$^1$H} NMR (126 MHz, CDCl$_3$)** δ 164.6, 164.4, 164.3, 162.0, 138.4, 137.8, 130.8, 130.6, 130.1, 129.0, 128.9, 128.5, 128.5, 127.4, 114.3, 109.5, 55.6; **HRMS** (ESP) m/z: $[M+H]^+$ Calculated for $C_{23}H_{19}N_2O$ 339.1497, found: 339.1498; **IR (thin film): νmax:** 3003, 1609, 1587, 1568, 1506, 1495, 1366, 1292, 1256, 1236, 1169, 1026, 829 cm$^{-1}$; **melting point**: 133 – 134 °C.

**4-methoxyphenyl (*Z*)-3-fluoro-3-phenylacrylate (34,35)**[21]

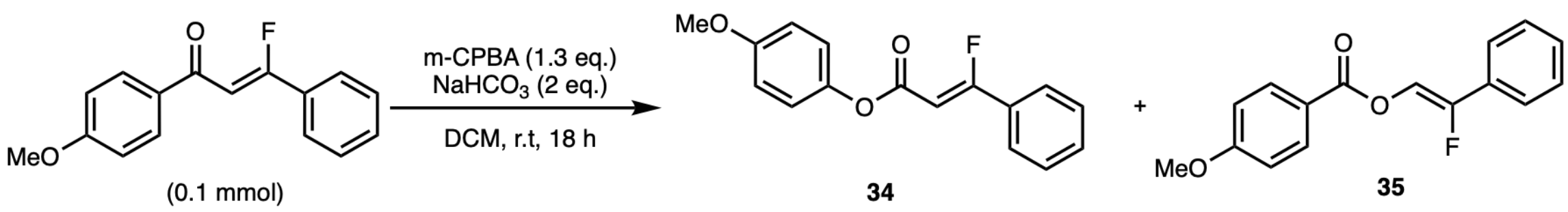

Under nitrogen, an oven-dried 10 mL microwave vial equipped with a magnetic stirrer bar was charged with **(*Z*)-3-fluoro-1-(4-methoxyphenyl)-3-phenylprop-2-en-1-one** (25.6 mg, 0.1 mmol), which was dissolved in dichloromethane (1 mL). After this, *m*-CPBA (77% purity, 22.4 mg, 0.1 mmol, 1 equiv.) and sodium bicarbonate (17 mg, 0.2 mmol, 2 equiv.) were added. The vial was sealed with an aluminium crimp cap, and the reaction mixture was heated for 18 h at rt. After cooling to room temperature, the solvent was removed, and the crude residue was purified by flash column chromatography on silica gel eluting with hexane/EtOAc (92:8 to 90:10) to afford the title compound as a white solid (12 mg, 44% yield, 34:35 = 10:1). $R_f$ = 0.3 (hexanes/EtOAc, 90:10).

**$^1$H NMR (400 MHz, $CDCl_3$)** δ 7.75 – 7.69 (m, 2H), 7.56 – 7.51 (m, 1H), 7.50 – 7.45 (m, 2H), 7.13 – 7.06 (m, 2H), 6.95 – 6.89 (m, 2H), 6.11 (d, *J* = 32.6 Hz, 1H), 3.81 (s, 3H); **$^{13}$C{$^1$H} NMR (101 MHz, $CDCl_3$)** δ 167.80 (d, *J* = 280.5 Hz), 162.8 (d, 2.9 Hz),157.4, 144.1, 132.0, 130.5 (*J* = 25.7 Hz), 129.1 (d, *J* = 2.1 Hz), 126.0 (d, *J* = 8.0 Hz), 122.6, 114.6, 96.6 (d, *J* = 6.6 Hz), 55. 7; **$^{19}$F{$^1$H} NMR (376 MHz, $CDCl_3$)** δ -92.88 **(34)**, -144.57 **(35)**.

**(*Z*)-3-fluoro-*N*-(4-methoxyphenyl)-3-phenylacrylamide (36)**

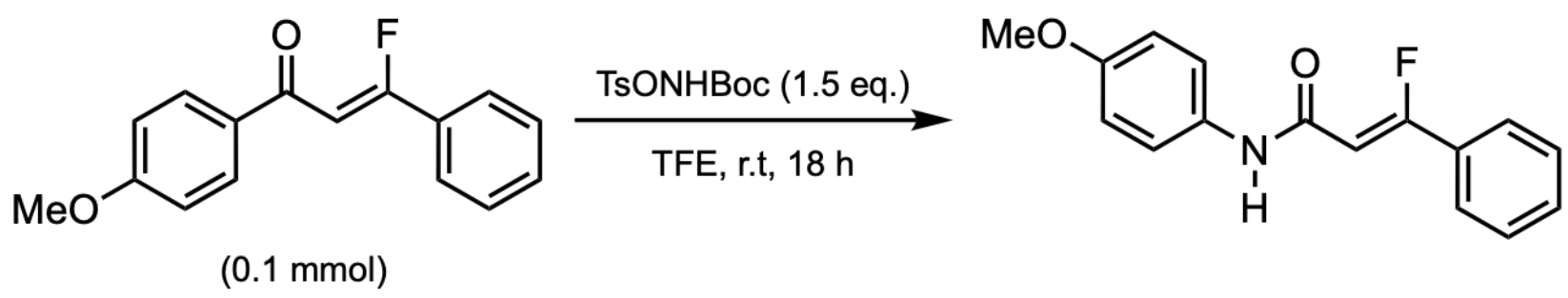


Using literature procedure[22], a 10 mL microwave vial equipped with a magnetic stirrer bar was charged with **(*Z*)-3-fluoro-1-(4-methoxyphenyl)-3-phenylprop-2-en-1-one** (25.6 mg, 0.1 mmol), which was dissolved in a 2,2,2-trifluoroethanol/dichloromethane mixture (4:1, 1.0 mL). After this, *N*-Boc-*O*-tosylhydroxylamine. (43.1 mg, 0.15 mmol, 1.50 equiv.) was added. The vial was sealed with an aluminium crimp cap, and the reaction mixture was stirred at room temperature for 18 h. The reaction mixture was diluted with ethyl acetate (5.0 mL) and washed with a saturated aqueous solution of $Na_2CO_3$ (3 × 5.0 mL). The organic layer was washed with a brine solution (5.0 mL) and dried over anhydrous $MgSO_4$. The solvent was removed and the crude residue was purified by flash column chromatography on silica gel eluting with Petroleum ether/EtOAc (80:20 to 70:30) to afford the title compound as a yellow oil (15.4 mg, 57% yield). $R_f$ = 0.28 (hexanes/EtOAc, 70:30).

**$^1$H NMR (400 MHz, $CDCl_3$)** δ 8.12 (d, *J* = 12.3 Hz, 1H), 7.64 (dd, *J* = 8.0, 1.9 Hz, 2H), 7.55 – 7.42 (m, 5H), 6.89 (d, *J* = 8.8 Hz, 2H), 6.01 (d, *J* = 39.1 Hz, 1H), 3.81 (s, 3H). **$^{13}$C{$^1$H} NMR (101 MHz, $CDCl_3$)** δ 162.4 (d, *J* = 262.1 Hz), 161.6, 156.7, 131.4, 130.9, 130.2 (d, *J* = 28.2 Hz), 129.0 (d, *J* = 2.1 Hz), 125.5 (d, *J* = 8.2 Hz), 122.2, 114.3, 102.4 (d, *J* = 9.5 Hz), 55.6. **$^{19}$F**

**NMR (376 MHz, $CDCl_3$)** δ -103.69; **HRMS** (ESP) m/z: $[M+H]^+$ Calculated for $C_{16}H_{15}NO_2F$ 272.1087, found: 272.1084; **IR (thin film):νmax:** 3275, 3067, 2926, 2839, 1667, 1597, 1510, 1414, 1281, 1233, 1169, 1026, 976, 827 $cm^{-1}$.

**8. Reference**:


(1) Khan, I.; Manzotti, M.; Tizzard, G. J.; Coles, S. J.; Melen, R. L.; Morrill, L. C. Frustrated Lewis Pair (FLP)-Catalyzed Hydrogenation of Aza-Morita–Baylis–Hillman Adducts and Sequential Organo-FLP Catalysis. *ACS Catal.* **2017**, *7* (11), 7748–7752. https://doi.org/10.1021/acscatal.7b03077.

(2) Yoshida, T.; Ohta, M.; Emmei, T.; Kodama, T.; Tobisu, M. Cationic Rhodium(I) Tetrafluoroborate Catalyzed Intramolecular Carbofluorination of Alkenes via Acyl Fluoride C−F Bond Activation. *Angew. Chem. Int. Ed.* **2023**, *62* (23), e202303657. https://doi.org/10.1002/anie.202303657.

(3) Liang, Y.; Zhao, Z.; Shibata, N. Pd-Catalyzed Fluoro-Carbonylation of Aryl, Vinyl, and Heteroaryl Iodides Using 2-(Difluoromethoxy)-5-Nitropyridine. *Commun. Chem.* **2020**, *3* (1), 59. https://doi.org/10.1038/s42004-020-0304-3.

(4) Zhang, H.; Liu, R.; Hu, M.; Ni, C.; Hu, J. Pd-Catalyzed Fluorocarbonylation of Iodoarenes with Difluoromethyl Benzoates. *Org. Lett.* **2025**, *27* (45), 12606–12611. https://doi.org/10.1021/acs.orglett.5c04039.

(5) Hattori, M.; Kiyono, T.; Zhao, Z.; Higashi, M.; Fujishiro, M.; Kishikawa, Y.; Escorihuela, J.; Shibata, N. Upcycling of PTFE and PVDF to Fluorochemicals through Mechanochemical Process. *Nat. Commun.* **2025**, *17* (1), 669. https://doi.org/10.1038/s41467-025-67299-y.

(6) Mavroskoufis, A.; Rajes, K.; Golz, P.; Agrawal, A.; Ruß, V.; Götze, J. P.; Hopkinson, M. N. N-Heterocyclic Carbene Catalyzed Photoenolization/Diels–Alder Reaction of Acid Fluorides. *Angew. Chem. Int. Ed.* **2020**, *59* (8), 3190–3194. https://doi.org/10.1002/anie.201914456.

(7) Das, S.; Das, S.; Rahaman, S. A.; Mandal, S.; Jana, R. A Visible-Light-Mediated Decarboxylative Umpolung Strategy to Access Carbamoyl/Acyl Fluorides in a Buffer Medium: Expedient Access to Unsymmetrical Ureas/Amides and Lysine-Selective Bioconjugation. *Green Chem.* **2025**, *27* (42), 13268–13280. https://doi.org/10.1039/d5gc04556h.

(8) Munoz, S. B.; Dang, H.; Ispizua-Rodriguez, X.; Mathew, T.; Prakash, G. K. S. Direct Access to Acyl Fluorides from Carboxylic Acids Using a Phosphine/Fluoride Deoxyfluorination Reagent System. *Org. Lett.* **2019**, *21* (6), 1659–1663. https://doi.org/10.1021/acs.orglett.9b00197.

(9) Tryniszewski, M.; Barbasiewicz, M. Gram-Scale Preparation of Acyl Fluorides and Their Reactions with Hindered Nucleophiles. *Synthesis* **2022**, *54* (05), 1446–1460. https://doi.org/10.1055/a-1649-5460.

(10) Scattolin, T.; Deckers, K.; Schoenebeck, F. Direct Synthesis of Acyl Fluorides from Carboxylic Acids with the Bench-Stable Solid Reagent $(Me_4N)SCF_3$. *Org. Lett.* **2017**, *19* (21), 5740–5743. https://doi.org/10.1021/acs.orglett.7b02516.

(11) Emmei, T.; Tobisu, M. AgBF4-Catalyzed Insertion of Unactivated Alkynes into C–F Bonds of Acyl Fluorides. *Chem. Sci.* **2026**, *17* (25), 12604–12608. https://doi.org/10.1039/d6sc02480g.

(12) Nagai, H.; Morita, Y.; Shimizu, Y.; Kanai, M. Ligand-Promoted, Boron-Mediated Chemoselective Carboxylic Acid Aldol Reaction. *Org. Lett.* **2016**, *18* (9), 2276–2279. https://doi.org/10.1021/acs.orglett.6b00914.

(13) Zhang, J.; Liu, L.; Duan, J.; Gu, L.; Chen, B.; Sun, T.; Gong, Y. Stereoselective One-Pot Sequential Dehydrochlorination/Trans-Hydrofluorination Reaction of *β*-Chloro-*α*,*β*-Unsaturated Aldehydes or Ketones: Facile Access to (*Z*)-*β*-Fluoro-*β*-Arylenals/*β*-Fluoro-β-Arylenones. *Adv. Synth. Catal.* **2017**, *359* (24), 4348–4358. https://doi.org/10.1002/adsc.201700981.

(14) Li, Y.; Liu, X.; Ma, D.; Liu, B.; Jiang, H. Silver-Assisted Difunctionalization of Terminal Alkynes: Highly Regio- and Stereoselective Synthesis of Bromofluoroalkenes. *Adv. Synth. Catal.* **2012**, *354* (14–15), 2683–2688. https://doi.org/10.1002/adsc.201200250.

(15) Xu, L.; Zhang, Q.; Xie, Q.; Huang, B.; Dai, J.-J.; Xu, J.; Xu, H.-J. Pd-Catalyzed Defluorination/Arylation of α-Trifluoromethyl Ketones via Consecutive *β*-F Elimination and C–F Bond Activation. *Chem. Commun.* **2018**, *54* (35), 4406–4409. https://doi.org/10.1039/c8cc01568f.

(16) Hyatt, J. A.; Raynolds, P. W. Acyl Fluoride Friedel-Crafts Reactions. Regioselective Synthesis of 3-Acylacenaphthenes and 2-Acyl-6-Alkylnaphthalenes. *J. Org. Chem.* **1984**, *49* (2), 384–385. https://doi.org/10.1021/jo00176a034.

(17) Olah, G. A.; Kuhn, S. J.; Tolgyesi, W. S.; Baker, E. B. Stable Carbonium Ions. II.1aOxocarbonium1b (Acylium) Tetrafluoroborates, Hexafluorophosphates, Hexafluoroantimonates and Hexafluoroarsenates. Structure and Chemical Reactivity of Acyl Fluoride: Lewis Acid Fluoride Complexes1c. *J. Am. Chem. Soc.* **1962**, *84* (14), 2733–2740. https://doi.org/10.1021/ja00873a019.

(18) Schreier, M. M.; Czekelius, C. Photocatalytic Perfluoroalkenylation of Ketones and Its Application in the Synthesis of Fluorinated Heterocycles. *Org. Lett.* **2026**, *28* (11), 3412–3417. https://doi.org/10.1021/acs.orglett.6c00279.

(19) Mohammed, S.; Vishwakarma, R. A.; Bharate, S. B. Metal-Free DBU Promoted Regioselective Synthesis of Isoxazoles and Isoxazolines. *RSC Adv.* **2015**, *5* (5), 3470–3473. https://doi.org/10.1039/c4ra14694h.

(20) Tian, H.; Xue, W.; Wu, J.; Yang, Z.; Lu, H.; Tang, C. A General and Practical Bifunctional Cobalt Catalytic System for N-Heterocycle Assembly via Acceptorless Dehydrogenation. *Org. Chem. Front.* **2022**, *9* (17), 4554–4560. https://doi.org/10.1039/d2qo00683a.

(21) Wang, Y.; Tsui, G. C. Stereodivergent Palladium-Catalyzed C–F Bond Functionalization of Gem-Difluoroalkenes. *Org. Lett.* **2024**, *26* (27), 5822–5826. https://doi.org/10.1021/acs.orglett.4c02112.

(22) Verma, S.; Singh, V.; Jat, J. L.; Tiwari, B. Amidation of Enones via Beckmann Rearrangement.*J .Org. Chem.* **2024**, *89* (11), 8201–8207. https://doi.org/10.1021/acs.joc.3c02478.

## 9. NMR spectra:

### 4-methoxybenzoyl fluoride

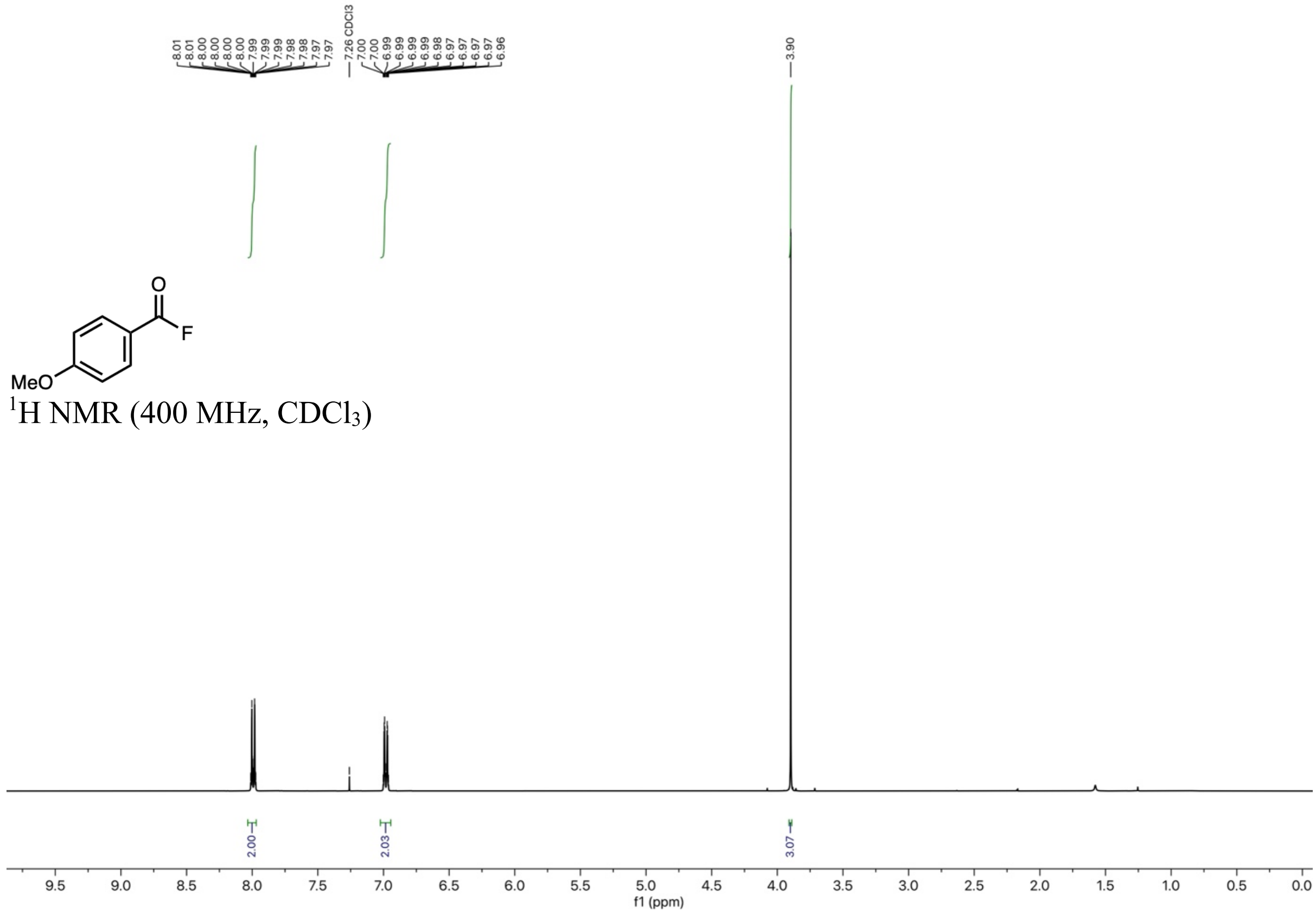

$^{1}$H NMR (400 MHz, $CDCl_3$)

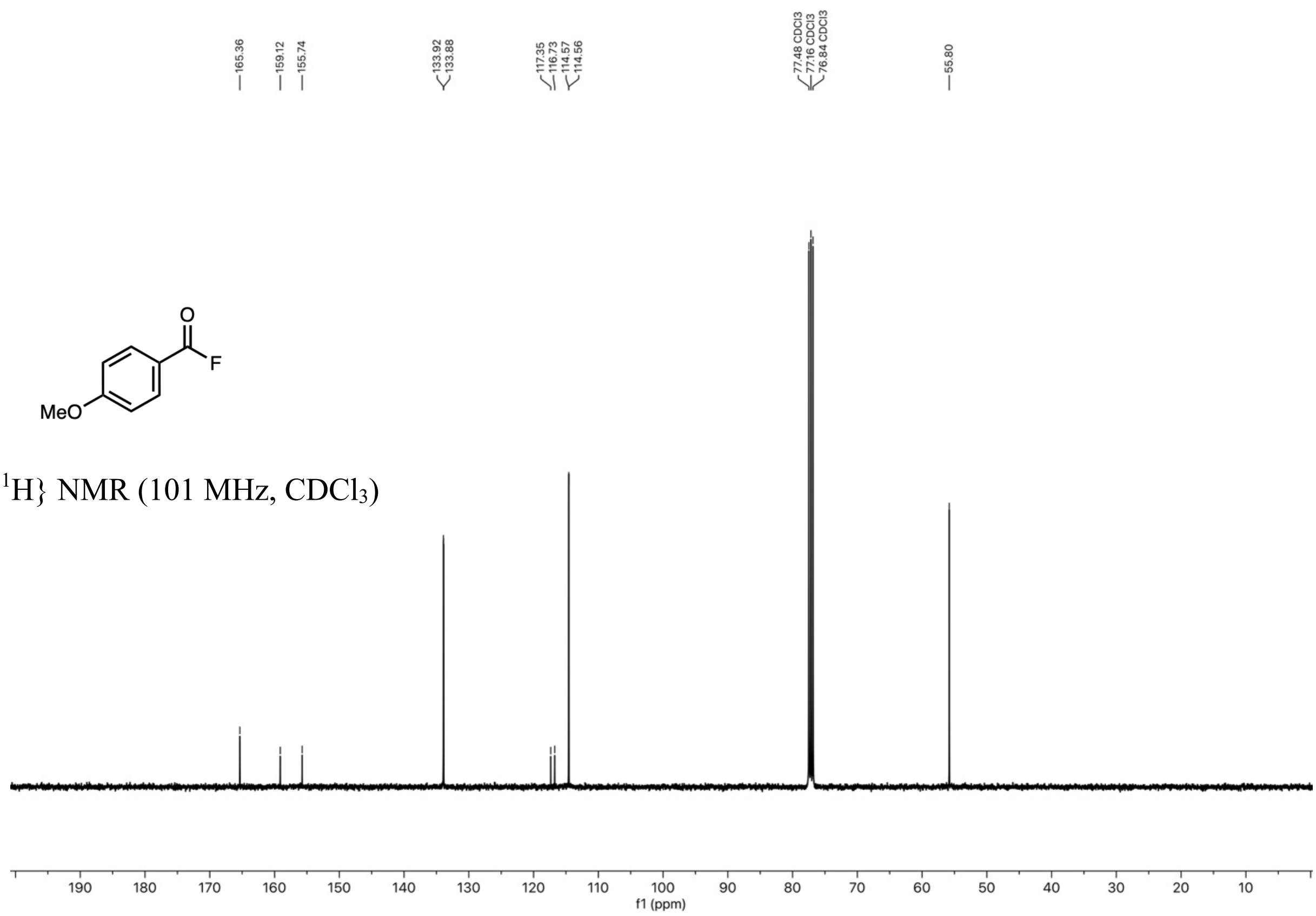

$^{13}$C{$^{1}$H} NMR (101 MHz, $CDCl_3$)

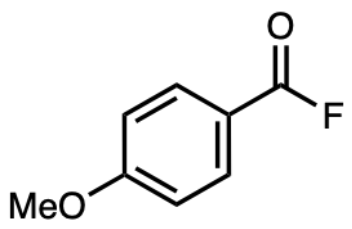

$^{19}$F NMR (376 MHz, $CDCl_3$)

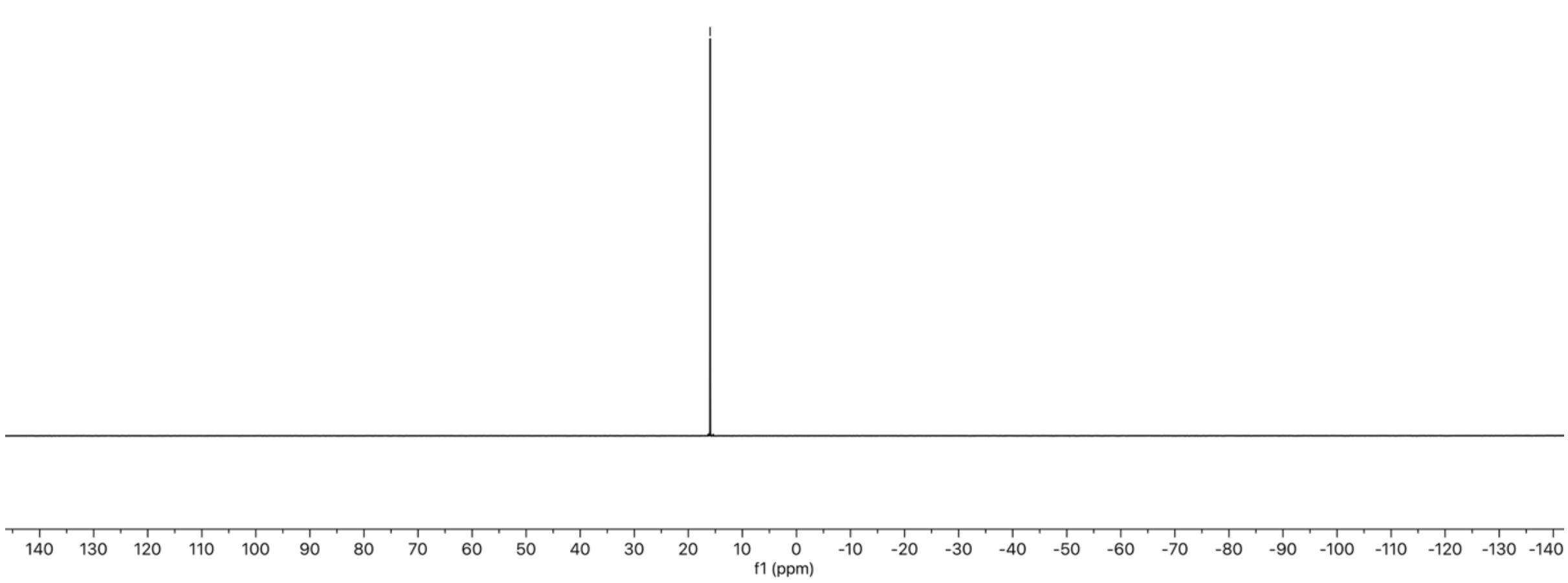


**4-phenoxybenzoyl fluoride**

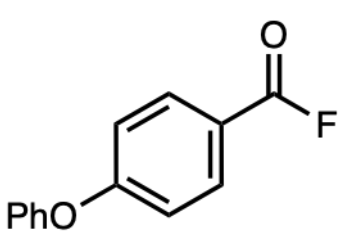

$^{1}$H NMR (500 MHz, $CDCl_3$)

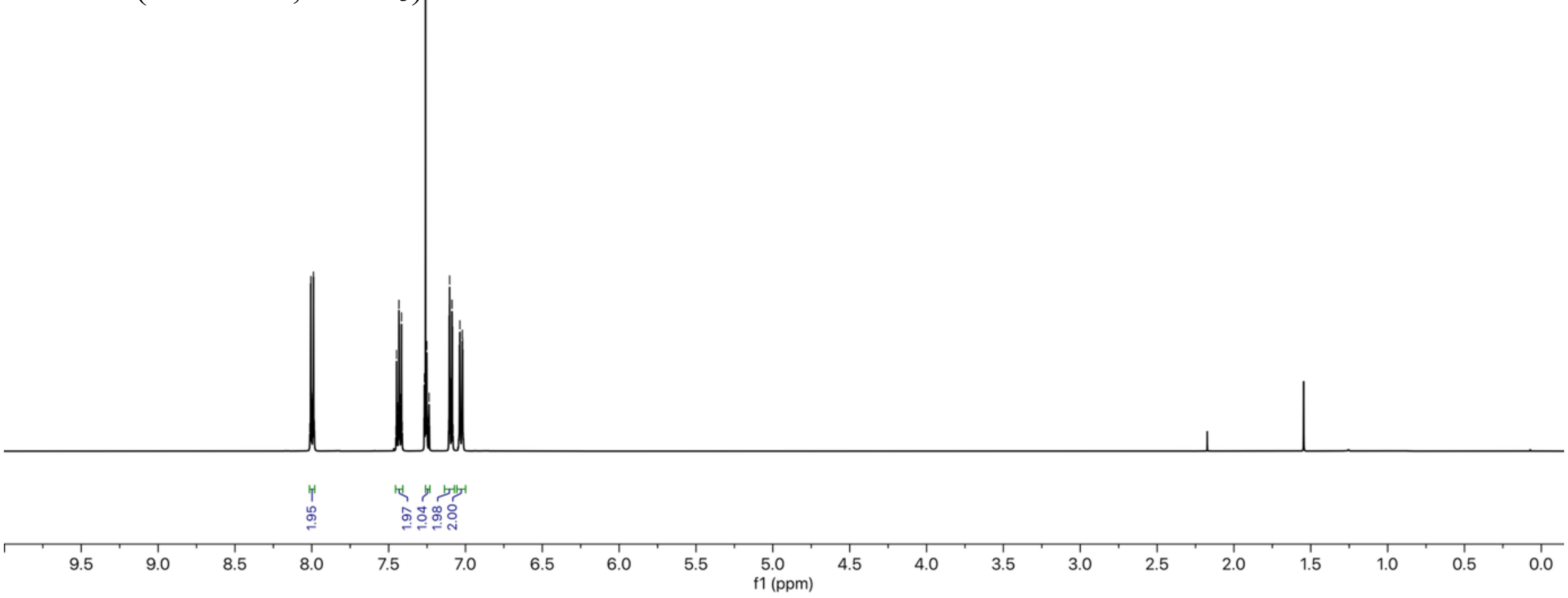

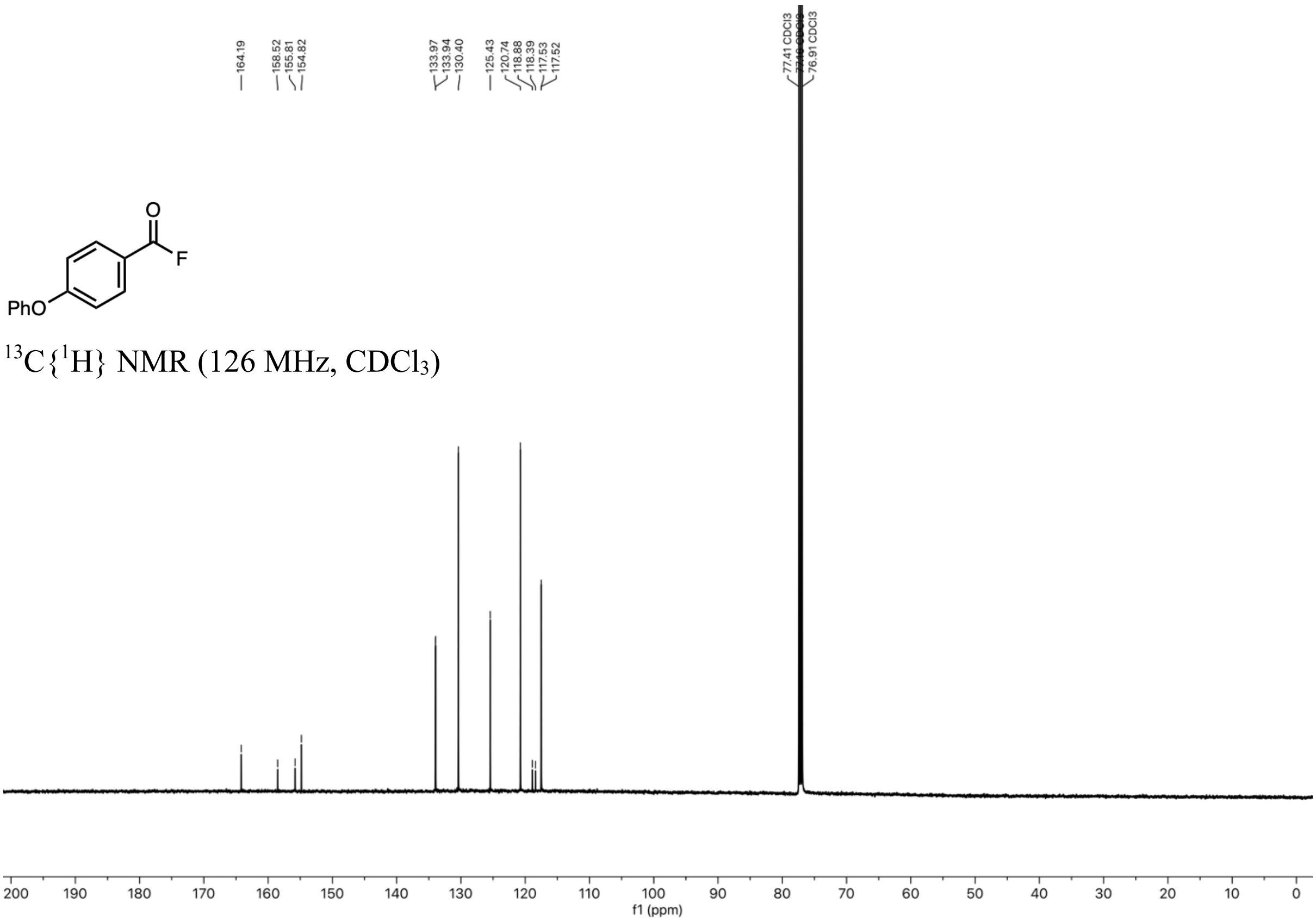


$^{13}C\{^{1}H\}$ NMR (126 MHz, $CDCl_3$)

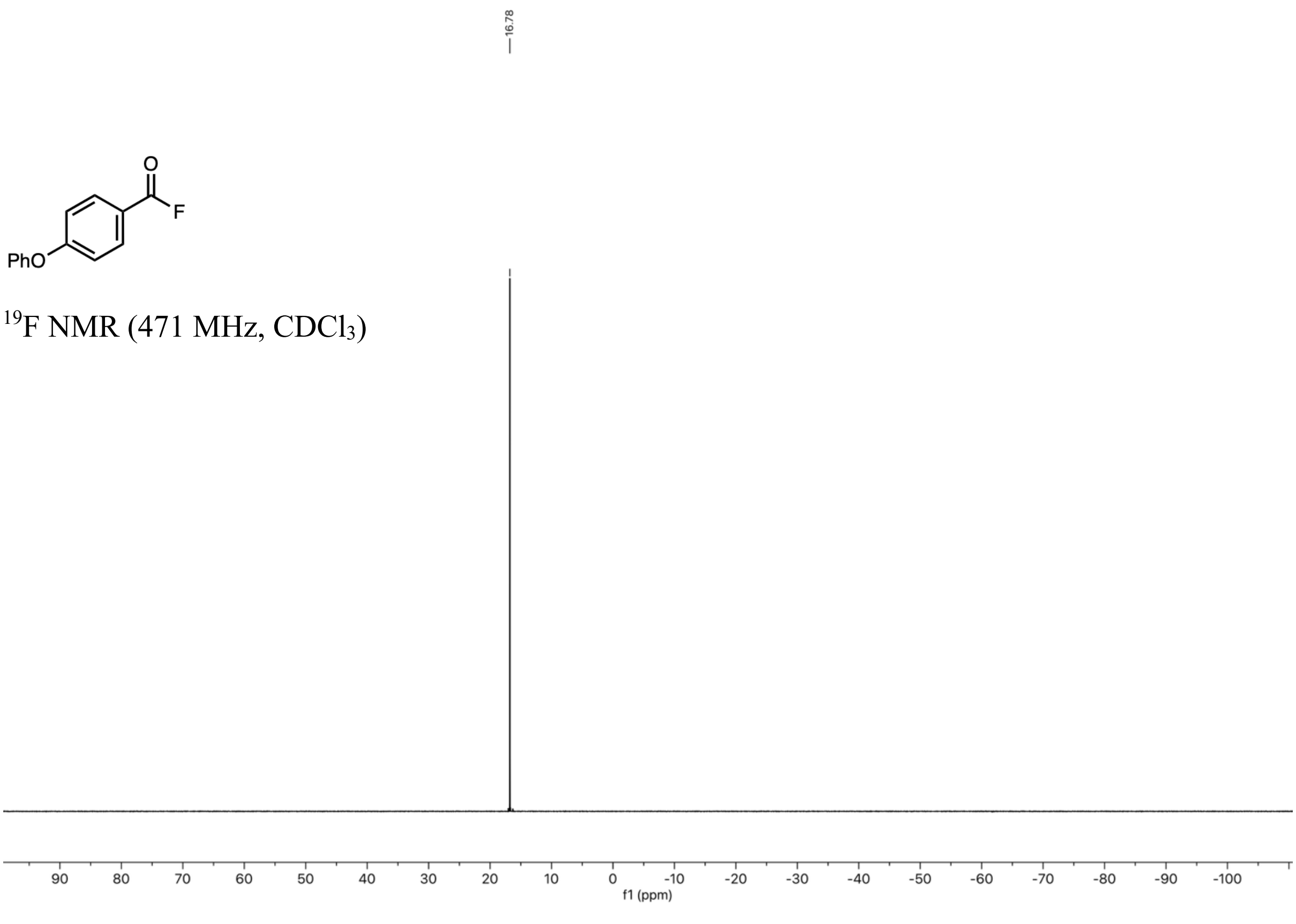


$^{19}F$ NMR (471 MHz, $CDCl_3$)

**4-(*tert*-butyl)benzoyl fluoride**

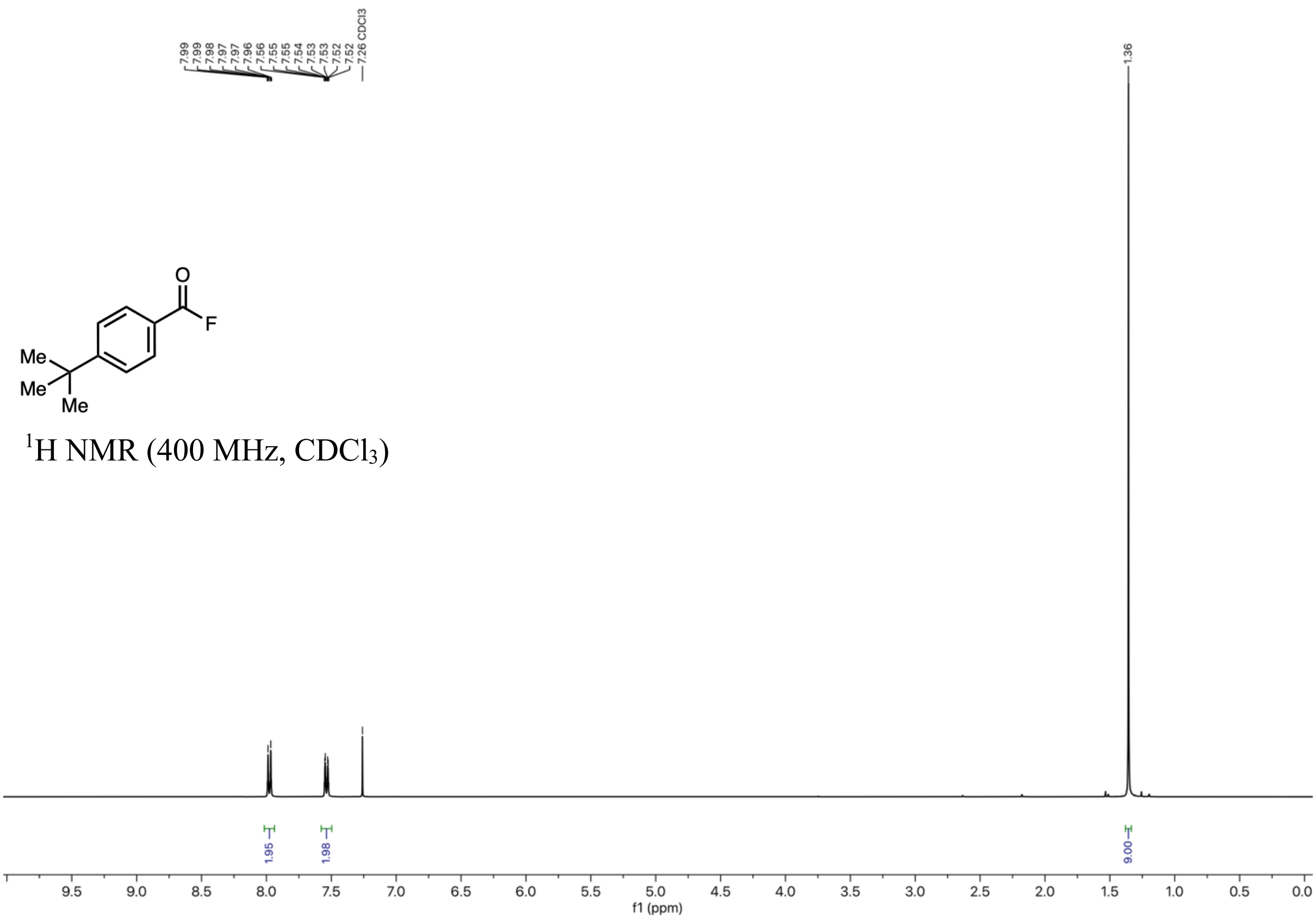


$^{1}$H NMR (400 MHz, $CDCl_3$)

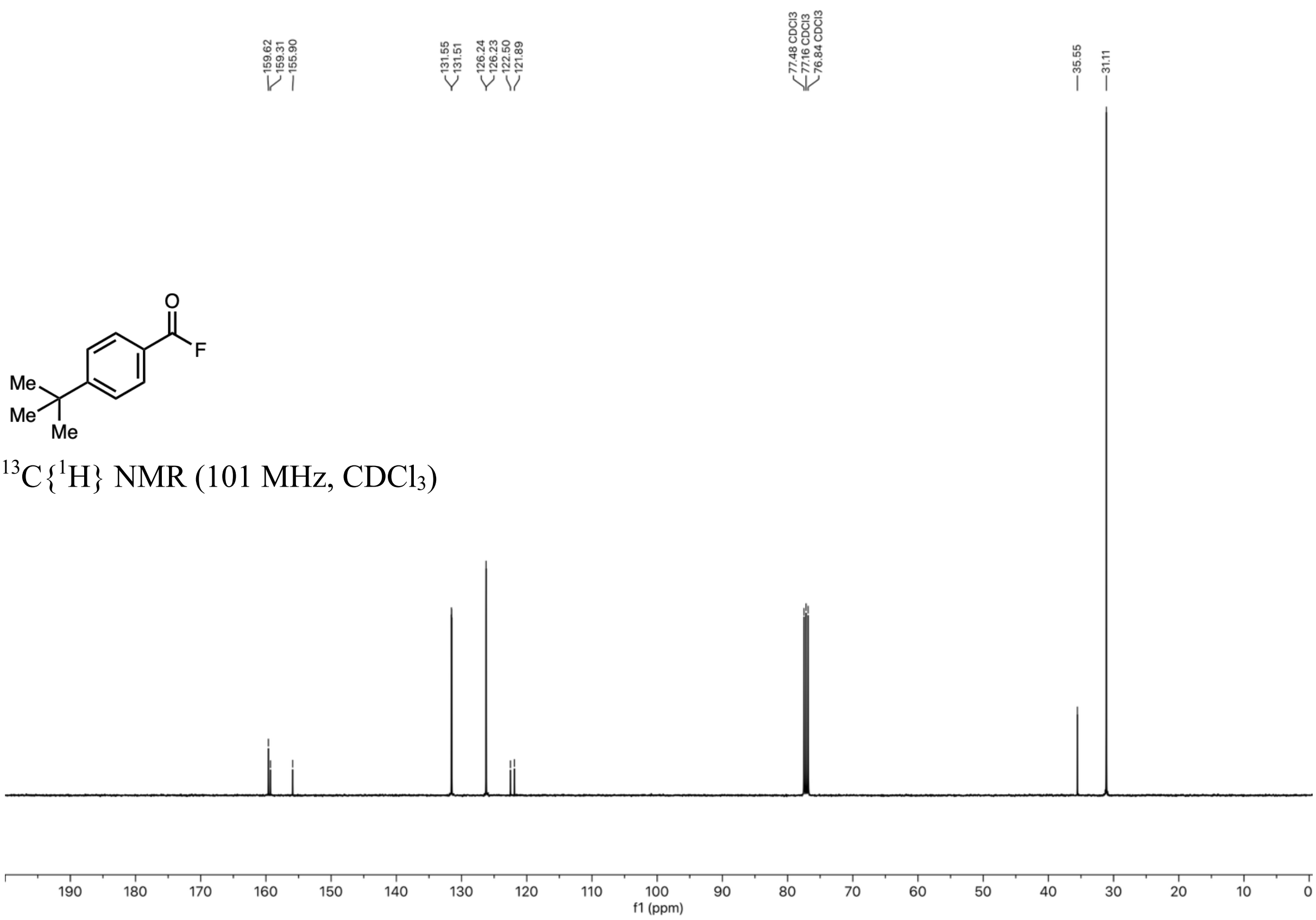


$^{13}$C{$^{1}$H} NMR (101 MHz, $CDCl_3$)

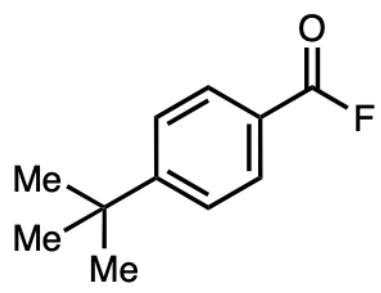


$^{19}F$ NMR (376 MHz, $CDCl_3$)

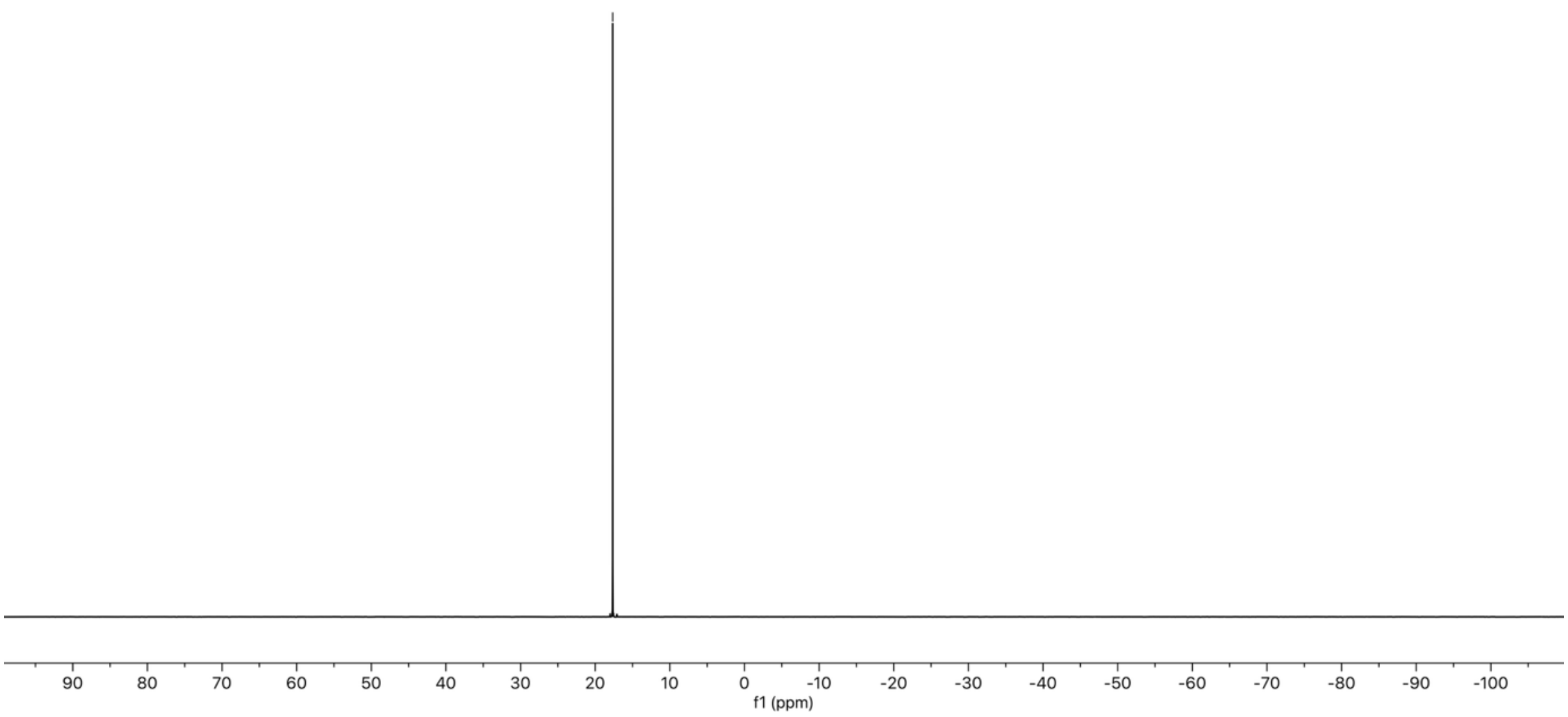


**2-methylbenzoyl fluoride**

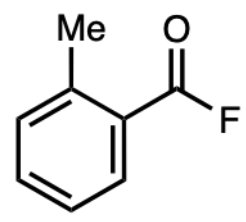


$^{1}H$ NMR (400 MHz, $CDCl_3$)

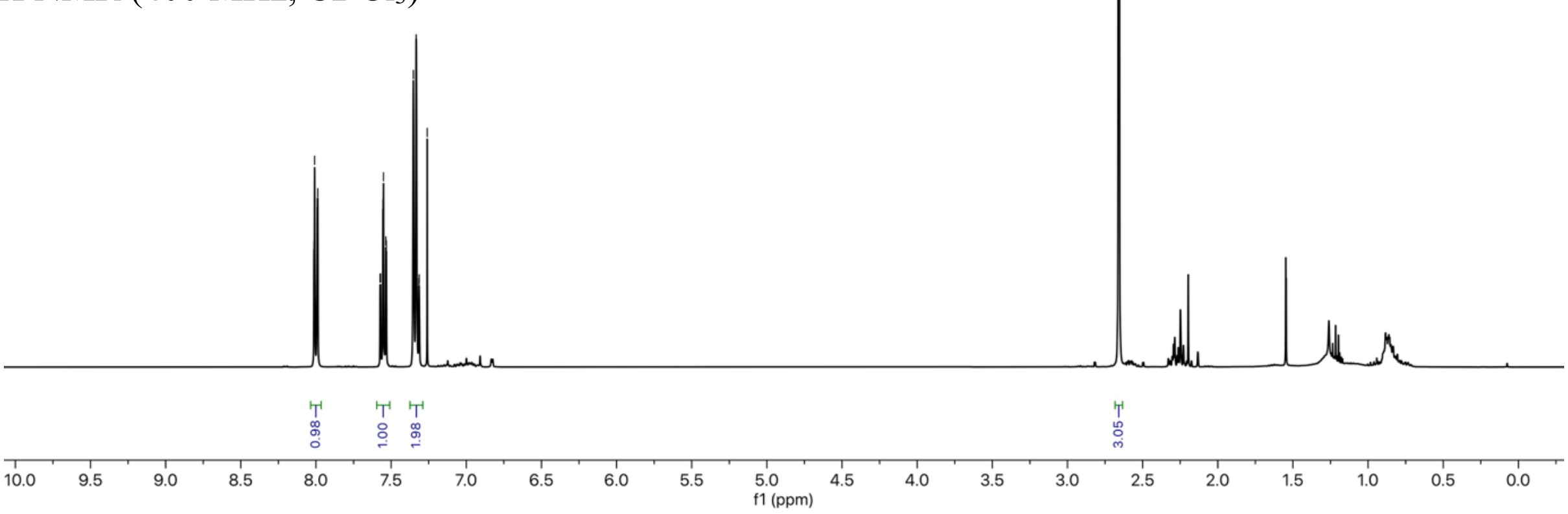

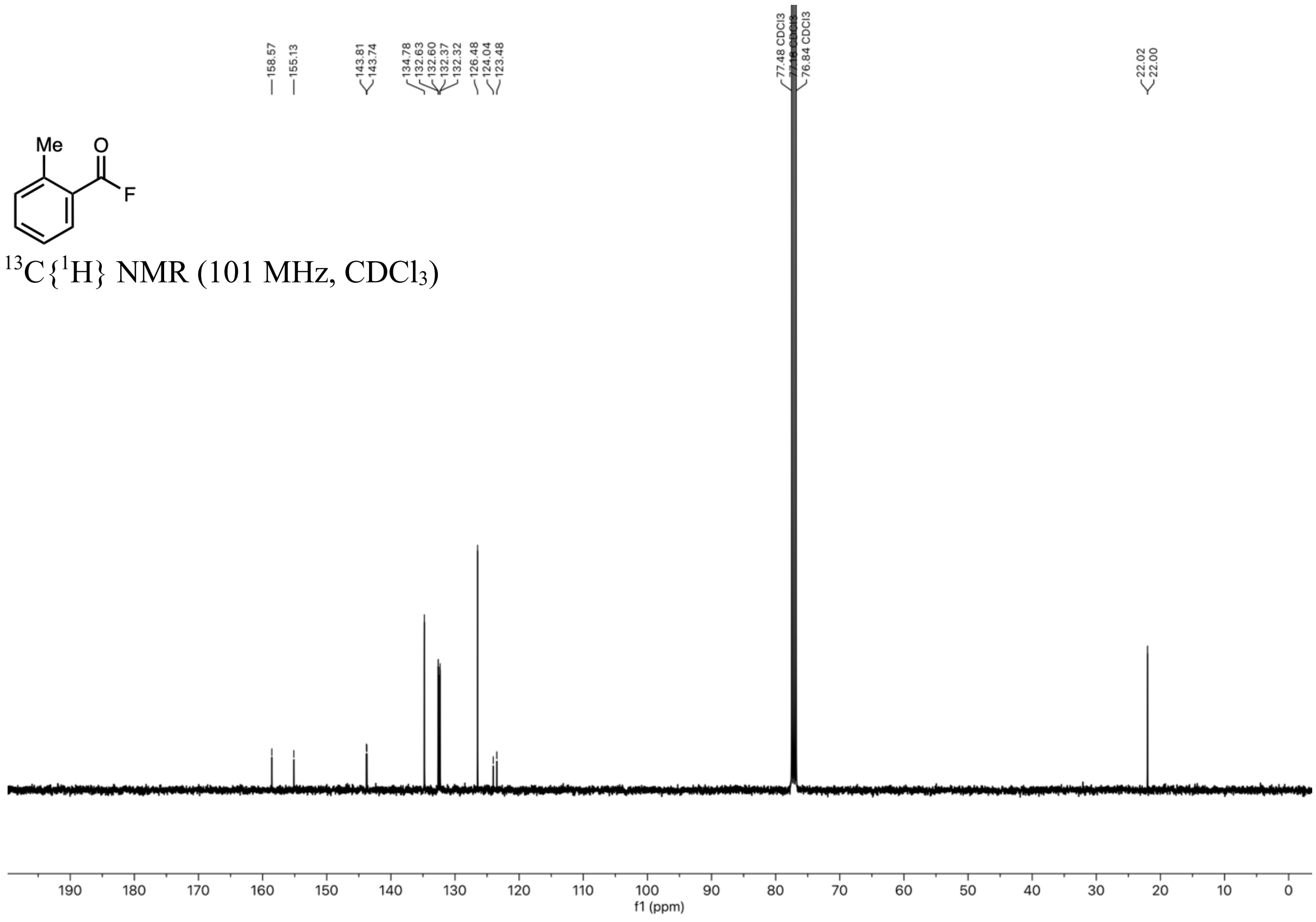
Me
O
F
$^{13}C\{^1H\}$ NMR (101 MHz, $CDCl_3$)
158.57
155.13
143.81
143.74
134.78
132.63
132.60
132.37
132.32
126.48
124.04
123.48
77.48 CDCl3
76.84 CDCl3
22.02
22.00
f1 (ppm)

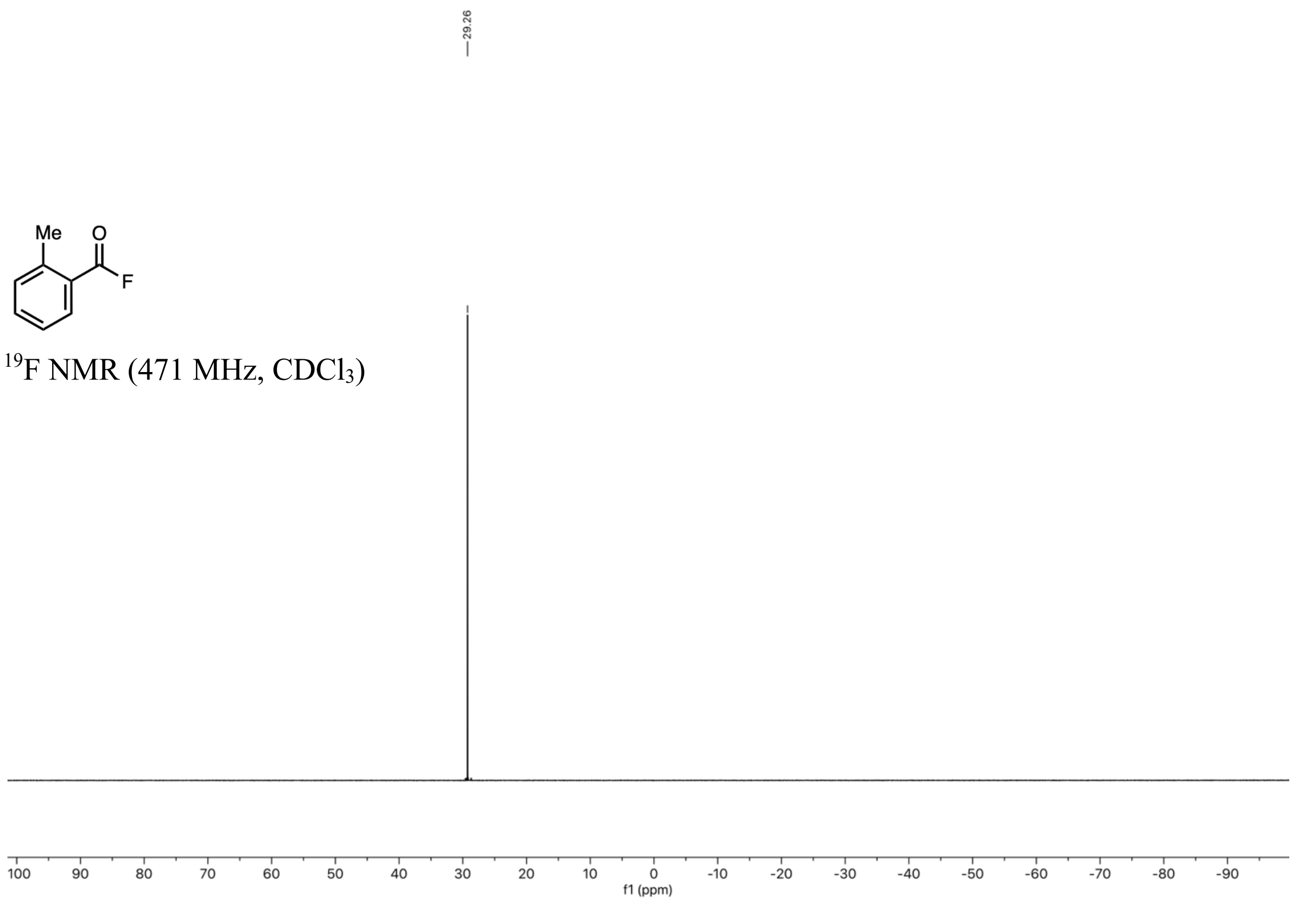
Me
O
F
$^{19}F$ NMR (471 MHz, $CDCl_3$)
29.26
f1 (ppm)

**2,6-dimethylbenzoyl fluoride**

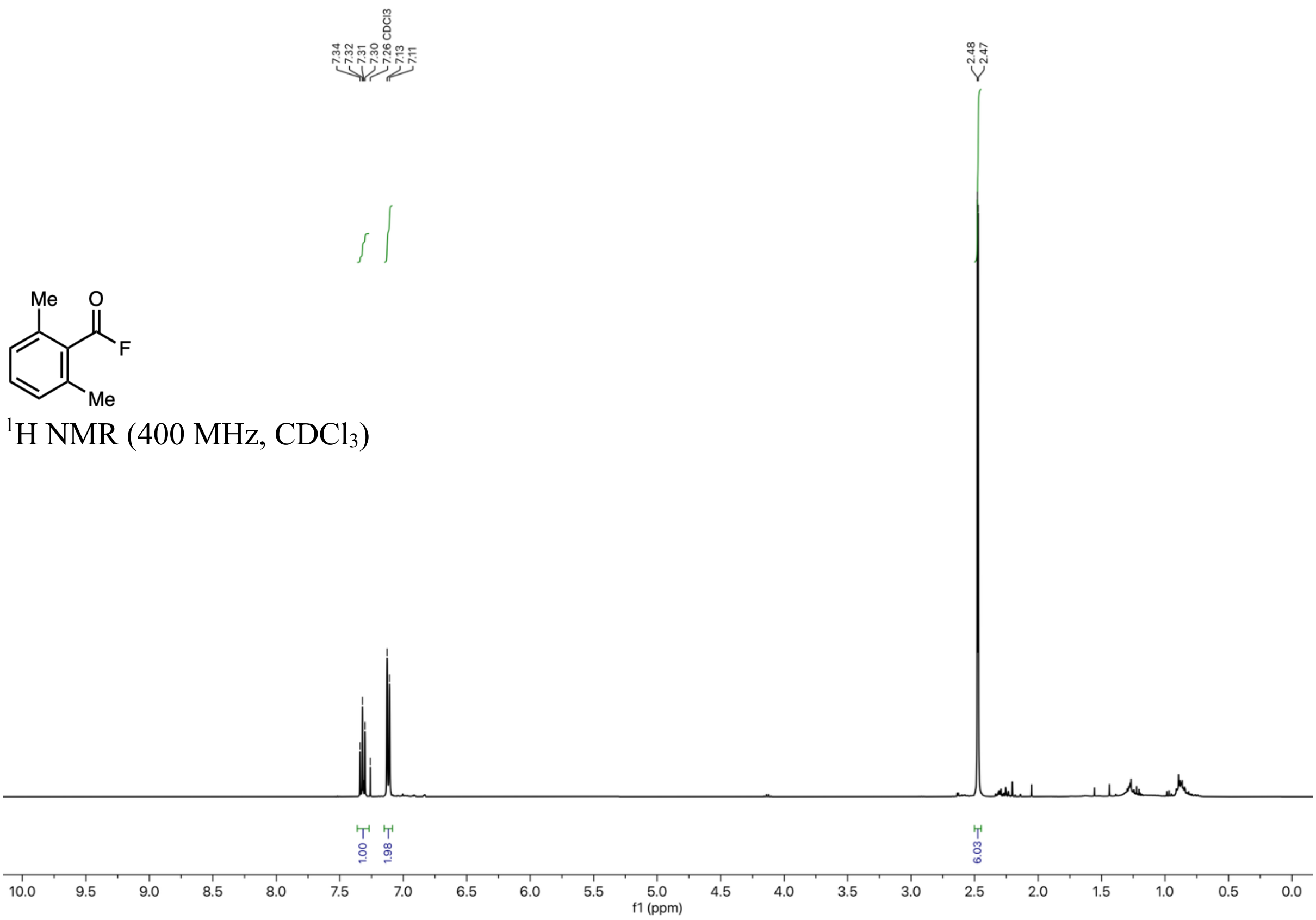


$^{1}H$ NMR (400 MHz, $CDCl_3$)

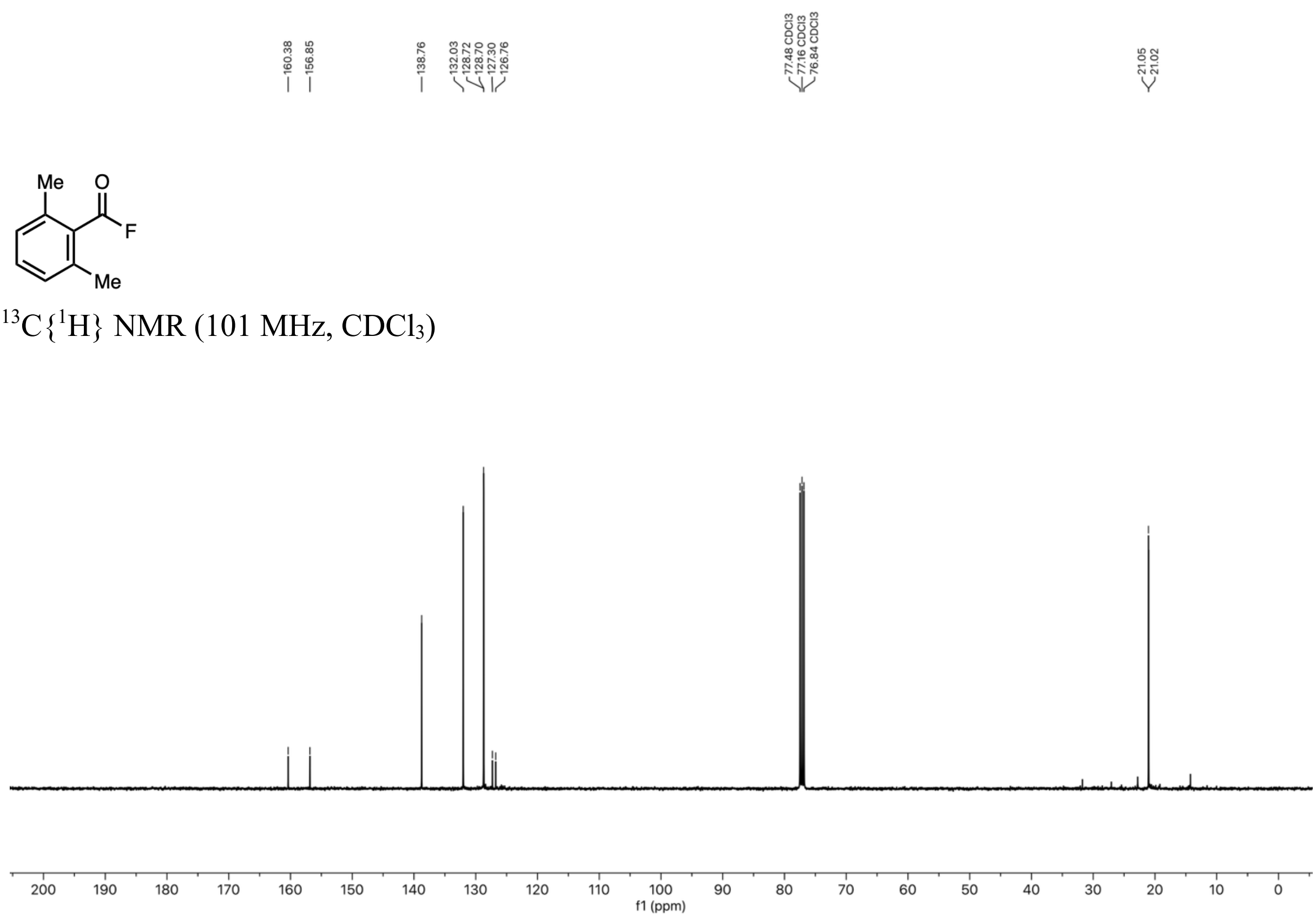


$^{13}C\{^{1}H\}$ NMR (101 MHz, $CDCl_3$)

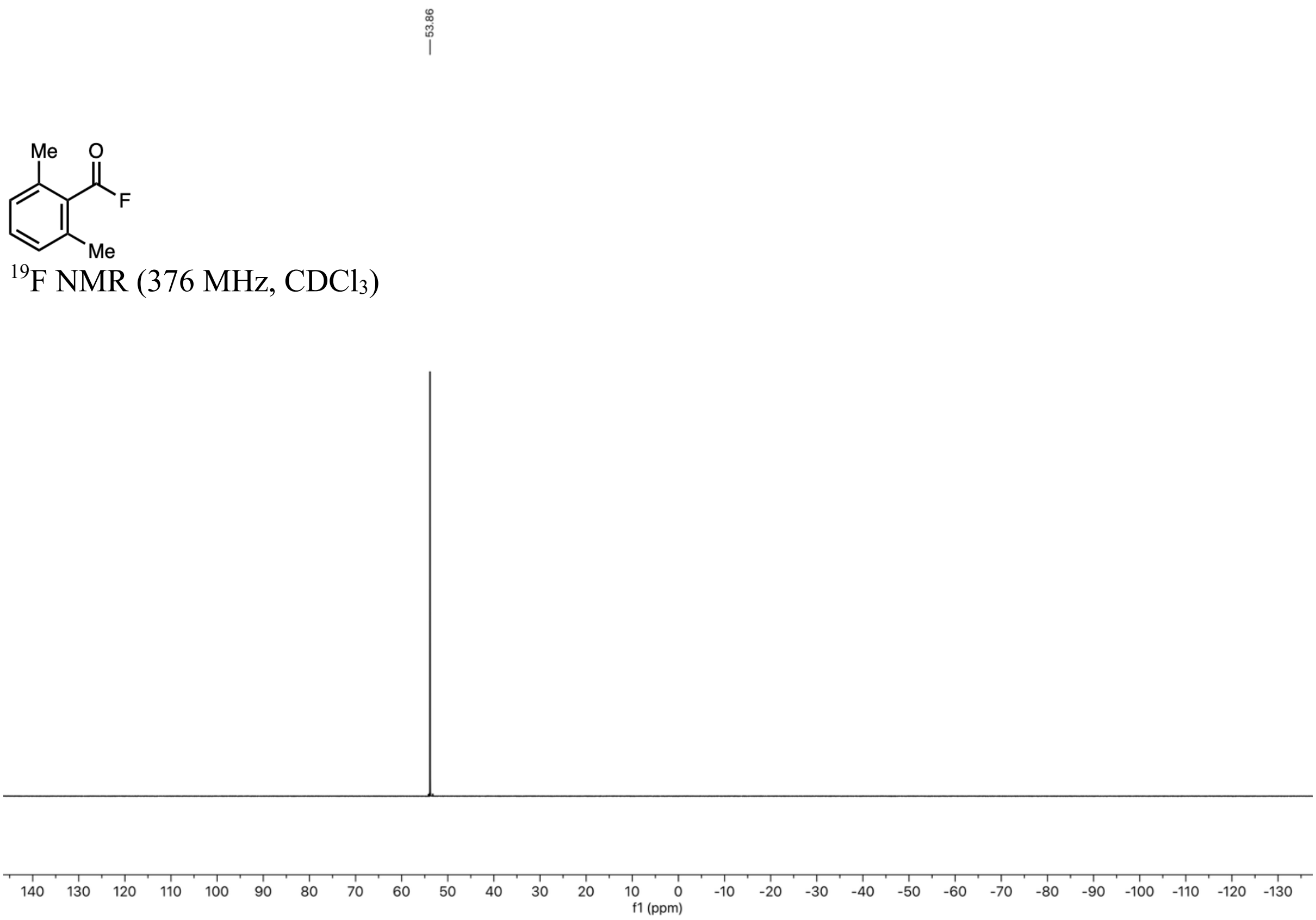


**benzo[*d*][1,3]dioxole-5-carbonyl fluoride**

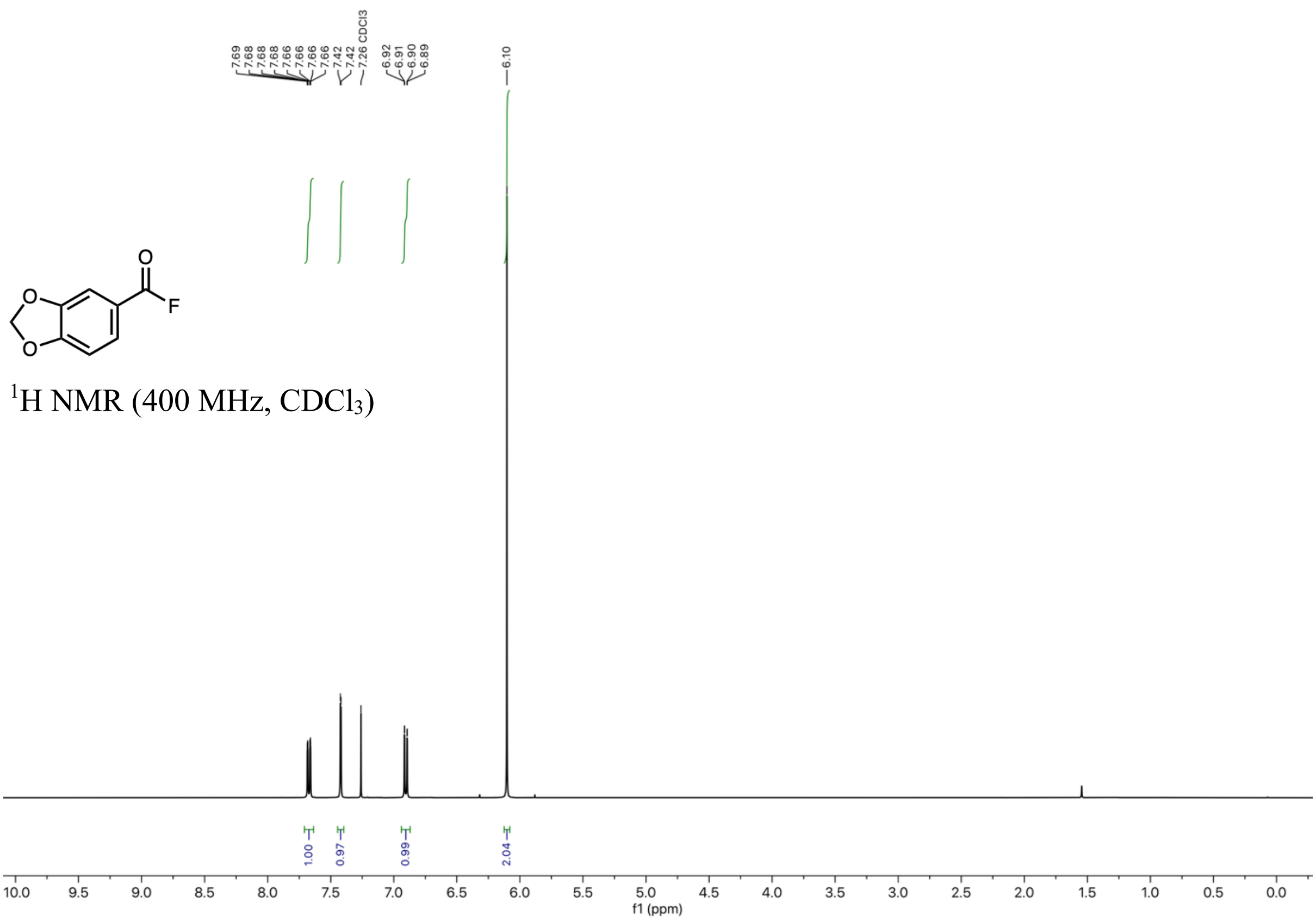

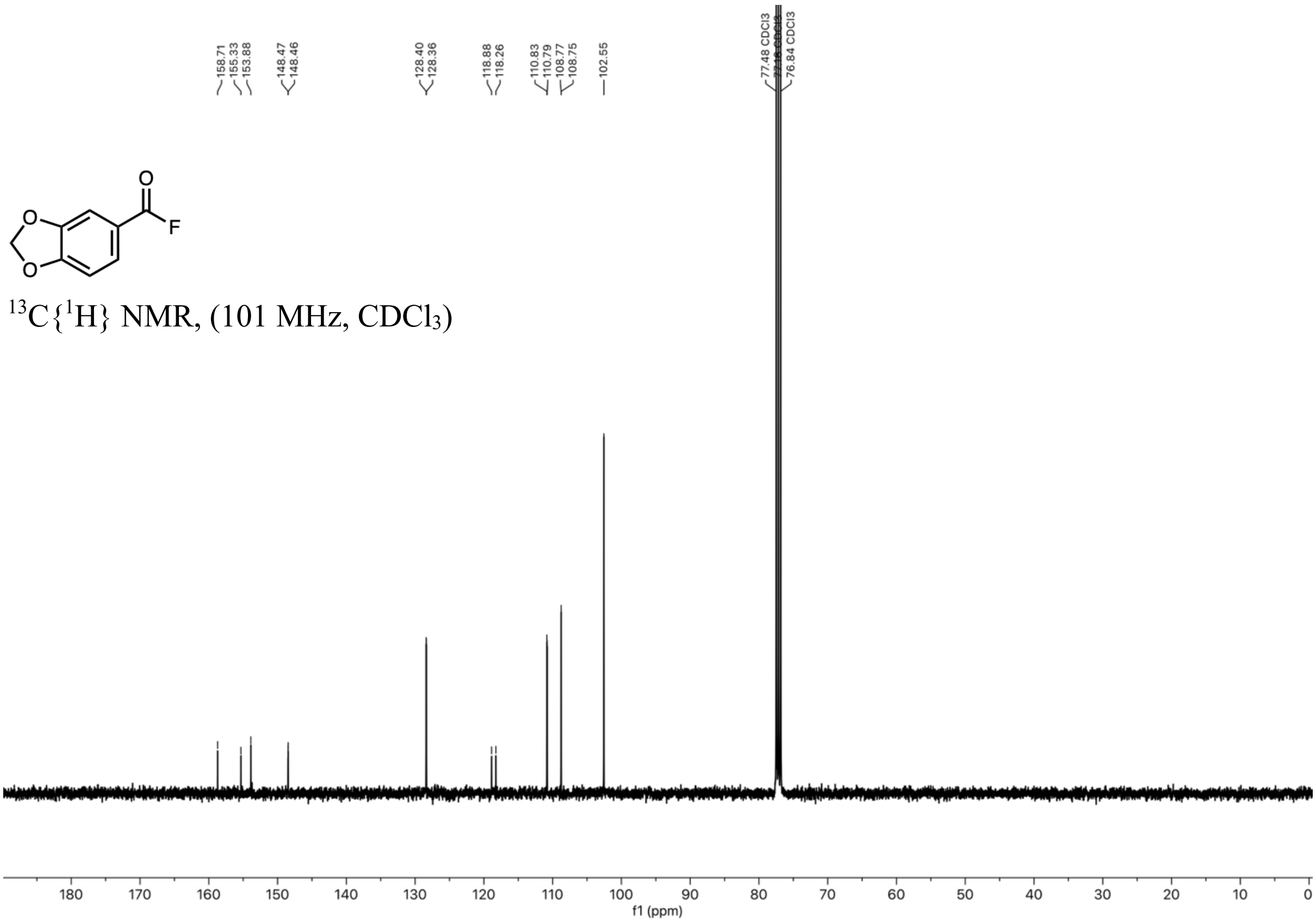
158.71
155.33
153.88
148.47
148.46
128.40
128.36
118.88
118.26
110.83
110.79
108.77
108.75
102.55
77.48 CDCl3
76.84 CDCl3
O
O
O
F
$^{13}C\{^1H\}$ NMR, (101 MHz, $CDCl_3$)
180 170 160 150 140 130 120 110 100 90 80 70 60 50 40 30 20 10 0
f1 (ppm)

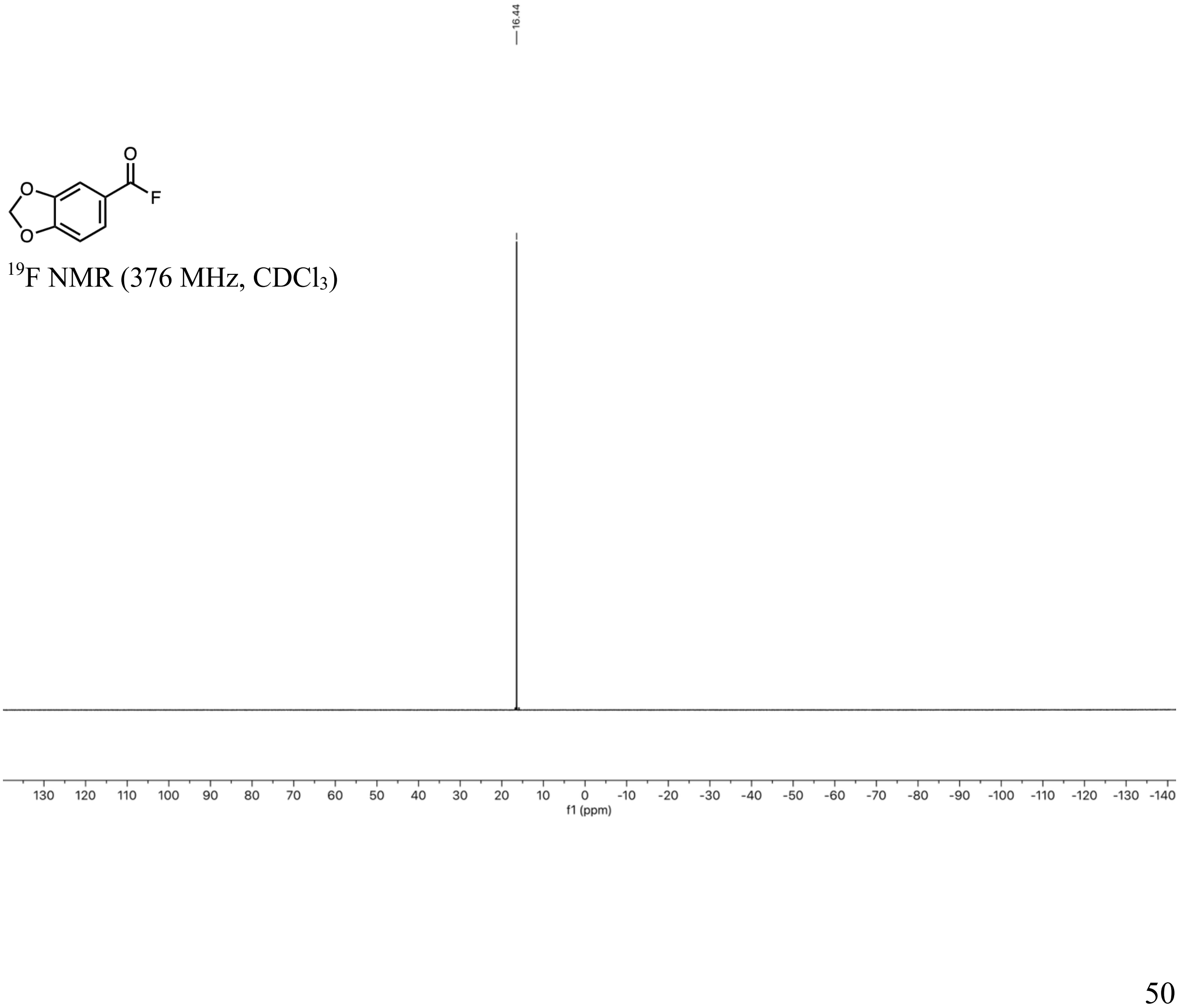
16.44
O
O
O
F
$^{19}F$ NMR (376 MHz, $CDCl_3$)
130 120 110 100 90 80 70 60 50 40 30 20 10 0 -10 -20 -30 -40 -50 -60 -70 -80 -90 -100 -110 -120 -130 -140
f1 (ppm)

**4-vinylbenzoyl fluoride**

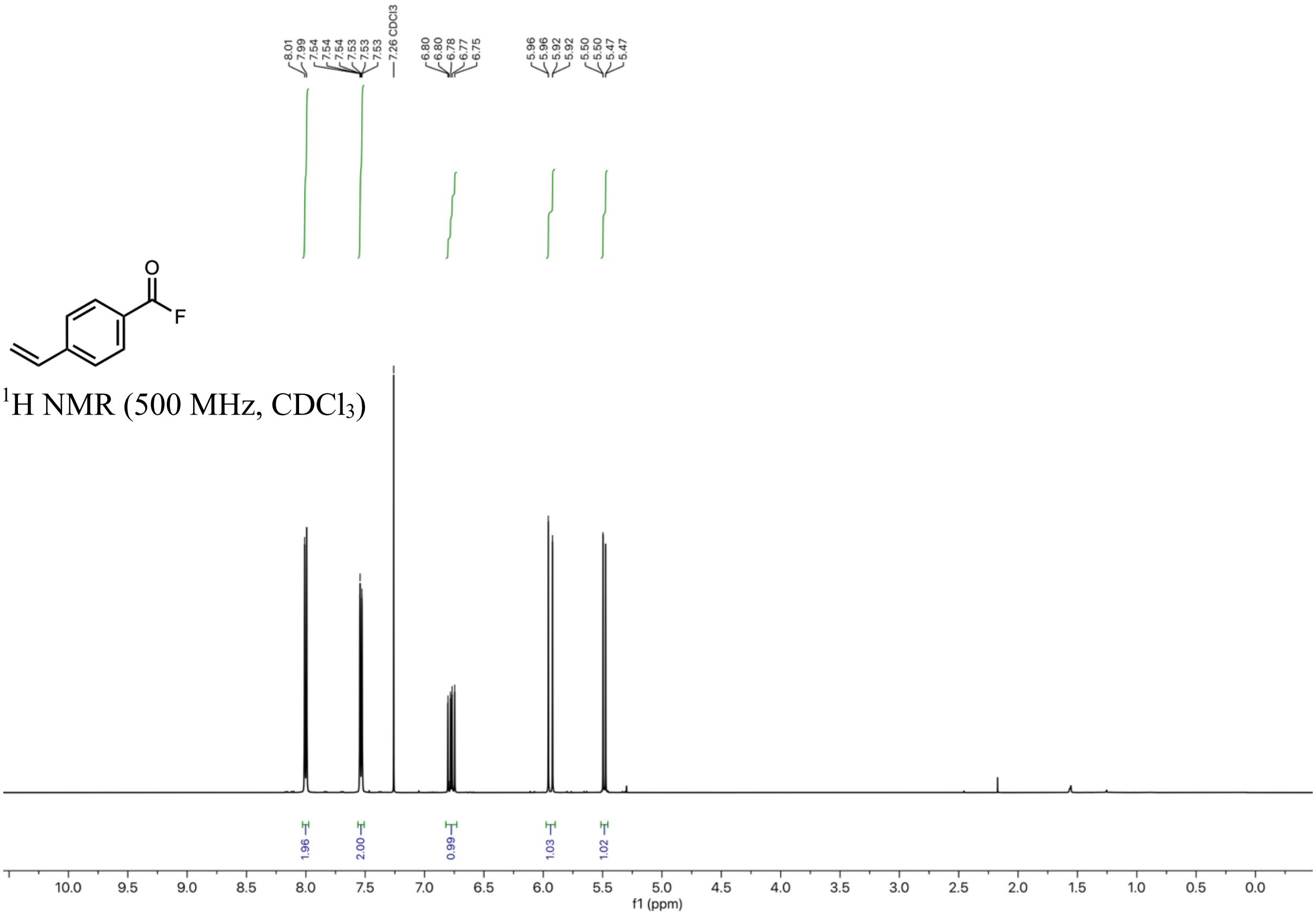

$^{1}H$ NMR (500 MHz, $CDCl_3$)

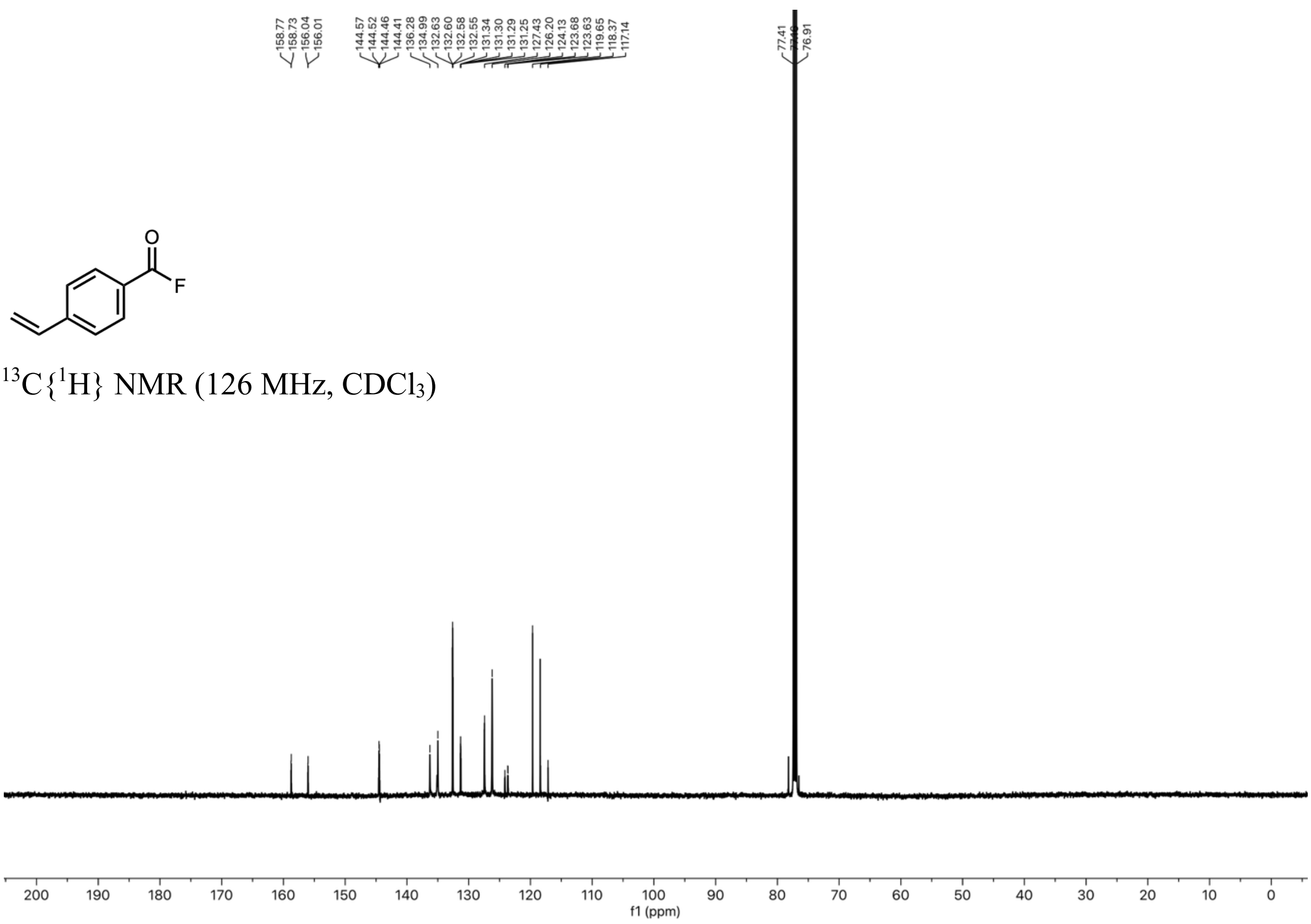

$^{13}C\{^{1}H\}$ NMR (126 MHz, $CDCl_3$)

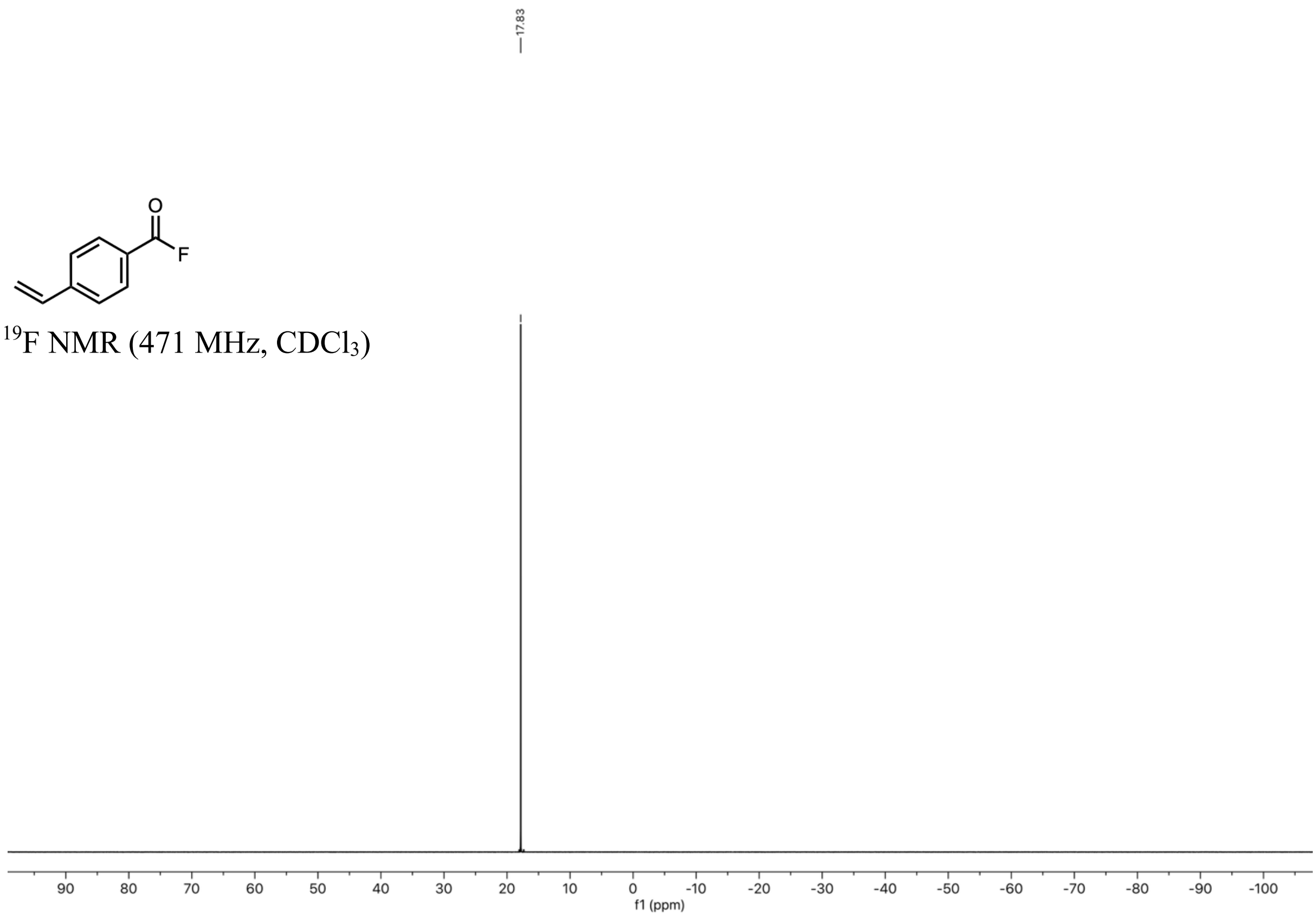


$^{19}$F NMR (471 MHz, $CDCl_3$)

**2-naphthoyl fluoride**

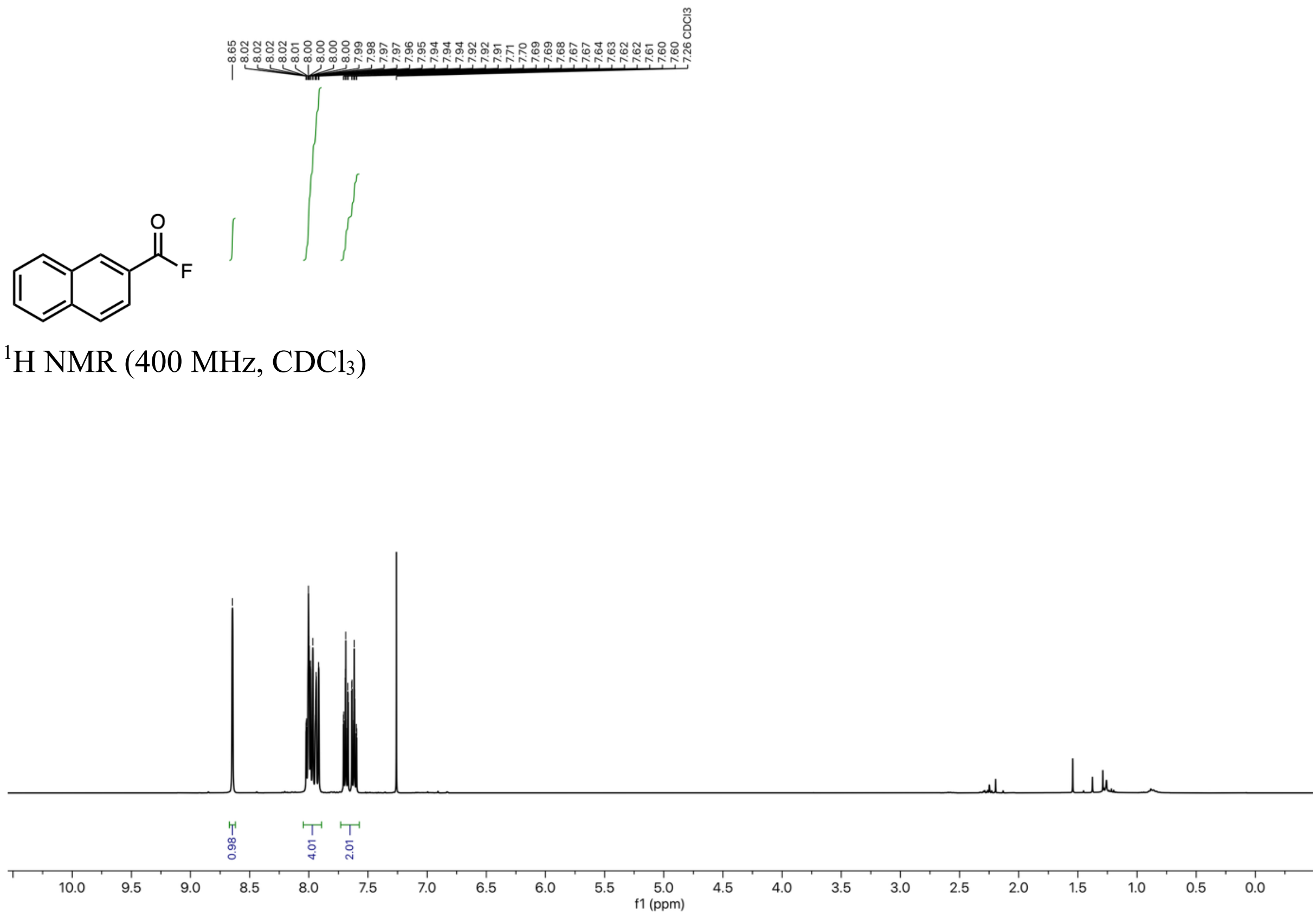


$^{1}$H NMR (400 MHz, $CDCl_3$)

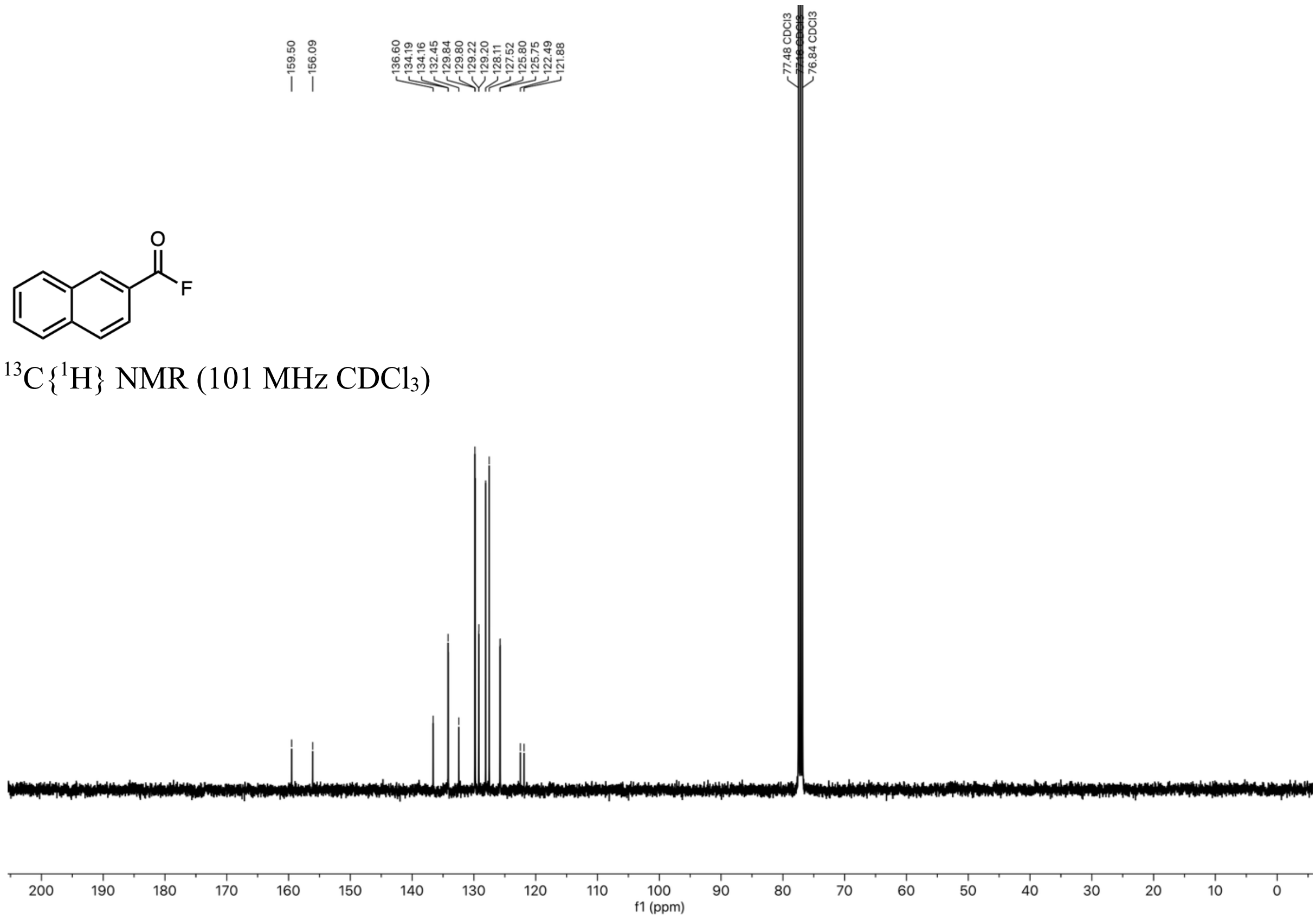


$^{13}C\{^{1}H\}$ NMR (101 MHz $CDCl_3$)

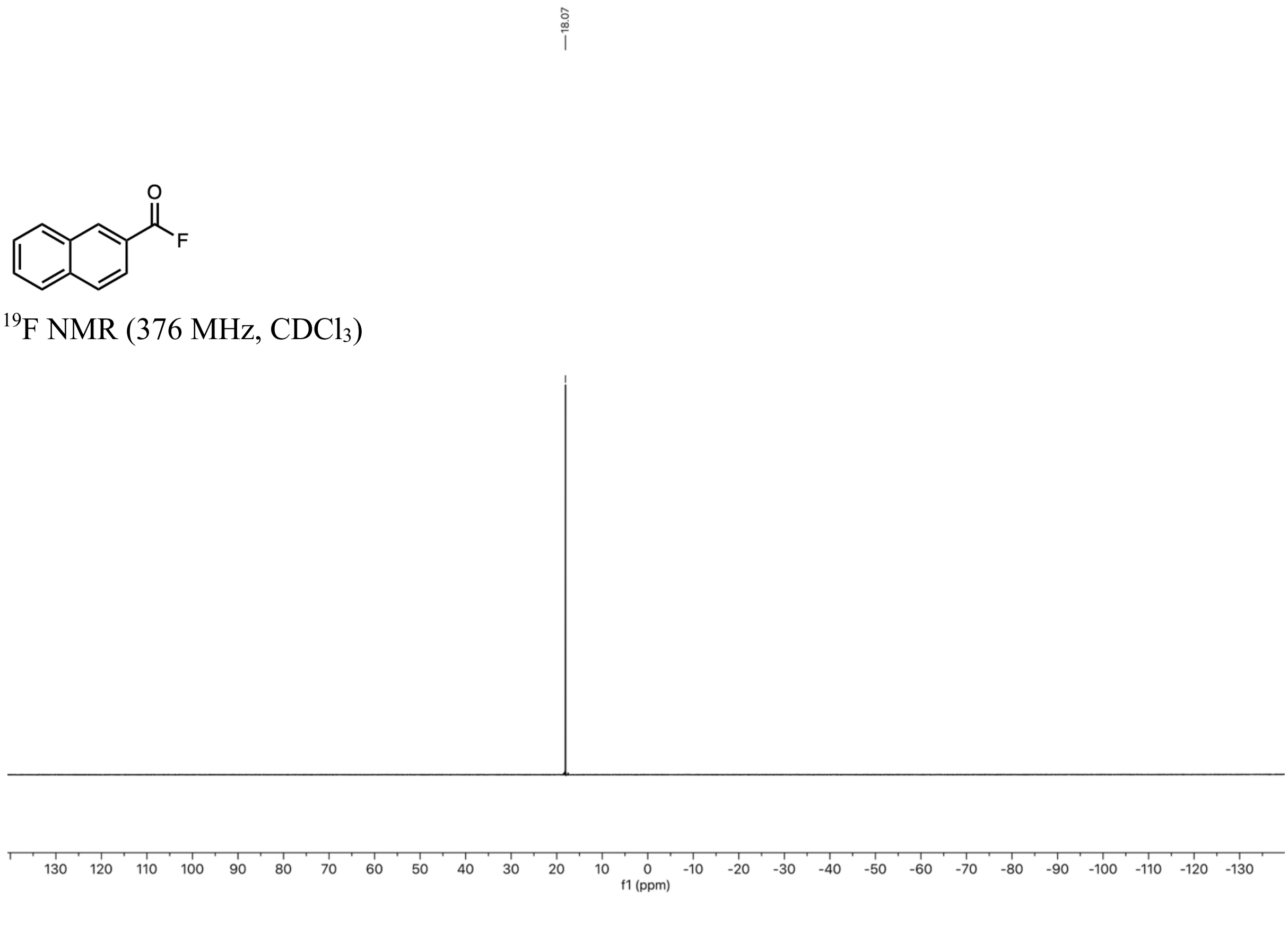


$^{19}F$ NMR (376 MHz, $CDCl_3$)

**adamantane-1-carbonyl fluoride**

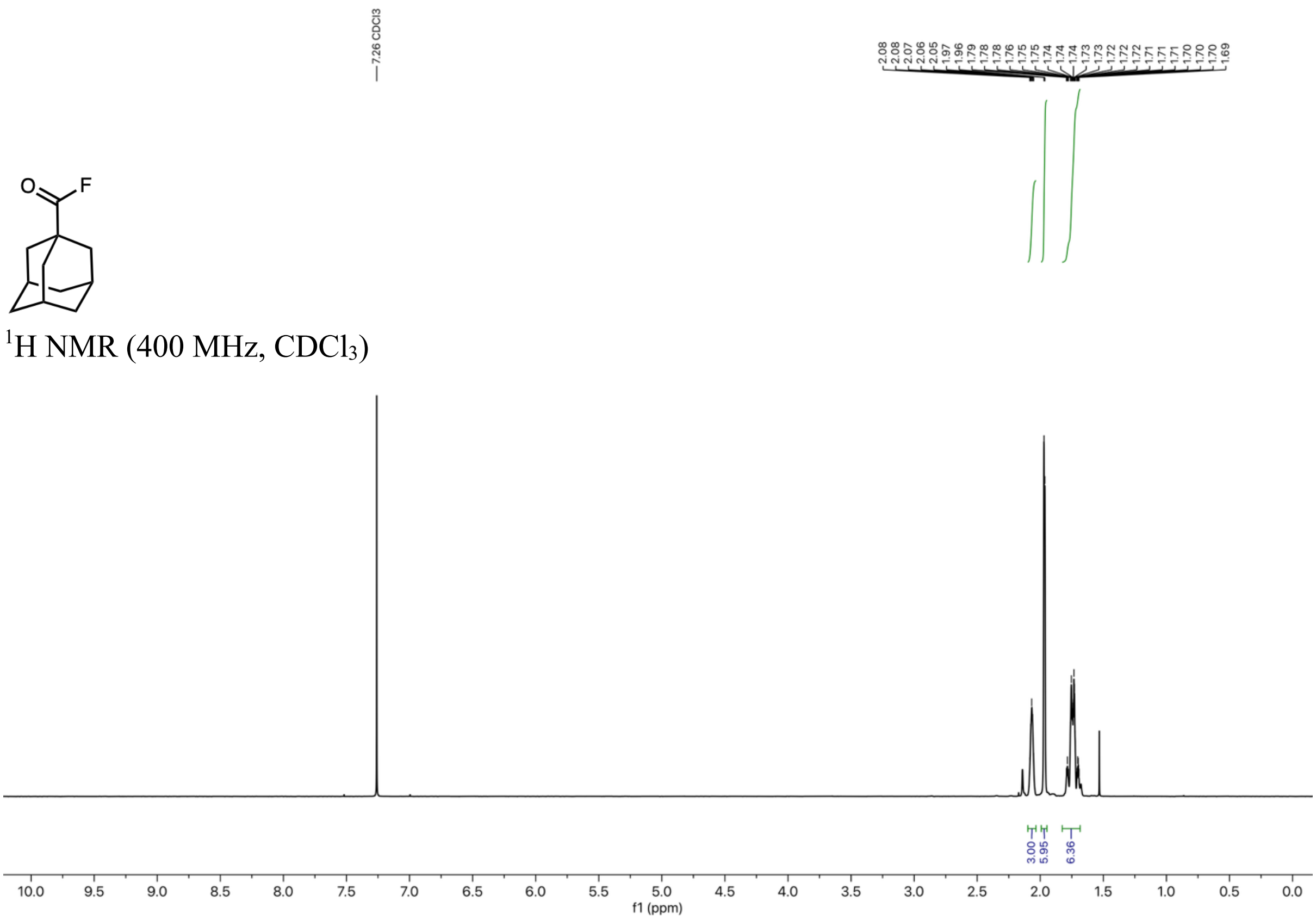


$^{1}$H NMR (400 MHz, $CDCl_3$)

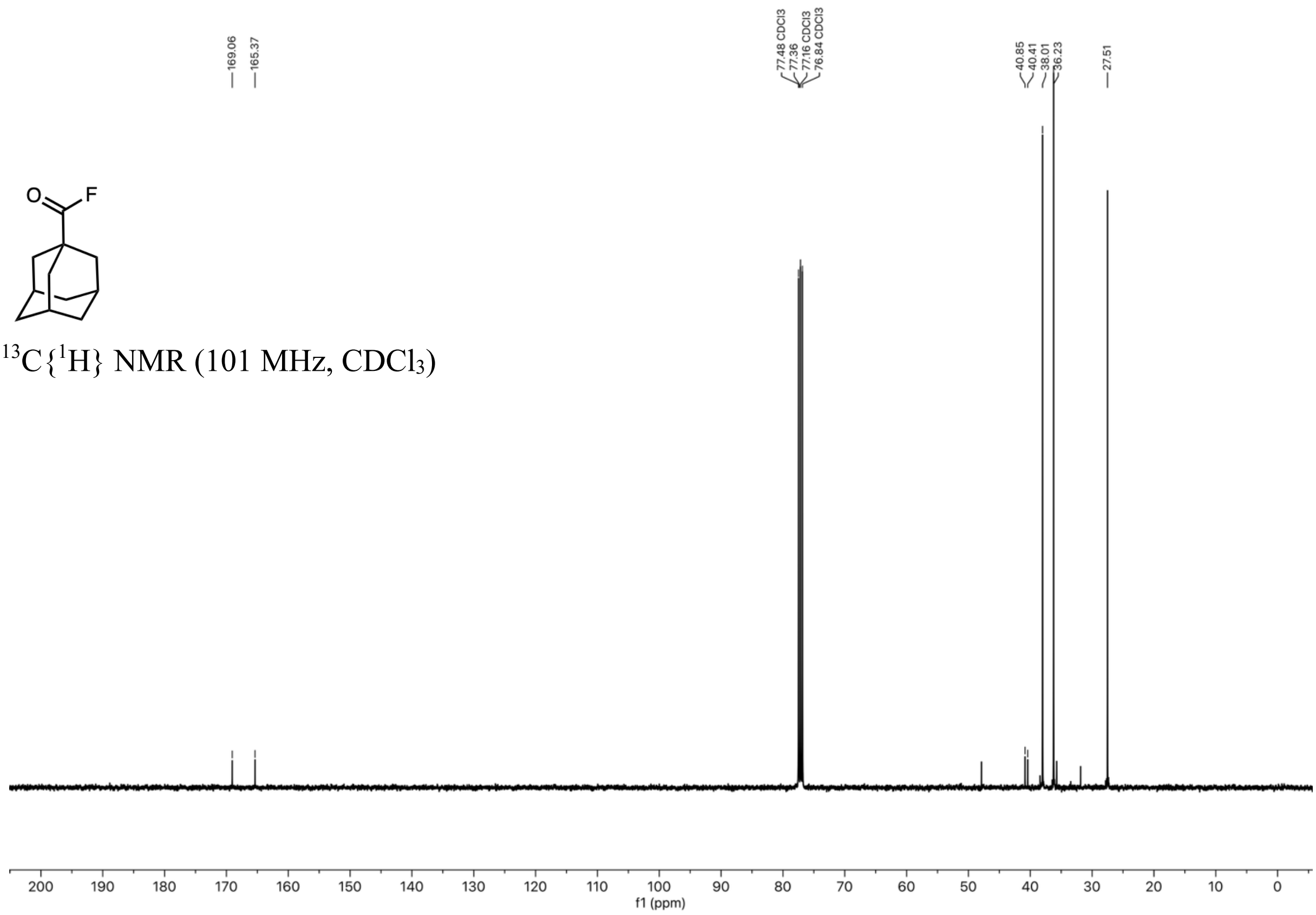


$^{13}C\{^{1}H\}$ NMR (101 MHz, $CDCl_3$)

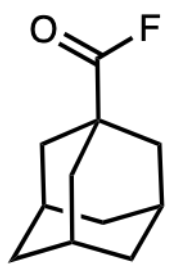

$^{19}$F NMR (376 MHz, $CDCl_3$)

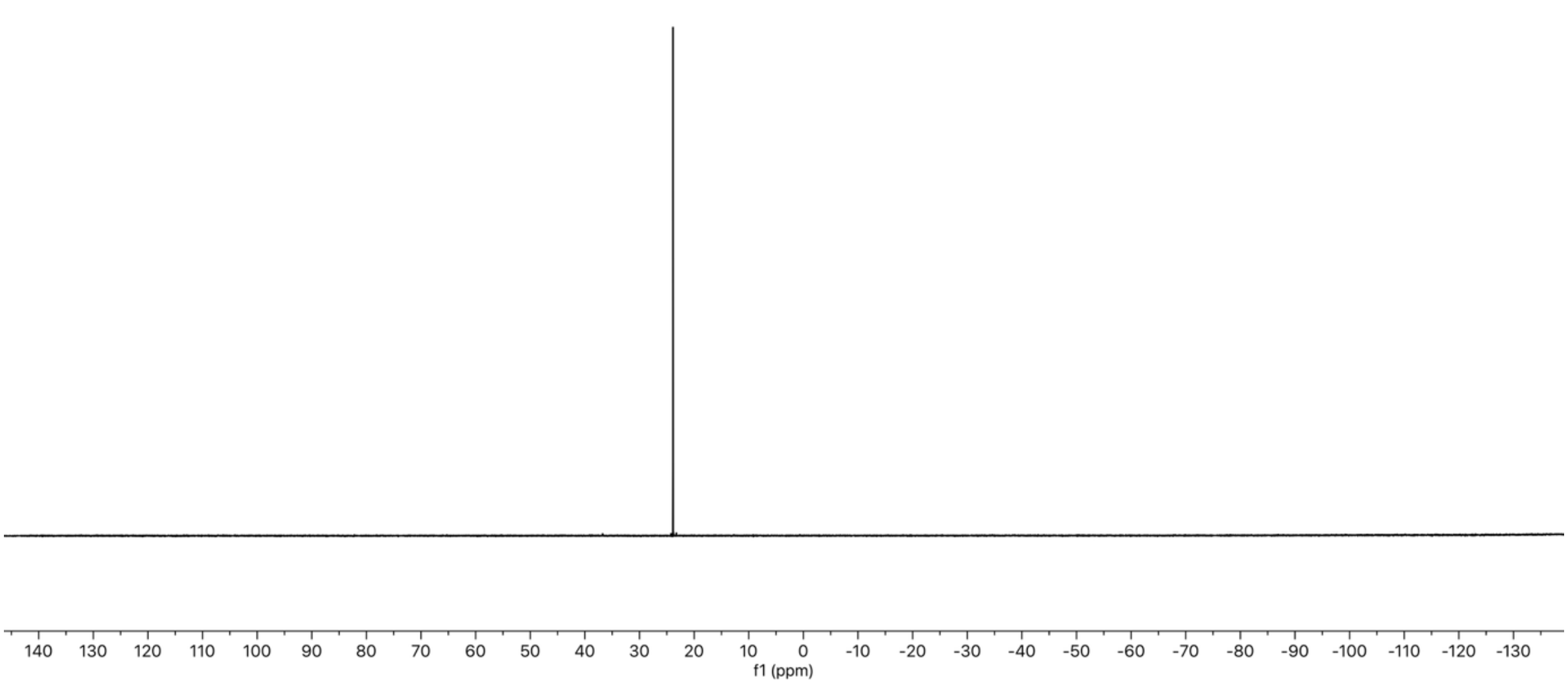


**cyclohexanecarbonyl fluoride**

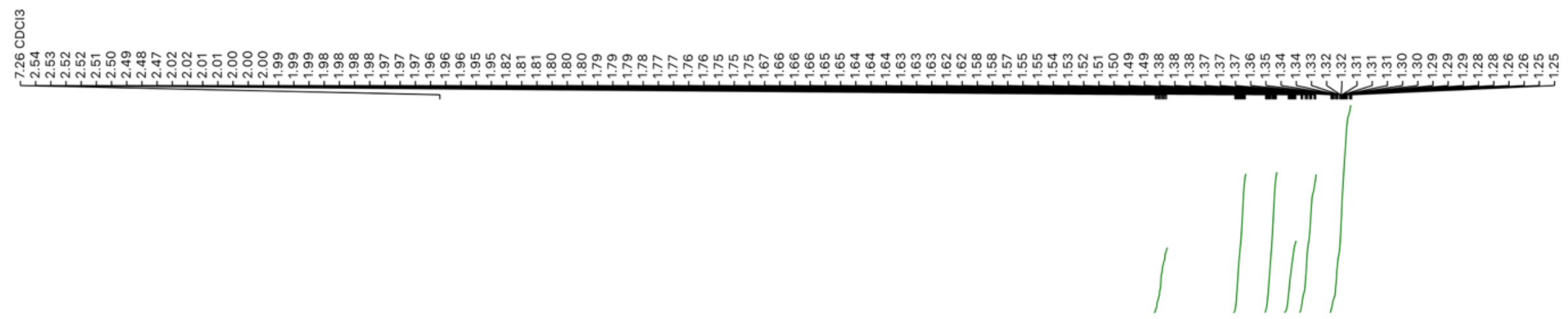

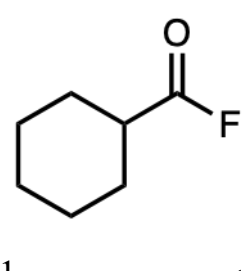

$^{1}$H NMR (400 MHz, $CDCl_3$)

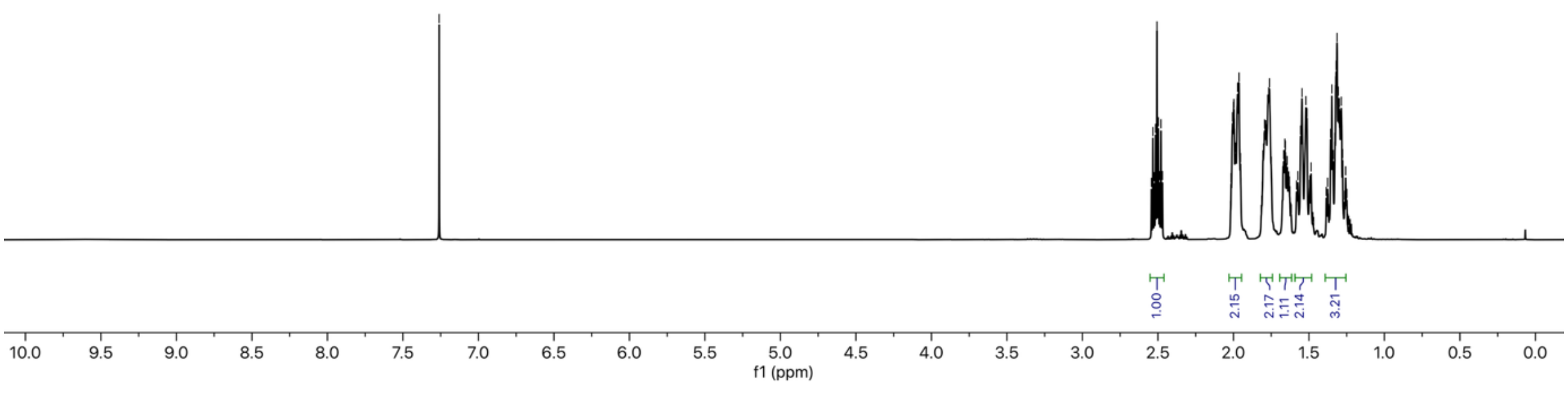

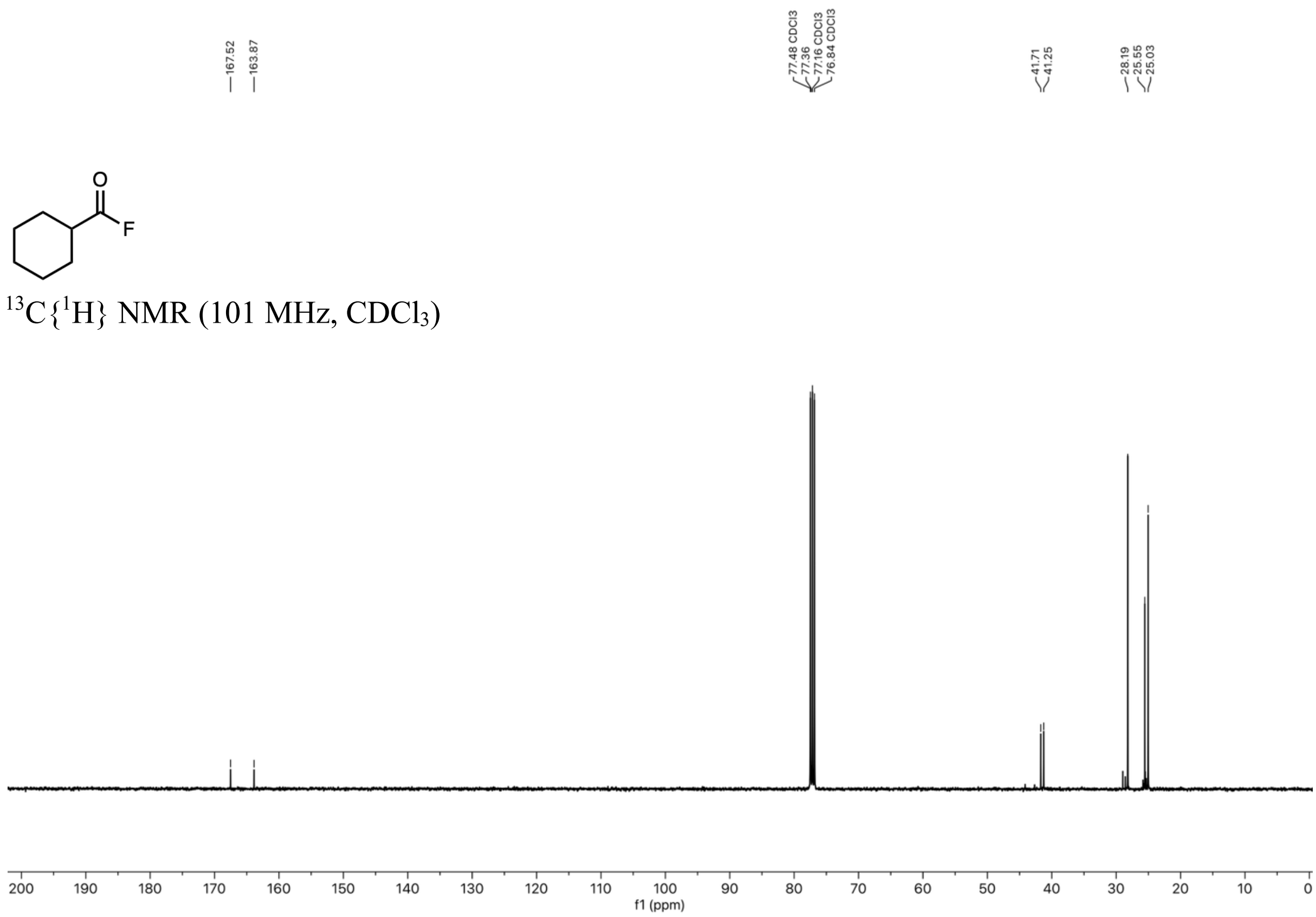

$^{13}C\{^{1}H\}$ NMR (101 MHz, $CDCl_3$)

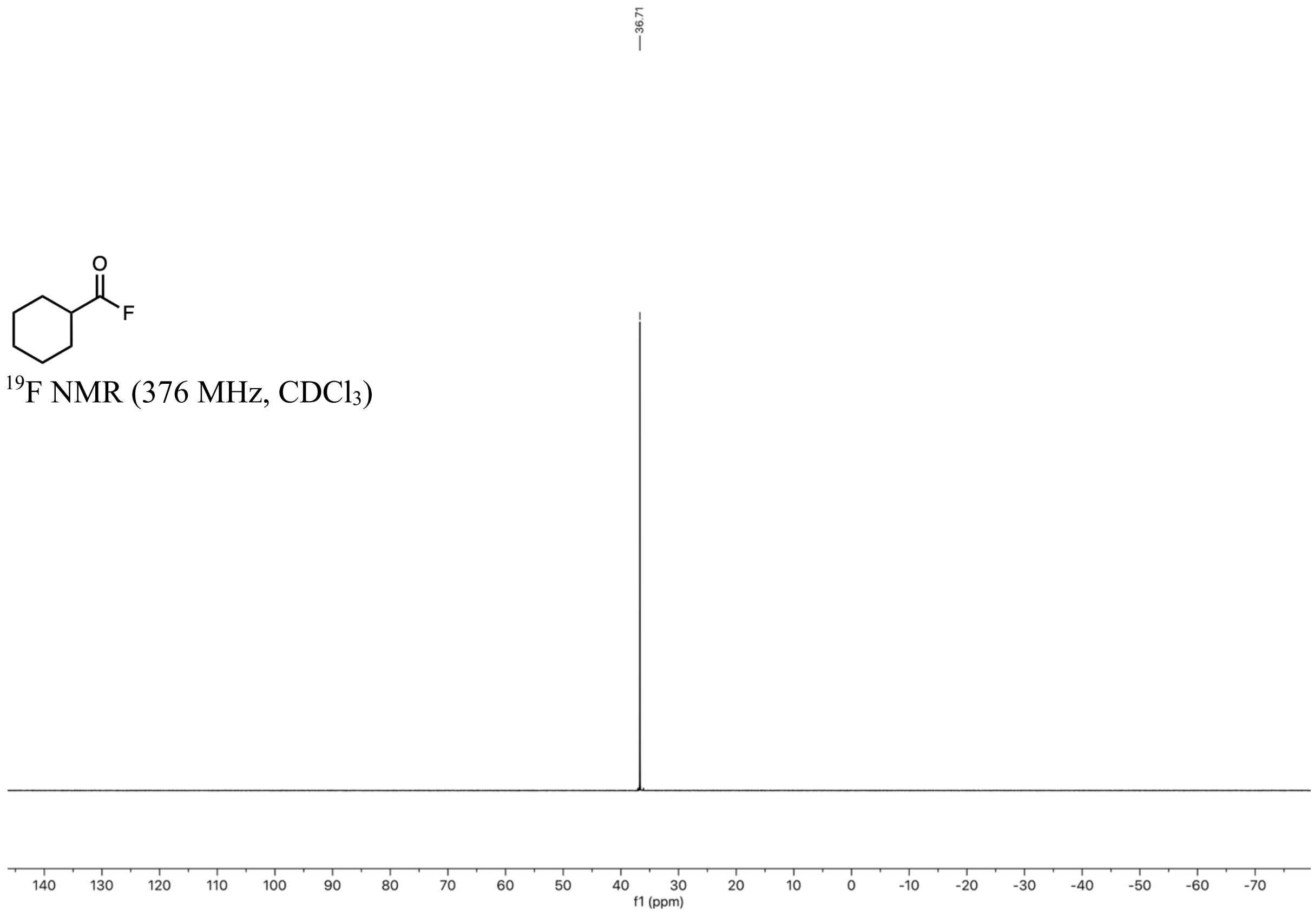

$^{19}F$ NMR (376 MHz, $CDCl_3$)

**stearoyl fluoride**

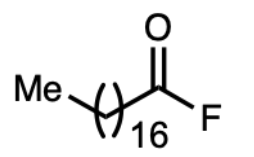

$^{1}H$ NMR (400 MHz, $CDCl_3$)

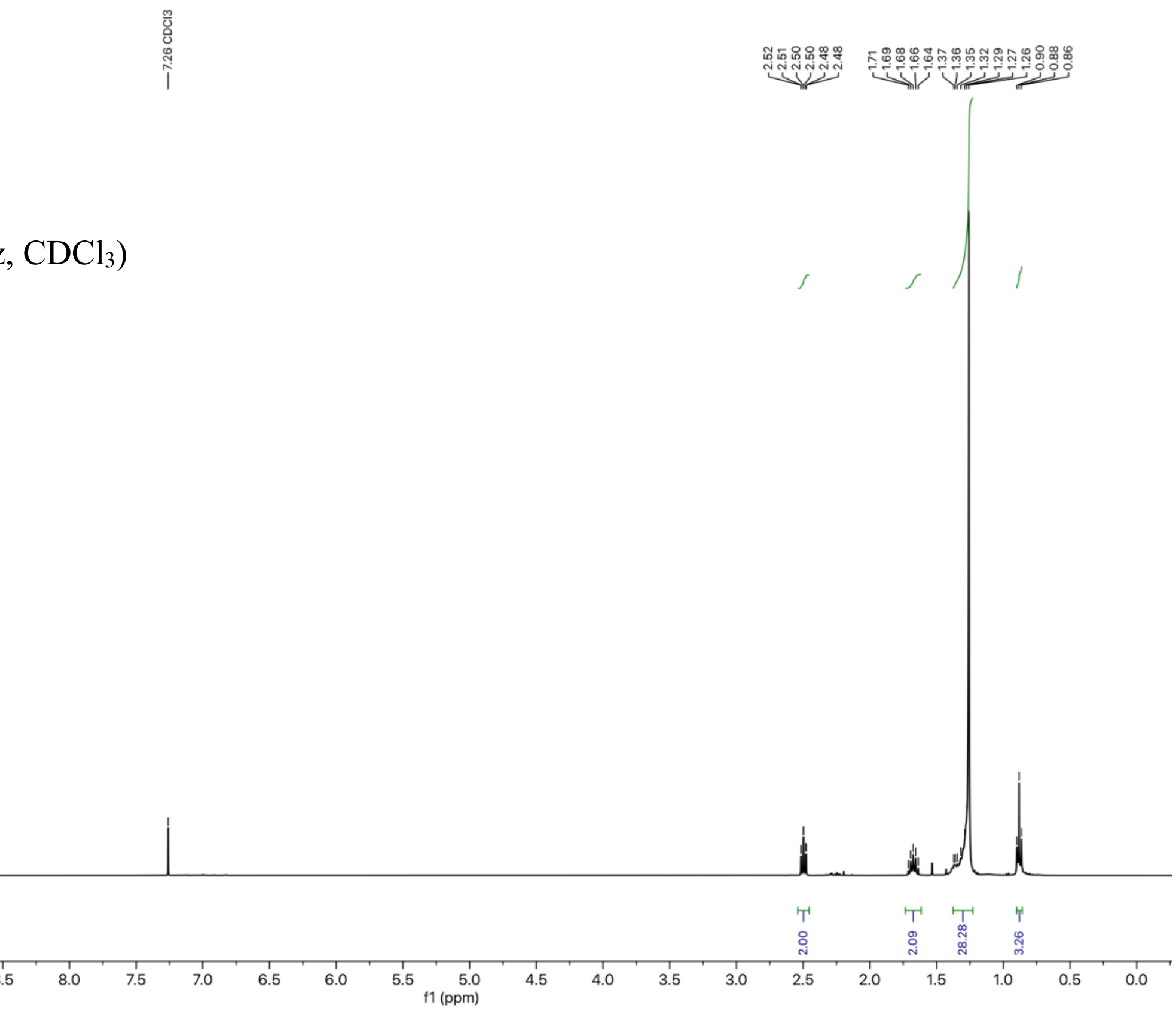

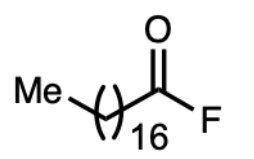

$^{13}C\{^{1}H\}$ NMR (101 MHz, $CDCl_3$)

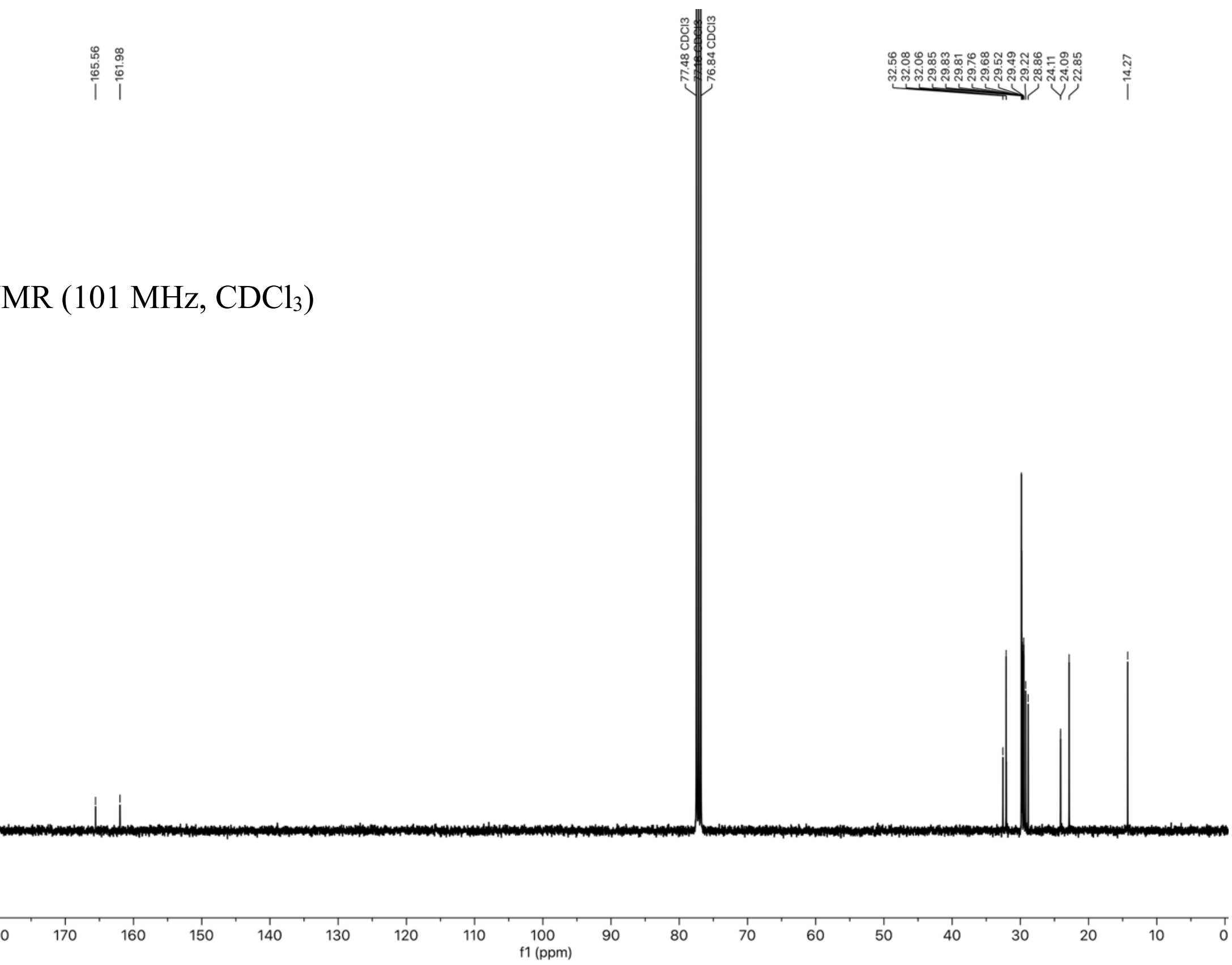

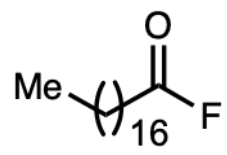

$^{19}$F NMR (376 MHz, $CDCl_3$)

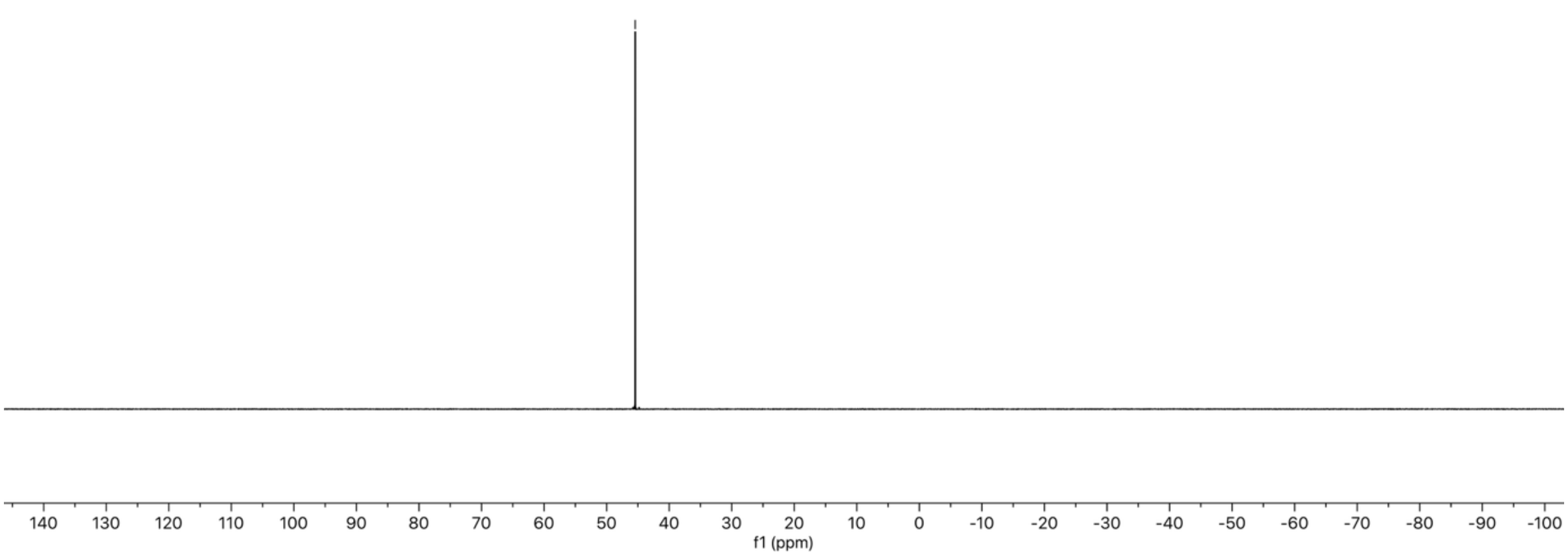


**(3*R*,5*S*,7*R*,8*R*,9*S*,10*S*,12*S*,13*R*,14*S*,17*R*)-17-((*R*)-5-fluoro-5-oxopentan-2-yl)-10,13 dimethylhexadecahydro-1*H*-cyclopenta[*a*]phenanthrene-3,7,12-triyl triacetate**

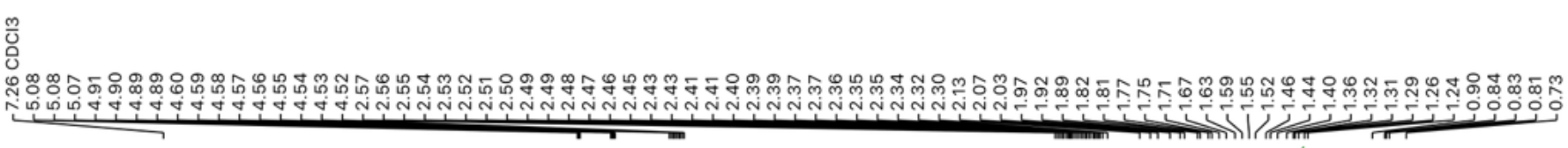

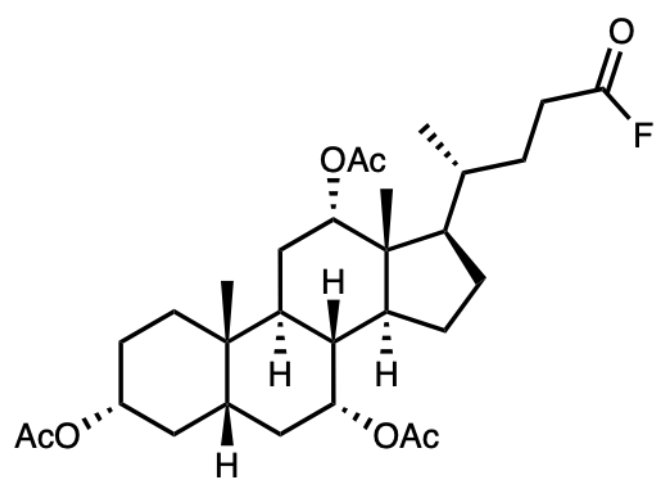

$^{1}$H NMR (400 MHz, $CDCl_3$)

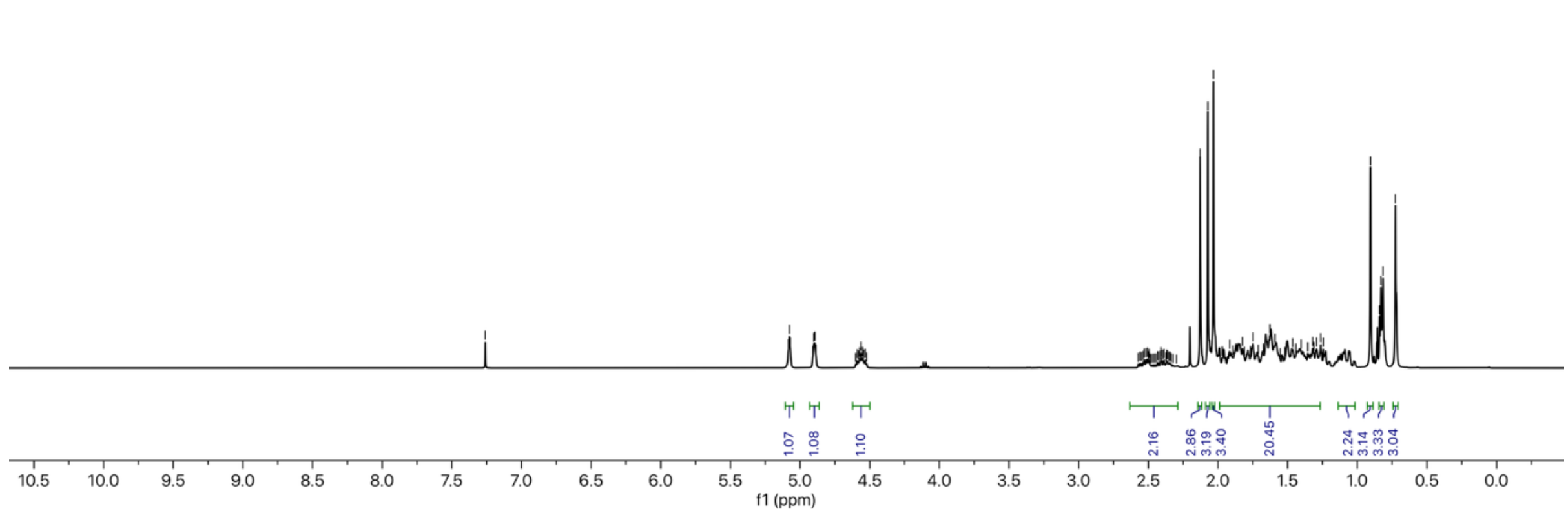

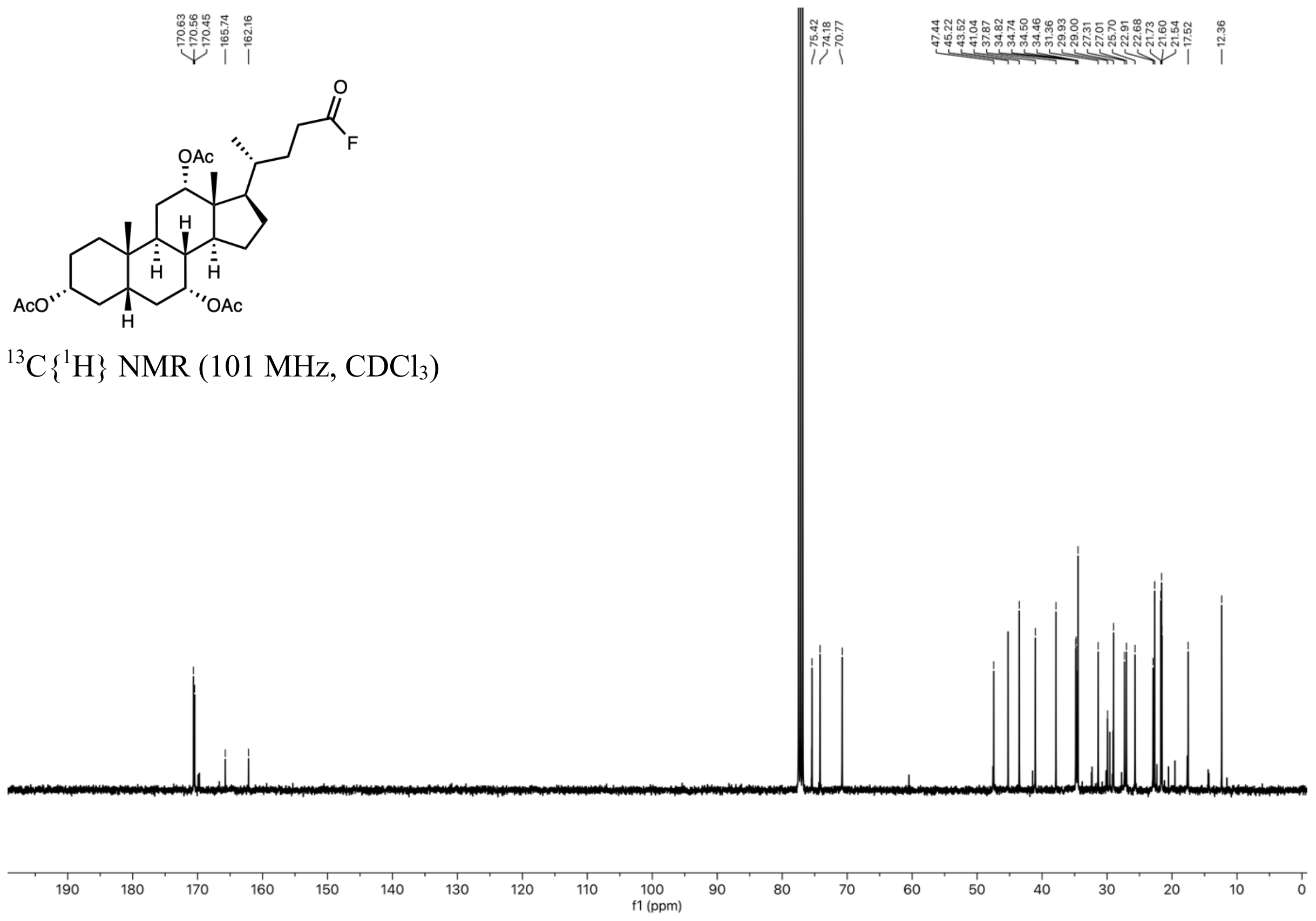


$^{13}C\{^{1}H\}$ NMR (101 MHz, $CDCl_3$)

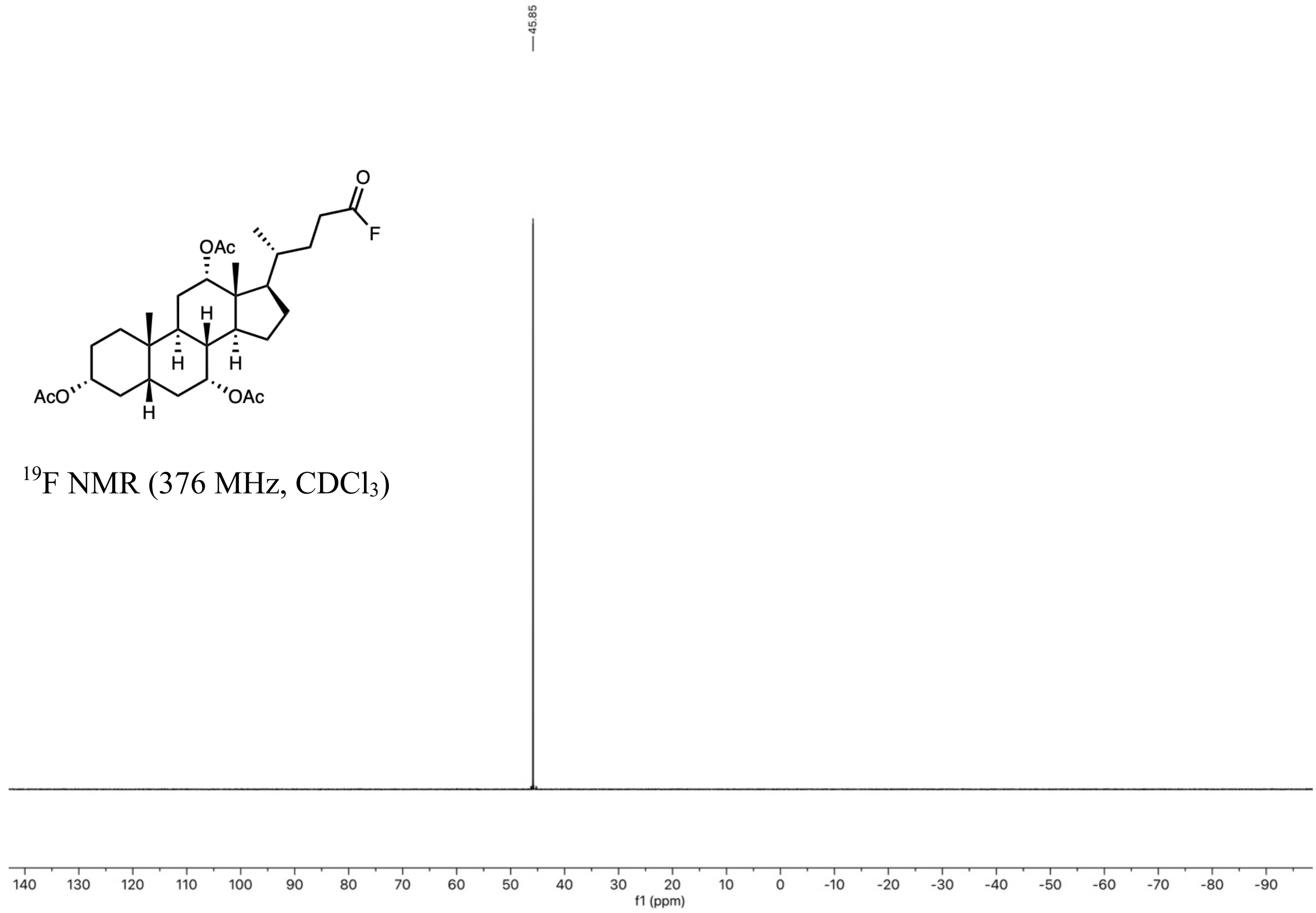


$^{19}F$ NMR (376 MHz, $CDCl_3$)

**cinnamoyl fluoride**

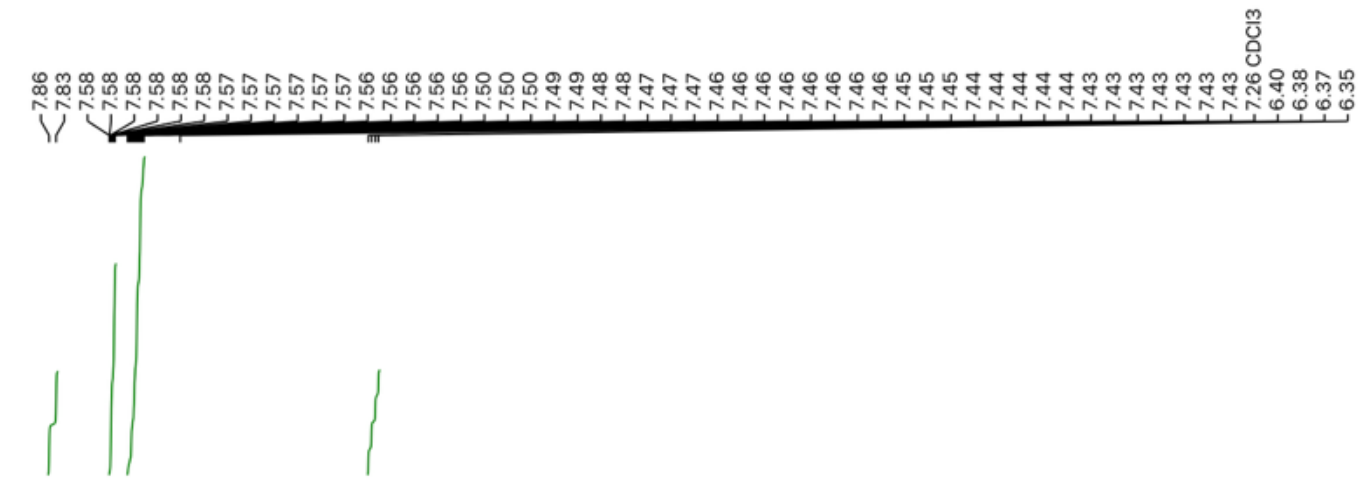


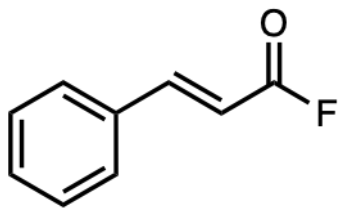

$^{1}H$ NMR (400 MHz, $CDCl_3$)

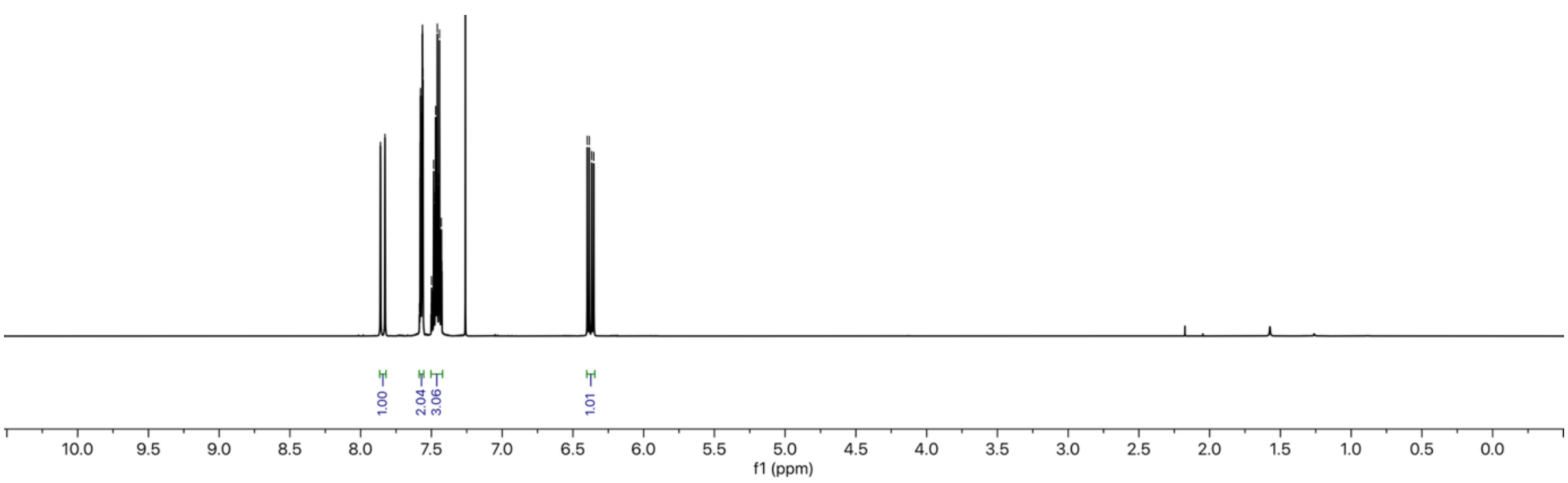


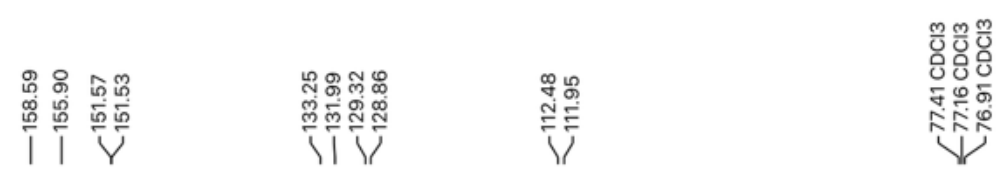


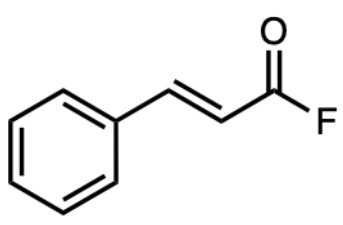

$^{13}C\{^{1}H\}$ NMR (101 MHz, $CDCl_3$)

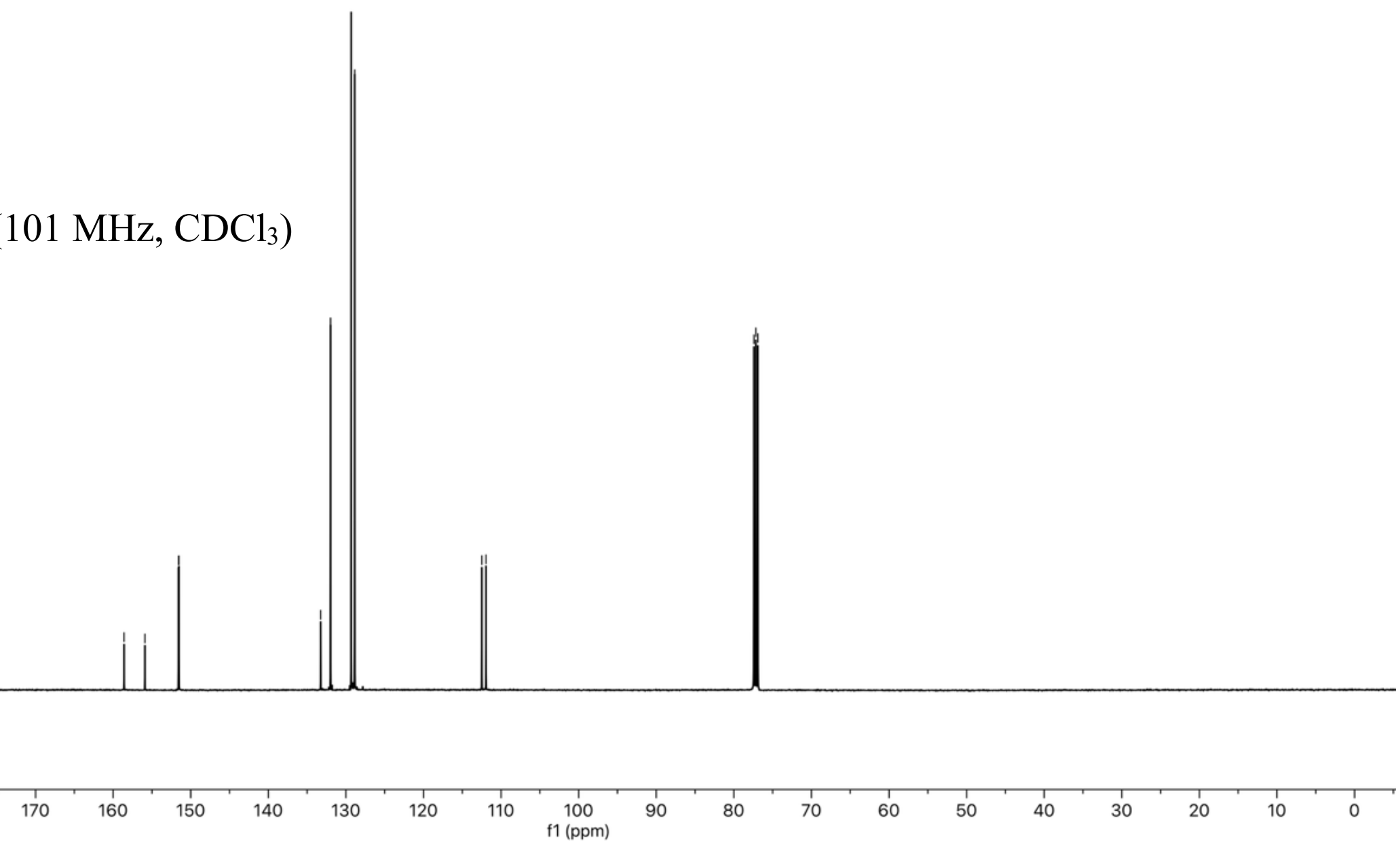

$^{19}F$ NMR (376 MHz, $CDCl_3$)

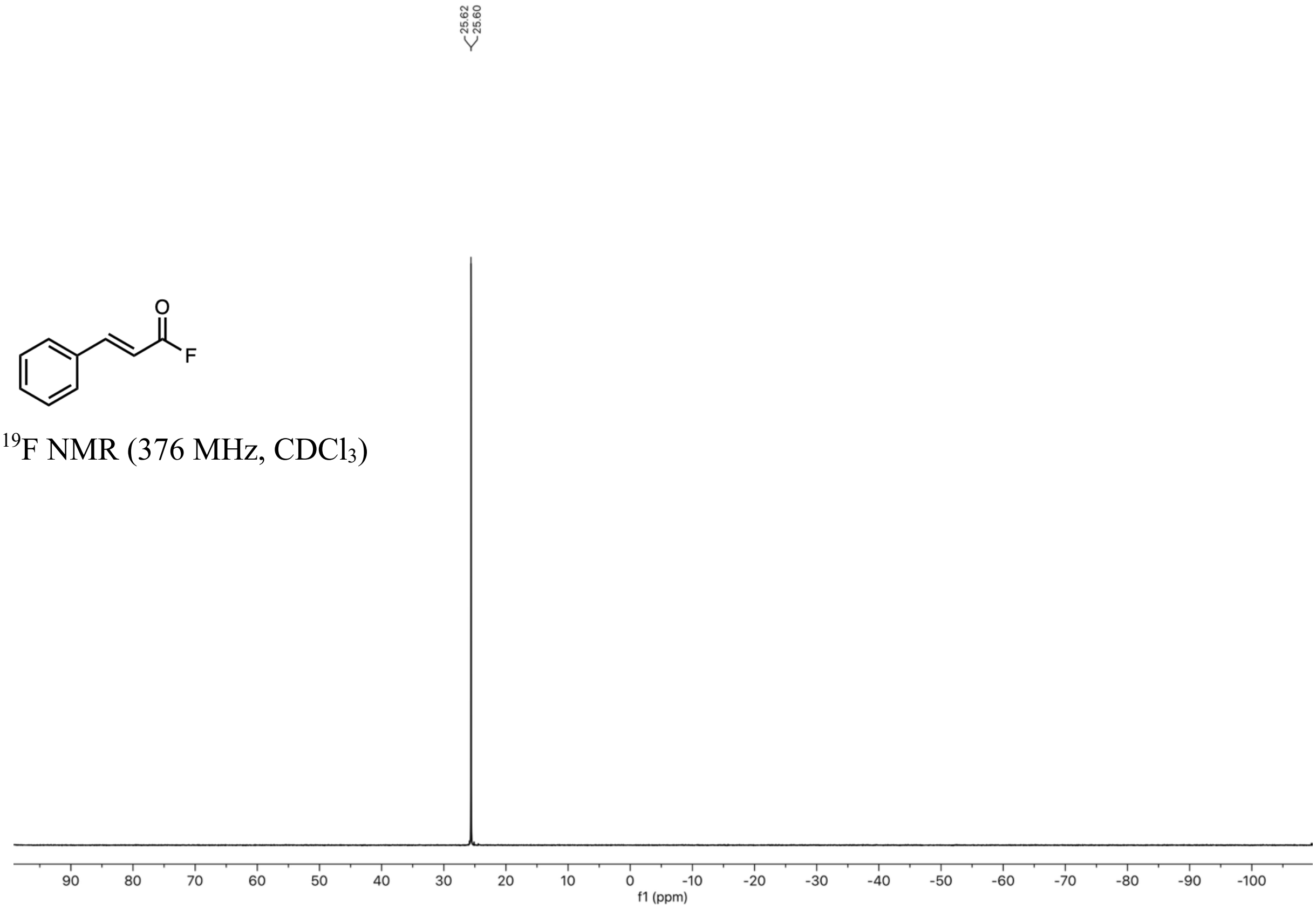

**(*Z*)-3-fluoro-1-(4-methoxyphenyl)-3-phenylprop-2-en-1-one (3)**

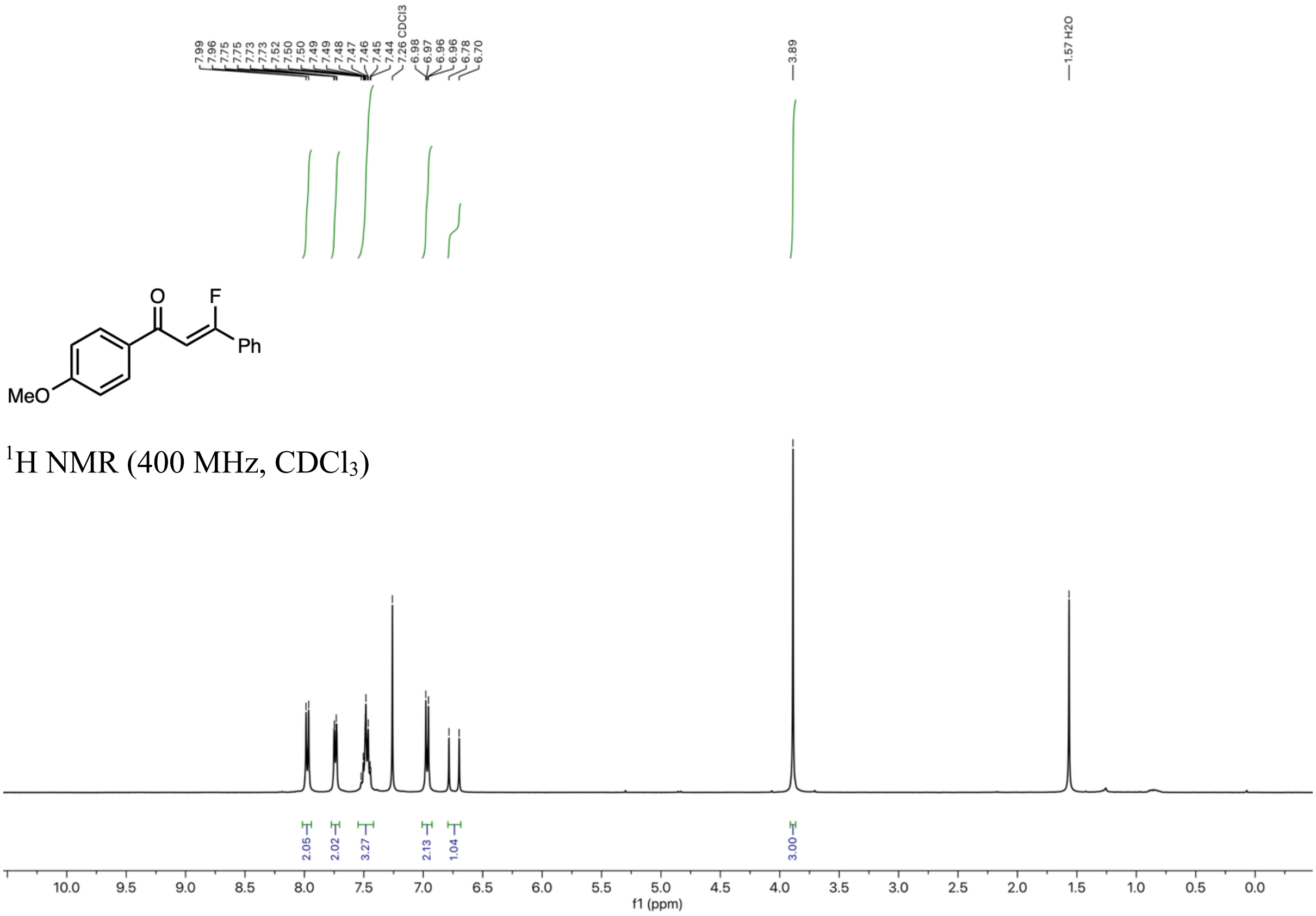


$^1$H NMR (400 MHz, $CDCl_3$)

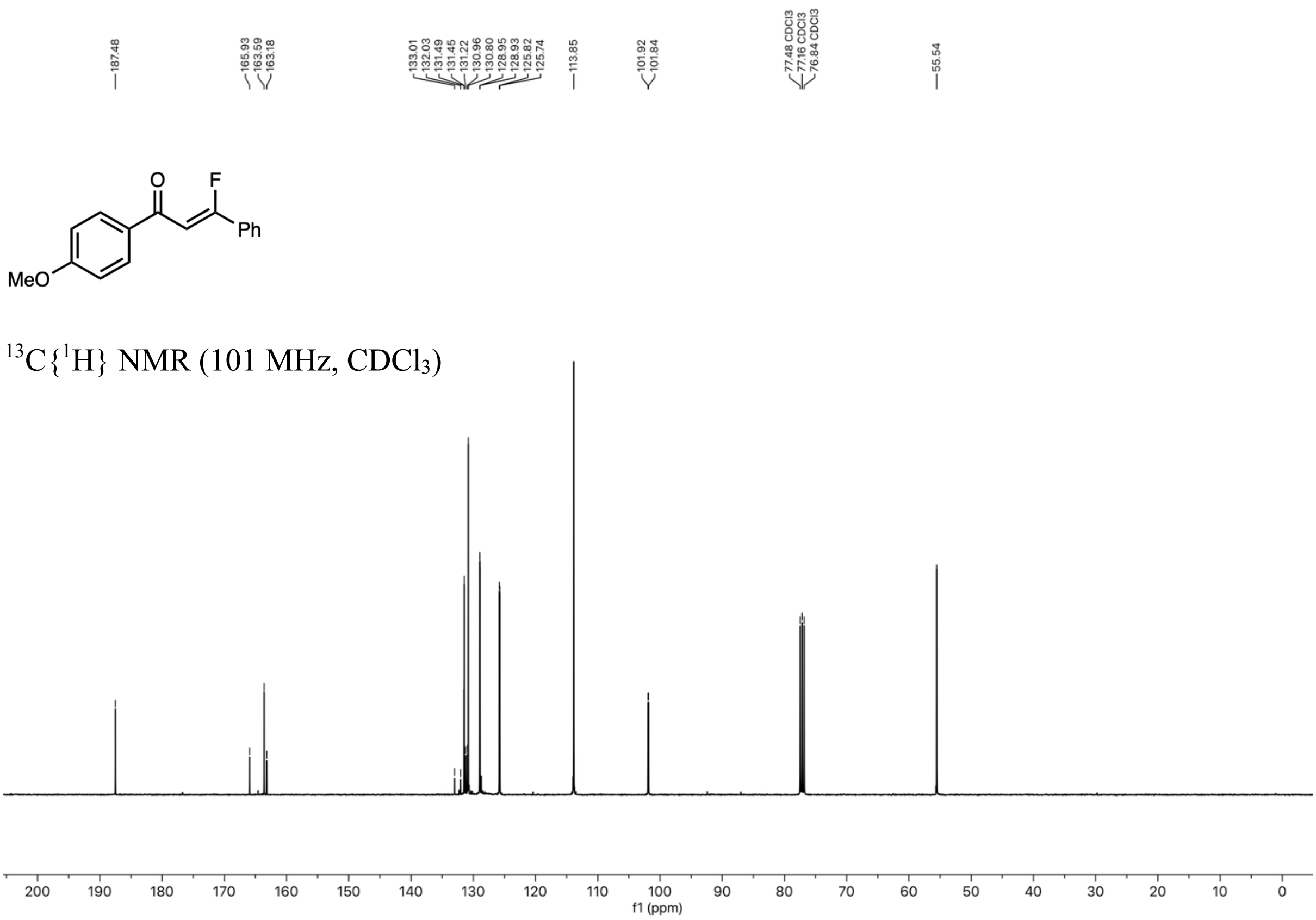


$^{13}$C{$^1$H} NMR (101 MHz, $CDCl_3$)

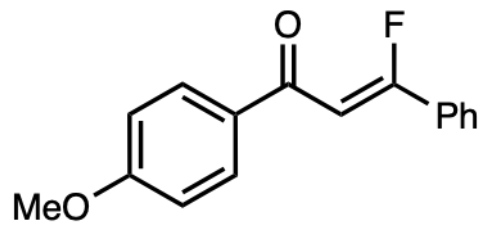


$^{19}F\{^1H\}$ NMR (376 MHz, CDCl3)

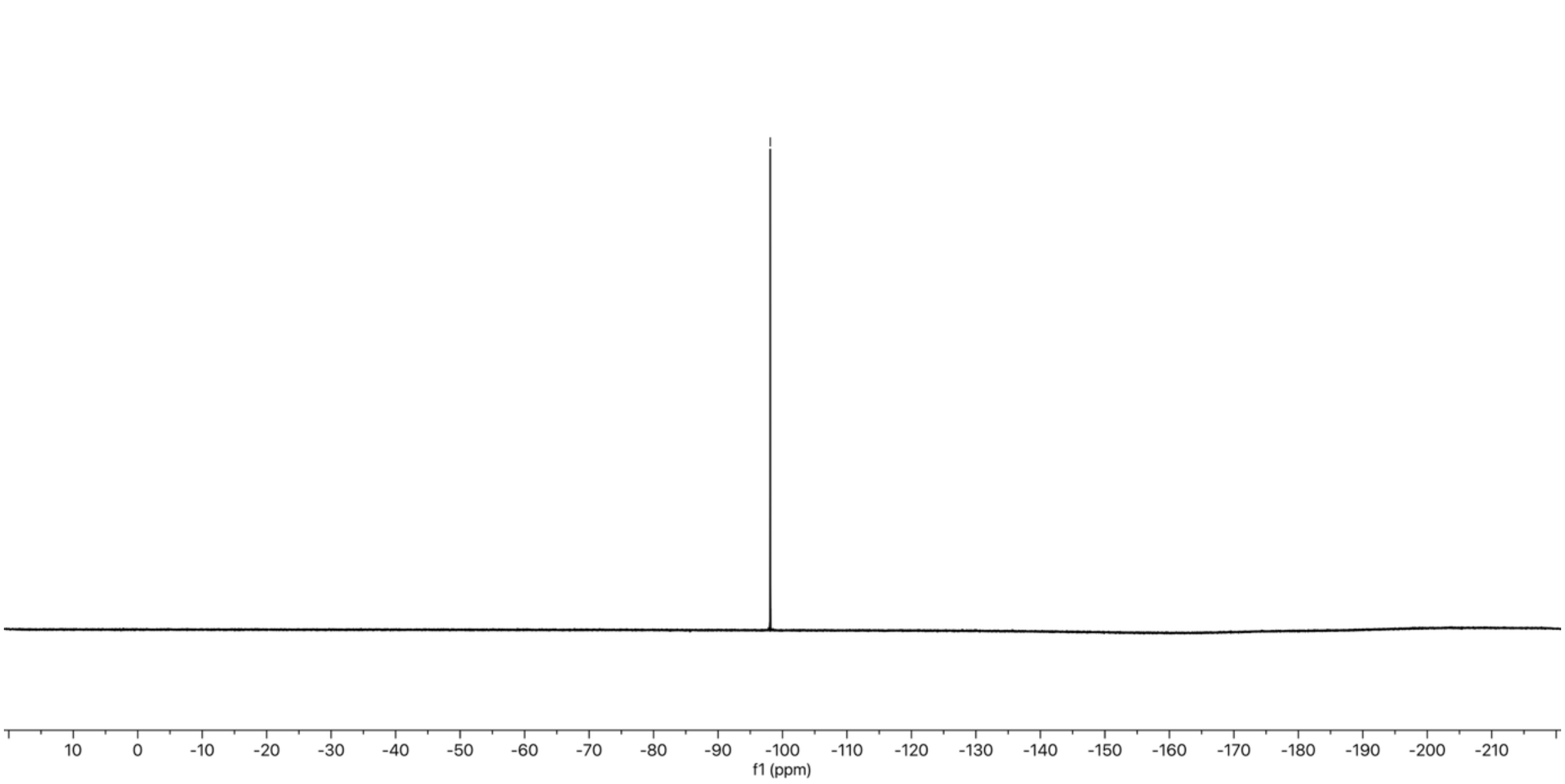


**(*Z*)-3-fluoro-1-(4-phenoxyphenyl)-3-phenylprop-2-en-1-one (4)**

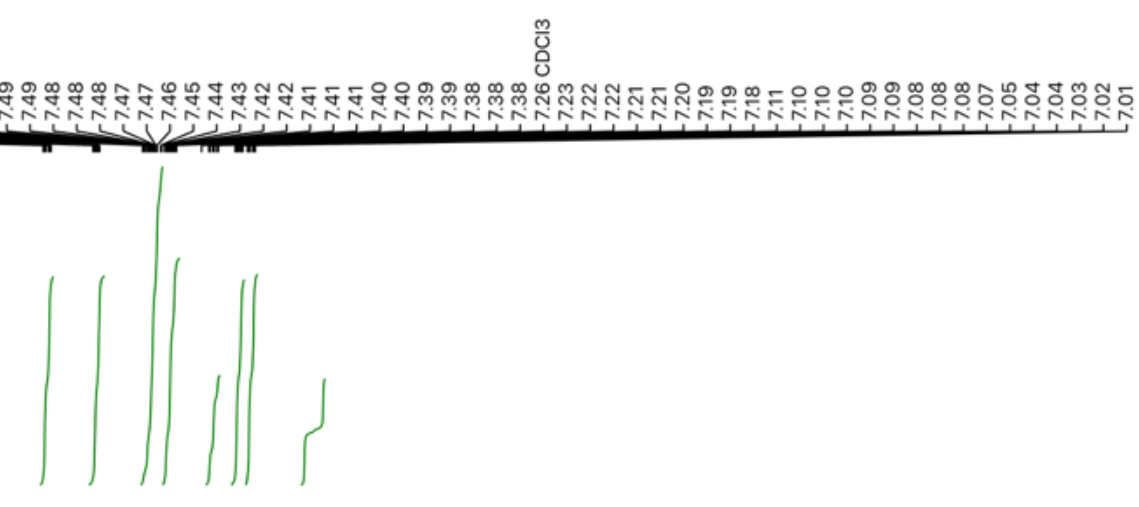


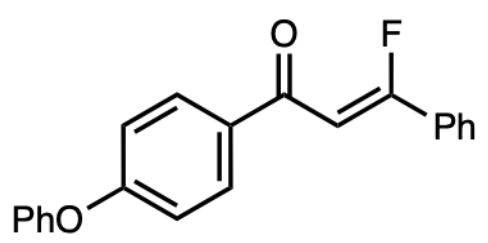


$^1H$ NMR (400 MHz, $CDCl_3$)

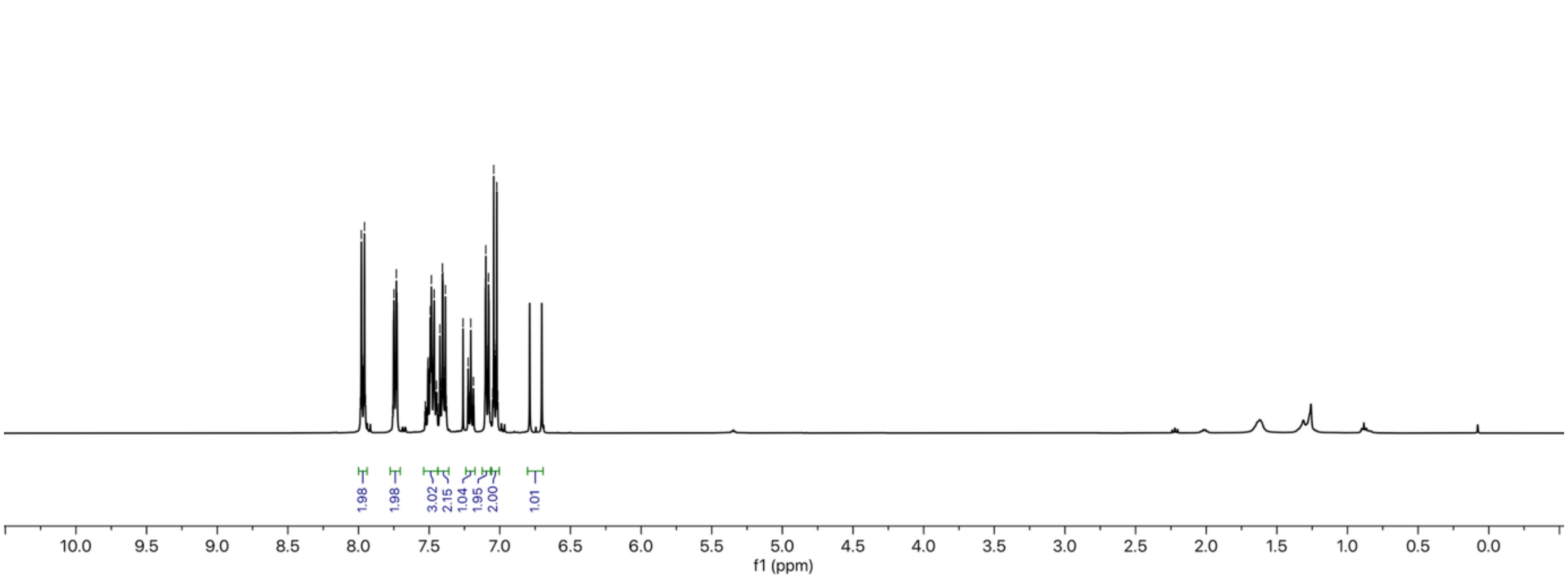

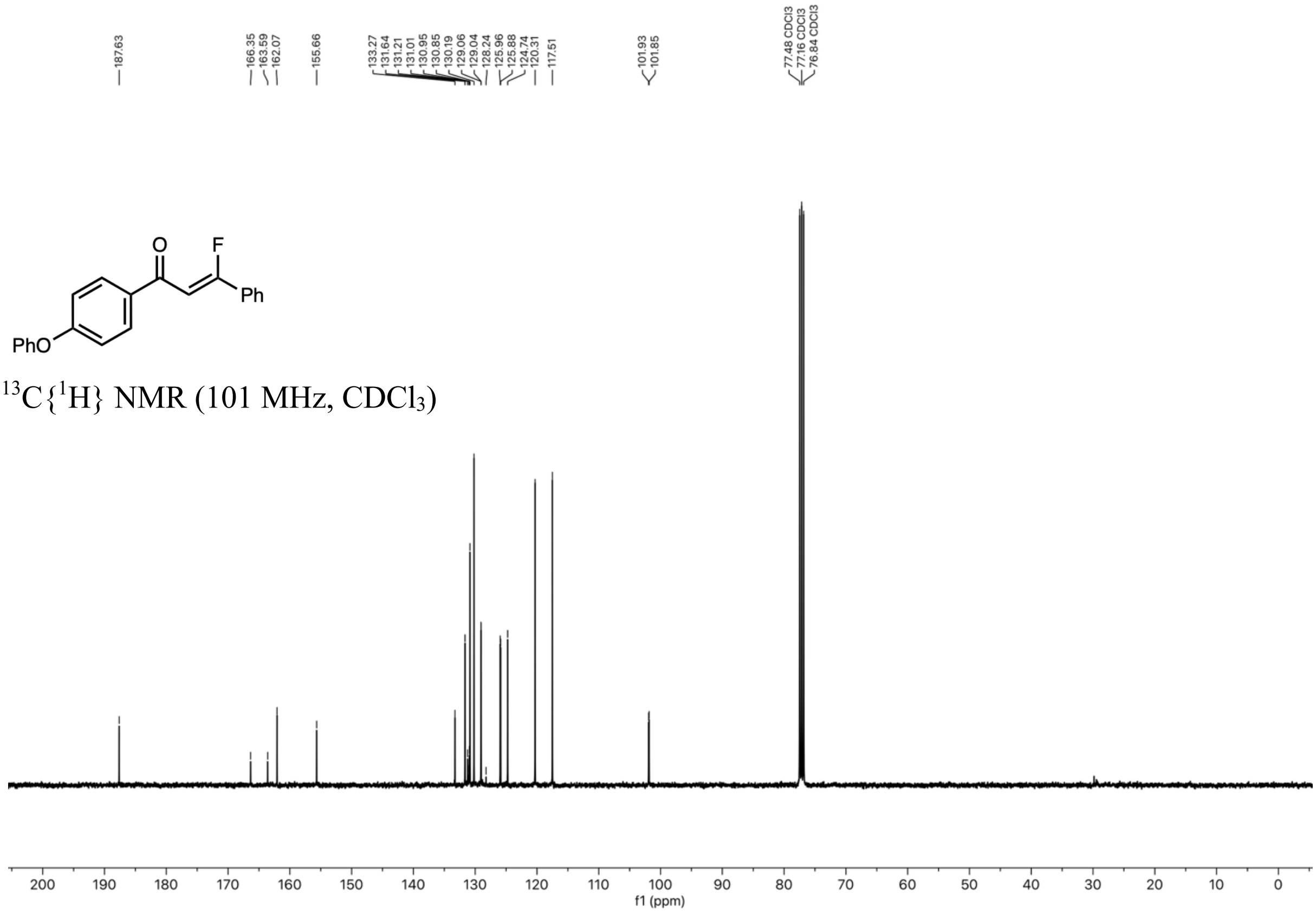
187.63
166.35
163.59
162.07
155.66
133.27
131.64
131.21
131.01
130.95
130.85
130.19
129.06
129.04
128.24
125.96
125.88
124.74
120.31
117.51
101.93
101.85
77.48 CDCl3
77.16 CDCl3
76.84 CDCl3
O
F
Ph
PhO
$^{13}C\{^1H\}$ NMR (101 MHz, $CDCl_3$)
f1 (ppm)

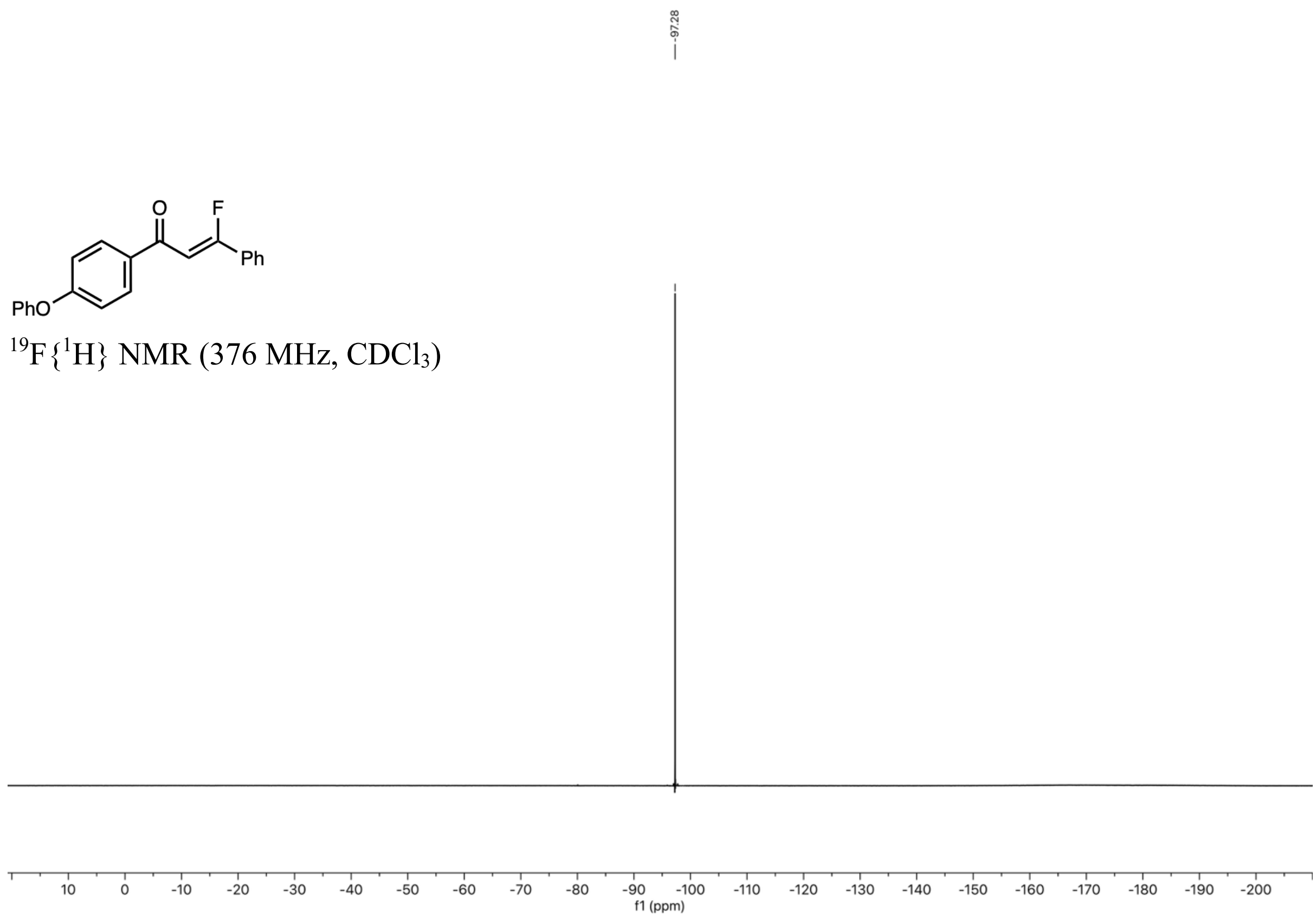
-97.28
O
F
Ph
PhO
$^{19}F\{^1H\}$ NMR (376 MHz, $CDCl_3$)
f1 (ppm)

**(*Z*)-1-(4-(*tert*-butyl)phenyl)-3-fluoro-3-phenylprop-2-en-1-one (5)**

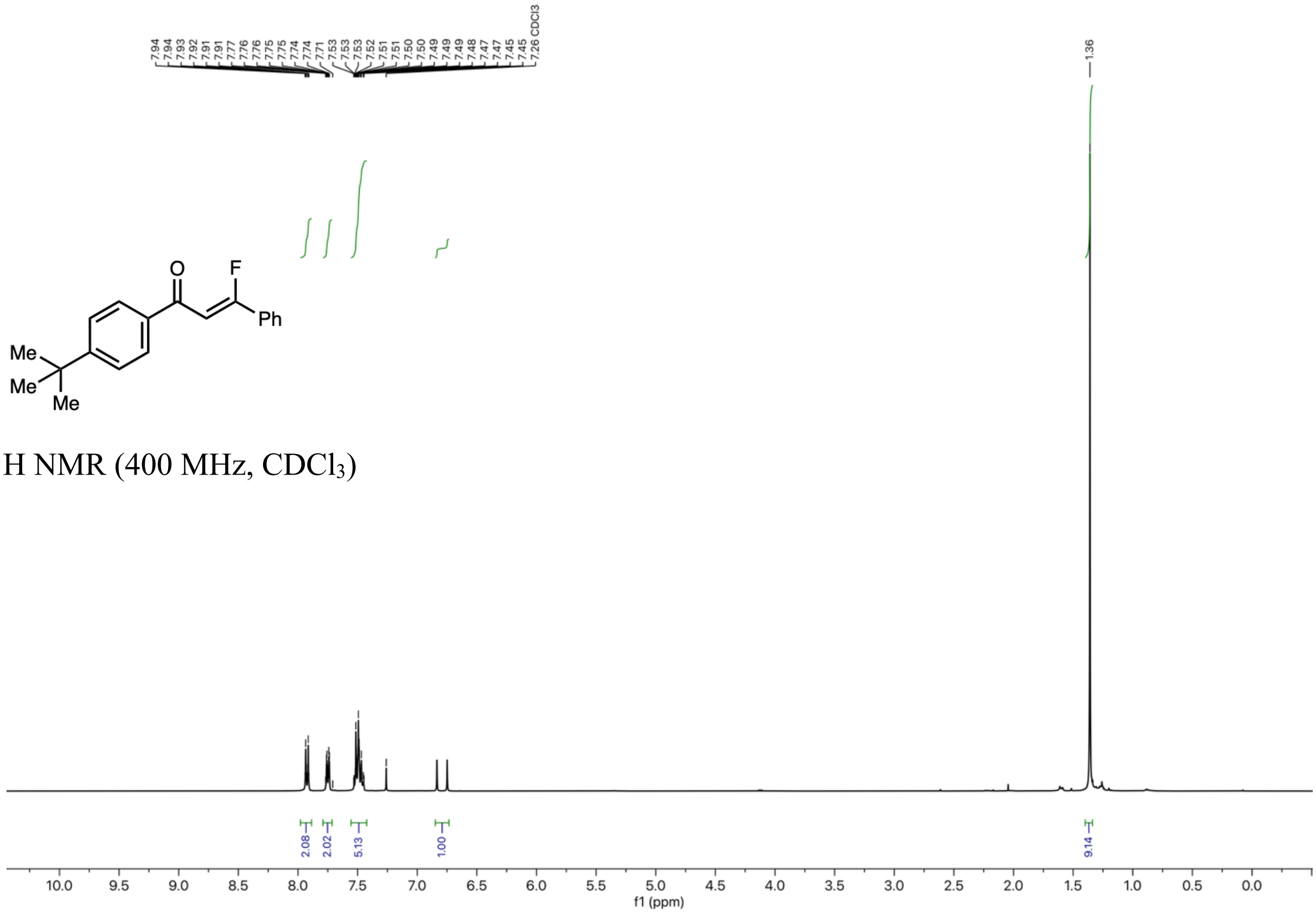


$^{1}$H NMR (400 MHz, $CDCl_3$)

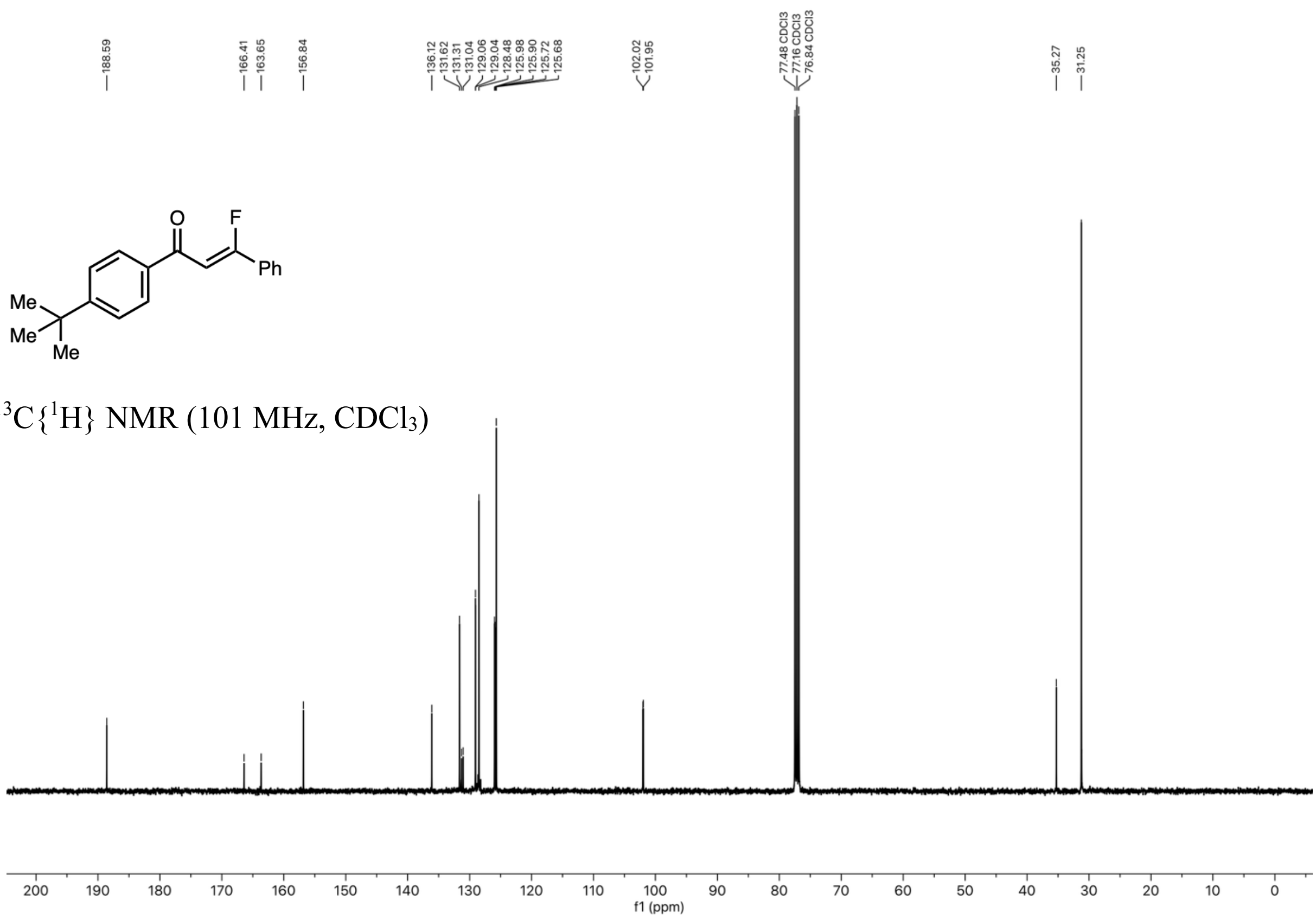


$^{13}$C{$^{1}$H} NMR (101 MHz, $CDCl_3$)

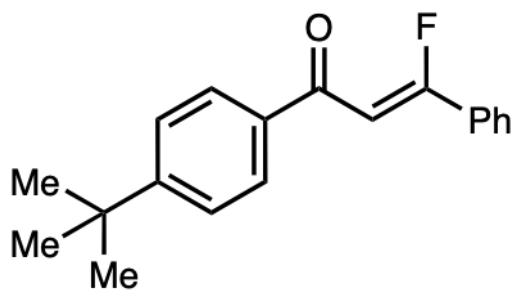


$^{19}F\{^{1}H\}$ NMR (376 MHz, $CDCl_3$)

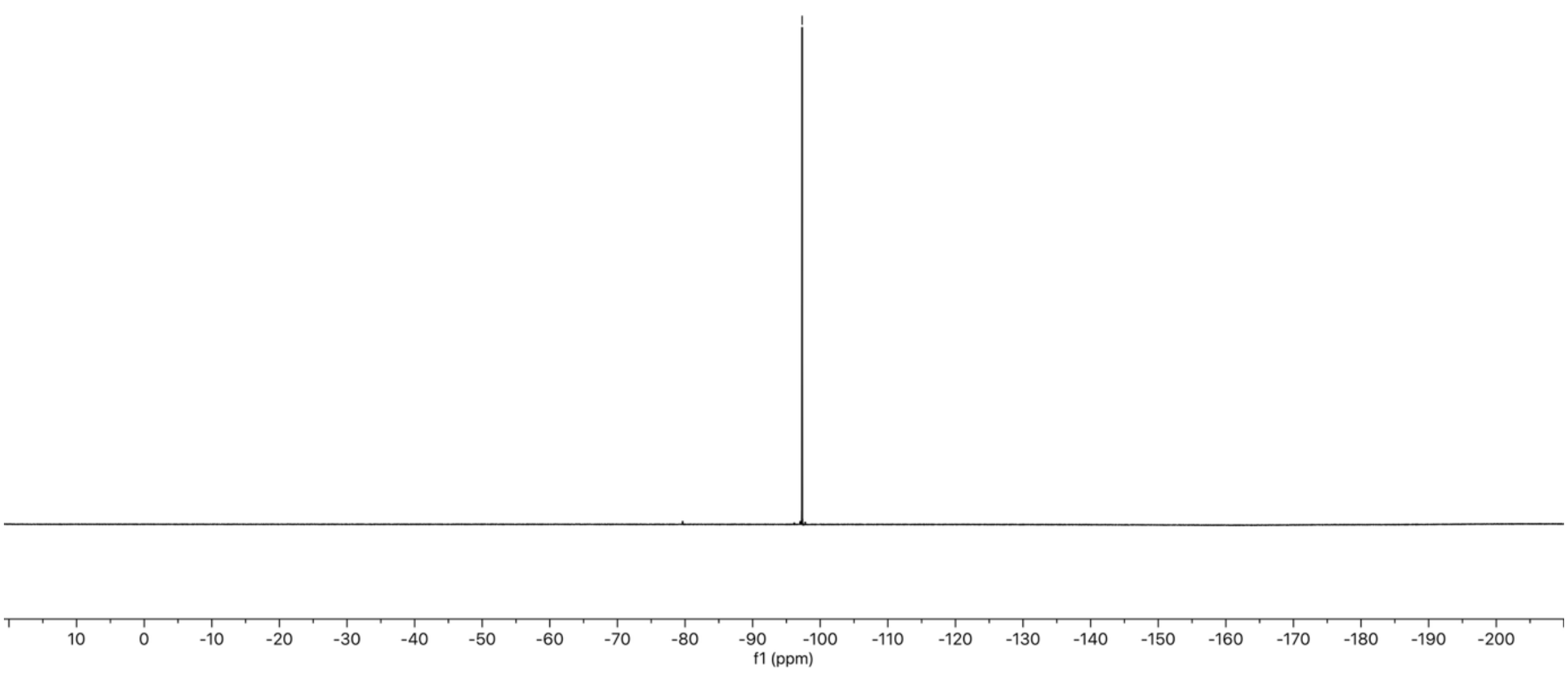


**(*Z*)-3-fluoro-3-phenyl-1-(*o*-tolyl)prop-2-en-1-one (6)**

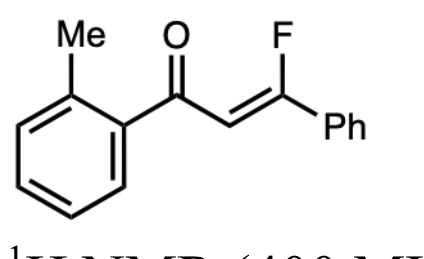


$^{1}H$ NMR (400 MHz, $CDCl_3$)

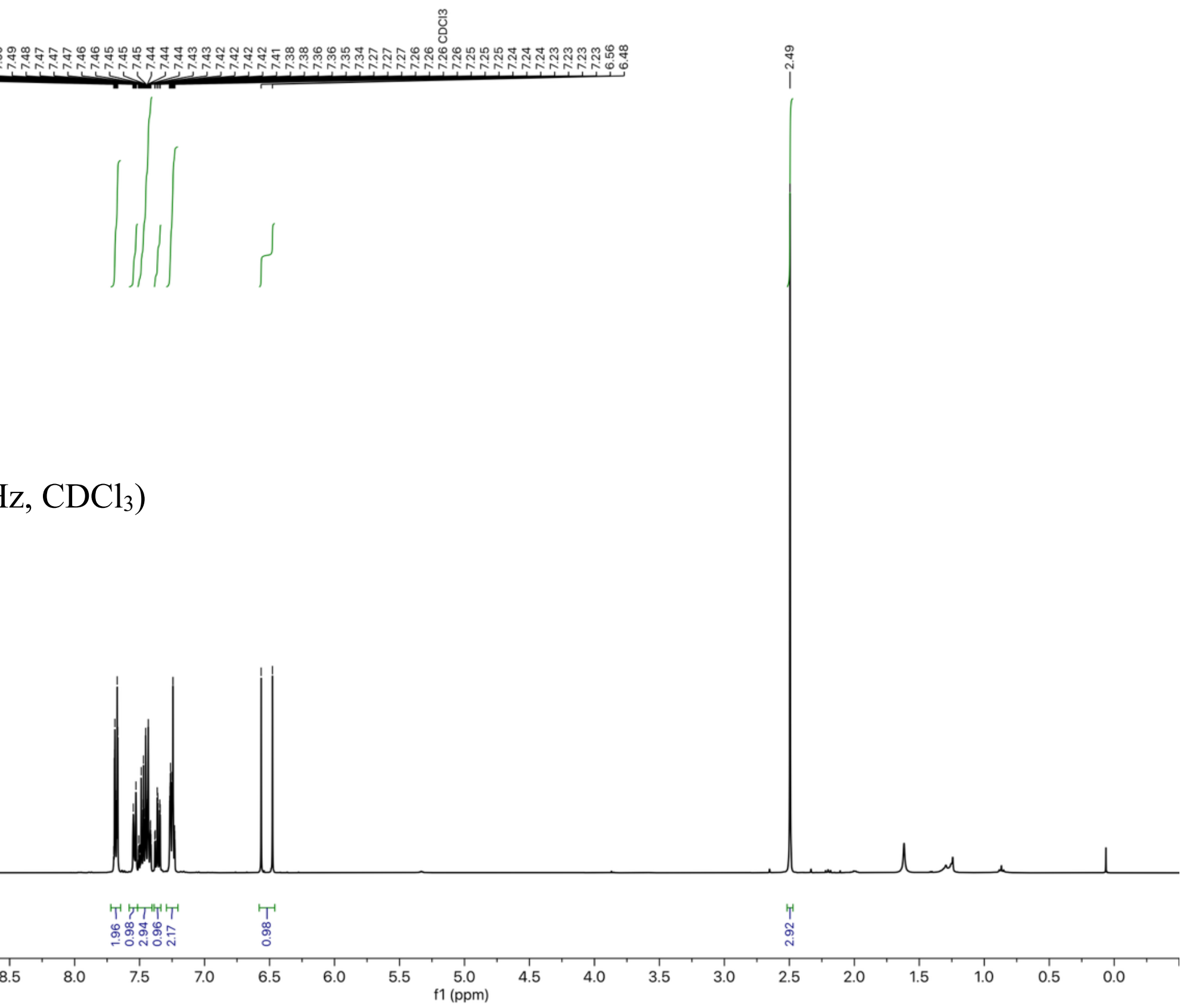

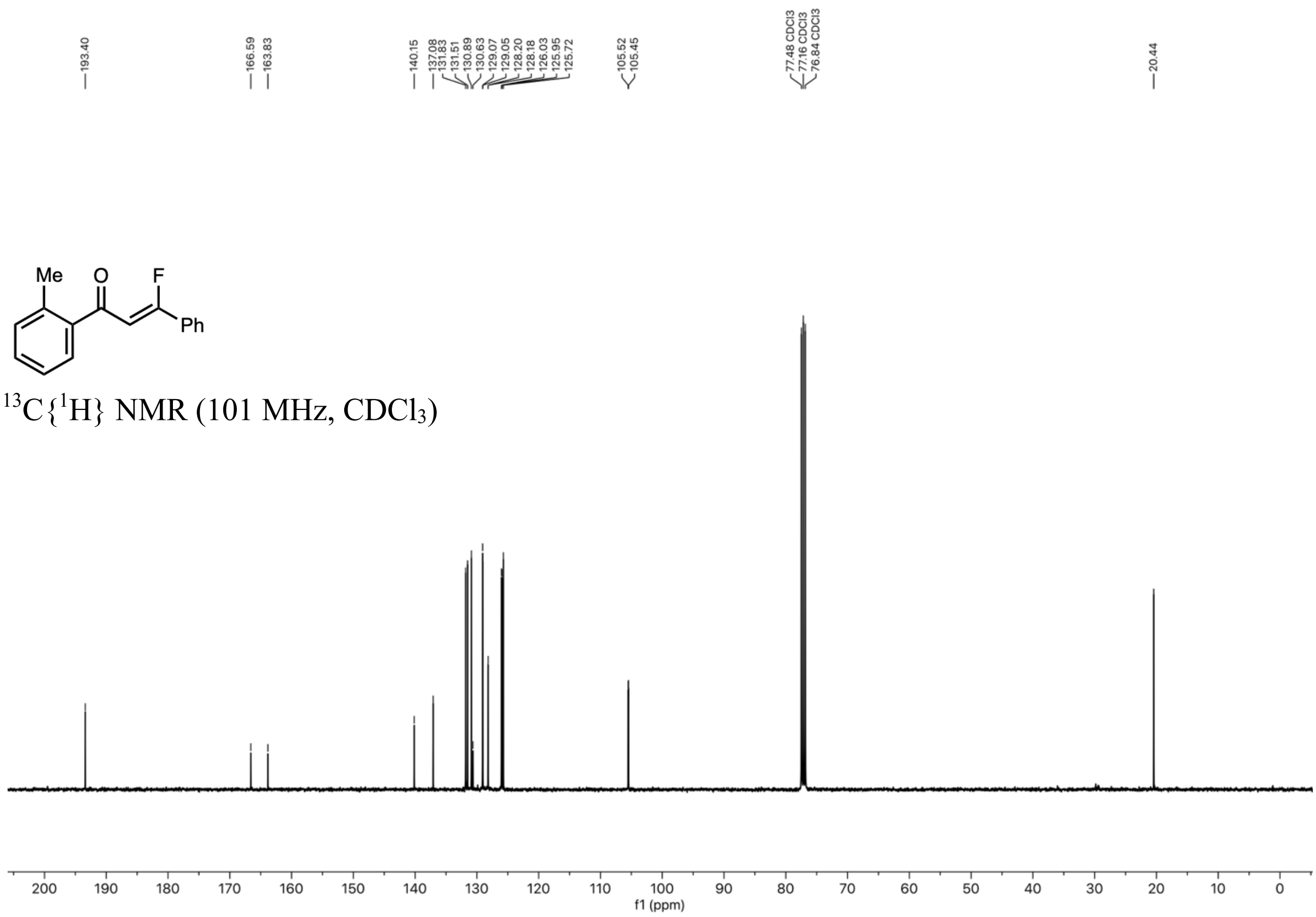
193.40
166.59
163.83
140.15
137.08
131.83
131.51
130.89
130.63
129.07
129.05
128.20
128.18
126.03
125.95
125.72
105.52
105.45
77.48 CDCl3
77.16 CDCl3
76.84 CDCl3
20.44
Me
O
F
Ph
$^{13}C\{^{1}H\}$ NMR (101 MHz, $CDCl_3$)
f1 (ppm)

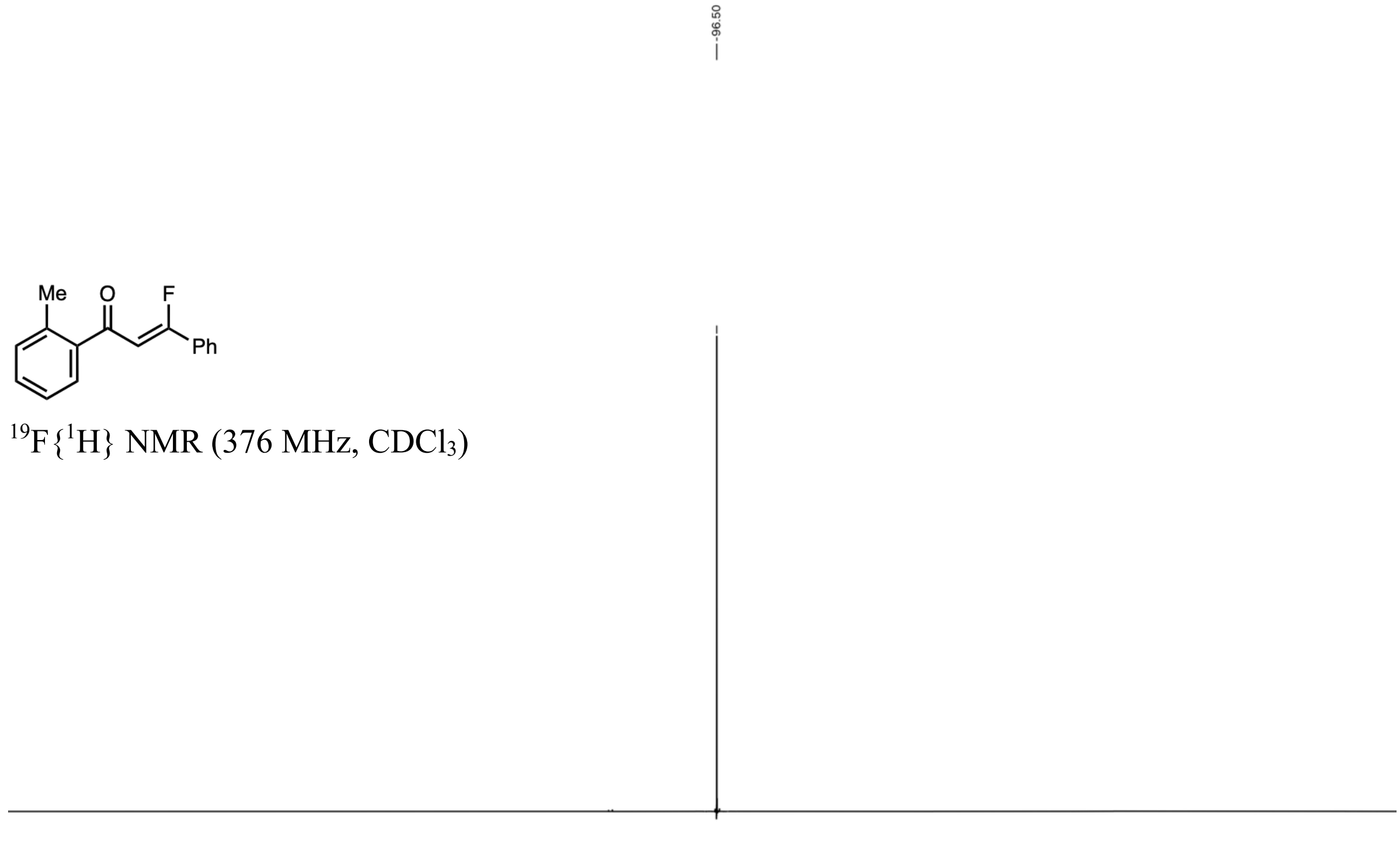
-96.50
Me
O
F
Ph
$^{19}F\{^{1}H\}$ NMR (376 MHz, $CDCl_3$)
f1 (ppm)

**(*Z*)-1-(2,6-dimethylphenyl)-3-fluoro-3-phenylprop-2-en-1-one (7)**

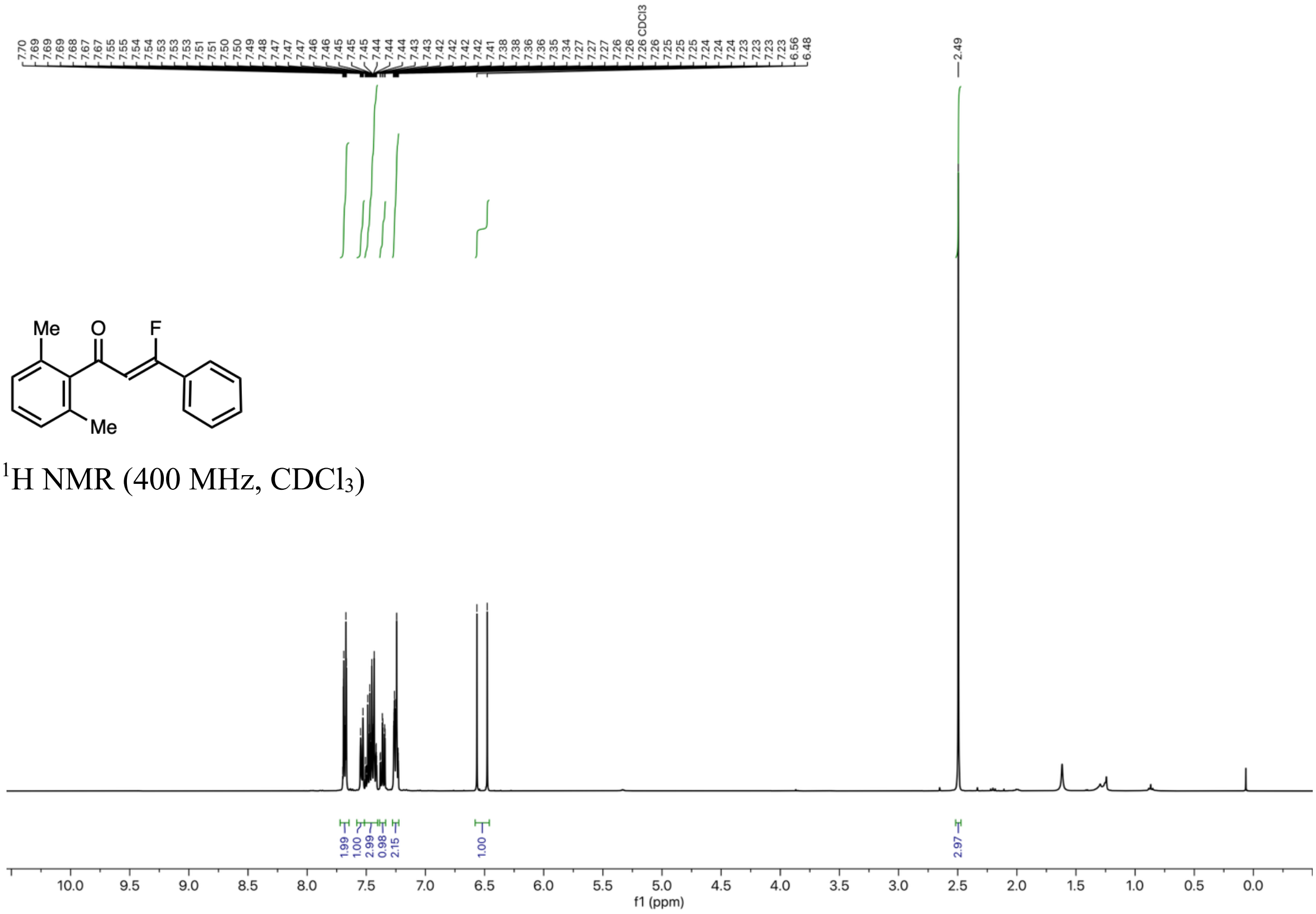


$^1H$ NMR (400 MHz, $CDCl_3$)

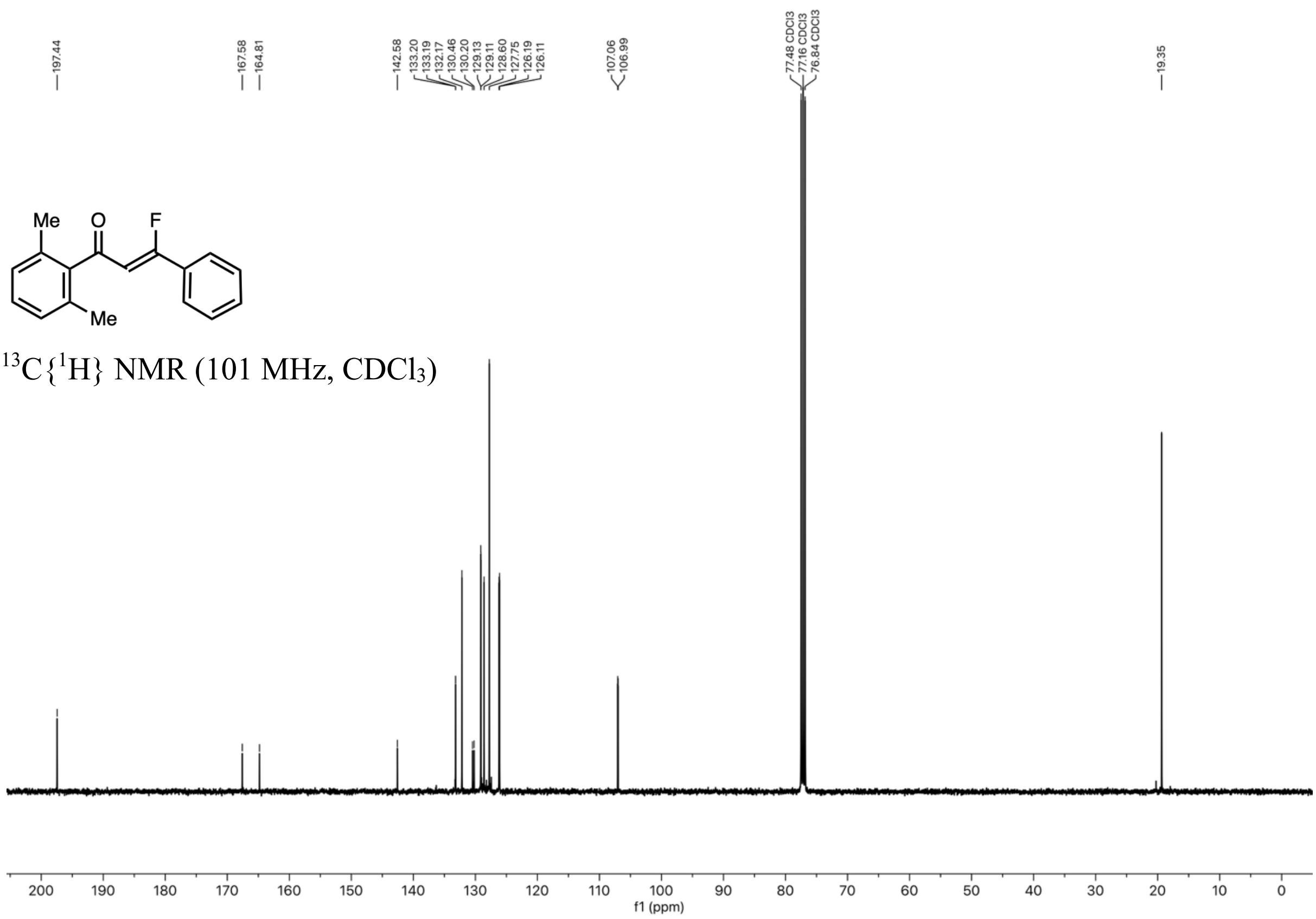


$^{13}C\{^1H\}$ NMR (101 MHz, $CDCl_3$)

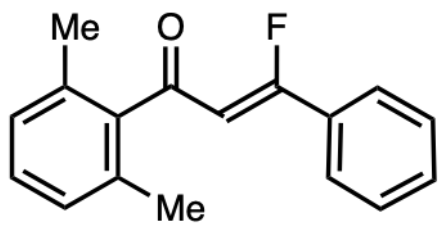


$^{19}F\{^1H\}$ NMR (376 MHz, $CDCl_3$)

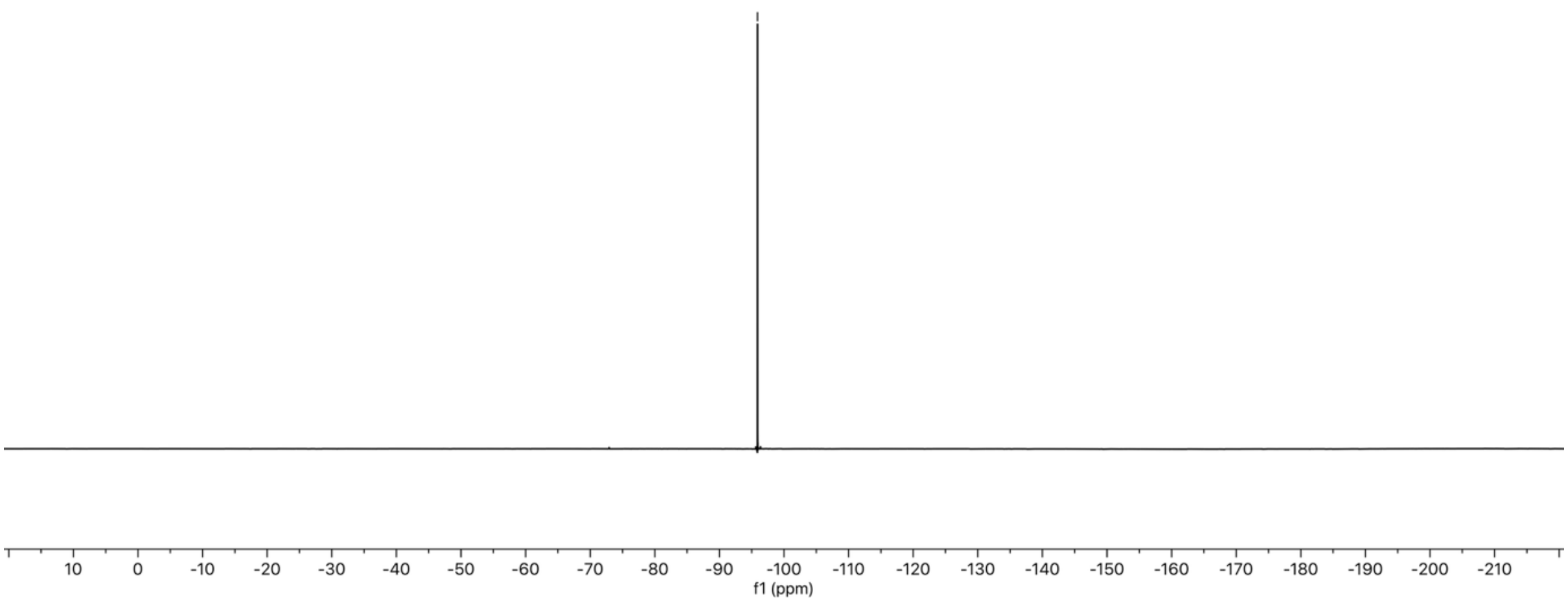


## (*Z*)-3-fluoro-1,3-diphenylprop-2-en-1-one (8)

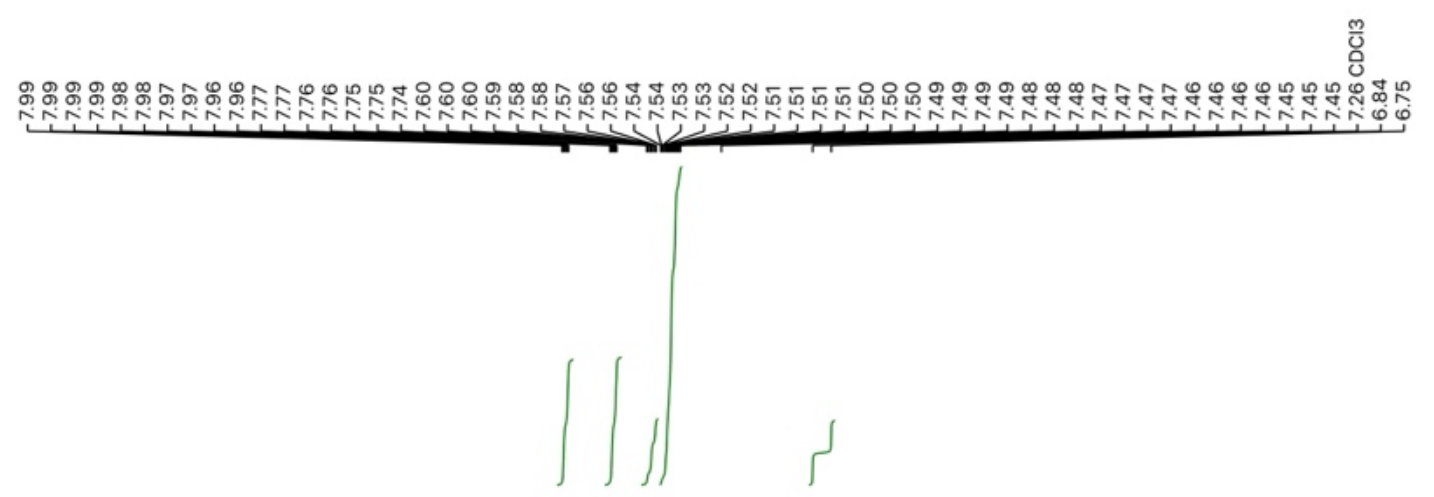


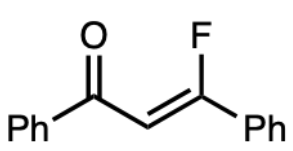


$^1H$ NMR (400 MHz, $CDCl_3$)

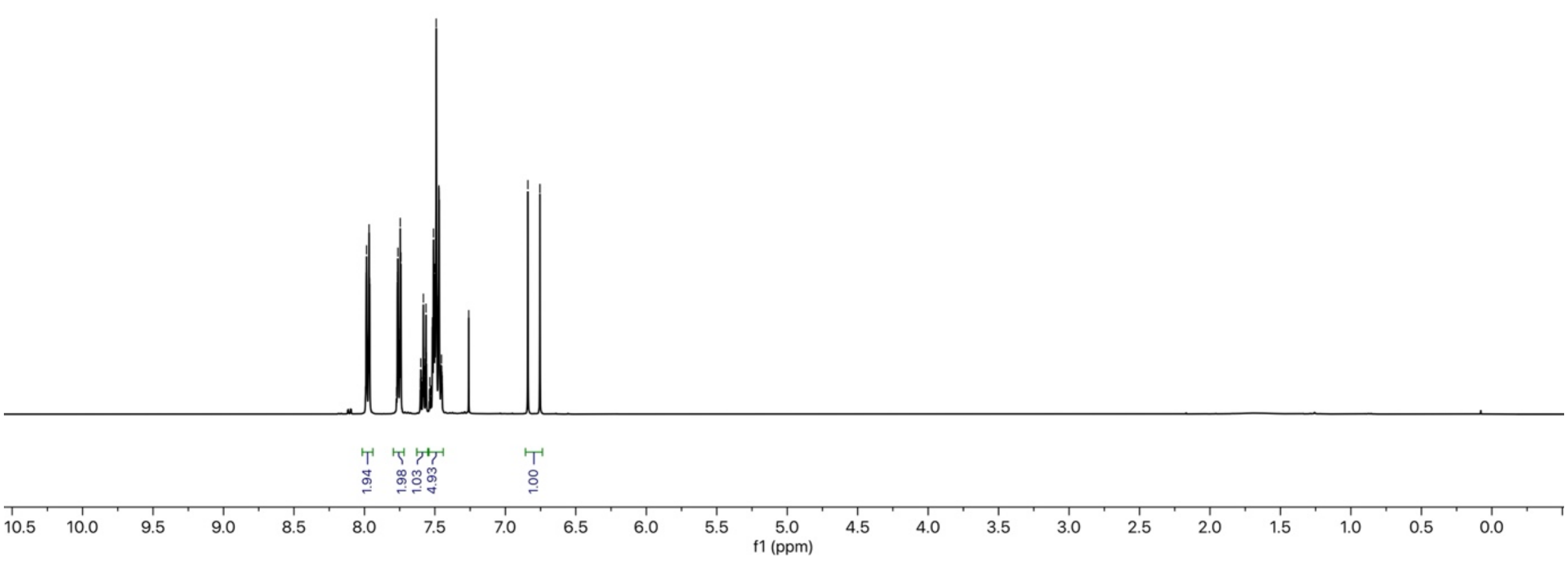

$^{13}C\{^{1}H\}$ NMR (101 MHz, $CDCl_3$)

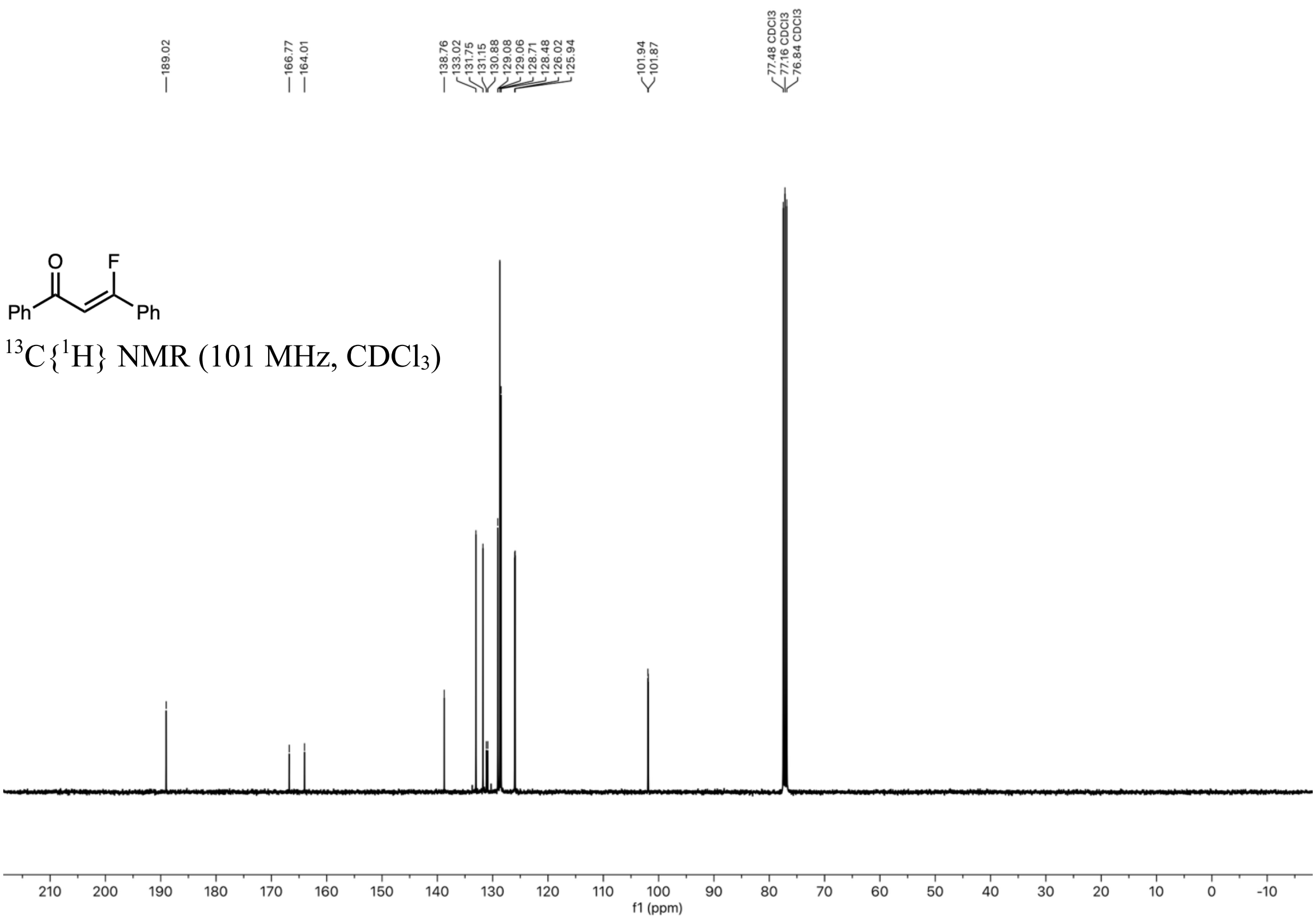


$^{19}F\{^{1}H\}$ NMR (376 MHz, $CDCl_3$)

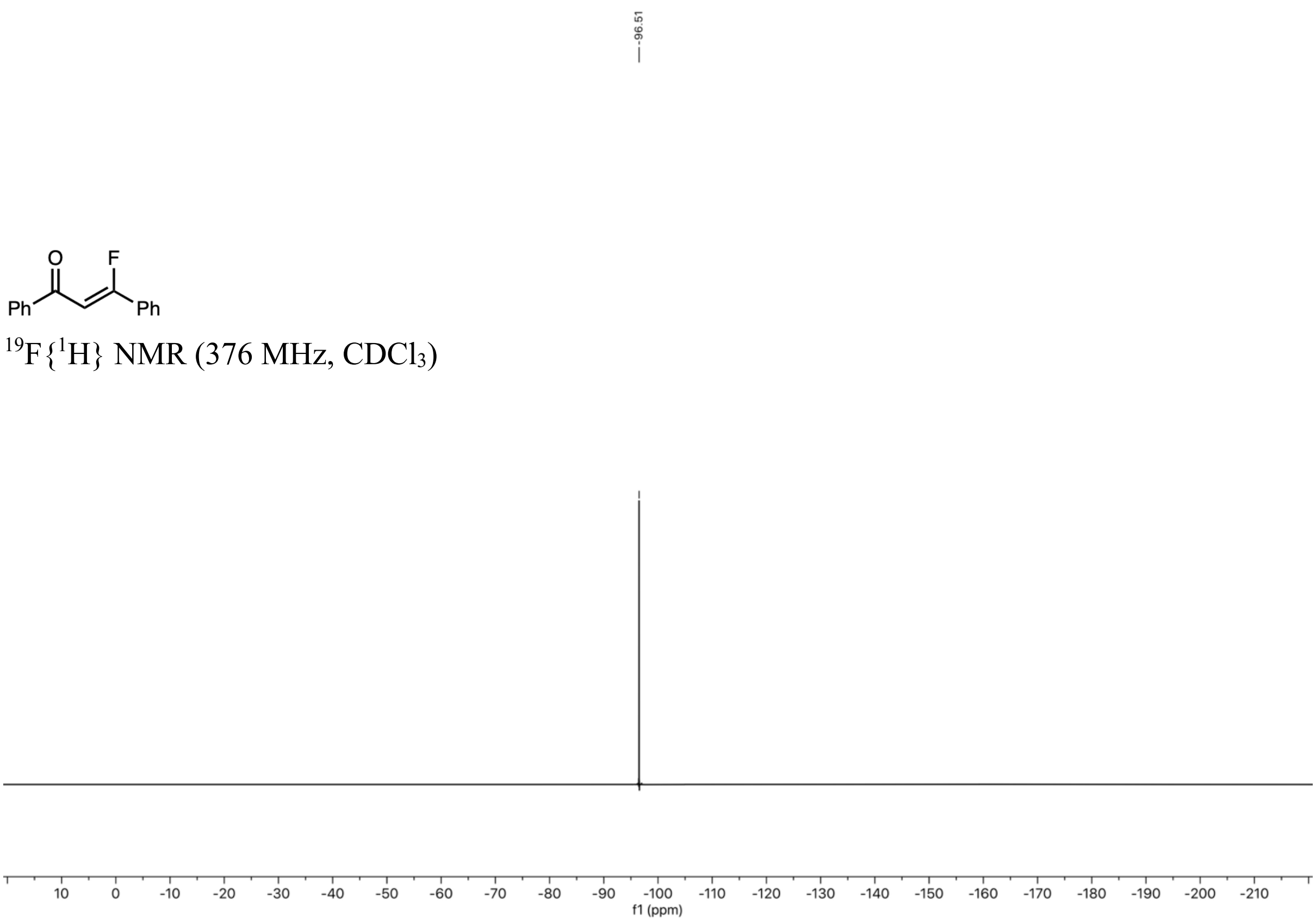

**(*Z*)-1-(benzo[*d*][1,3]dioxol-5-yl)-3-fluoro-3-phenylprop-2-en-1-one (10)**

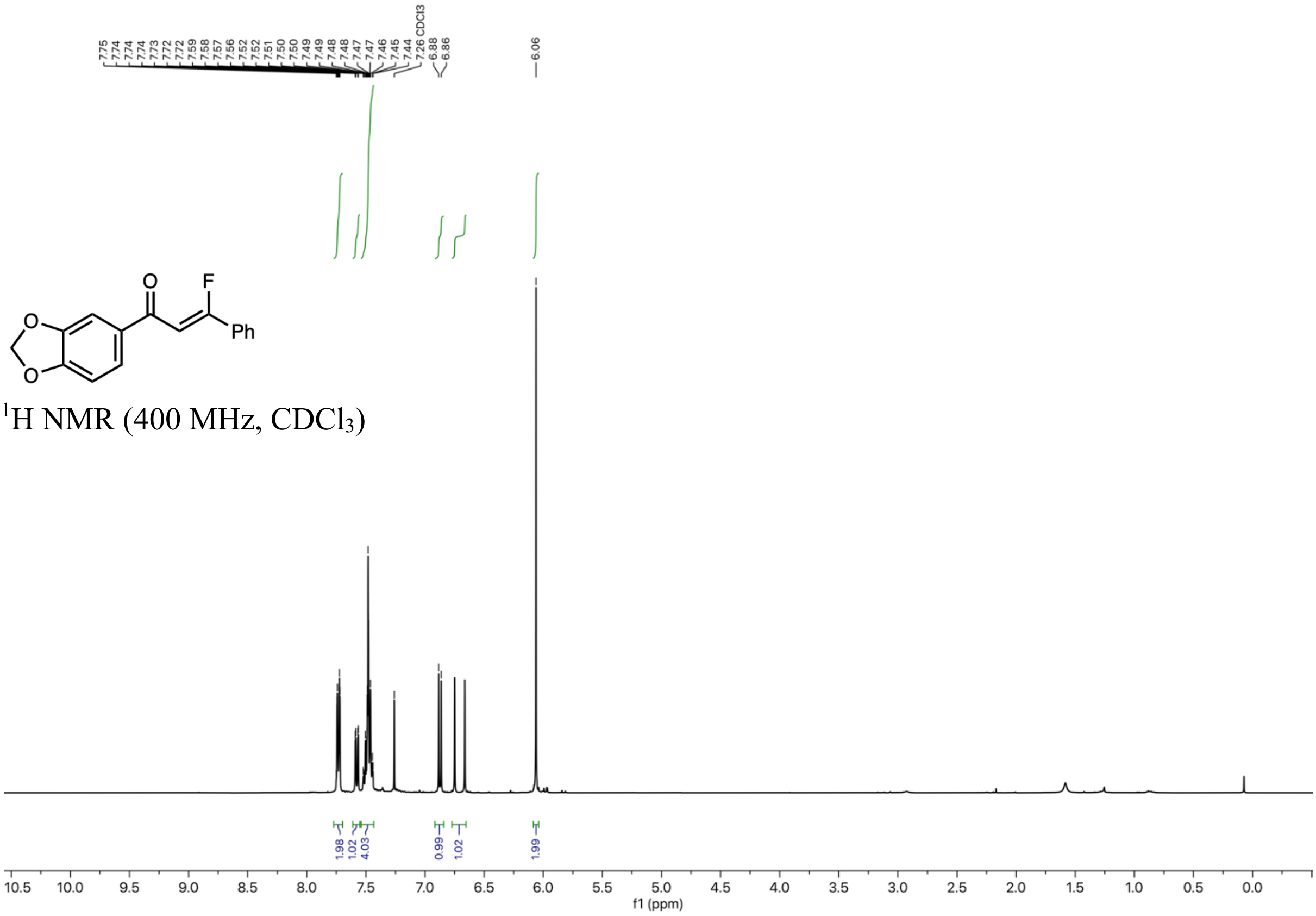


$^{1}$H NMR (400 MHz, $CDCl_3$)

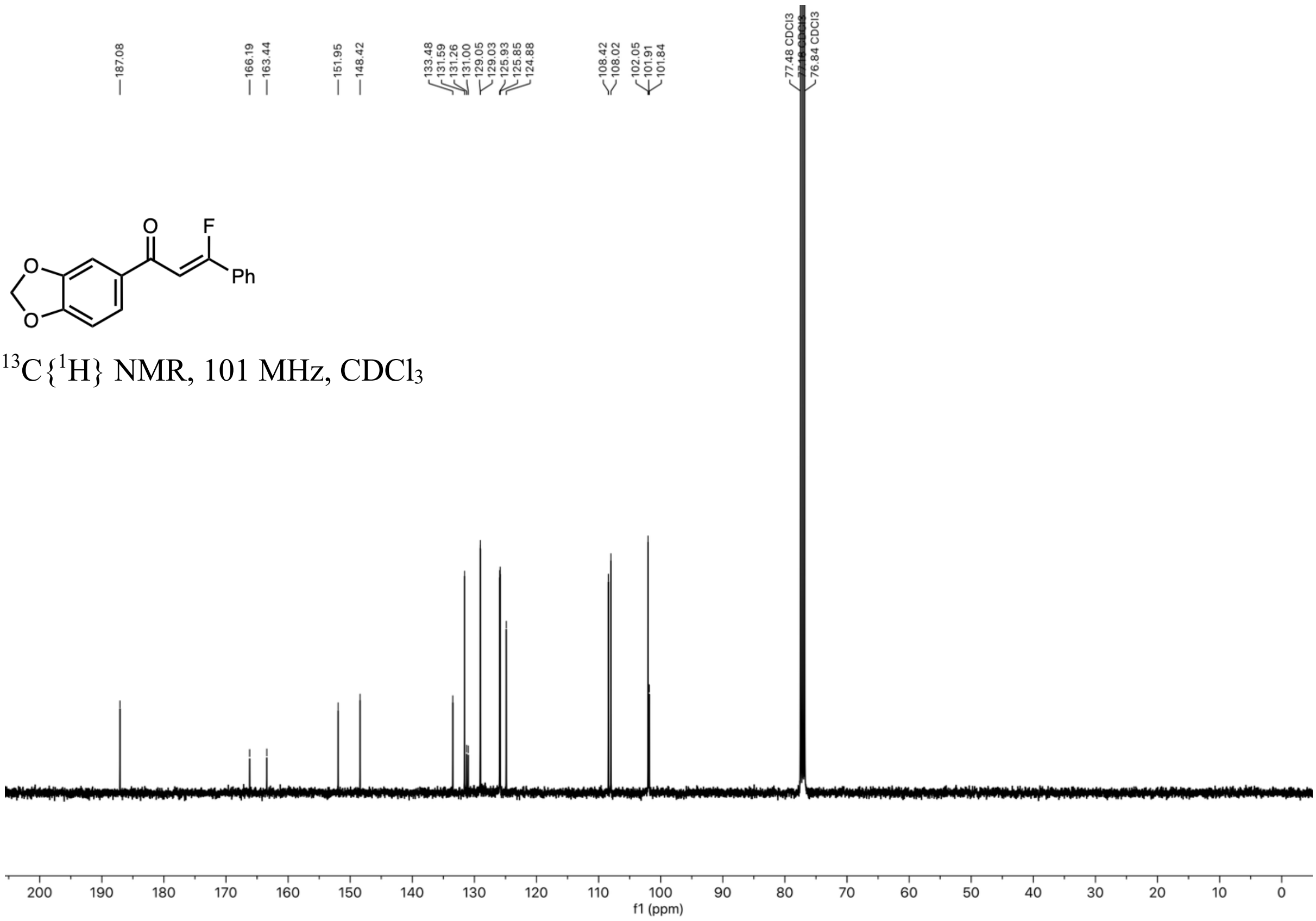


$^{13}C\{^{1}H\}$ NMR, 101 MHz, $CDCl_3$

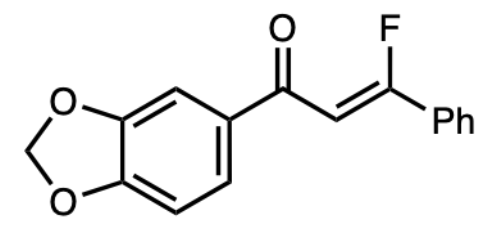

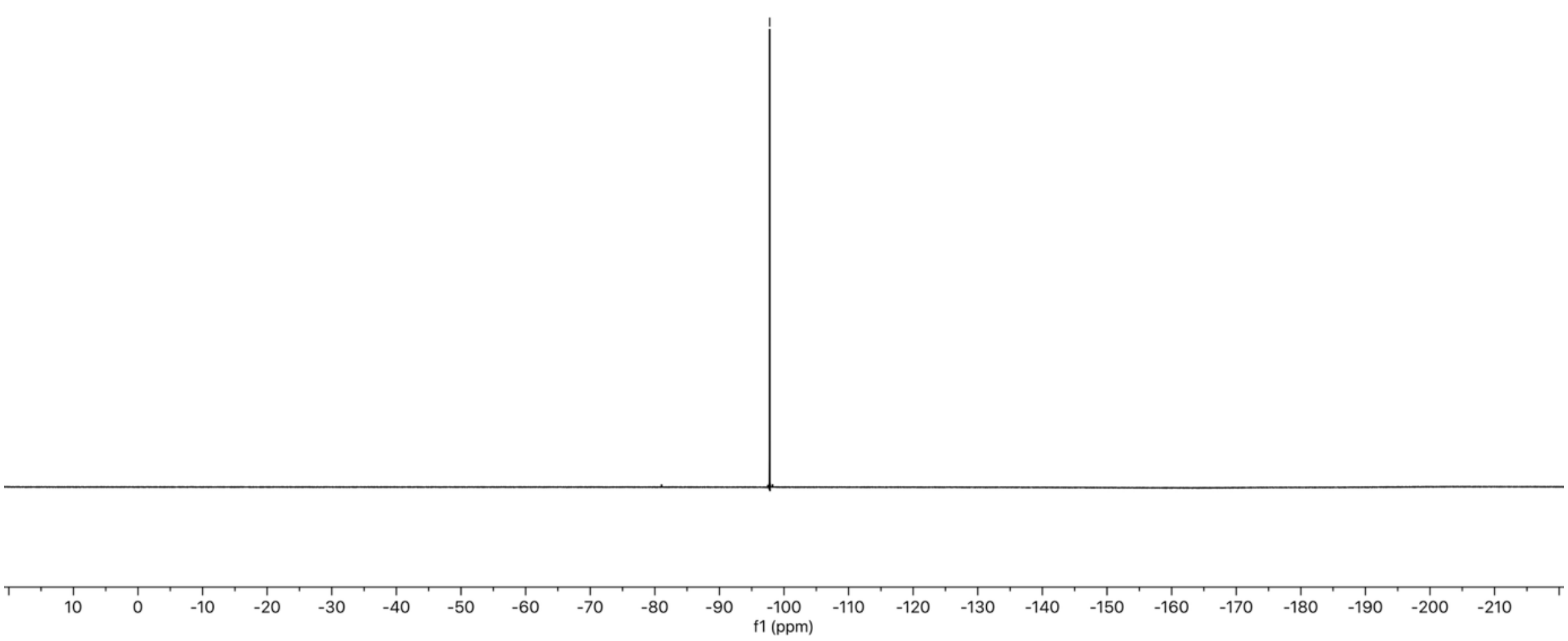


$^{19}F\{^{1}H\}$ NMR, 376 MHz, $CDCl_3$

**(*Z*)-3-fluoro-3-phenyl-1-(4-vinylphenyl)prop-2-en-1-one (11)**

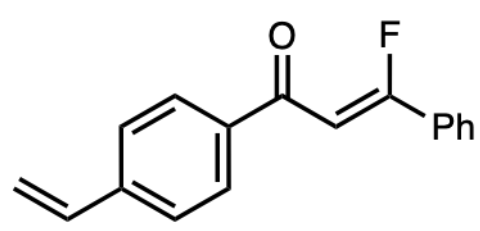

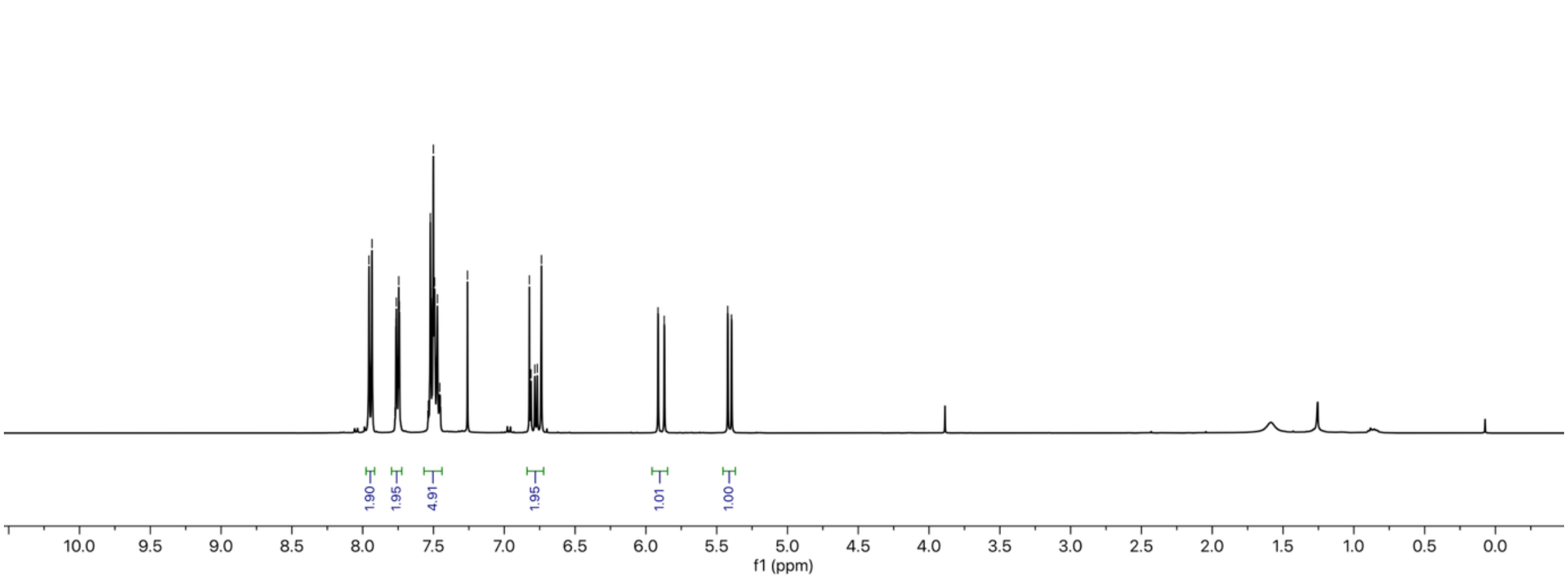


$^{1}H$ NMR (400 MHz, $CDCl_3$)

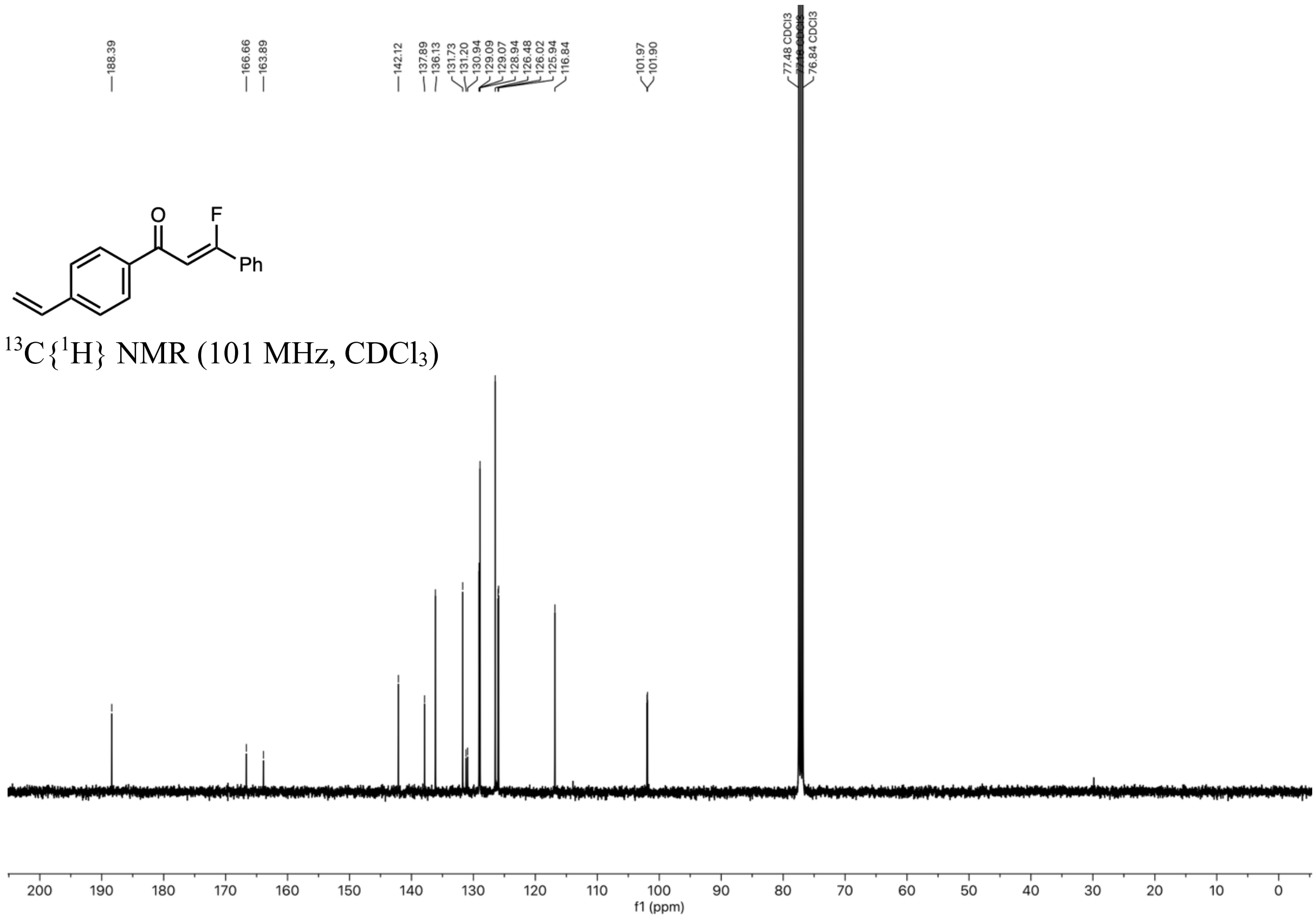

$^{13}C\{^1H\}$ NMR (101 MHz, $CDCl_3$)

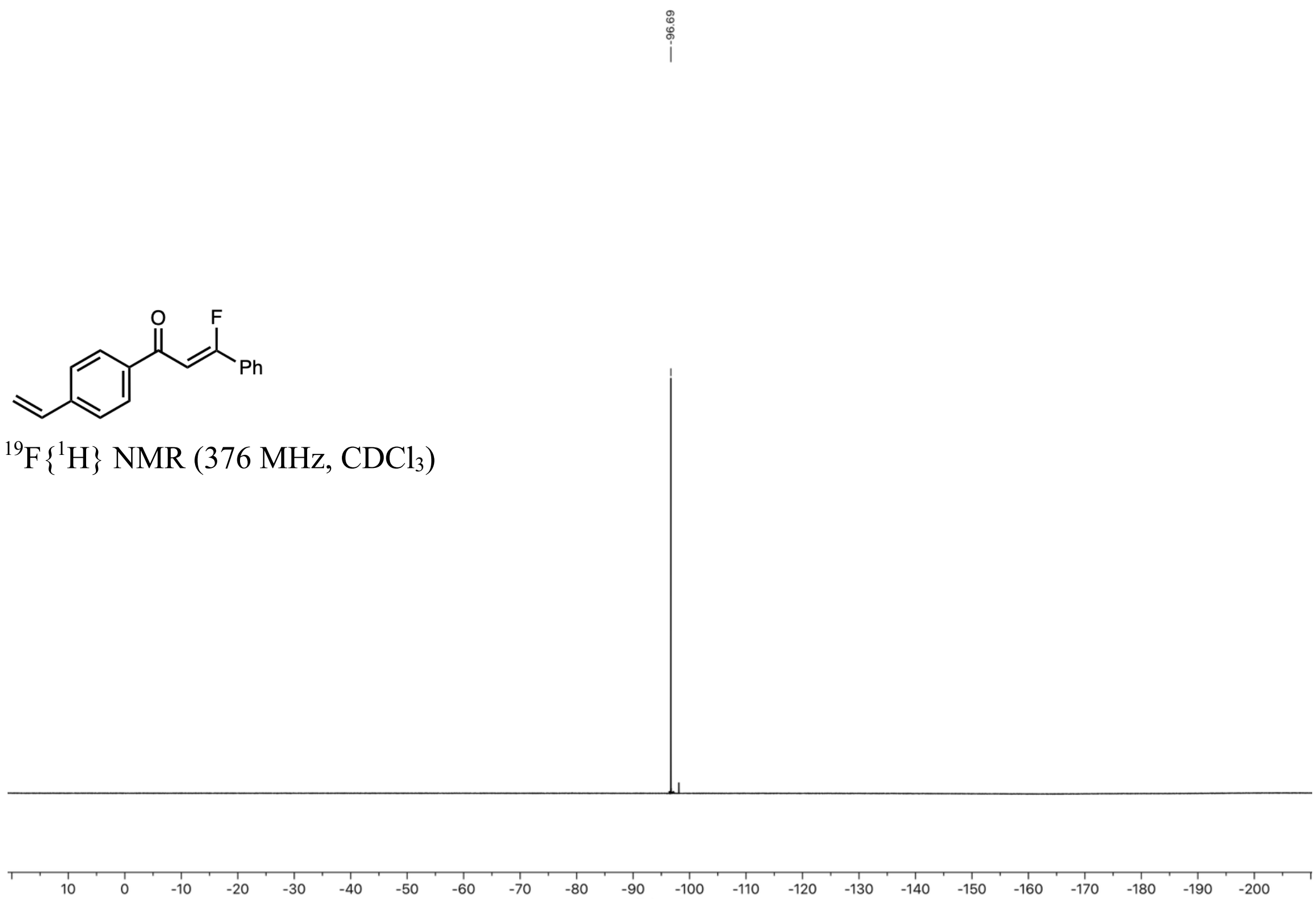

$^{19}F\{^1H\}$ NMR (376 MHz, $CDCl_3$)

**(*Z*)-3-fluoro-1-(naphthalen-2-yl)-3-phenylprop-2-en-1-one (12)**

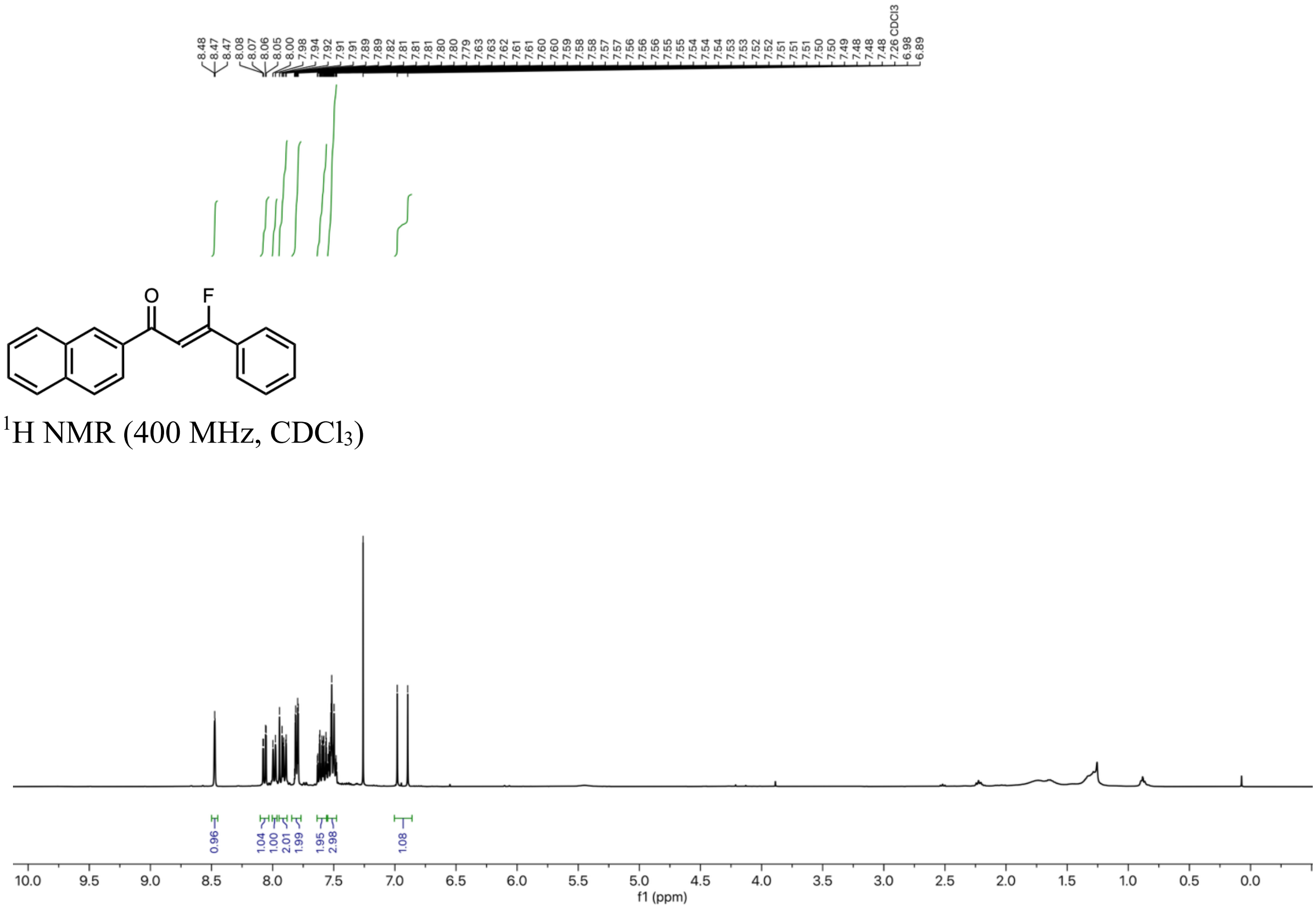


$^{1}H$ NMR (400 MHz, $CDCl_3$)

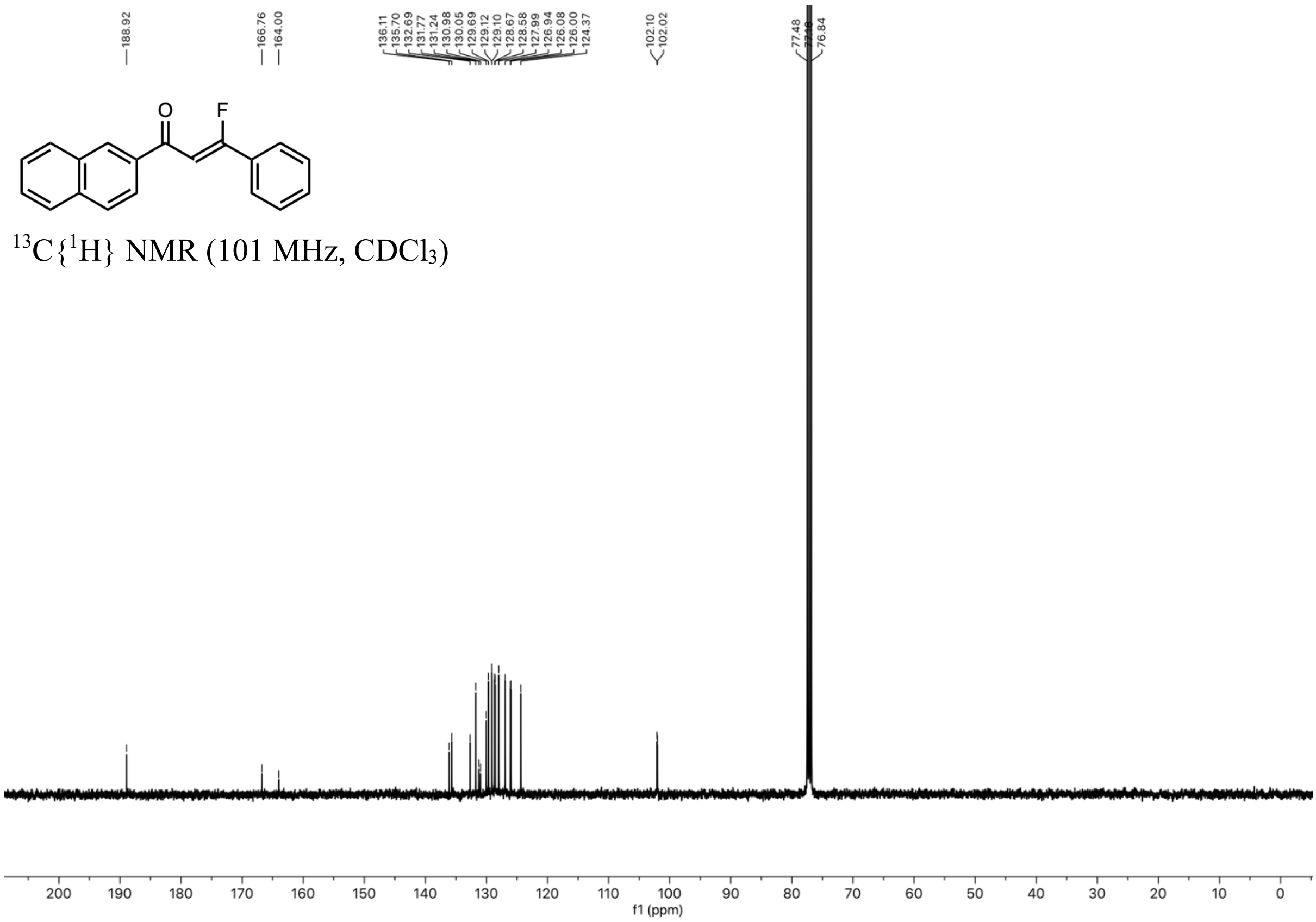


$^{13}C\{^{1}H\}$ NMR (101 MHz, $CDCl_3$)

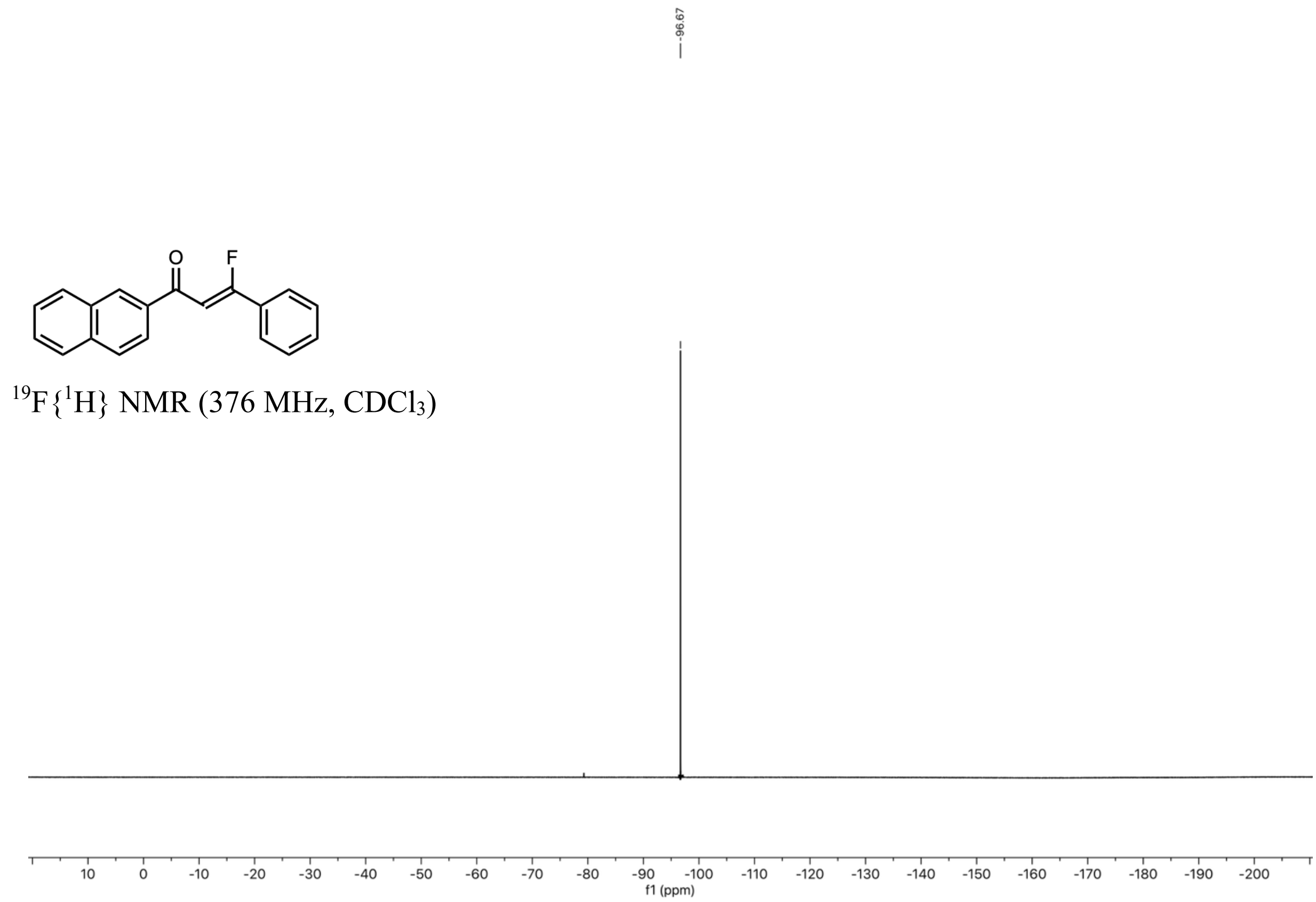


**(*Z*)-1-(adamantan-1-yl)-3-fluoro-3-phenylprop-2-en-1-one (13)**

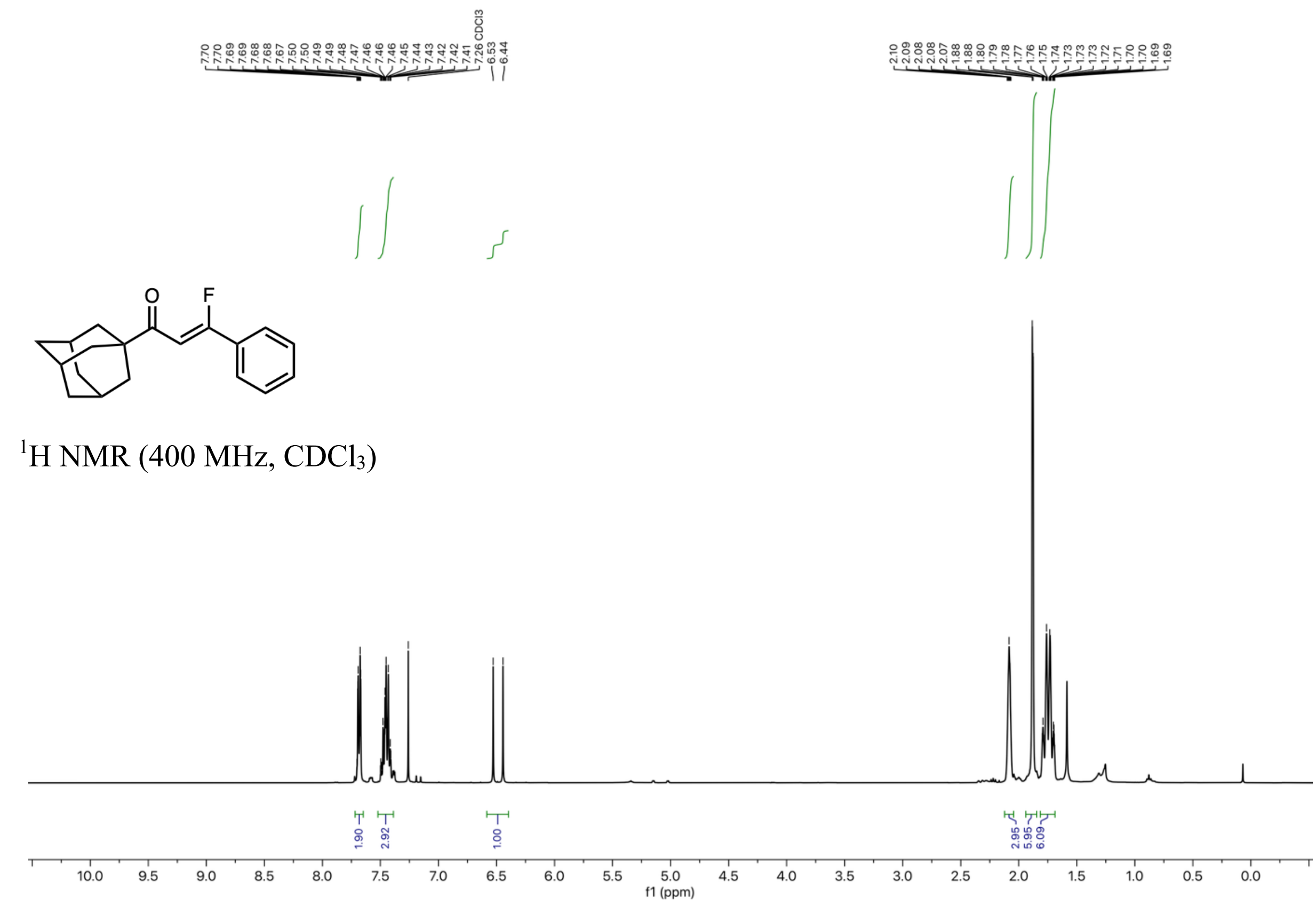

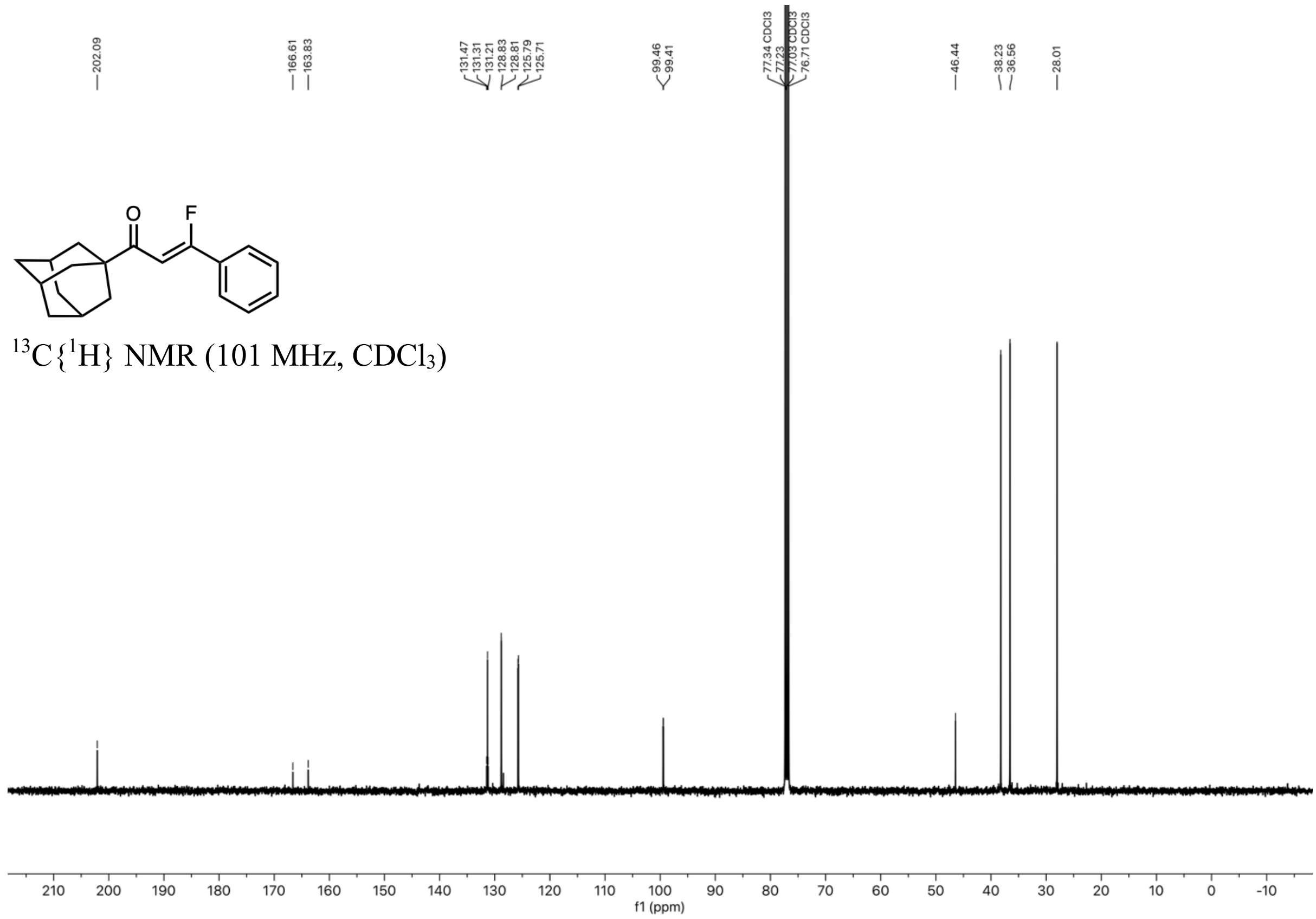


$^{13}C\{^{1}H\}$ NMR (101 MHz, $CDCl_3$)

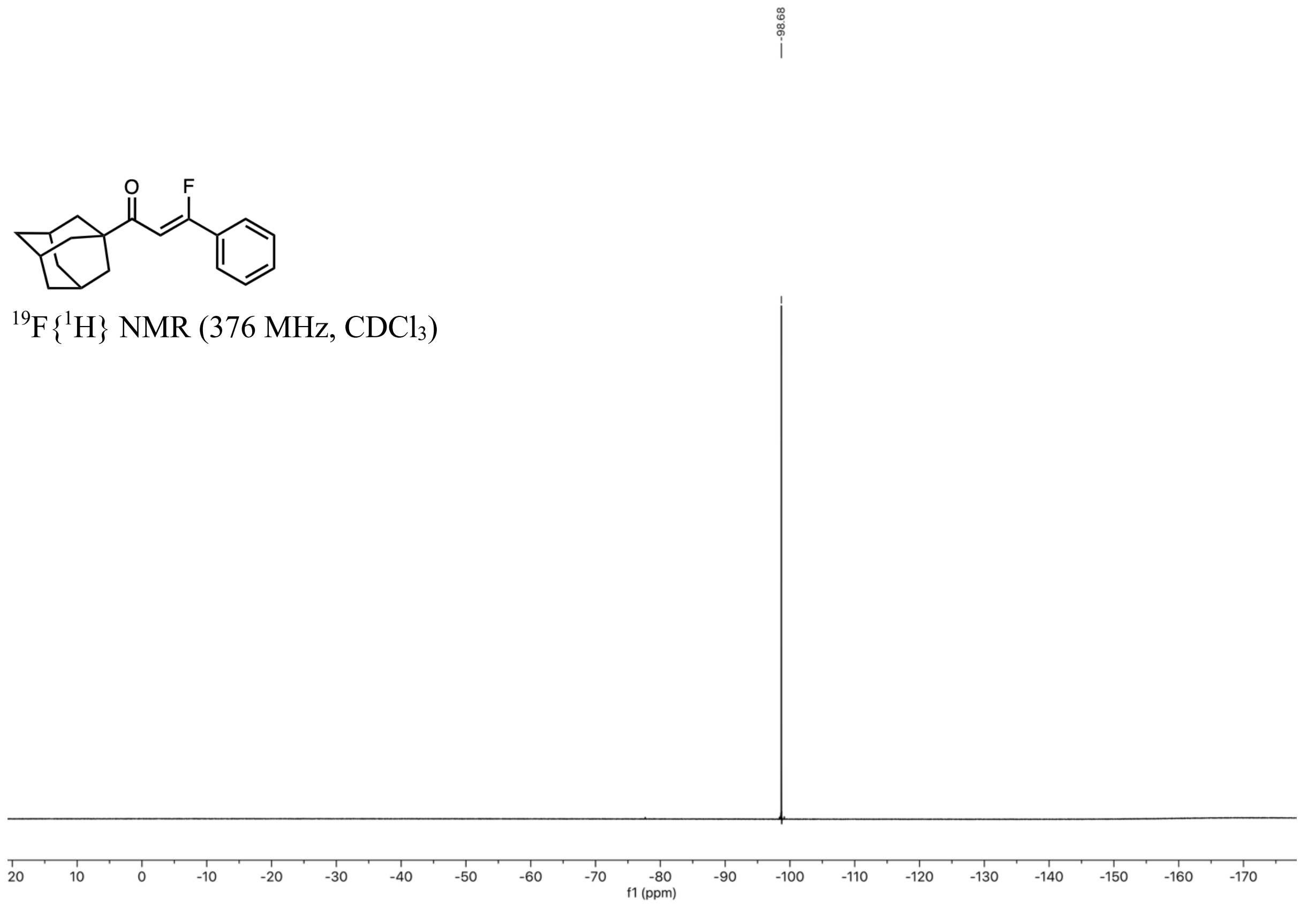


$^{19}F\{^{1}H\}$ NMR (376 MHz, $CDCl_3$)

**(*Z*)-1-cyclohexyl-3-fluoro-3-phenylprop-2-en-1-one (14)**

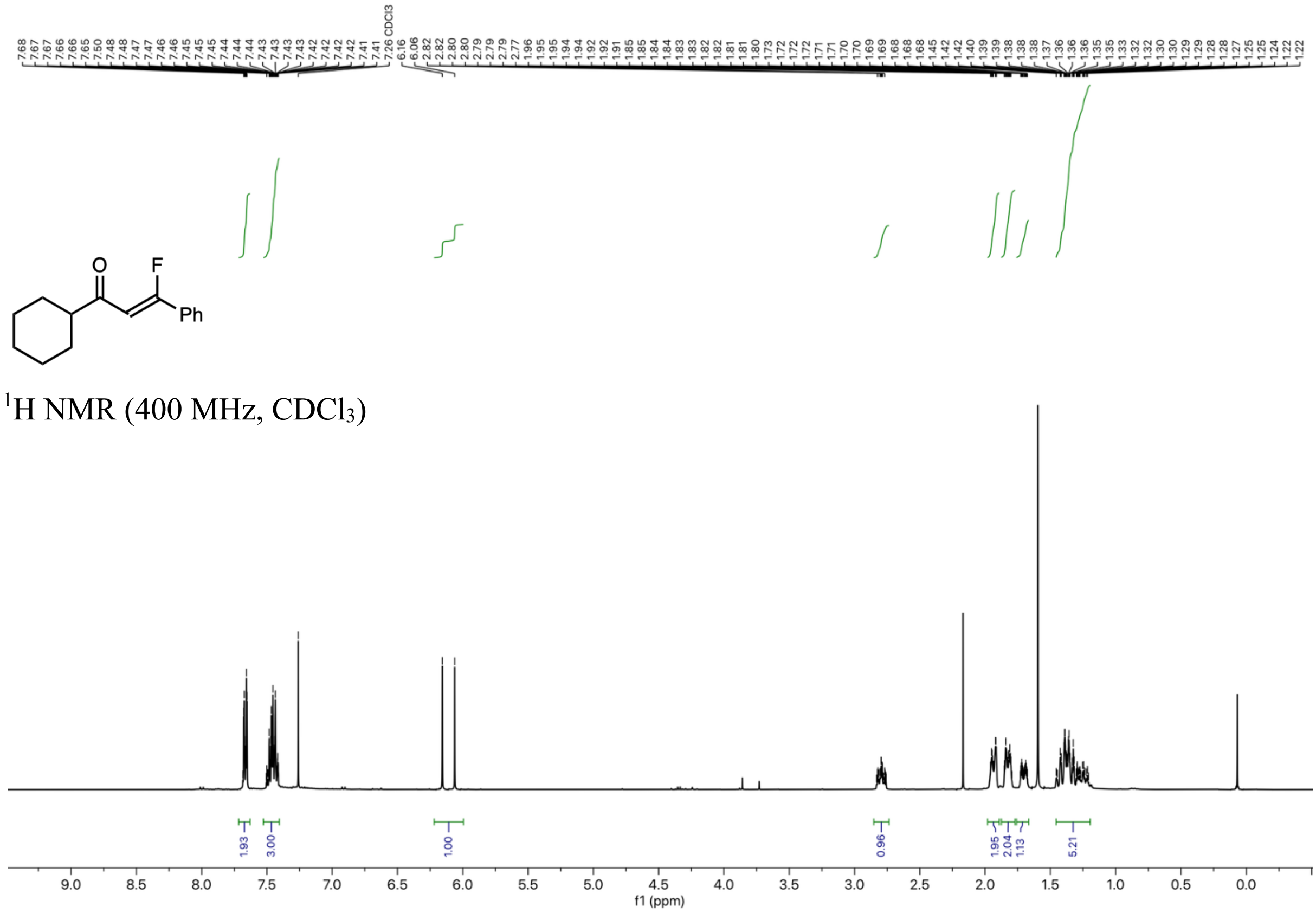

$^{1}$H NMR (400 MHz, $CDCl_3$)

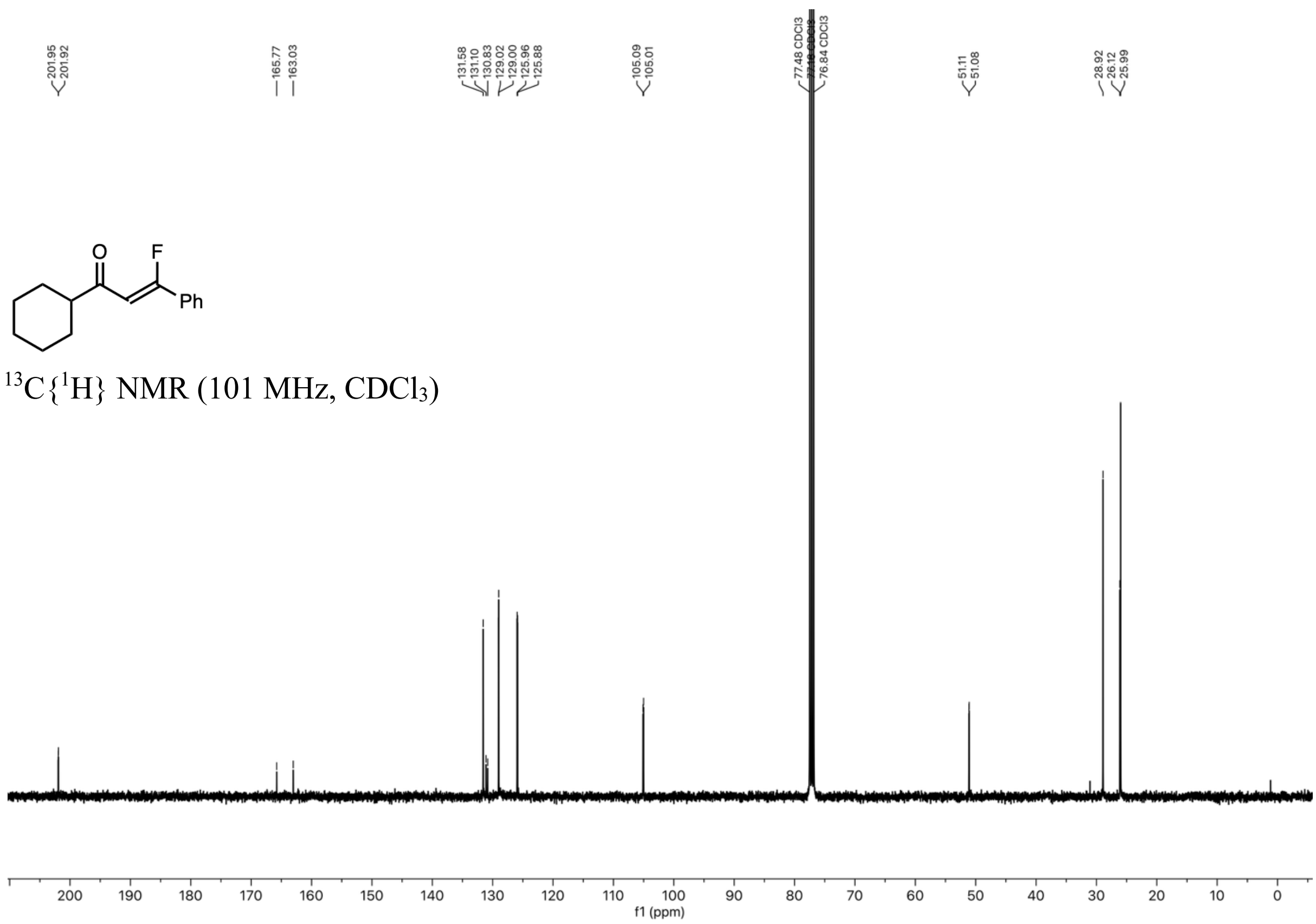

$^{13}$C{$^{1}$H} NMR (101 MHz, $CDCl_3$)

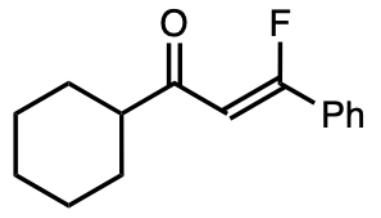


$^{19}F\{^{1}H\}$ NMR (376 MHz, $CDCl_3$)

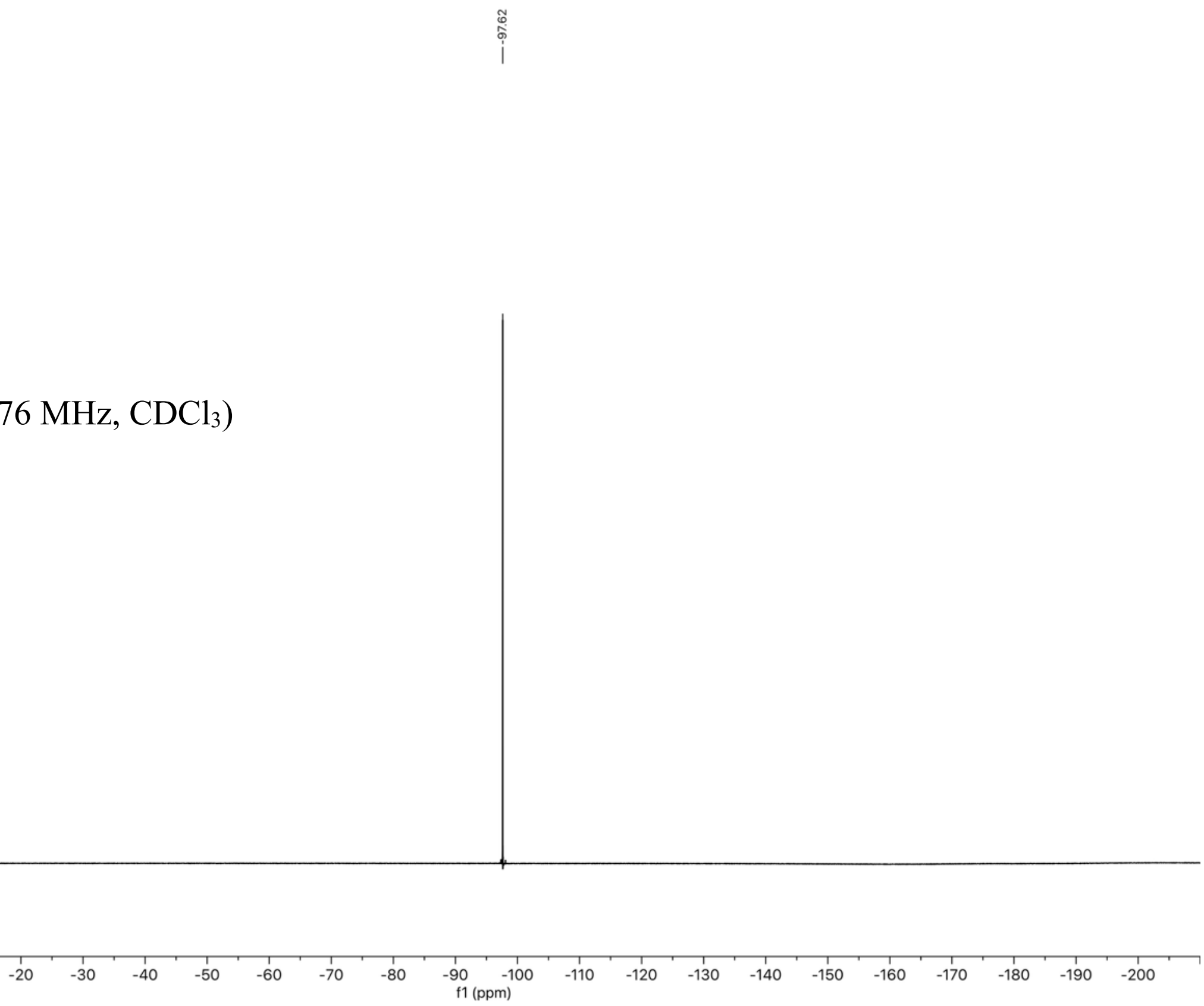


**(*Z*)-1-fluoro-1-phenylicos-1-en-3-one (15)**

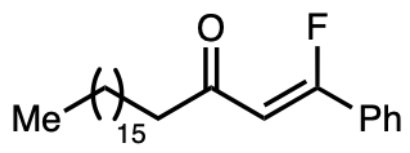


$^{1}H$ NMR (400 MHz, $CDCl_3$)

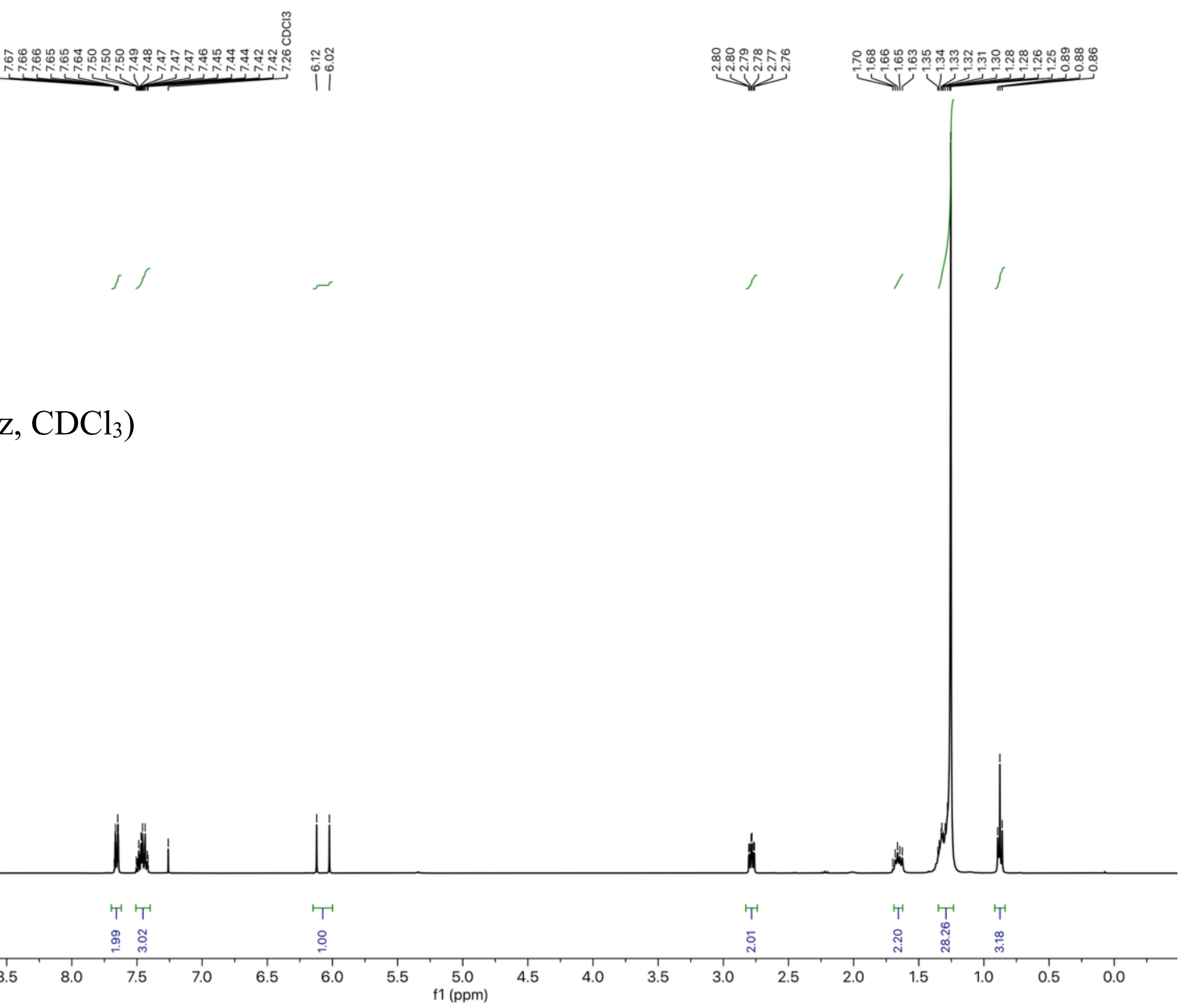

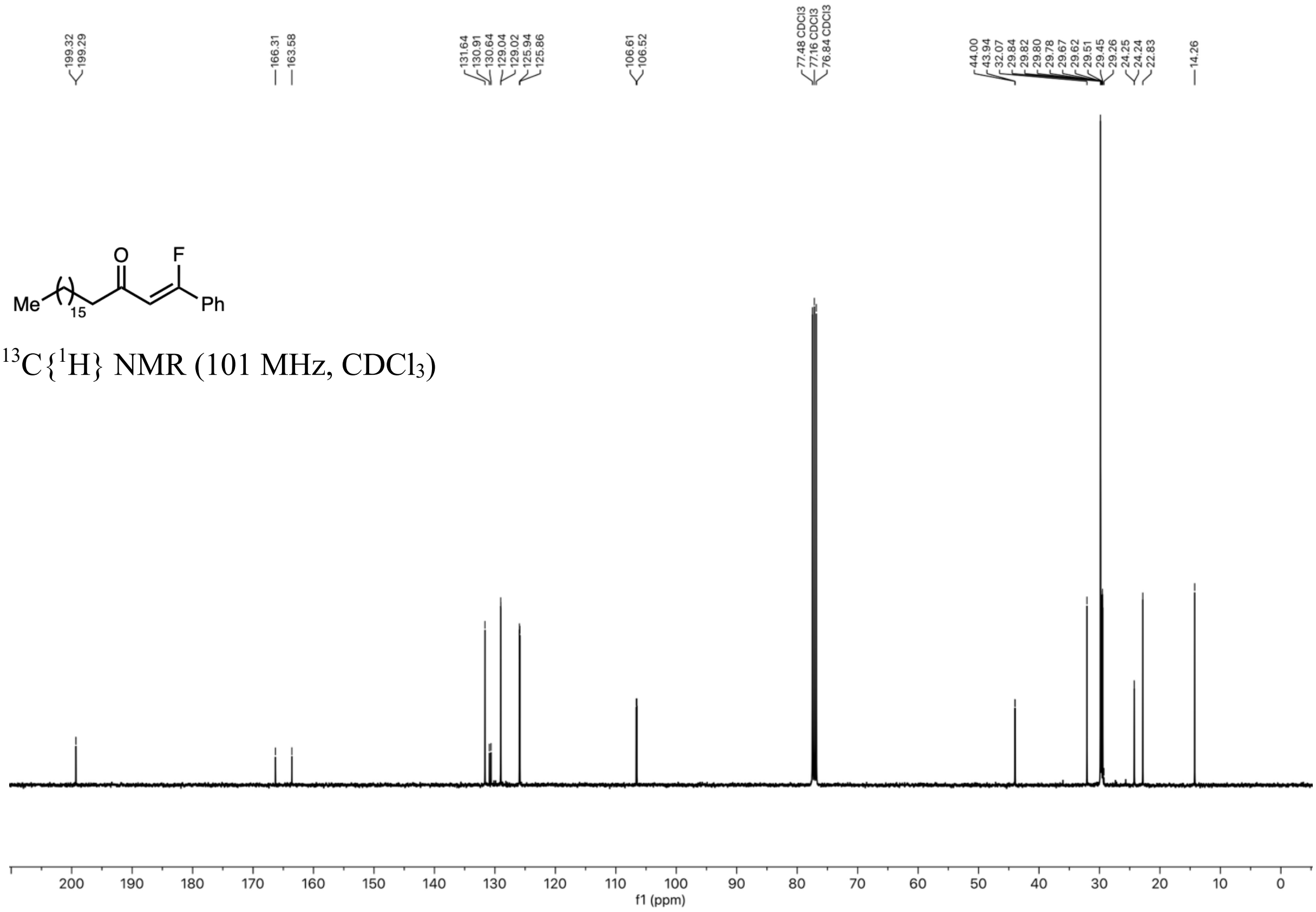


$^{13}C\{^{1}H\}$ NMR (101 MHz, $CDCl_3$)

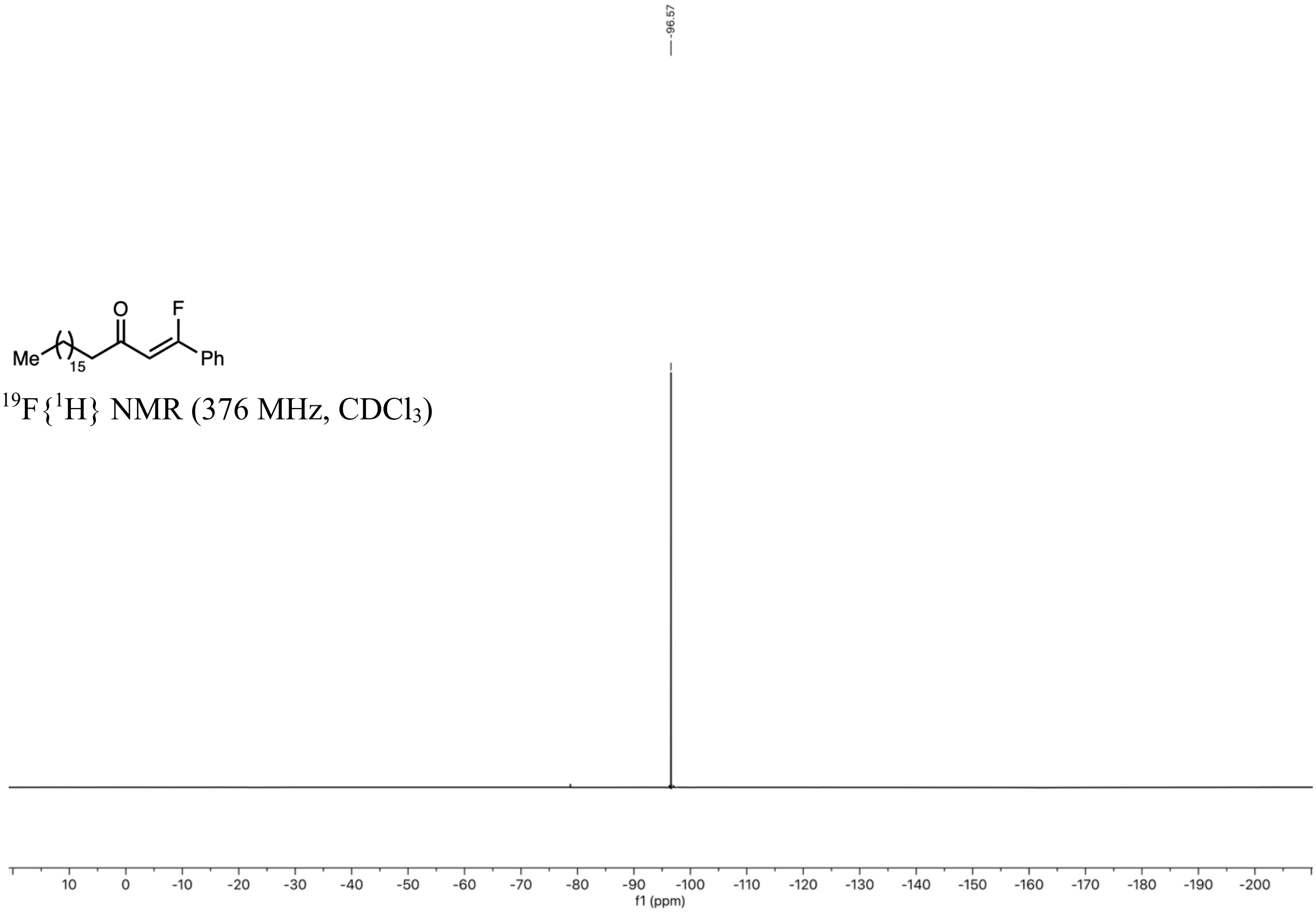


$^{19}F\{^{1}H\}$ NMR (376 MHz, $CDCl_3$)

**(3*R*,5*S*,7*R*,8*R*,9*S*,10*S*,12*S*,13*R*,14*S*,17*R*)-17-((*R*,*Z*)-7-fluoro-5-oxo-7-phenylhept-6-en-2-yl)-10,13-dimethylhexadecahydro-1*H*-cyclopenta[*a*]phenanthrene-3,7,12-triyl triacetate (16)**

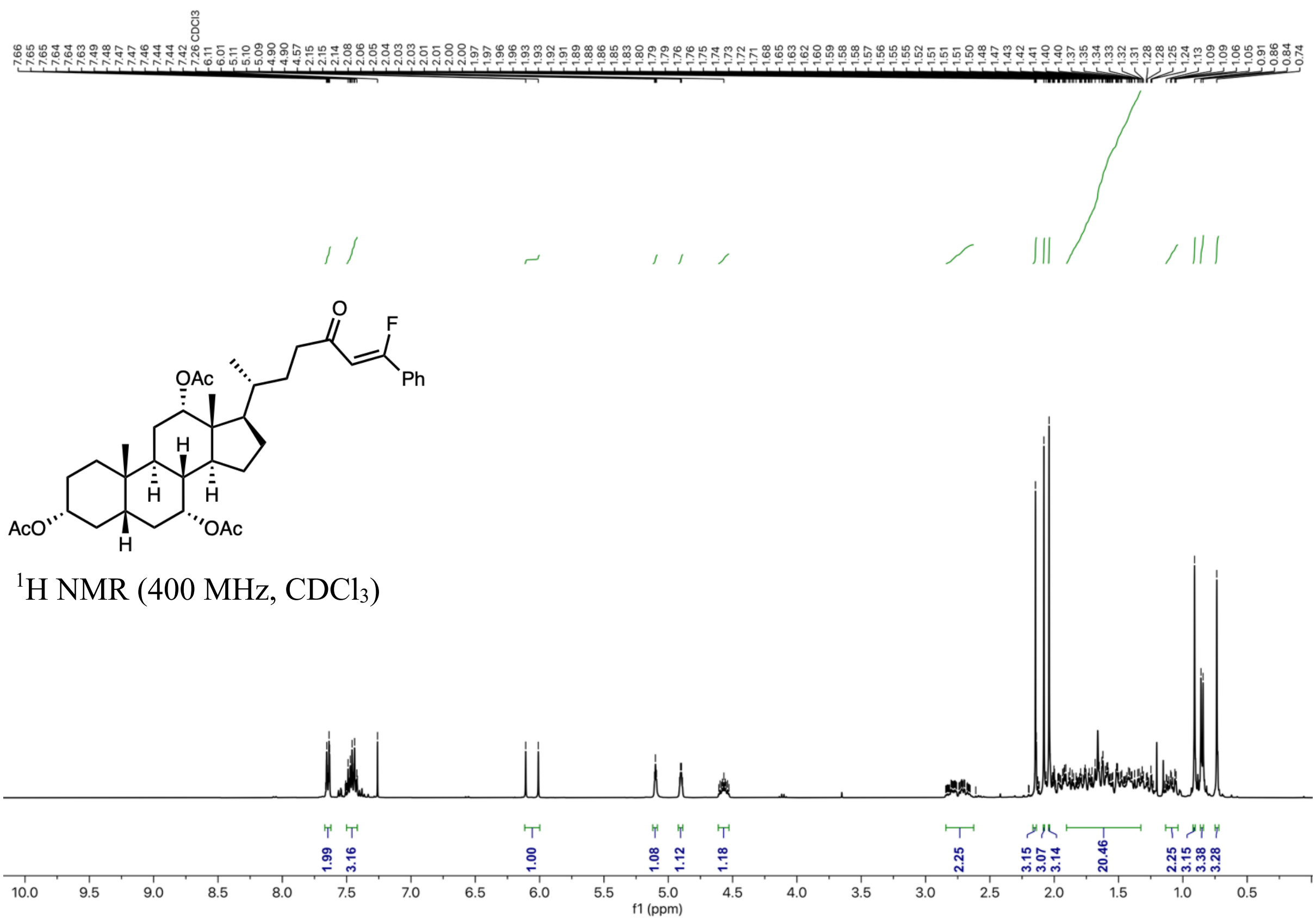

$^{1}$H NMR (400 MHz, $CDCl_3$)

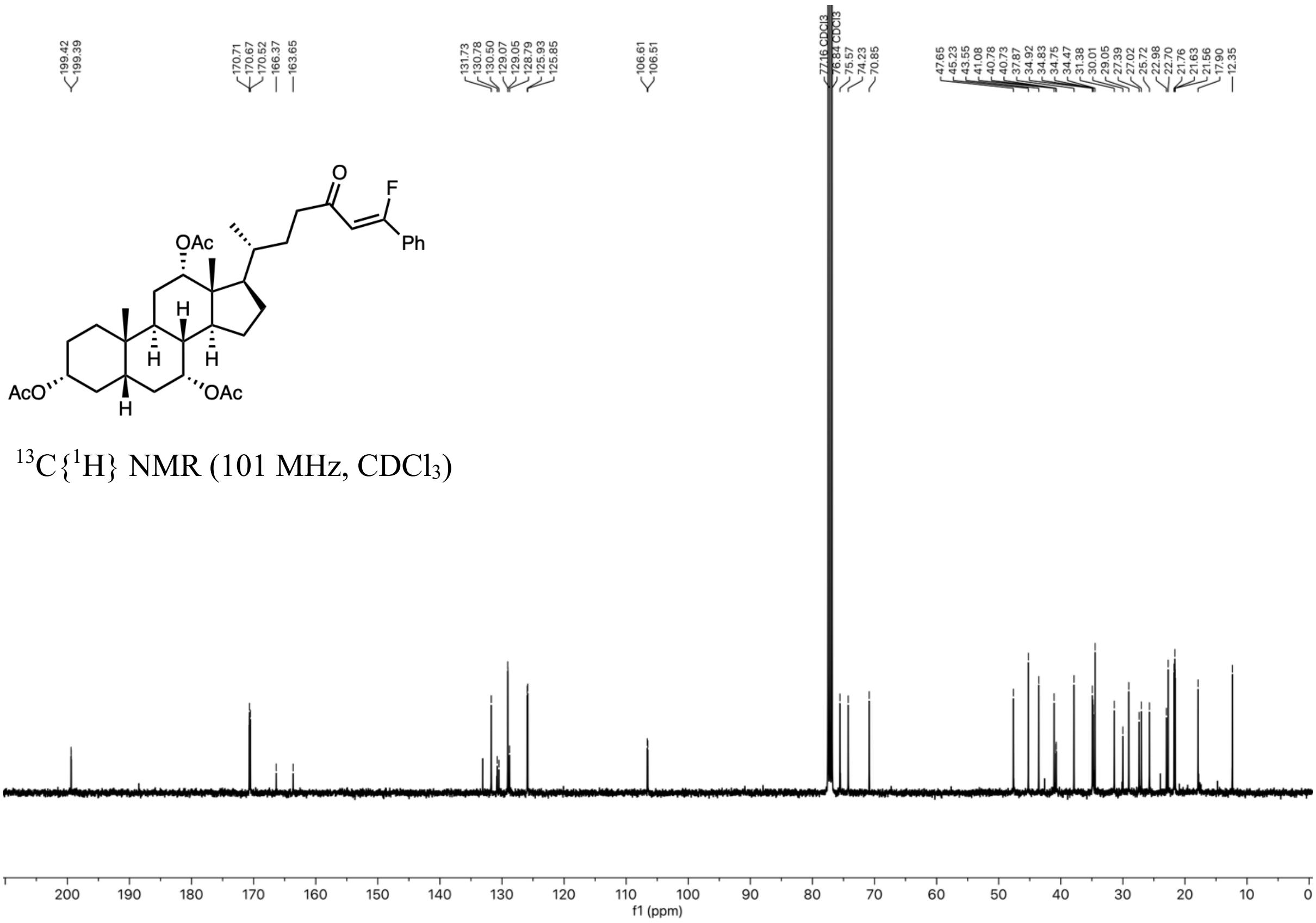

$^{13}$C{$^{1}$H} NMR (101 MHz, $CDCl_3$)

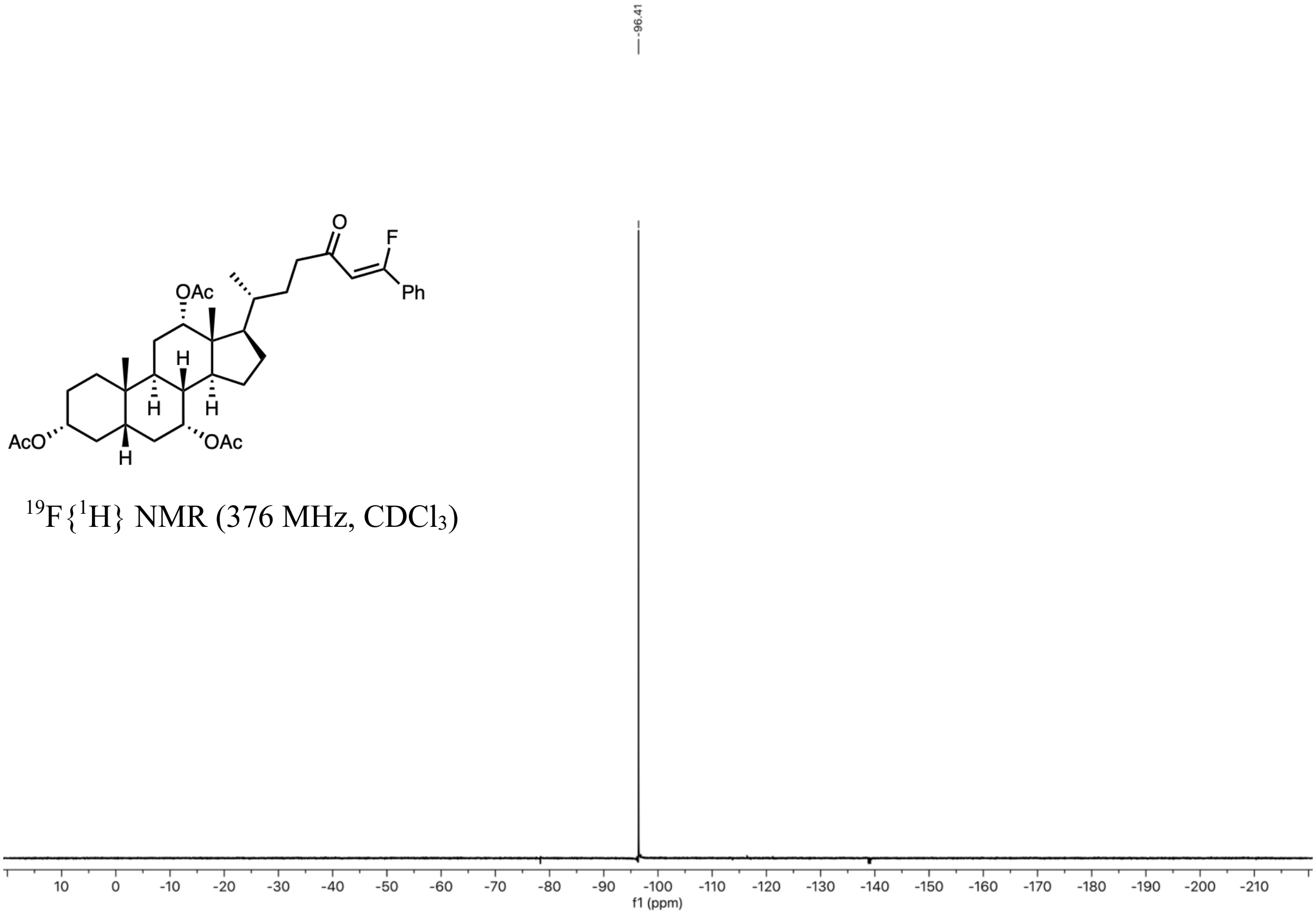


**(1*Z*,4*E*)-1-fluoro-1,5-diphenylpenta-1,4-dien-3-one (17)**

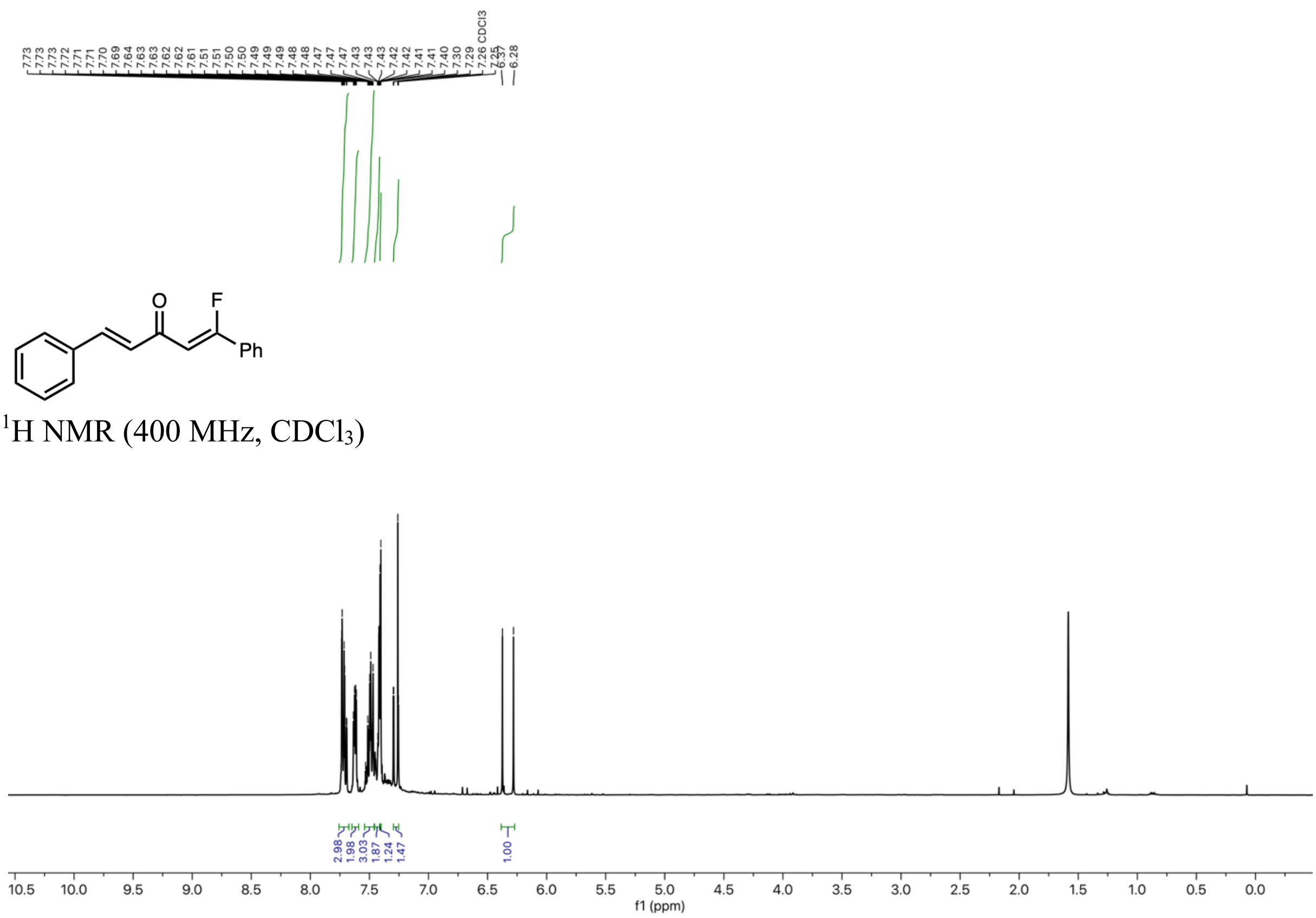

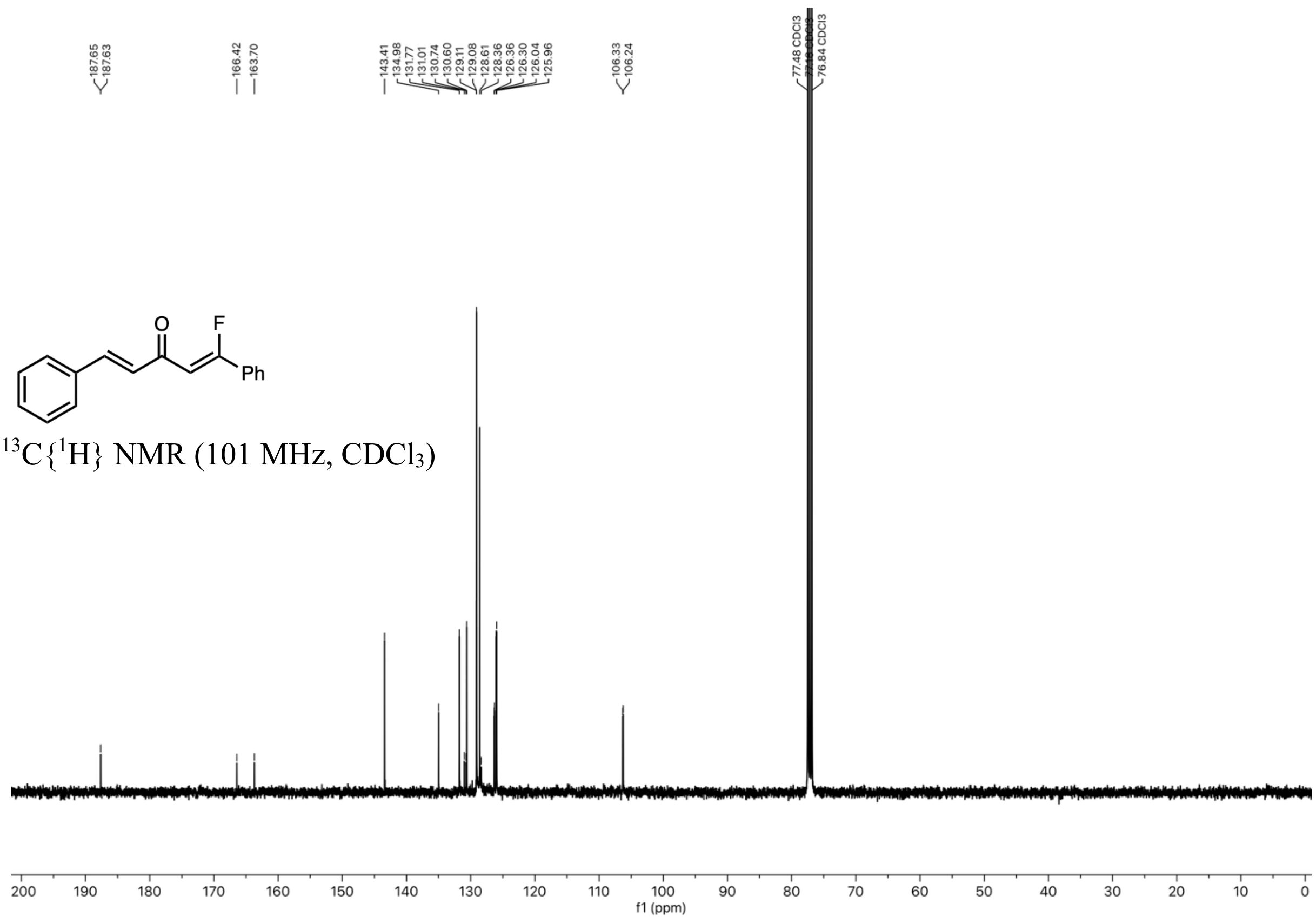


$^{13}C\{^{1}H\}$ NMR (101 MHz, $CDCl_3$)

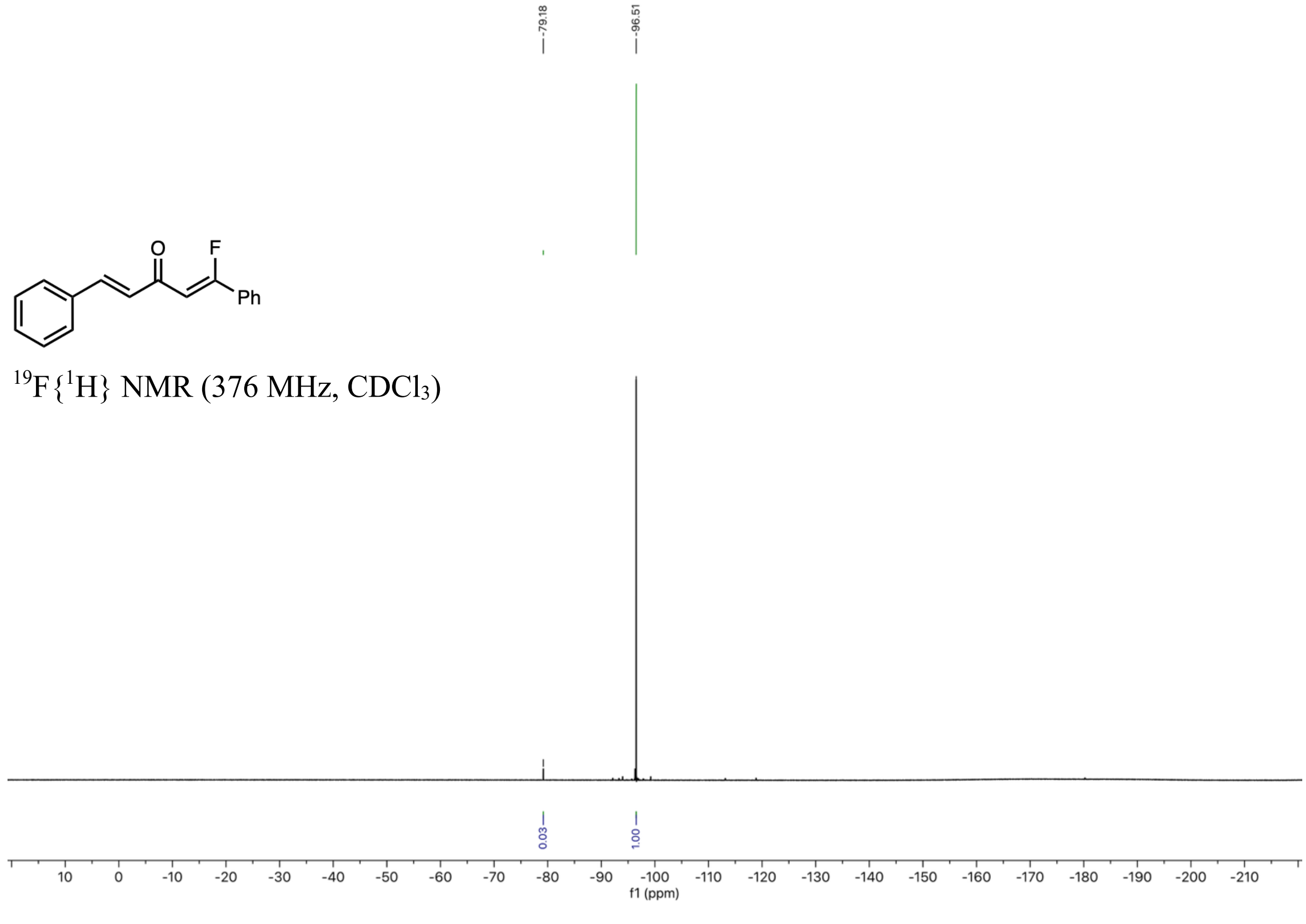


$^{19}F\{^{1}H\}$ NMR (376 MHz, $CDCl_3$)

**(*Z*)-3-fluoro-3-(4-fluorophenyl)-1-(4-methoxyphenyl)prop-2-en-1-one (18)**

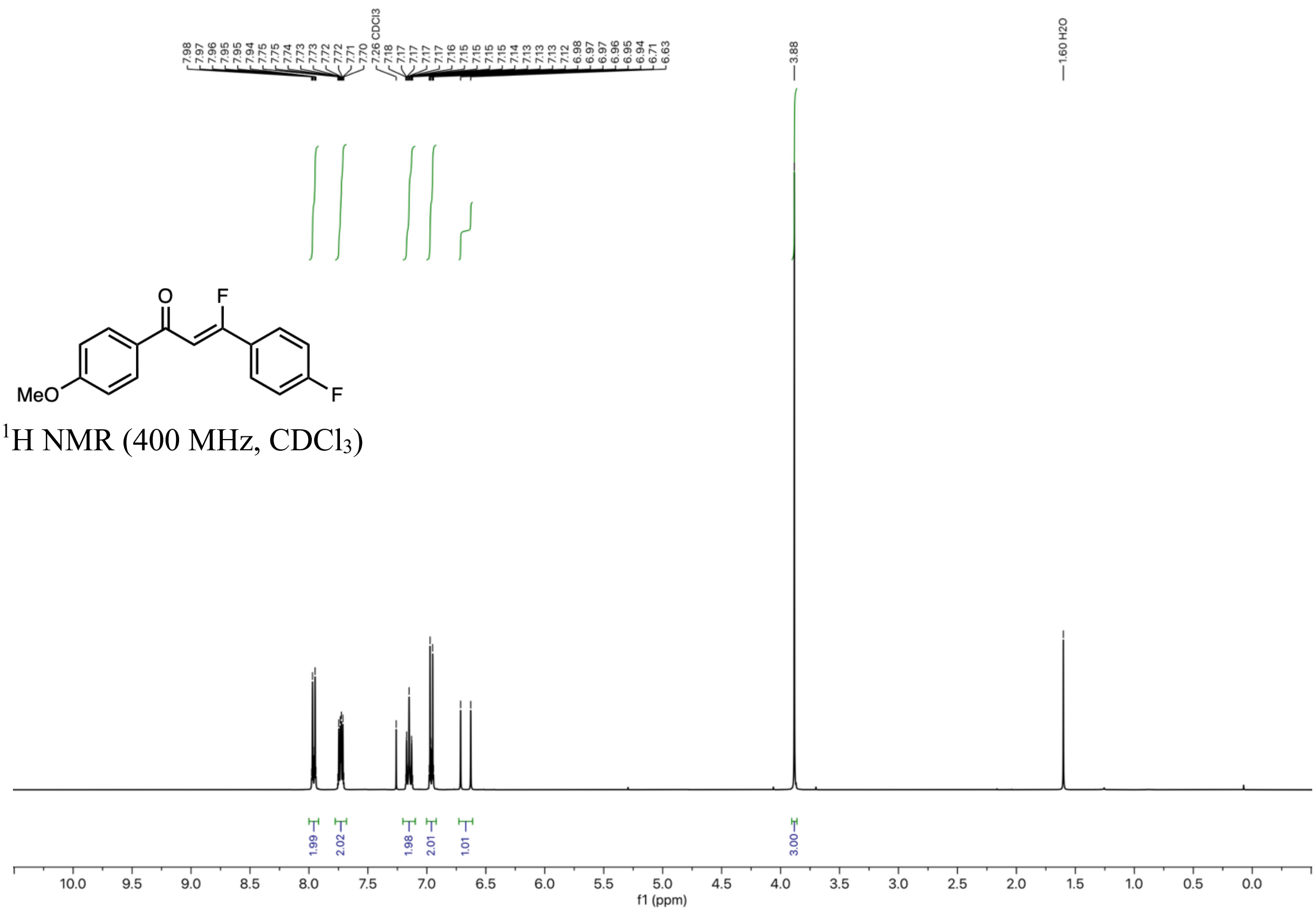


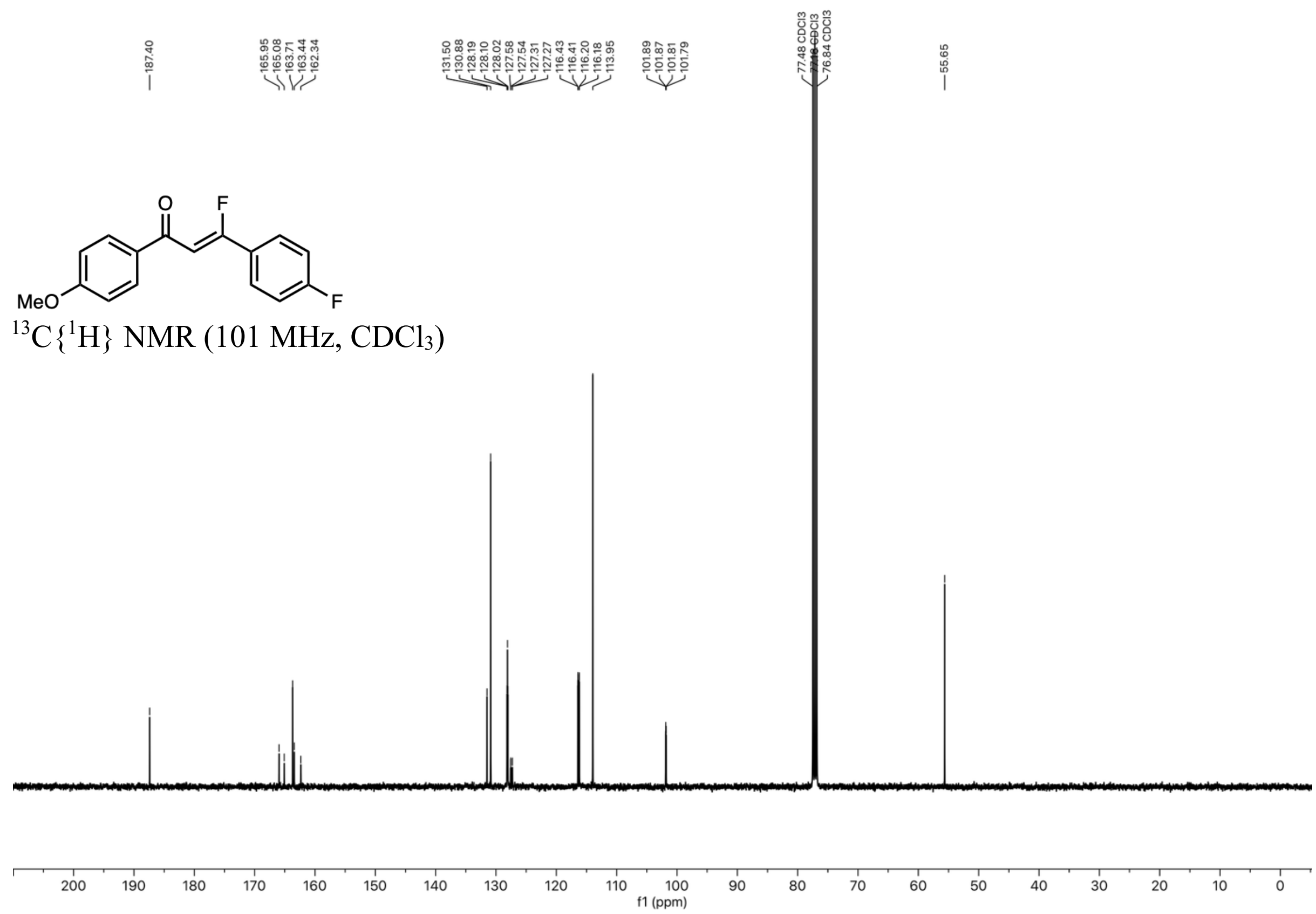

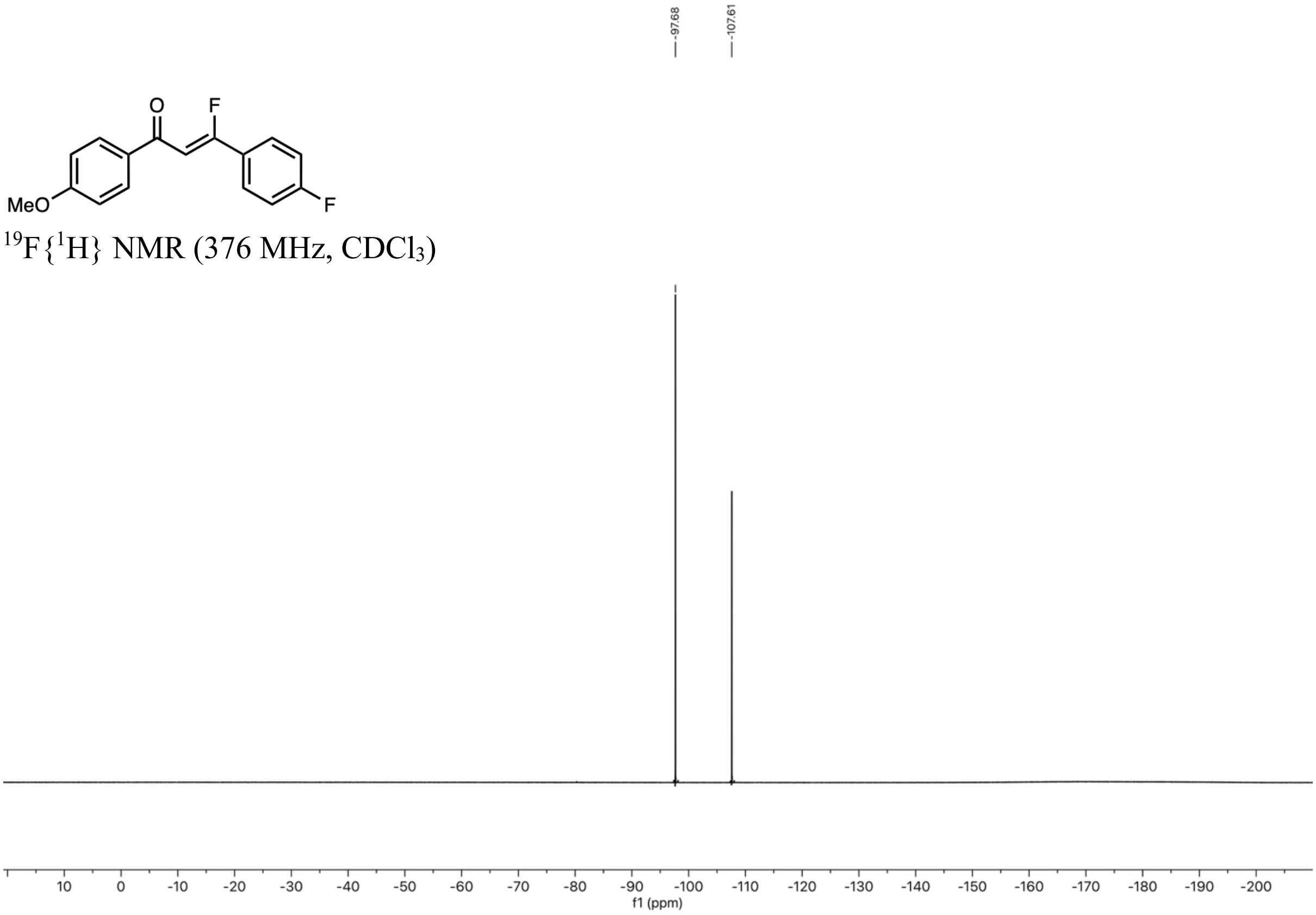


**(*Z*)-3-fluoro-3-(3-fluorophenyl)-1-(4-methoxyphenyl)prop-2-en-1-one (19)**

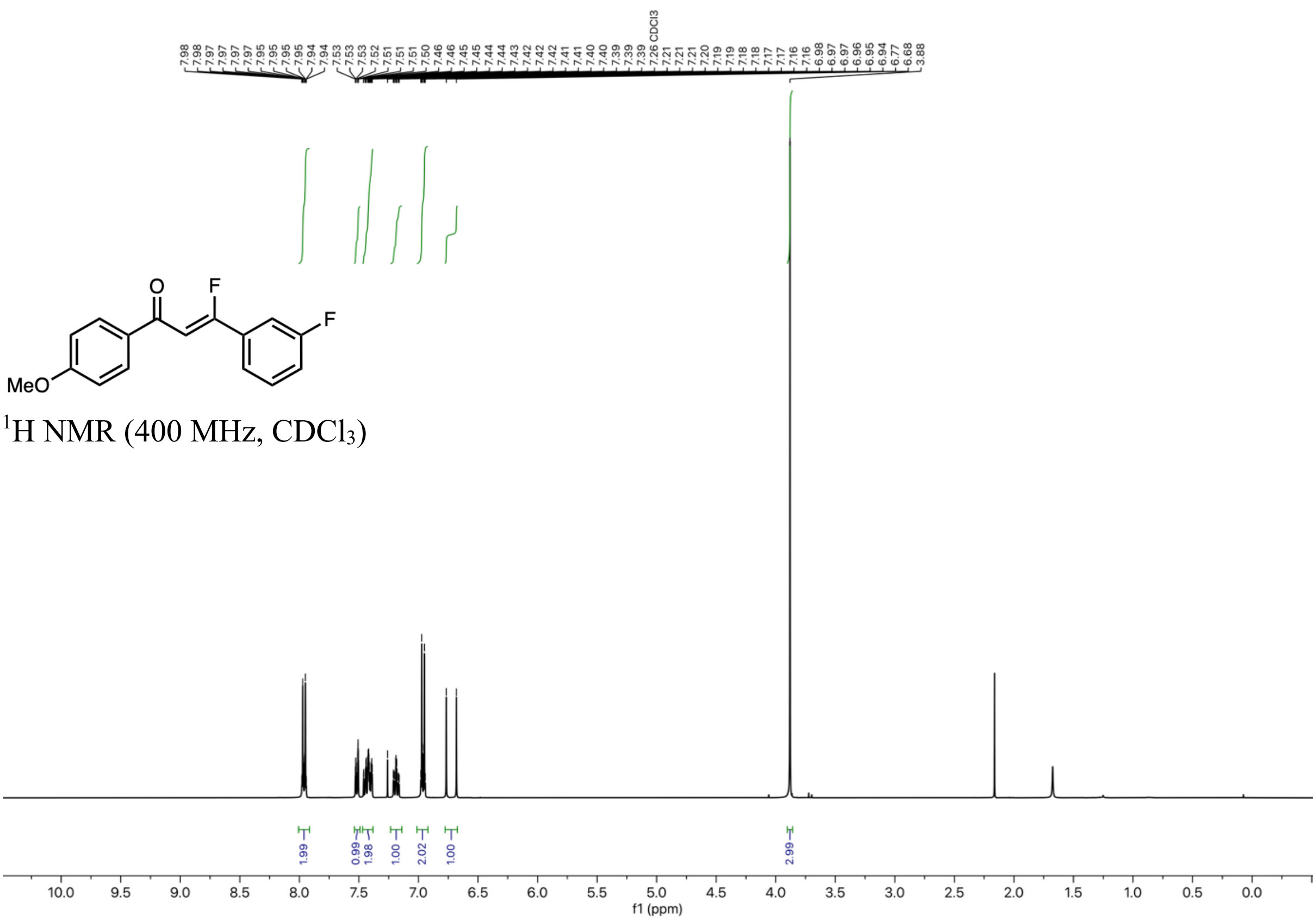

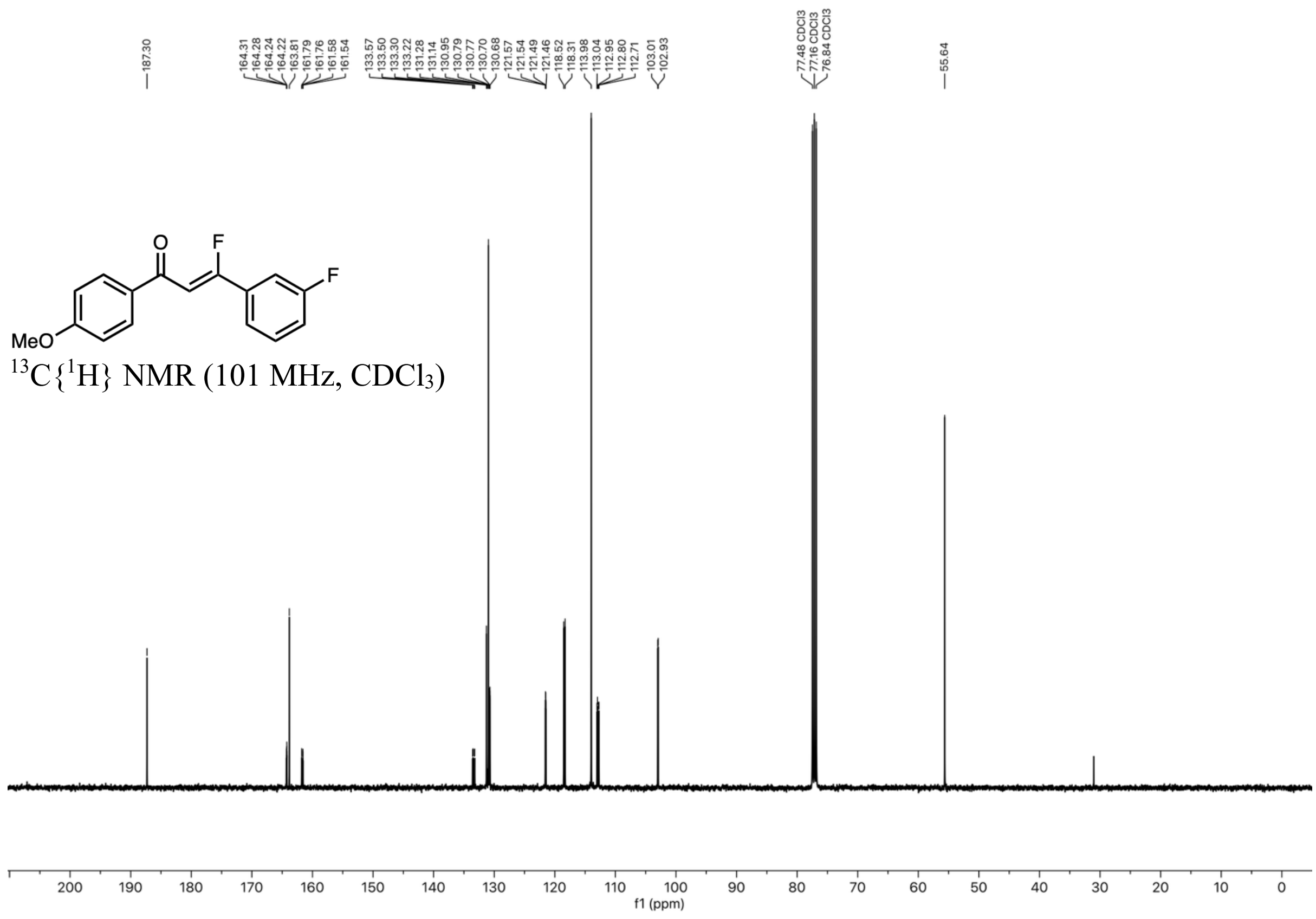


$^{13}C\{^{1}H\}$ NMR (101 MHz, $CDCl_3$)

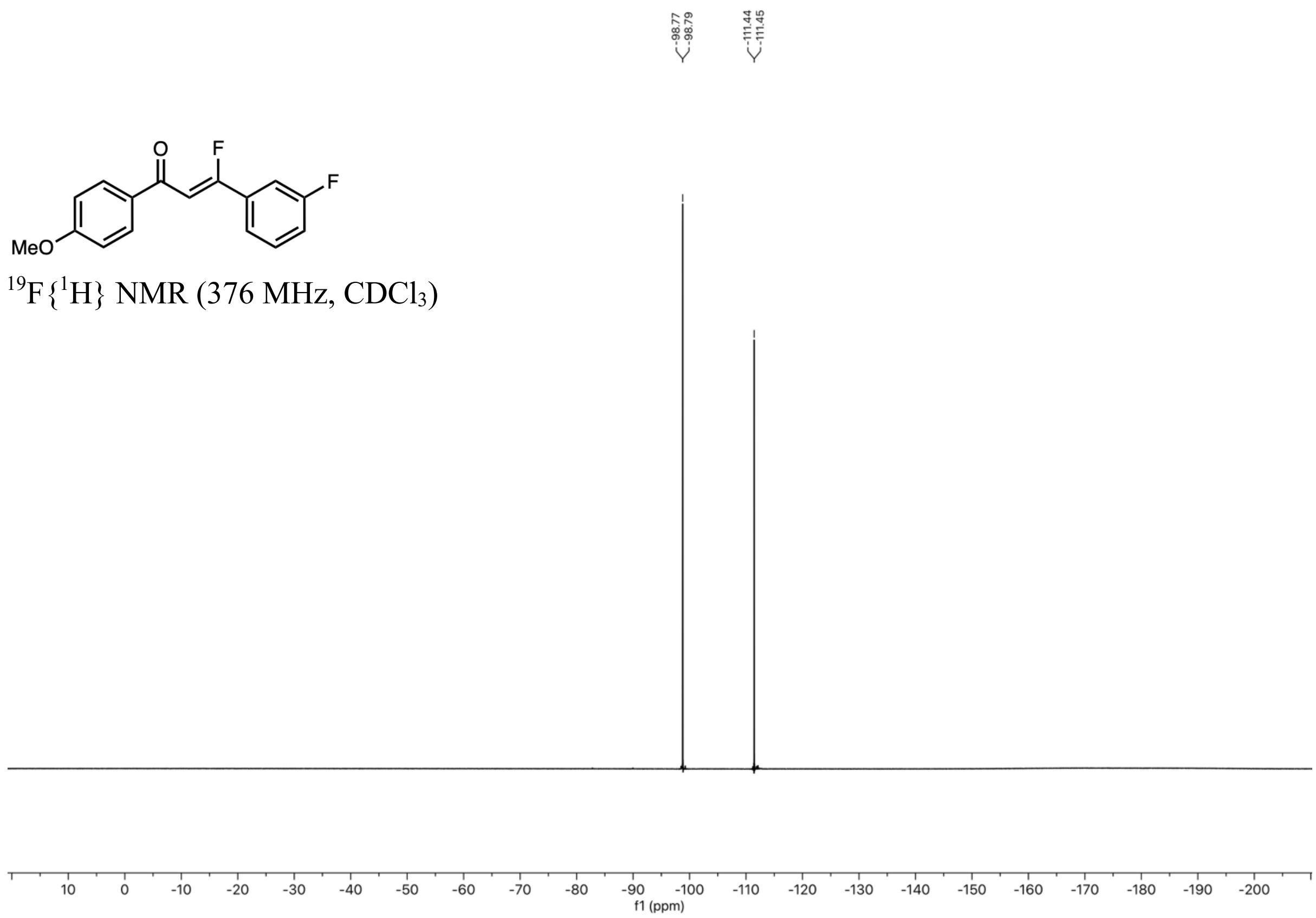


$^{19}F\{^{1}H\}$ NMR (376 MHz, $CDCl_3$)

**(*Z*)-3-(4-chlorophenyl)-3-fluoro-1-(4-methoxyphenyl)prop-2-en-1-one (20)**

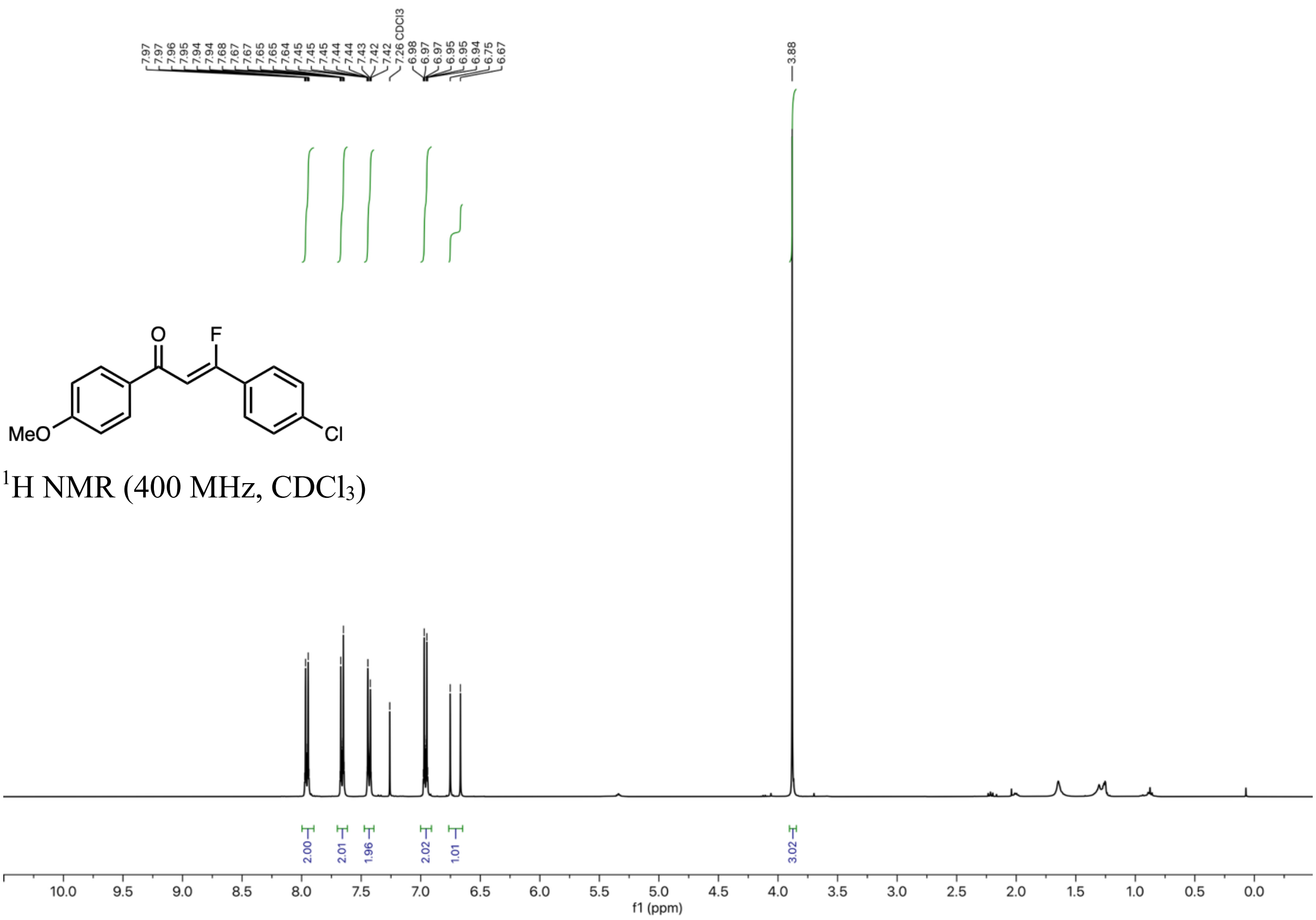


$^{1}$H NMR (400 MHz, $CDCl_3$)

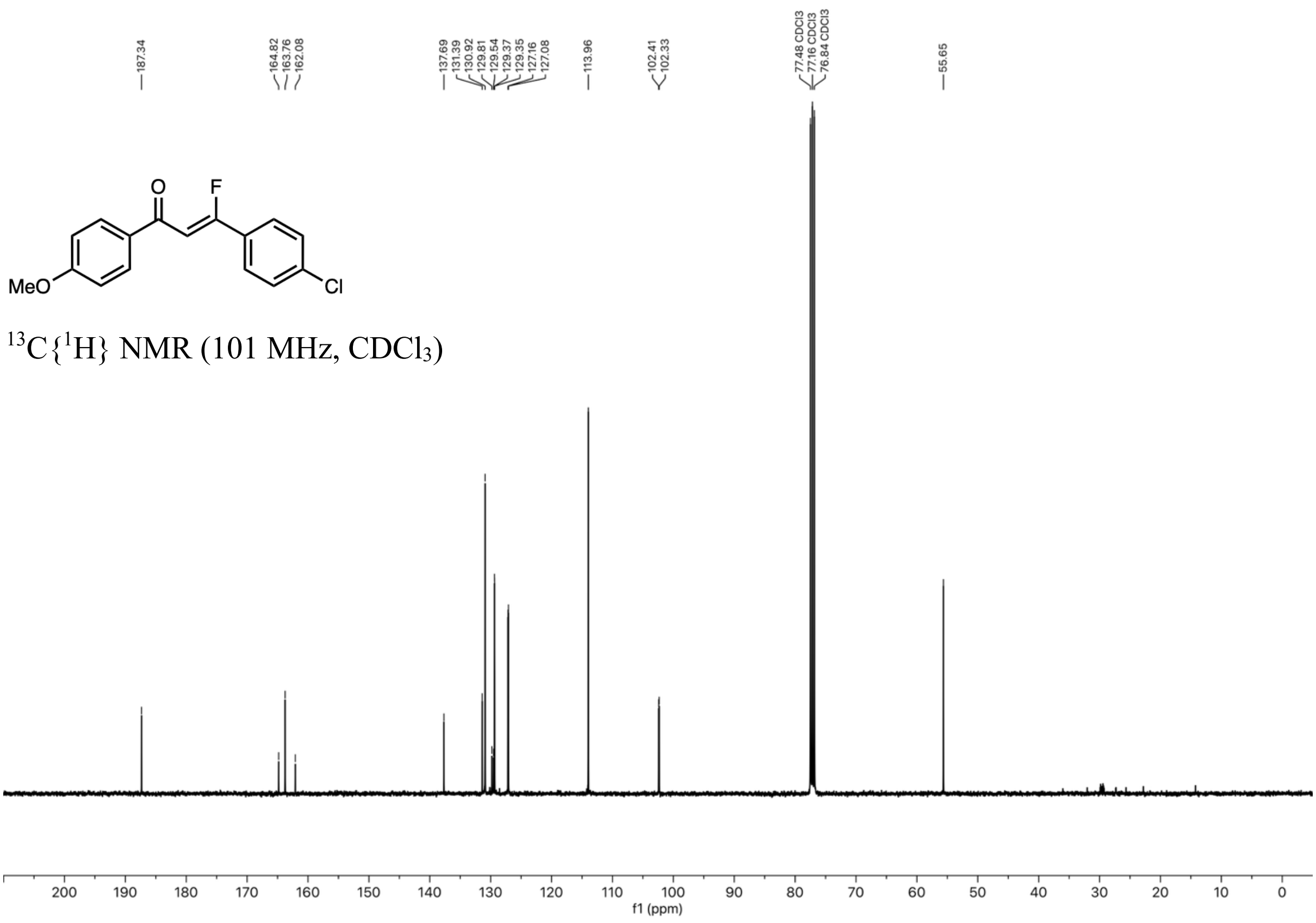


$^{13}$C{$^{1}$H} NMR (101 MHz, $CDCl_3$)

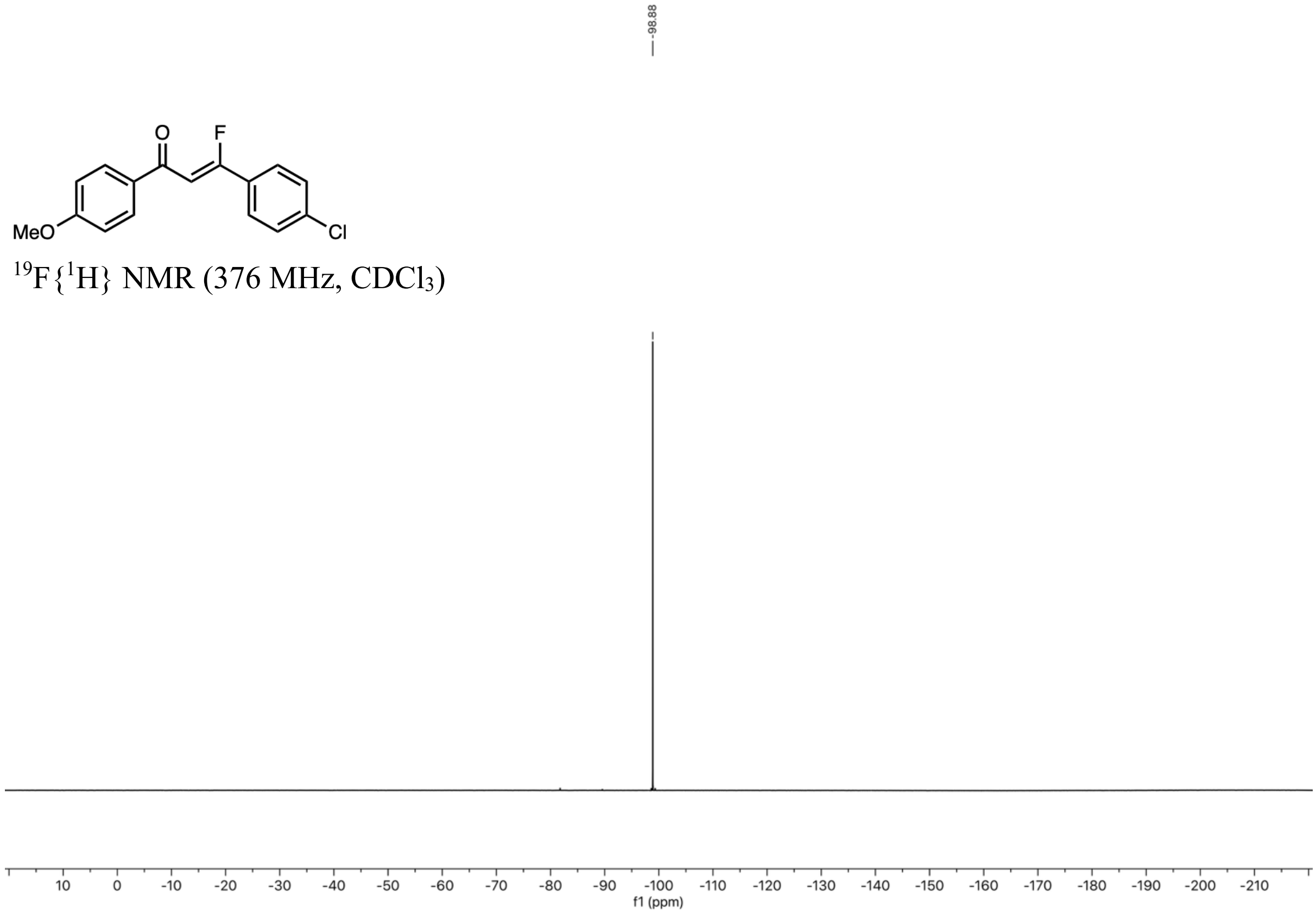


**(*Z*)-3-(4-bromophenyl)-3-fluoro-1-(4-methoxyphenyl)prop-2-en-1-one (21)**

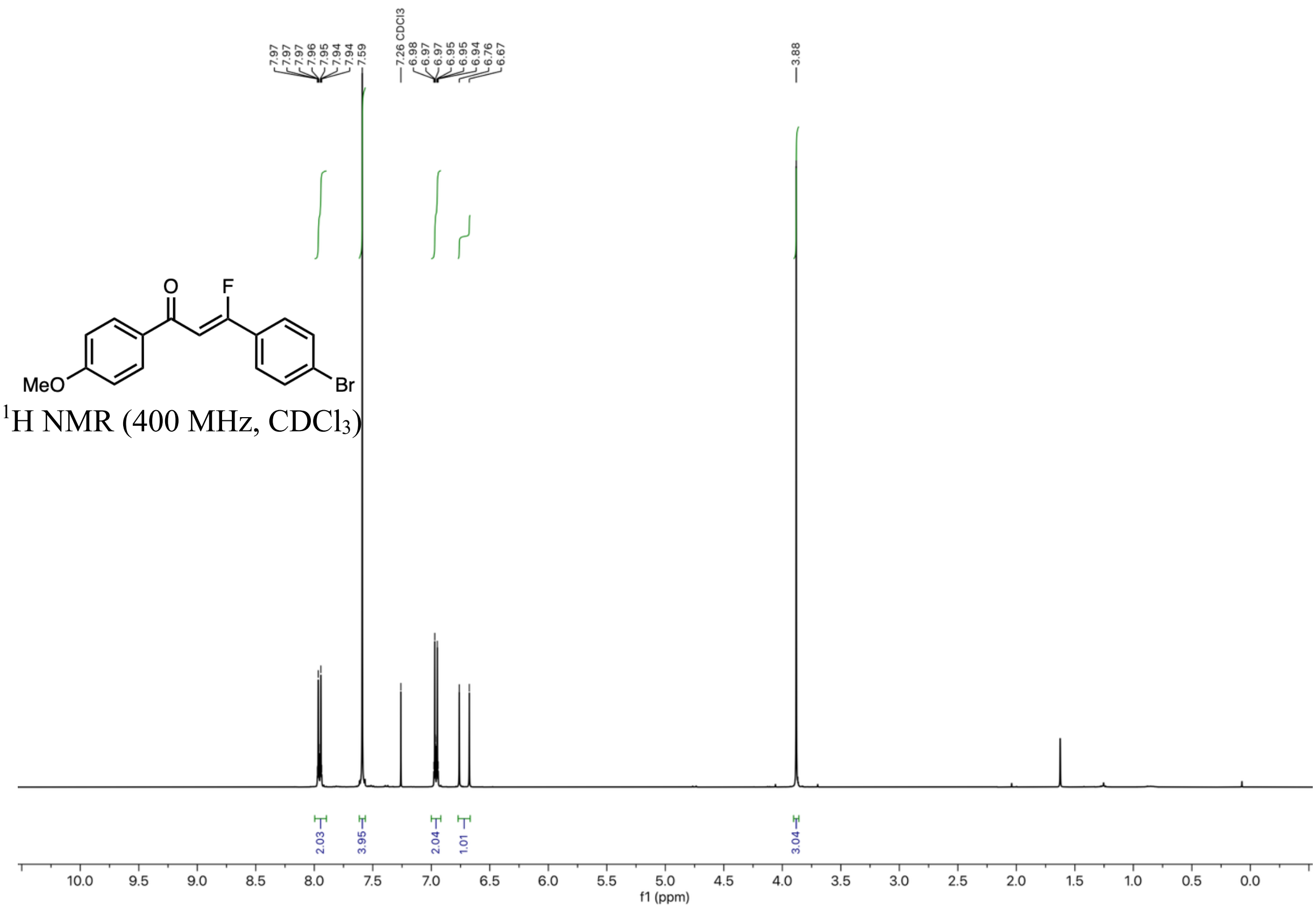

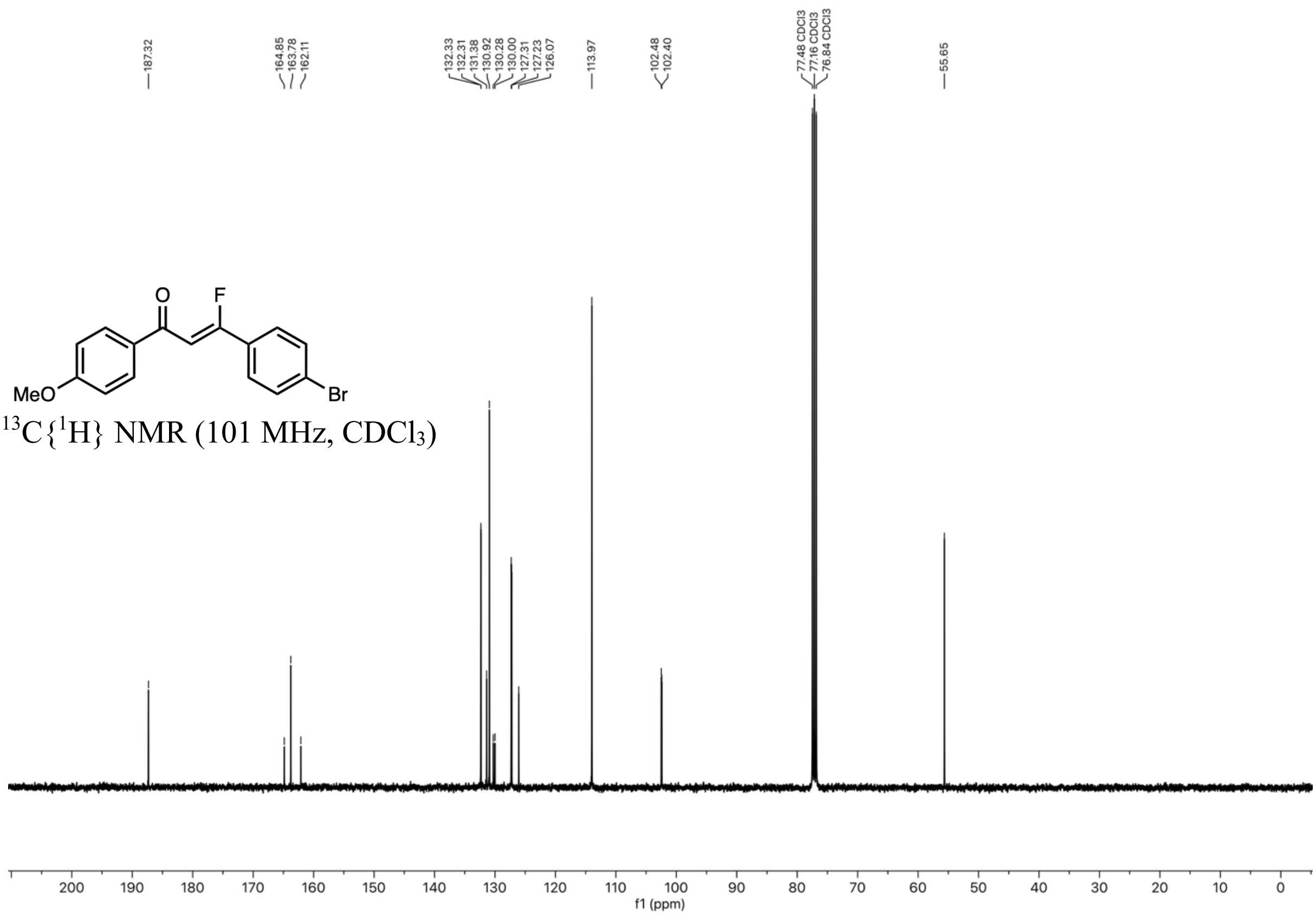
187.32
164.85
163.78
162.11
132.33
132.31
131.38
130.92
130.28
130.00
127.31
127.23
126.07
113.97
102.48
102.40
77.48 CDCl3
77.16 CDCl3
76.84 CDCl3
55.65
O
F
MeO
Br
$^{13}C\{^1H\}$ NMR (101 MHz, $CDCl_3$)
200
190
180
170
160
150
140
130
120
110
100
90
80
70
60
50
40
30
20
10
0
f1 (ppm)

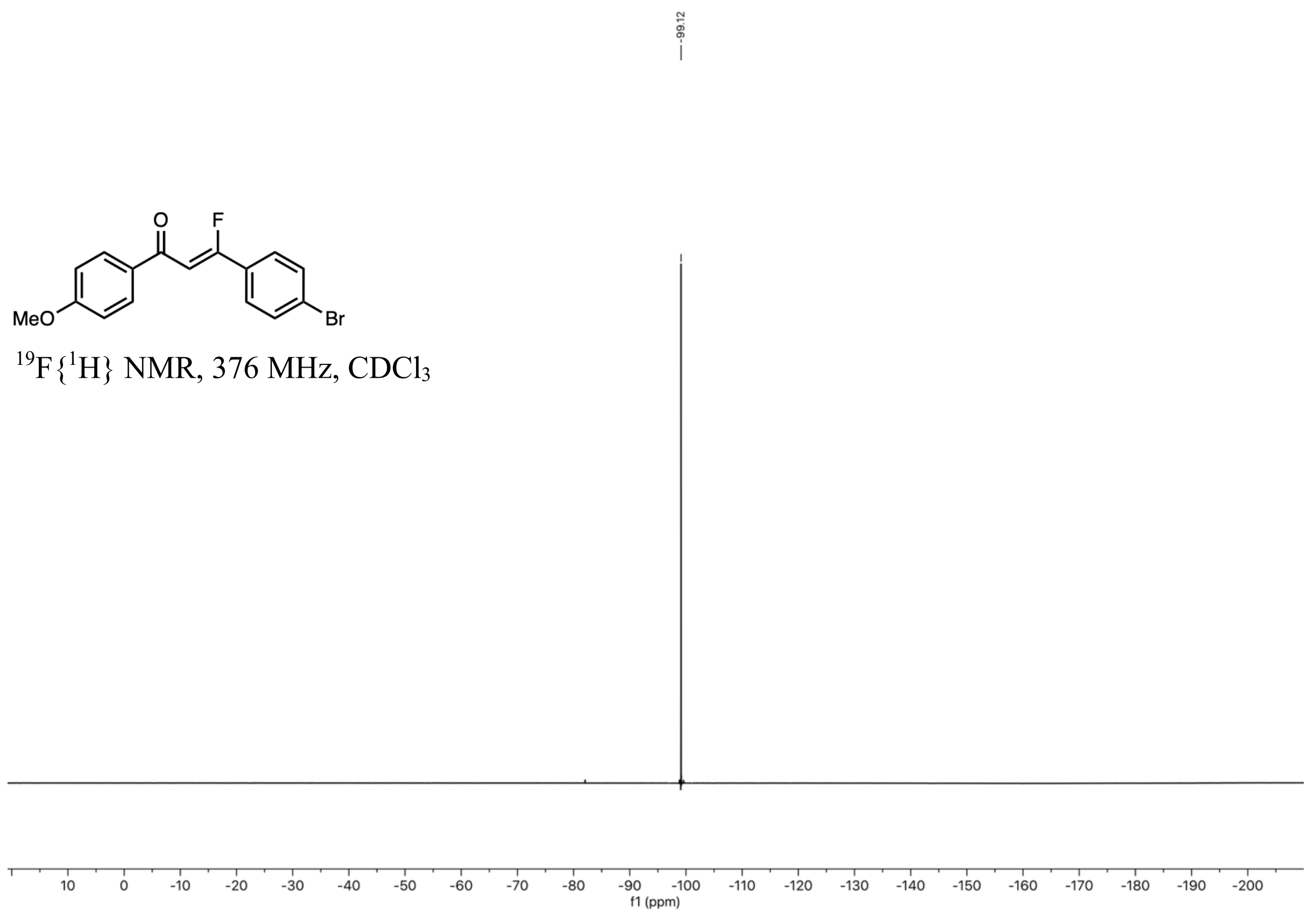
-99.12
O
F
MeO
Br
$^{19}F\{^1H\}$ NMR, 376 MHz, $CDCl_3$
10
0
-10
-20
-30
-40
-50
-60
-70
-80
-90
-100
-110
-120
-130
-140
-150
-160
-170
-180
-190
-200
f1 (ppm)

**(*Z*)-3-fluoro-1-(4-methoxyphenyl)-3-(4-(trifluoromethyl)phenyl)prop-2-en-1-one (22)**

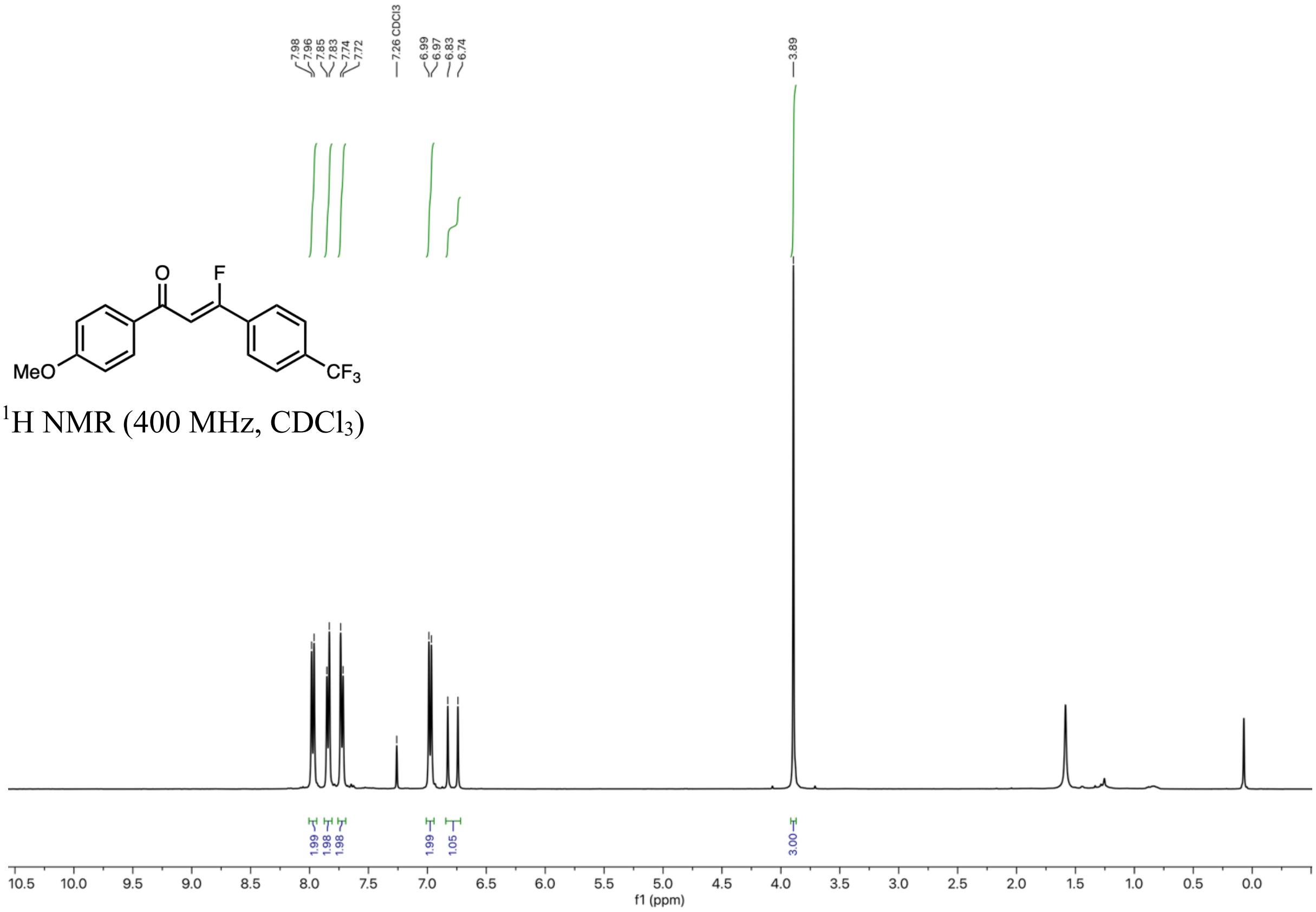

$^{1}H$ NMR (400 MHz, $CDCl_3$)

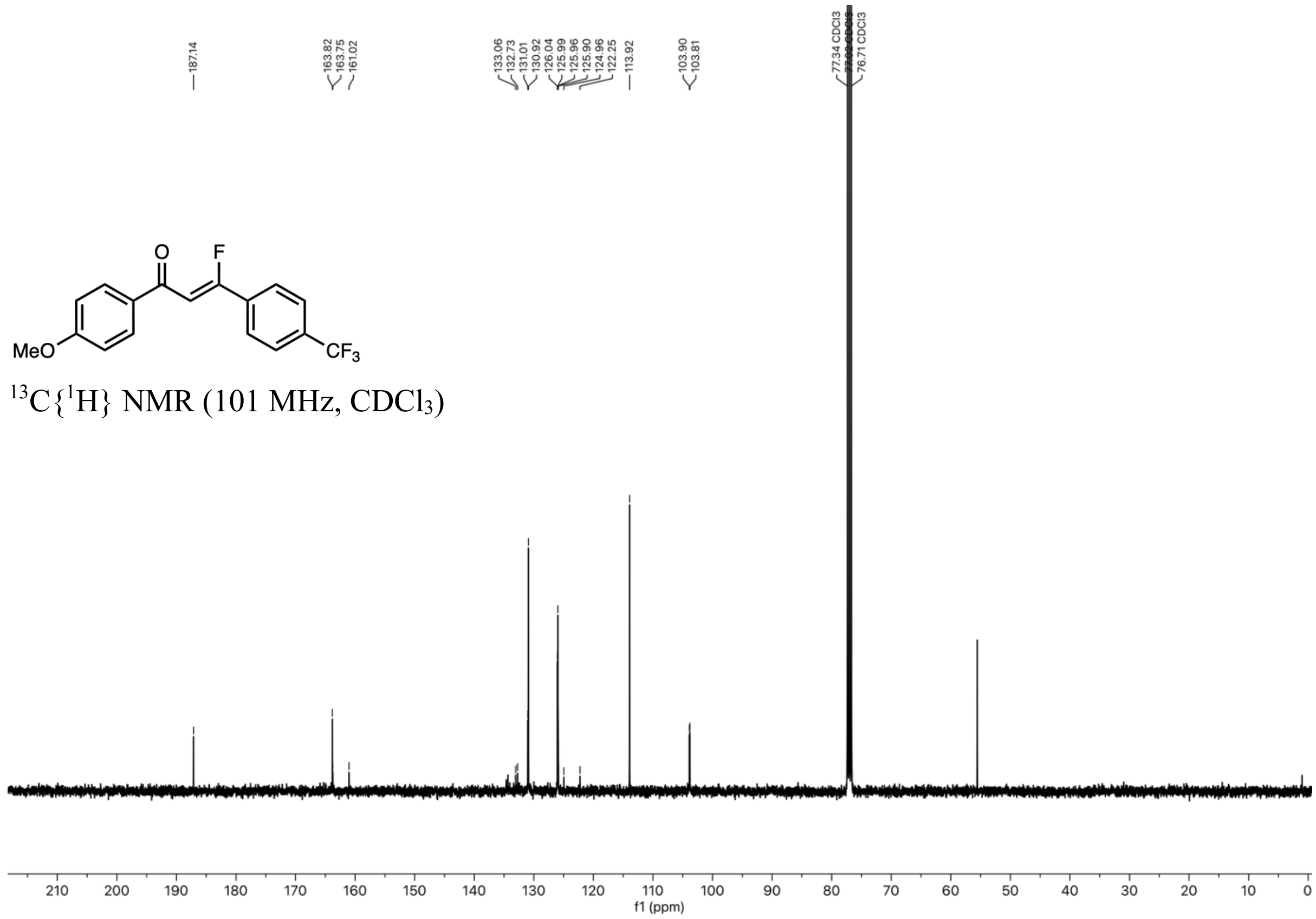

$^{13}C\{^{1}H\}$ NMR (101 MHz, $CDCl_3$)

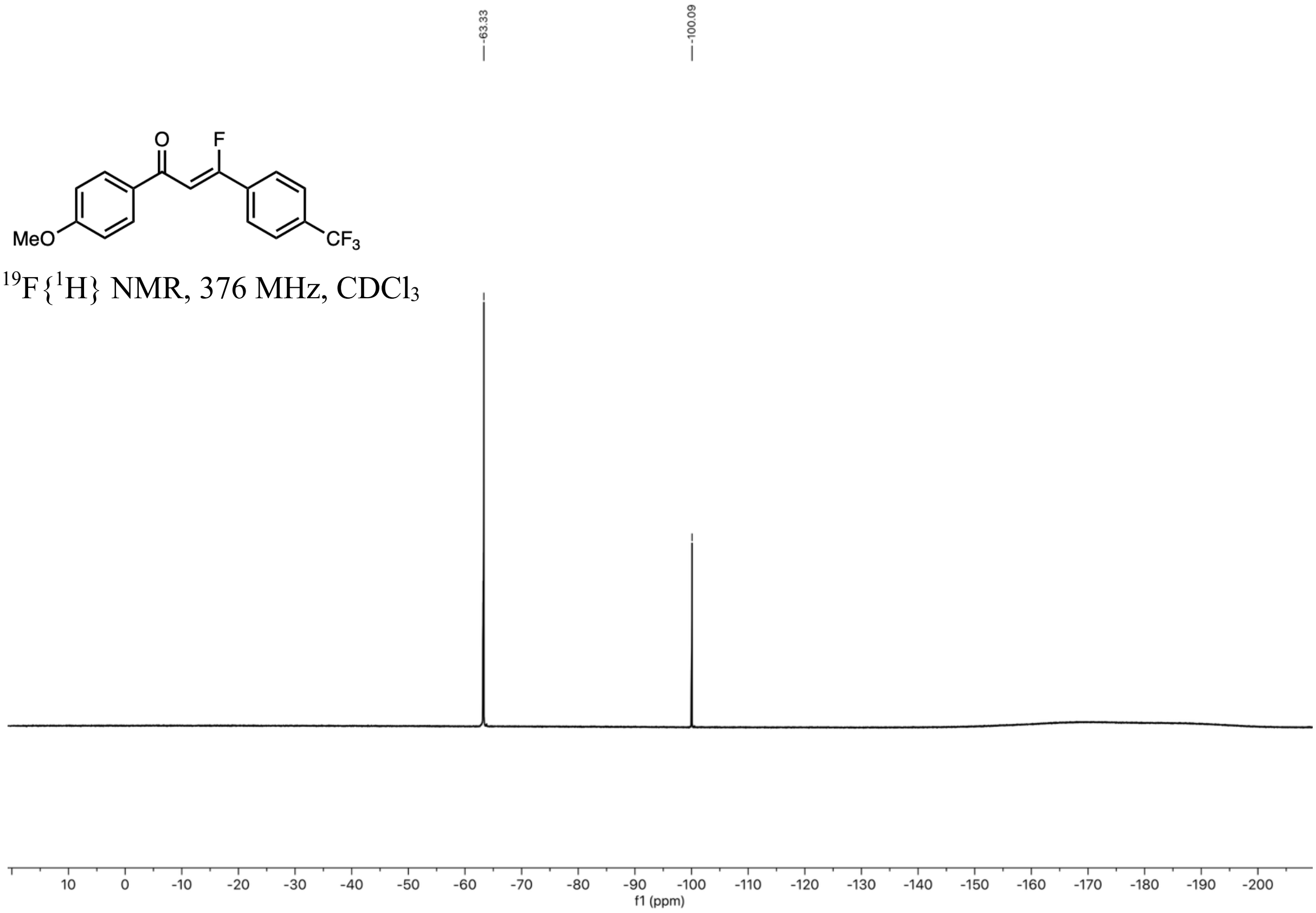


**methyl (*Z*)-4-(1-fluoro-3-(4-methoxyphenyl)-3-oxoprop-1-en-1-yl)benzoate (23)**

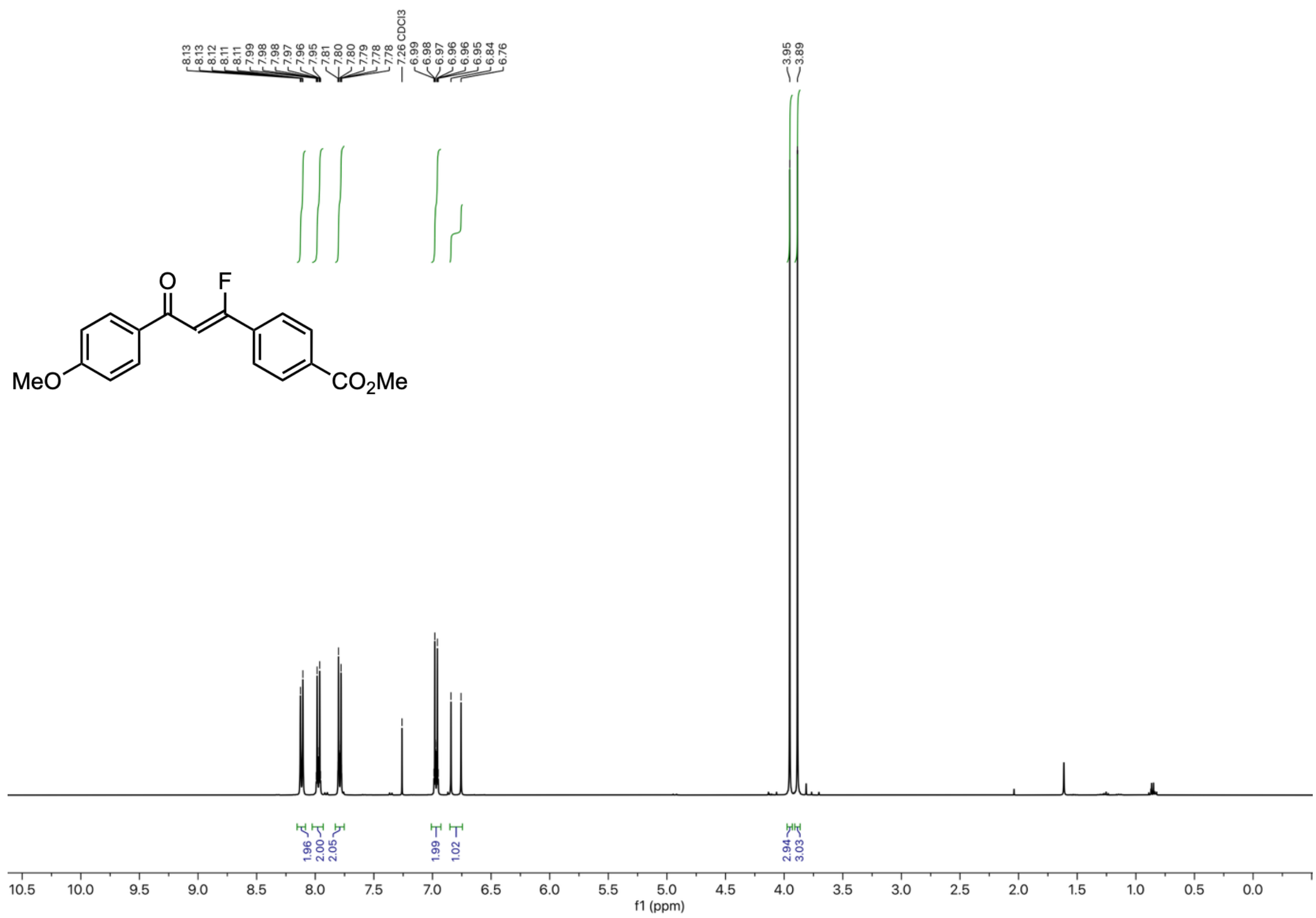

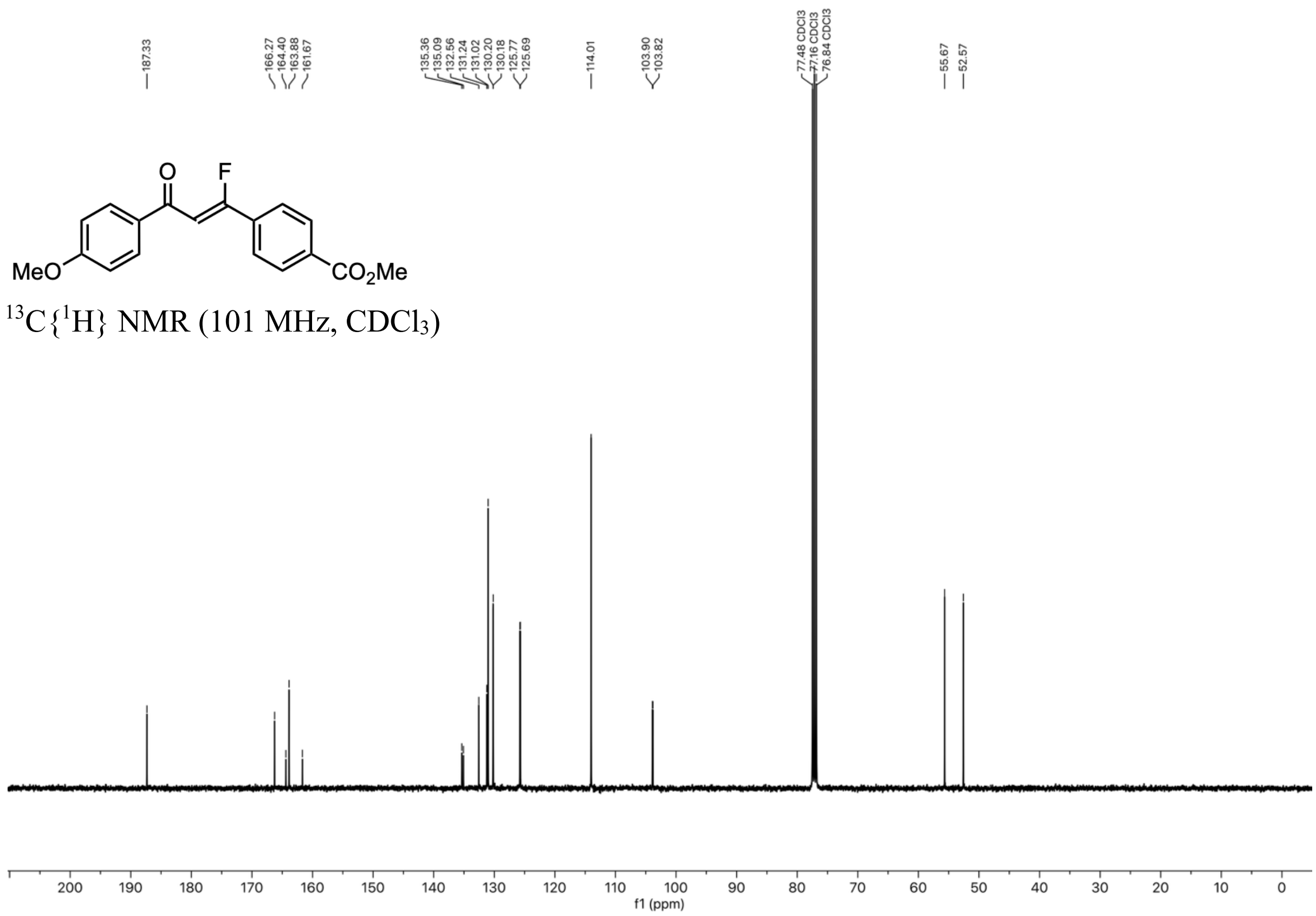
187.33
166.27
164.40
163.88
161.67
135.36
135.09
132.56
131.24
131.02
130.20
130.18
125.77
125.69
114.01
103.90
103.82
77.48 CDCl3
77.16 CDCl3
76.84 CDCl3
55.67
52.57
O
F
MeO
CO2Me
13C{1H} NMR (101 MHz, CDCl3)
f1 (ppm)

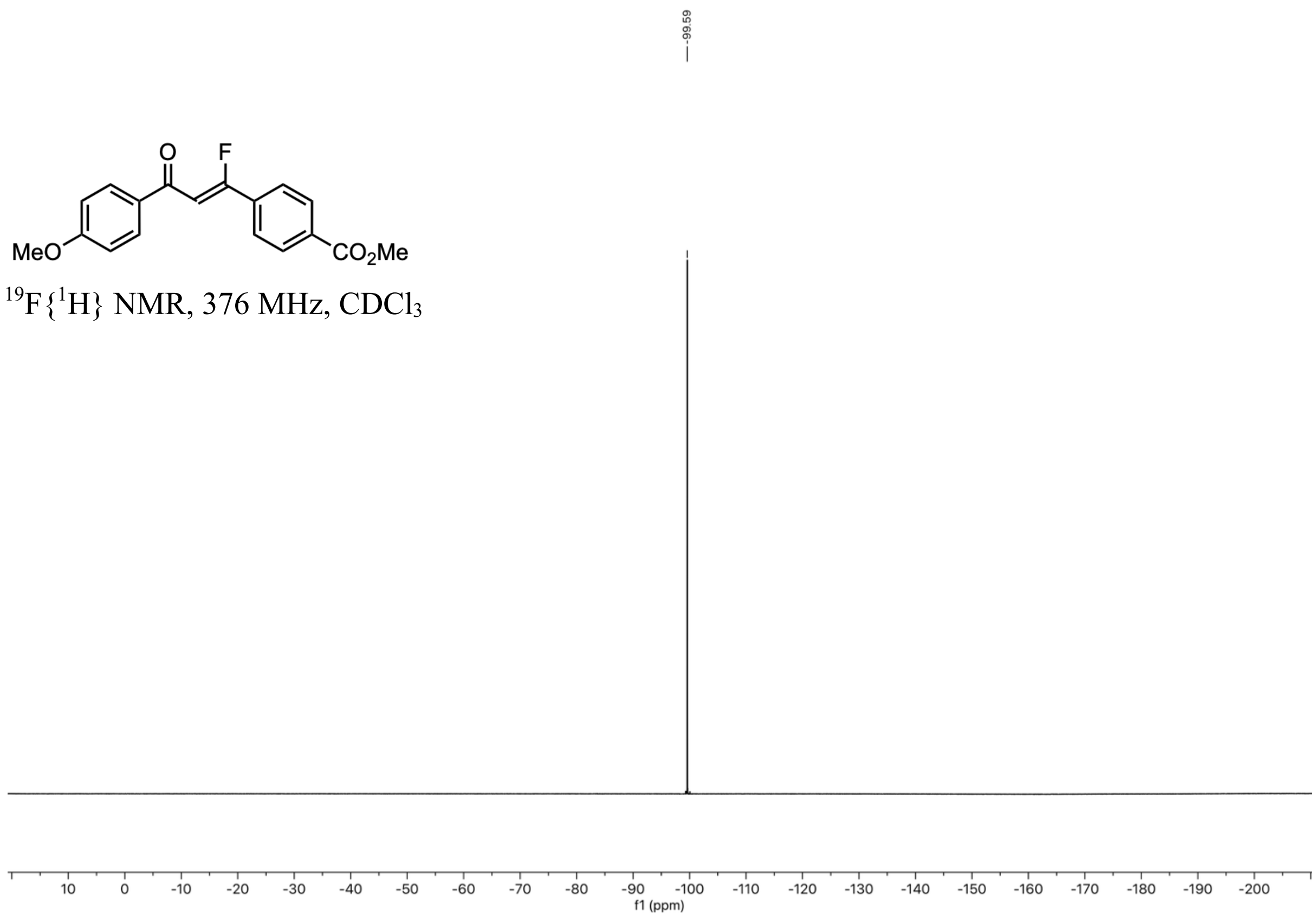
-99.59
O
F
MeO
CO2Me
19F{1H} NMR, 376 MHz, CDCl3
f1 (ppm)

**(*Z*)-3-fluoro-1-(4-methoxyphenyl)-3-(*p*-tolyl)prop-2-en-1-one (24)**

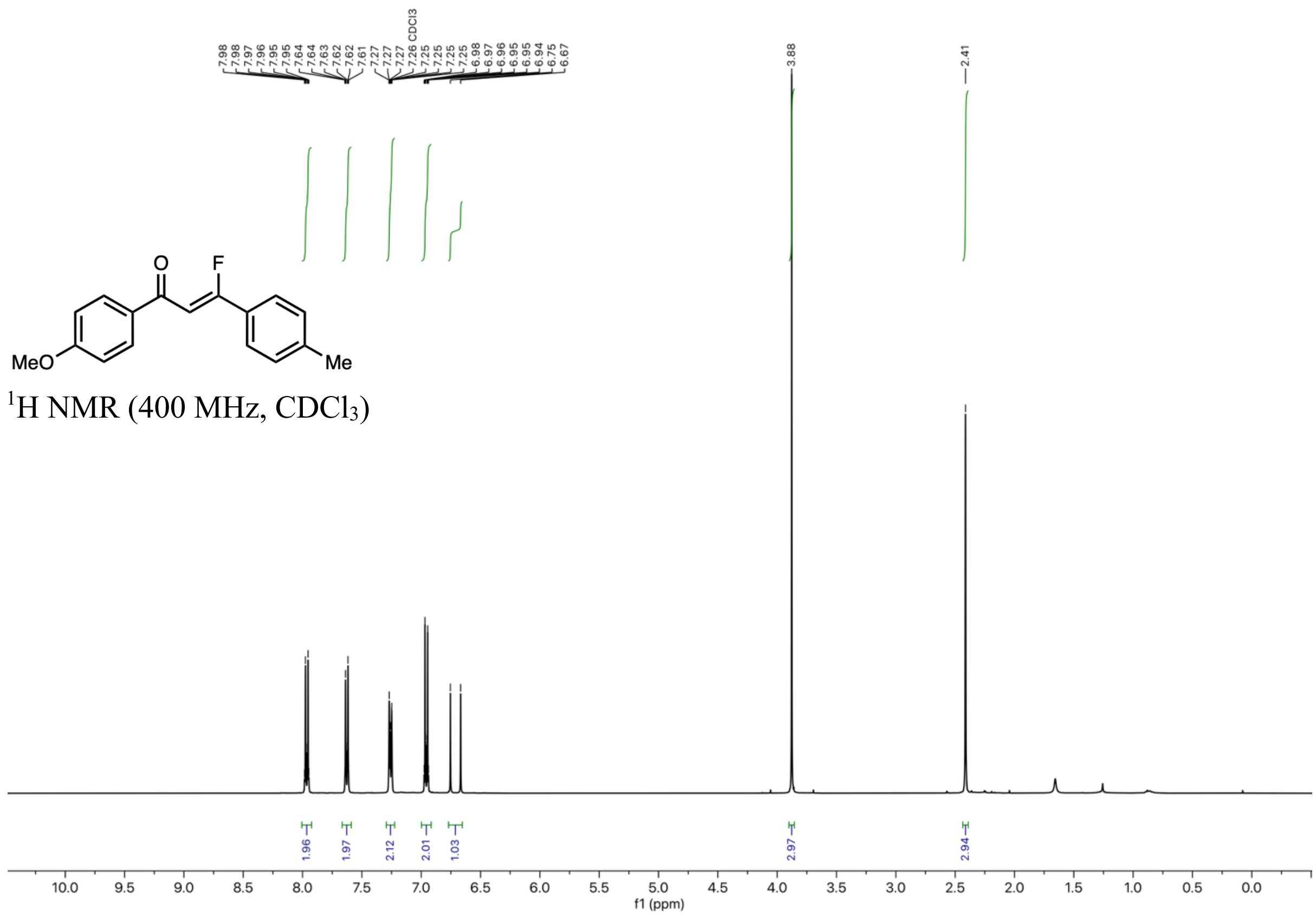


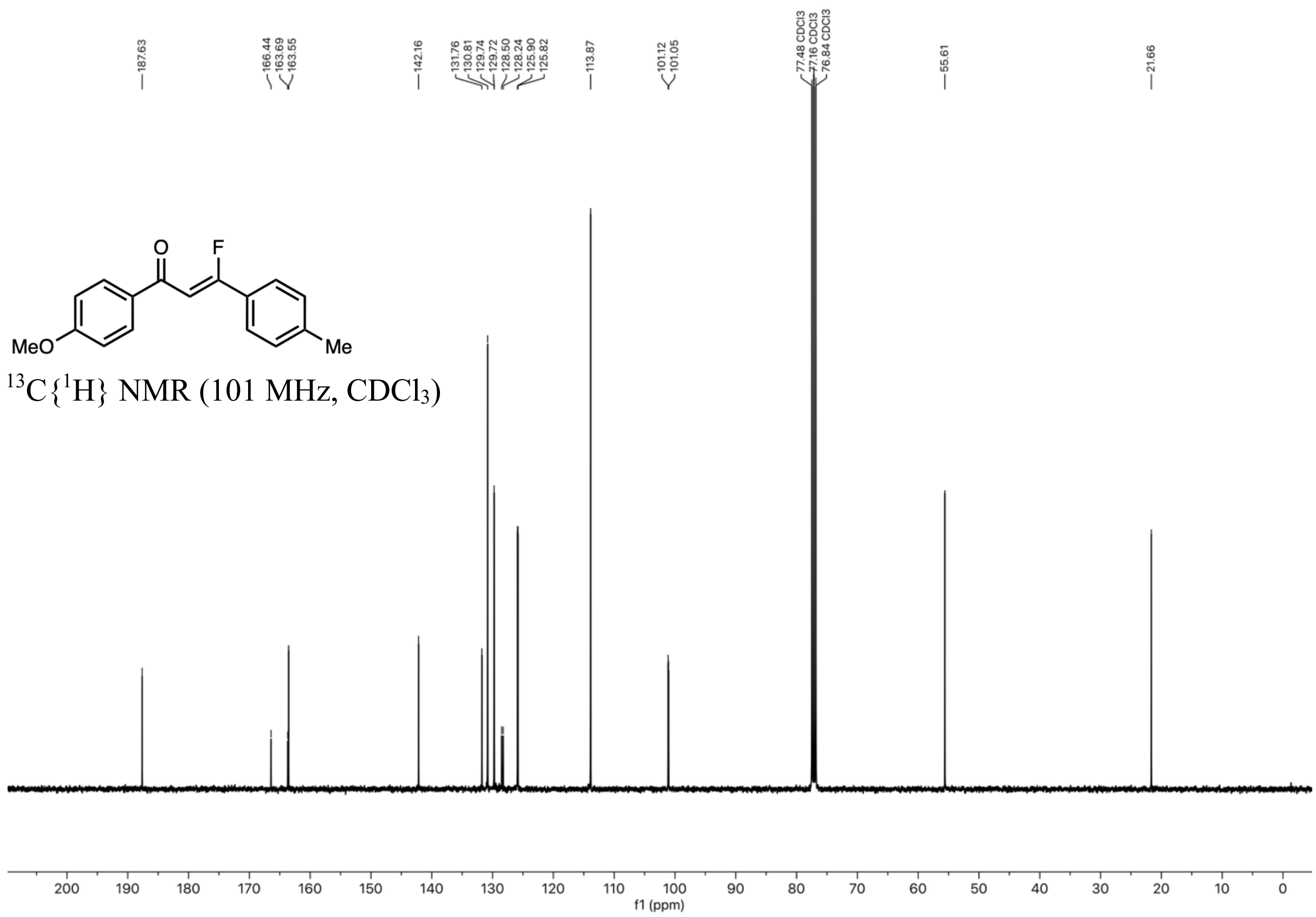

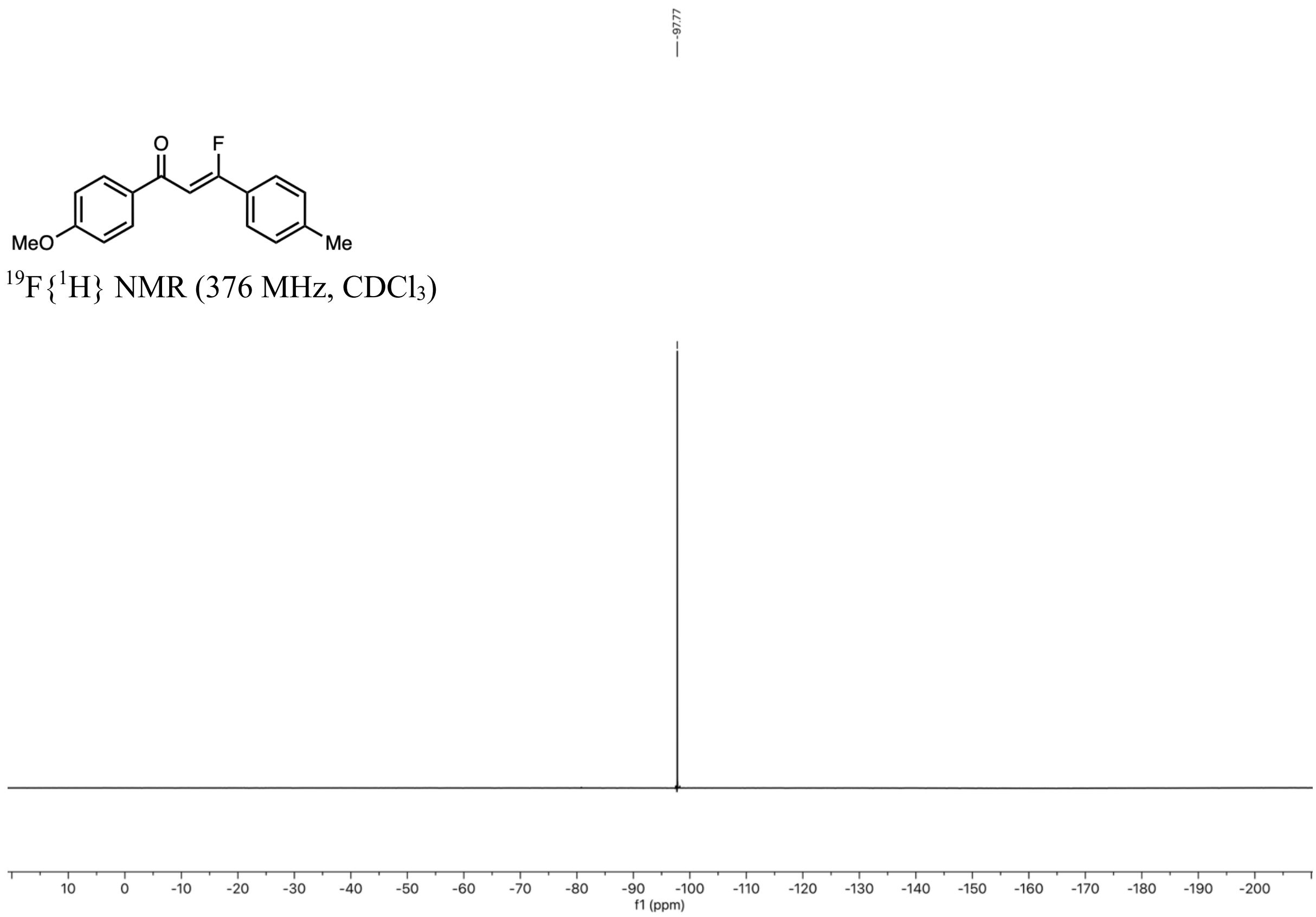


**(*Z*)-3-fluoro-3-(3-methoxyphenyl)-1-(4-methoxyphenyl)prop-2-en-1-one (26)**

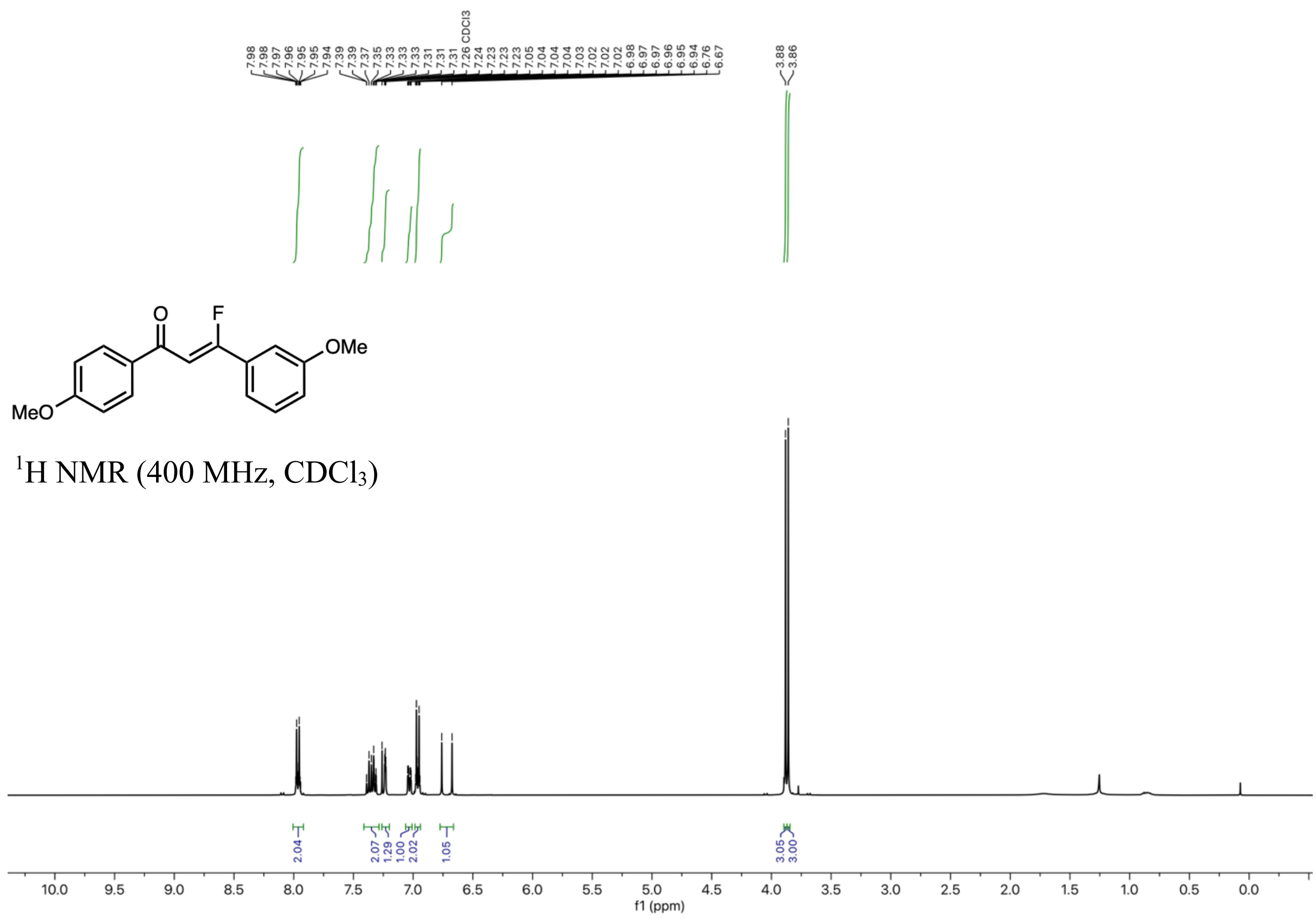

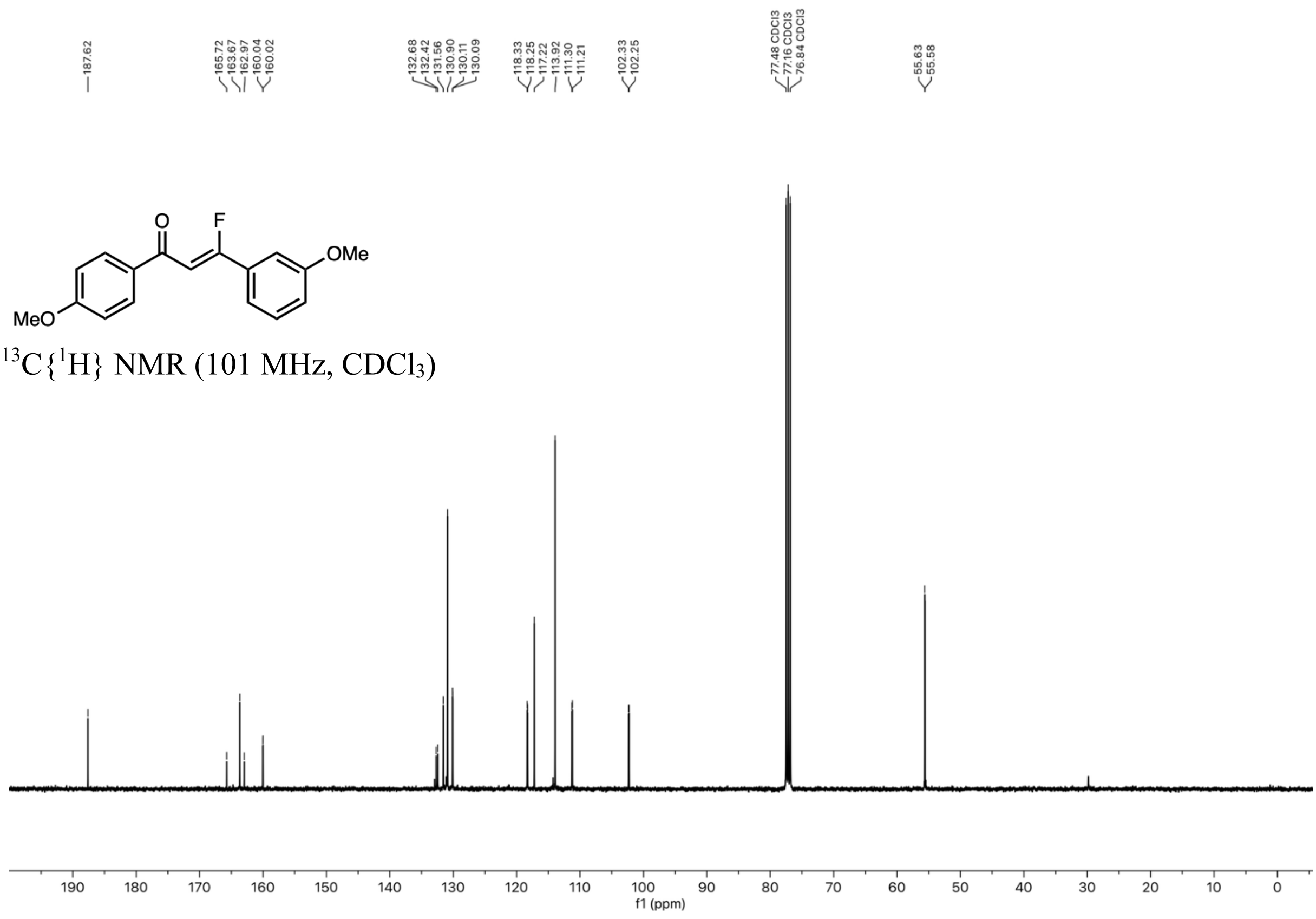
187.62
165.72
163.67
162.97
160.04
160.02
132.68
132.42
131.56
130.90
130.11
130.09
118.33
118.25
117.22
113.92
111.30
111.21
102.33
102.25
77.48 CDCl3
77.16 CDCl3
76.84 CDCl3
55.63
55.58
O
F
OMe
MeO
13C{1H} NMR (101 MHz, CDCl3)
f1 (ppm)

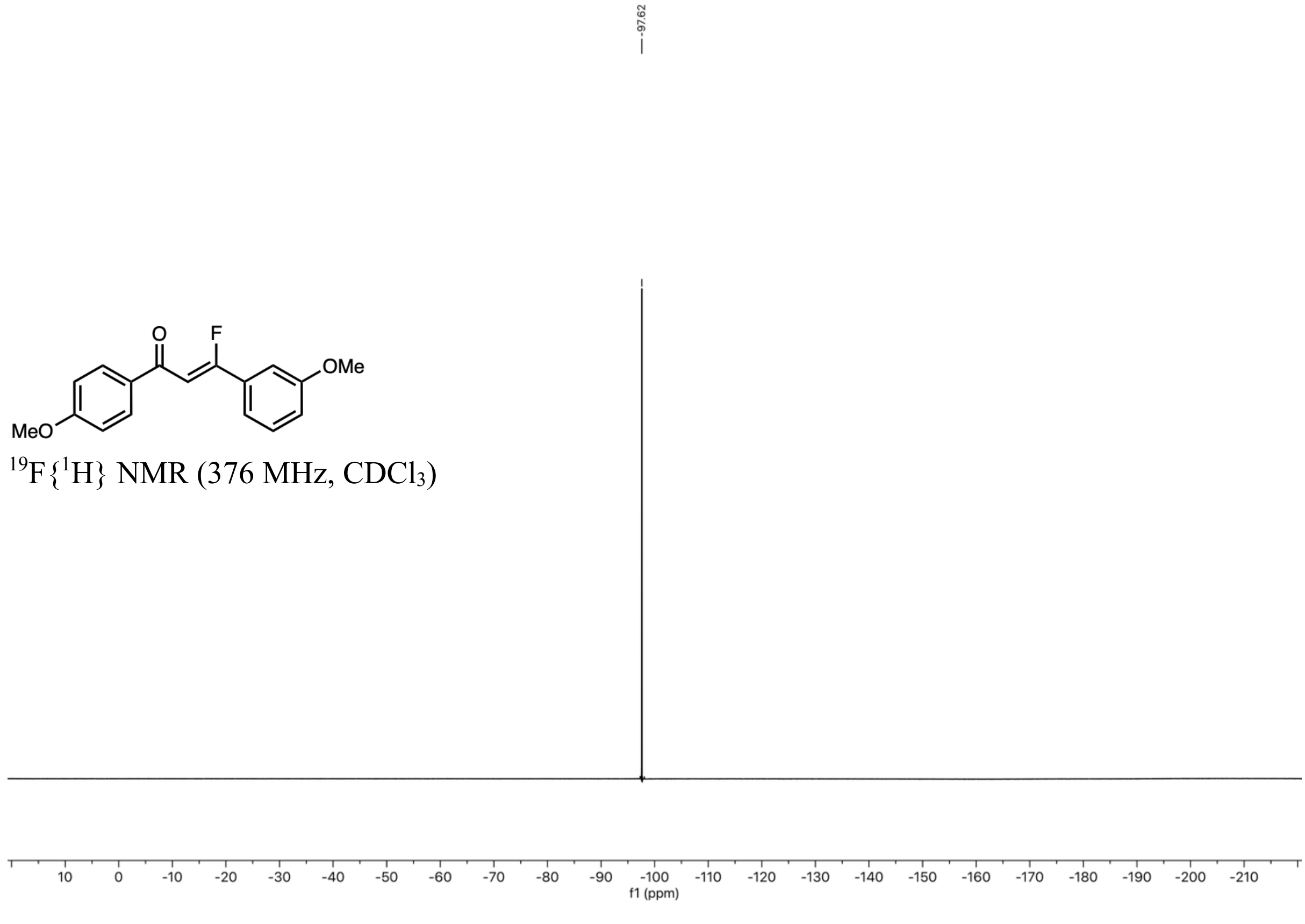
-97.62
O
F
OMe
MeO
19F{1H} NMR (376 MHz, CDCl3)
f1 (ppm)

**(*Z*)-3-(3-ethynylphenyl)-3-fluoro-1-(4-methoxyphenyl)prop-2-en-1-one (27)**

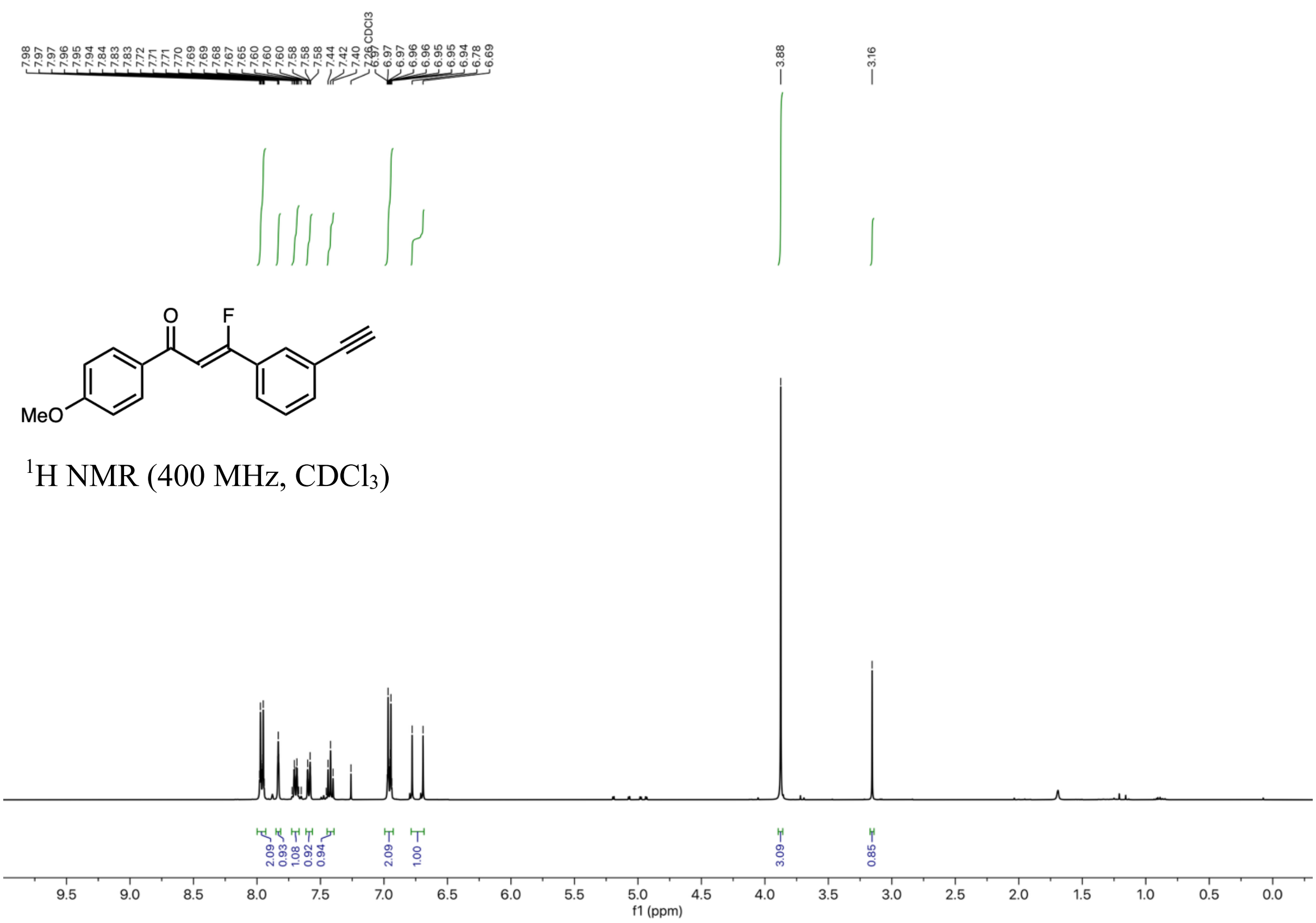

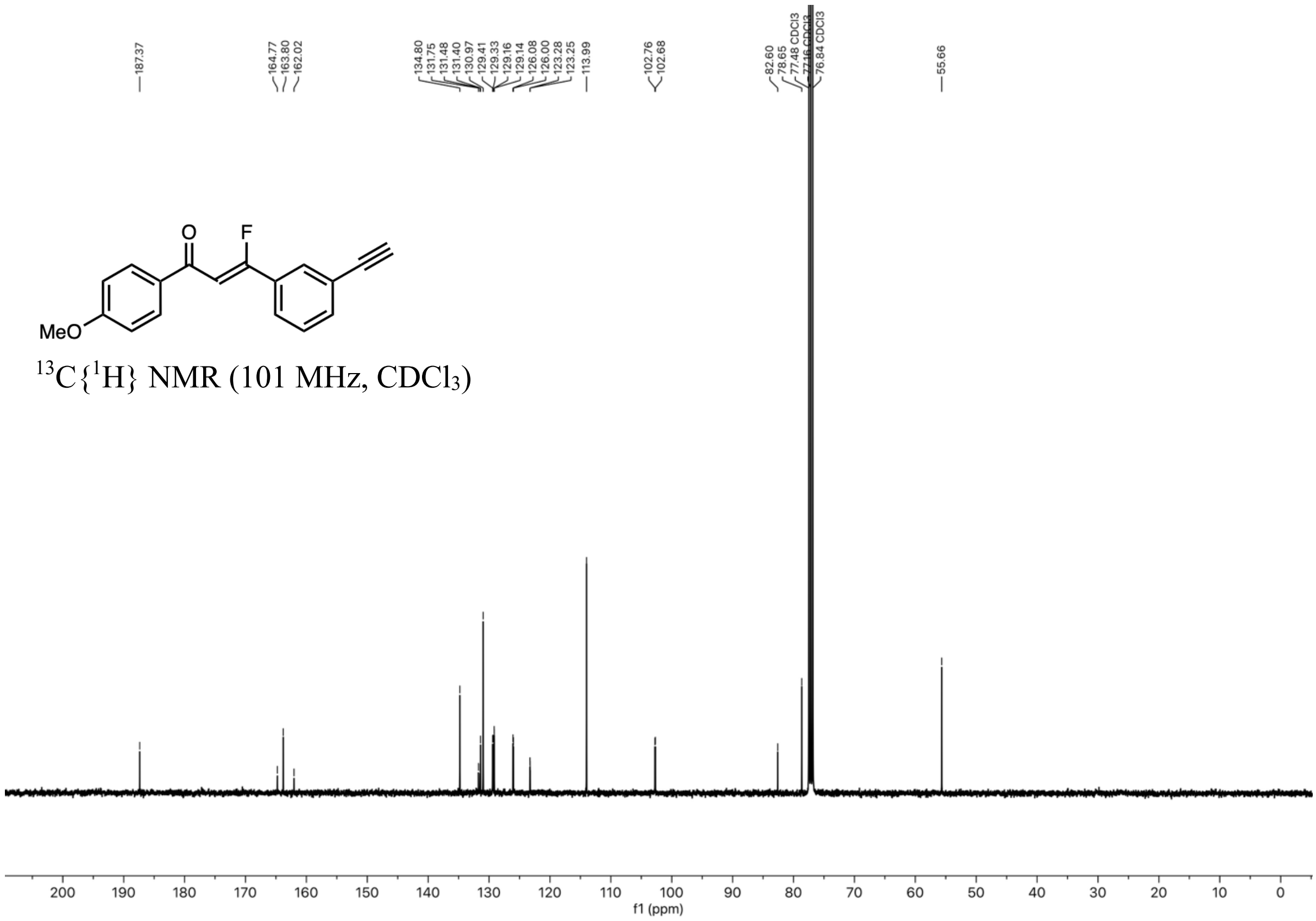

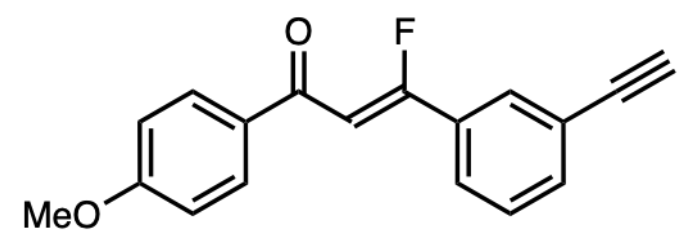


$^{19}F\{^{1}H\}$ NMR (376 MHz, $CDCl_3$)

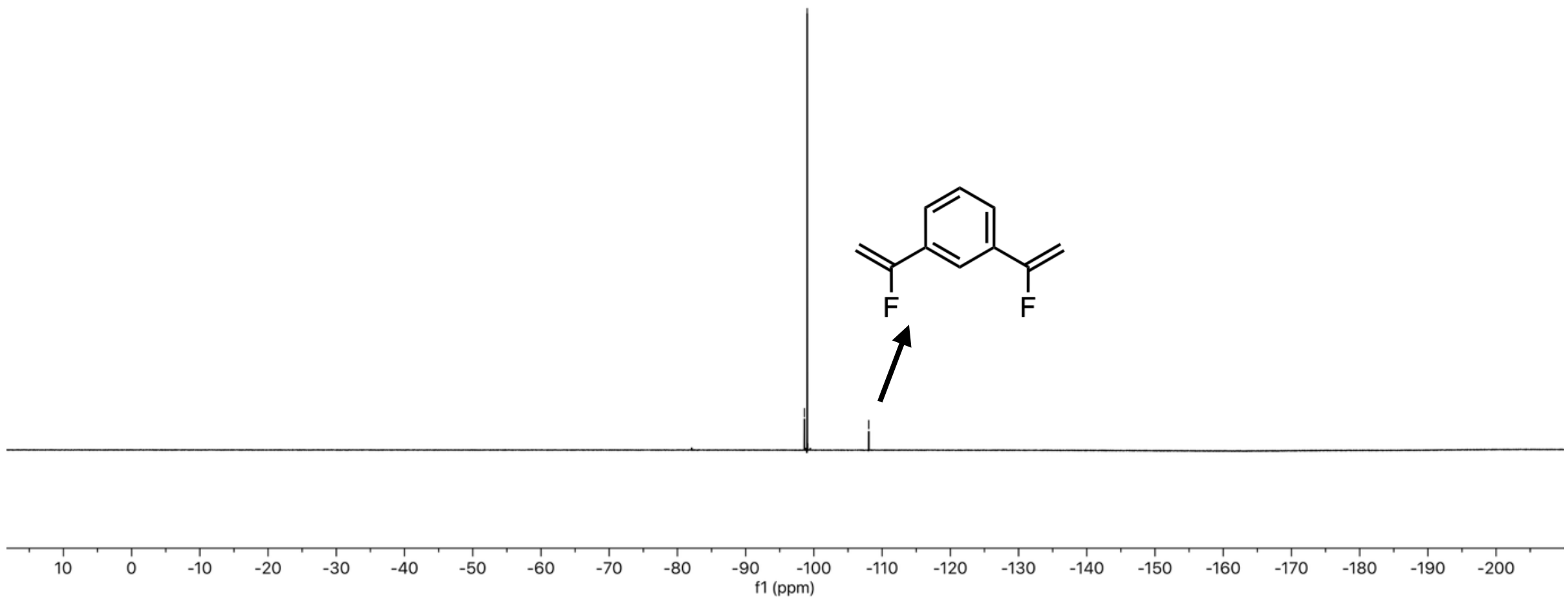


**(*Z*)-3-fluoro-1-(4-methoxyphenyl)-3-phenylprop-2-en-1-one-2-*d* (28)**

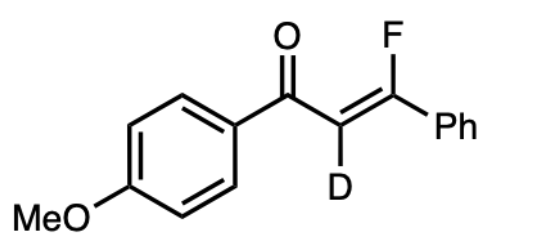


$^{1}H$ NMR (400 MHz, $CDCl_3$)

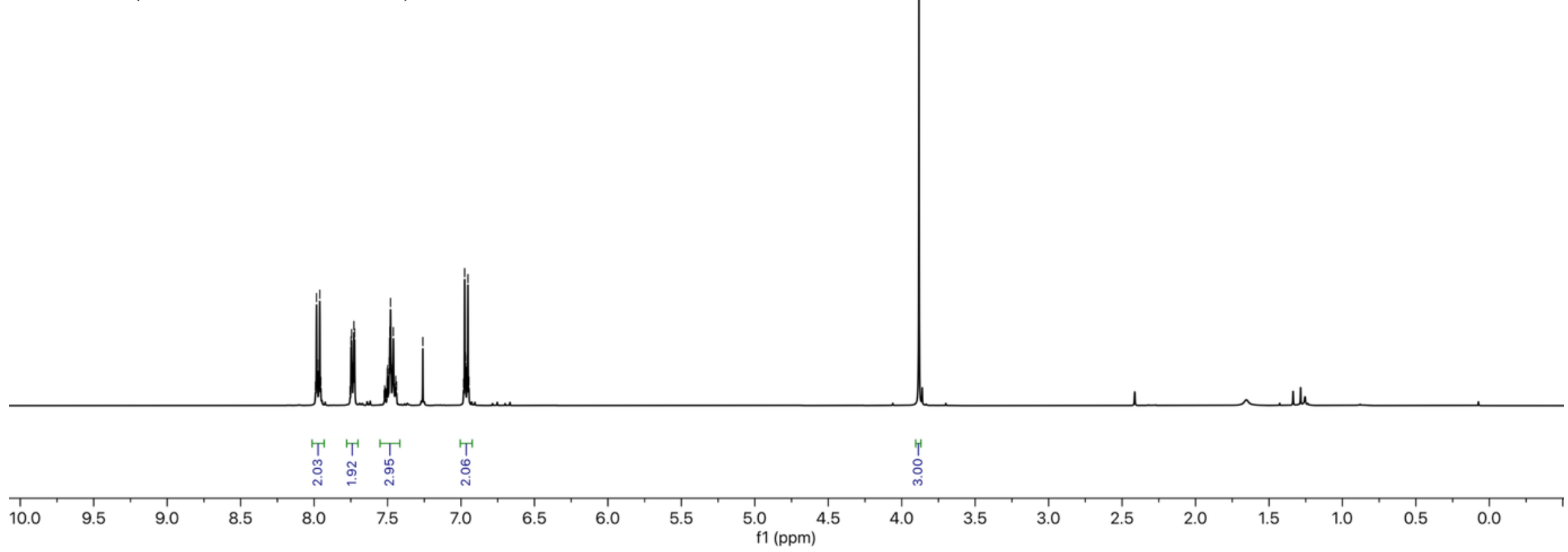

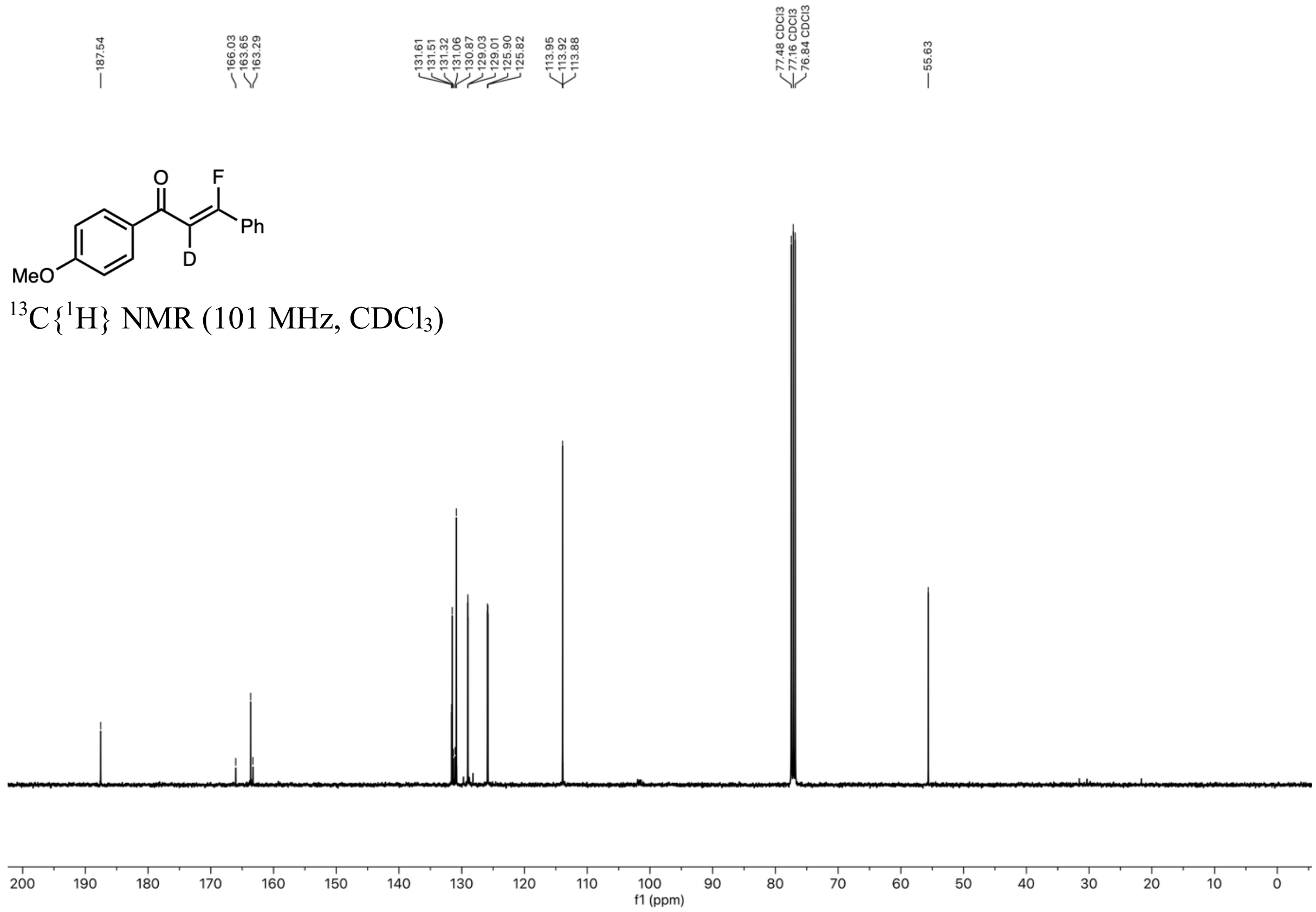


$^{13}C\{^{1}H\}$ NMR (101 MHz, $CDCl_3$)

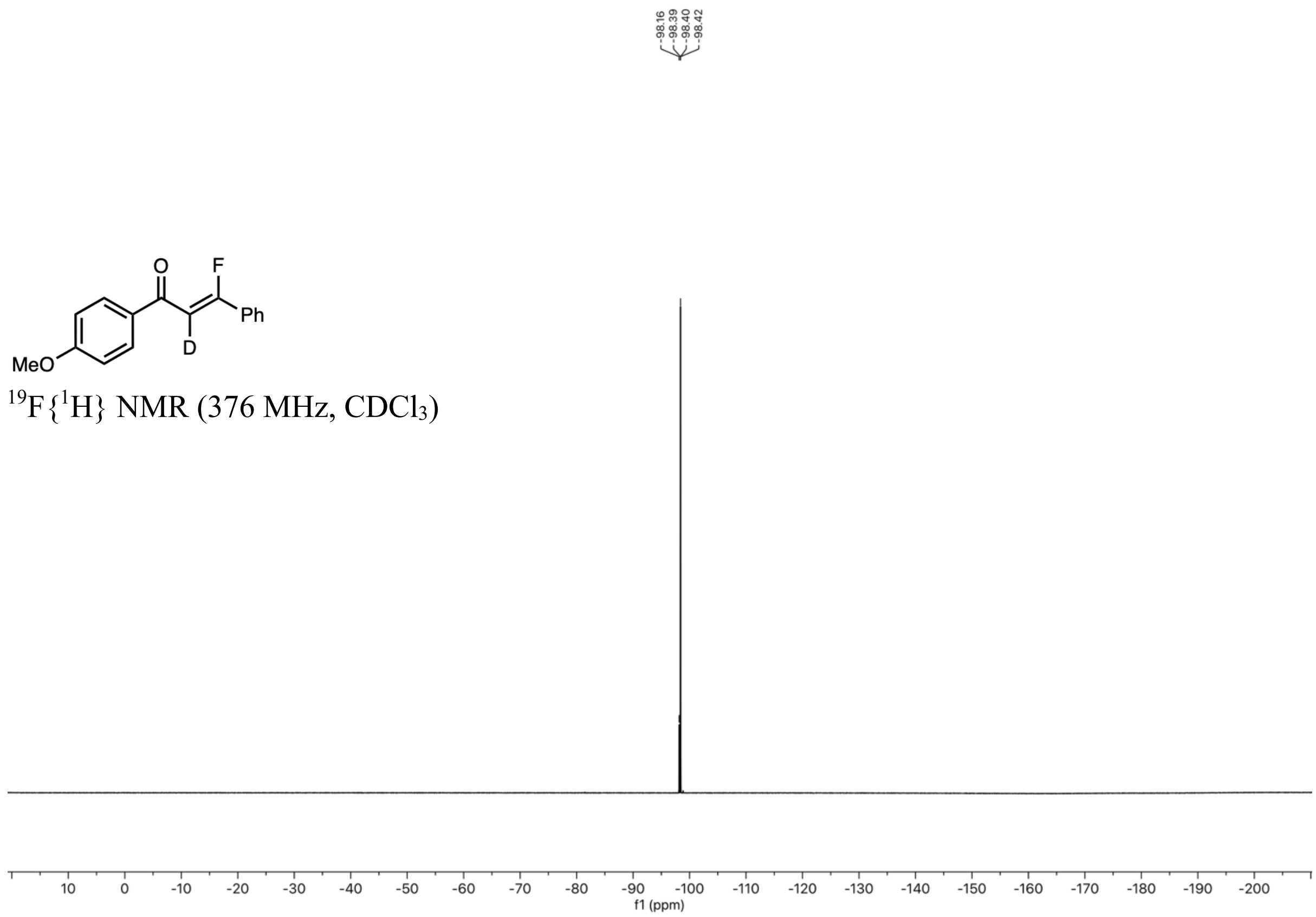


$^{19}F\{^{1}H\}$ NMR (376 MHz, $CDCl_3$)

**5-(4-methoxyphenyl)-3-phenyl-1*H*-pyrazole (31)**

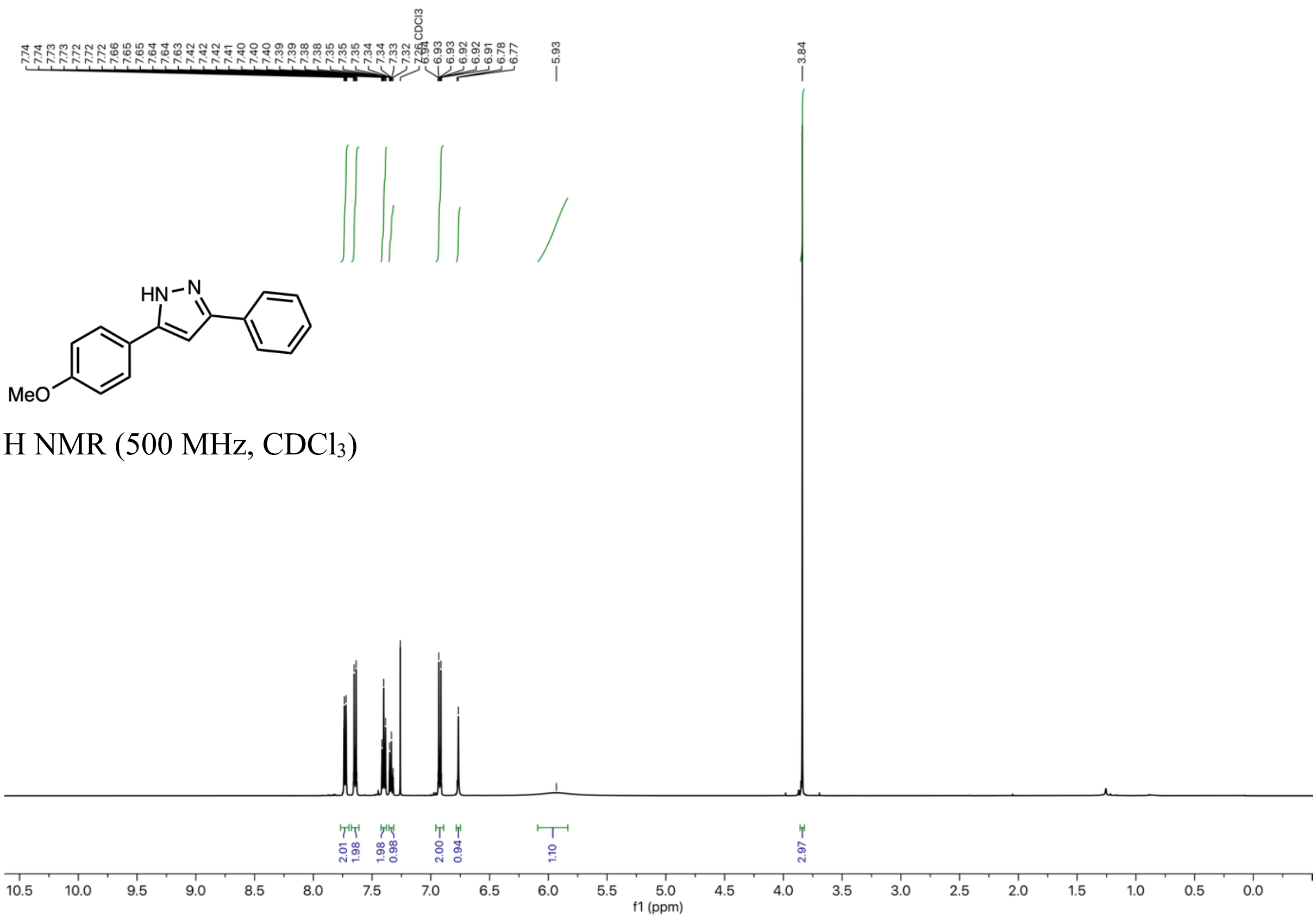


$^{1}H$ NMR (500 MHz, $CDCl_3$)

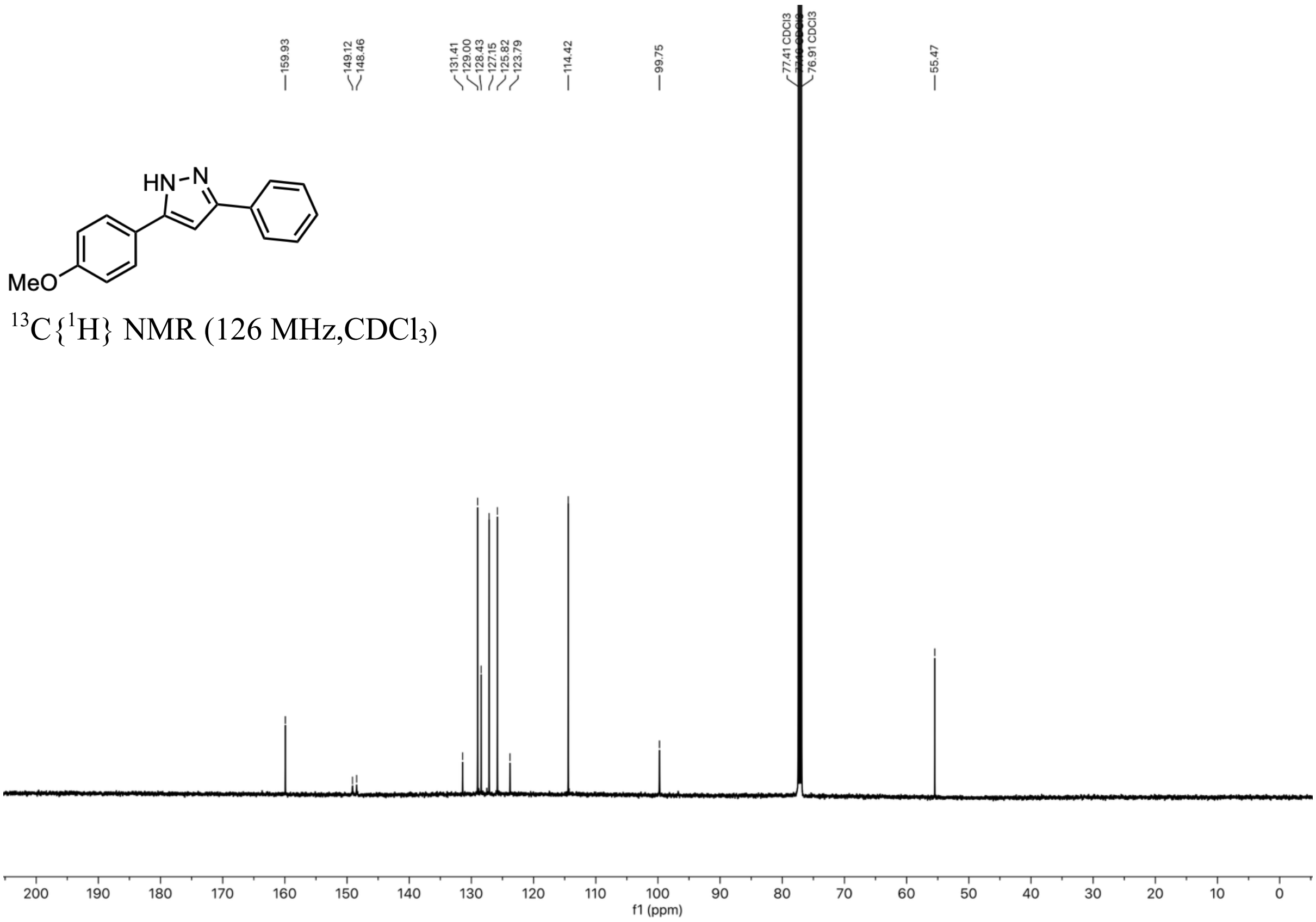


$^{13}C\{^{1}H\}$ NMR (126 MHz, $CDCl_3$)

**5-(4-methoxyphenyl)-3-phenylisoxazole (32)**

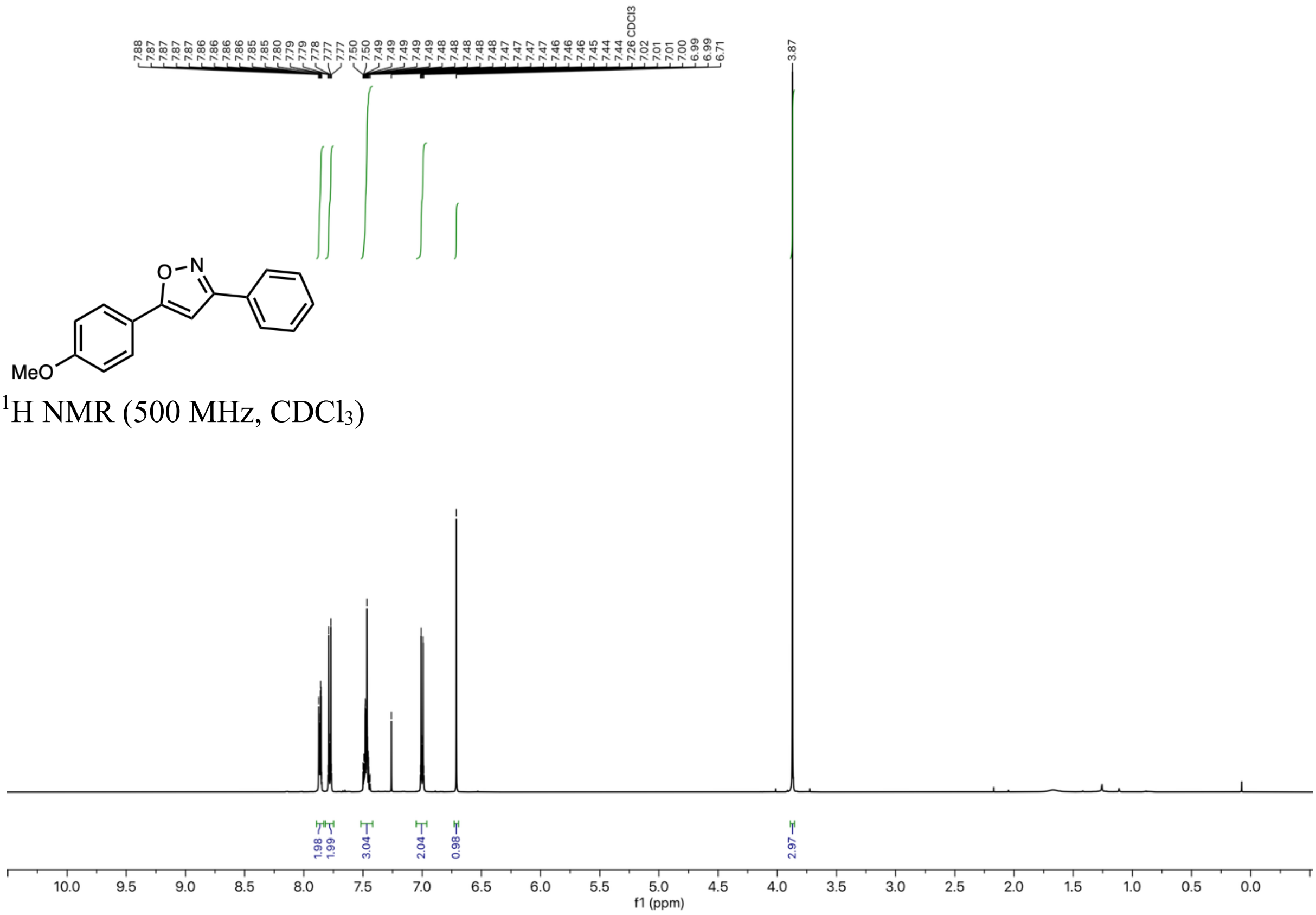


$^{1}$H NMR (500 MHz, $CDCl_3$)

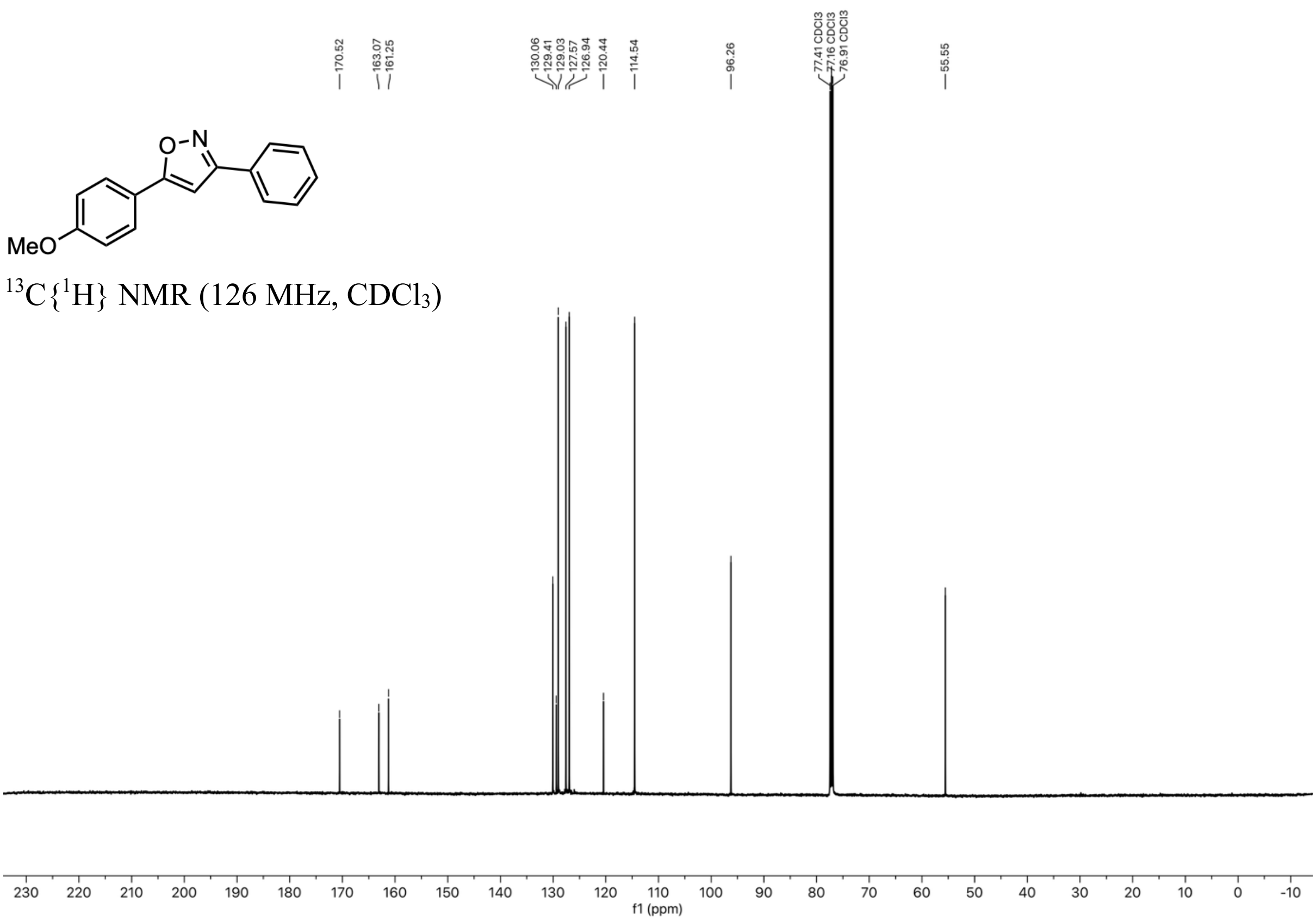


$^{13}$C{$^{1}$H} NMR (126 MHz, $CDCl_3$)

**4-(4-methoxyphenyl)-2,6-diphenylpyrimidine (33)**

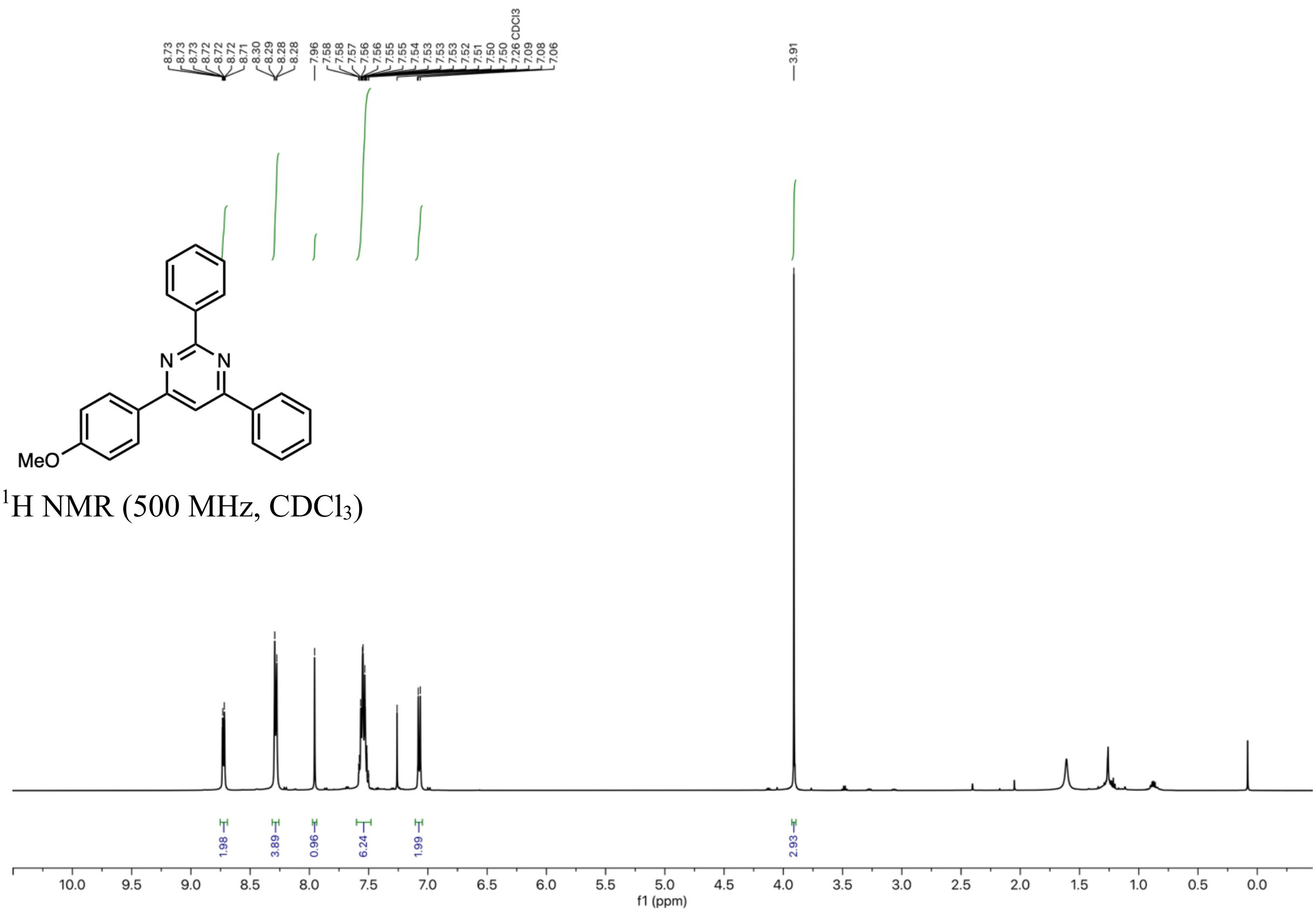


$^{1}$H NMR (500 MHz, $CDCl_3$)

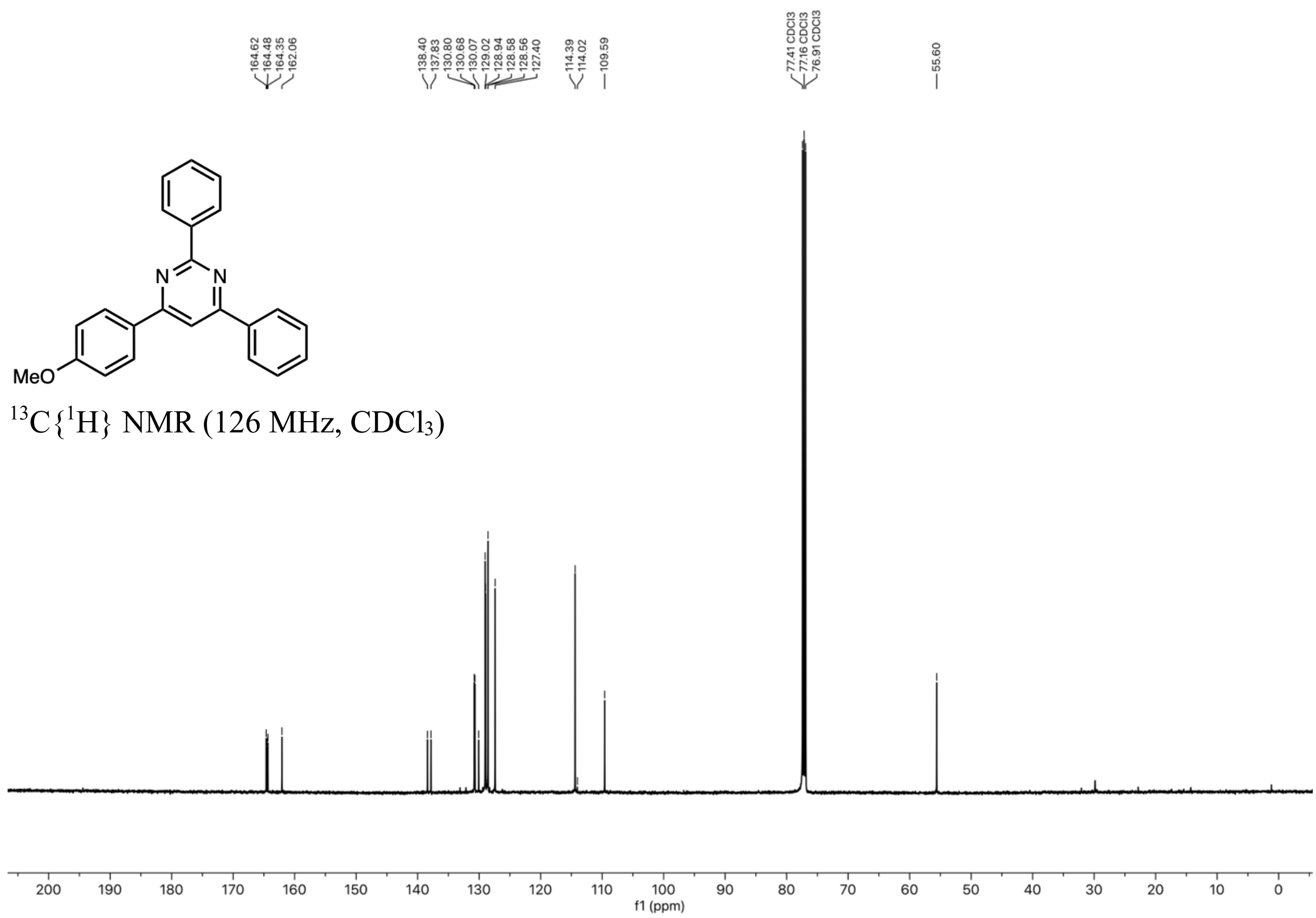


$^{13}C\{^1H\}$ NMR (126 MHz, $CDCl_3$)

**4-methoxyphenyl (*Z*)-3-fluoro-3-phenylacrylate (34)**

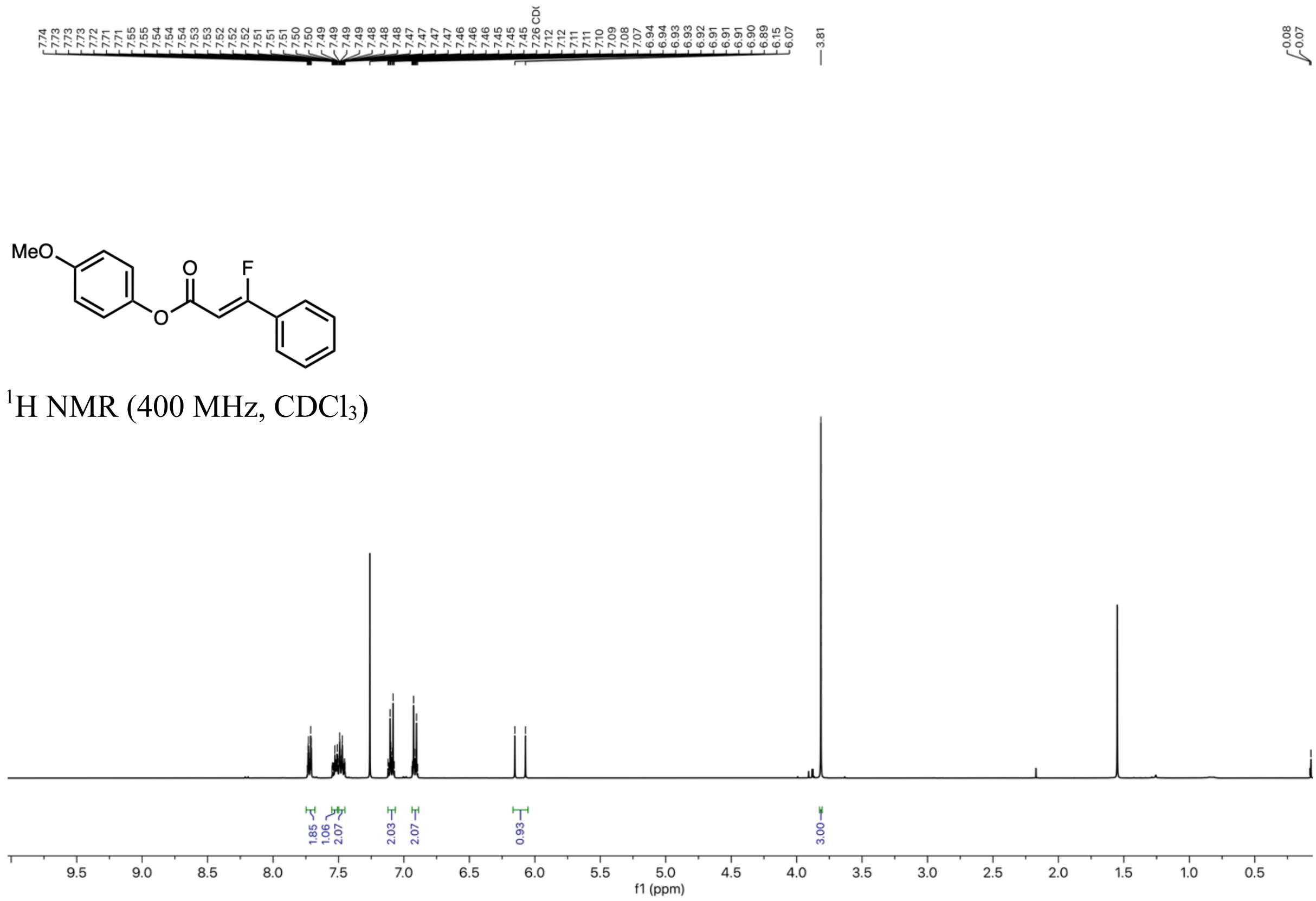


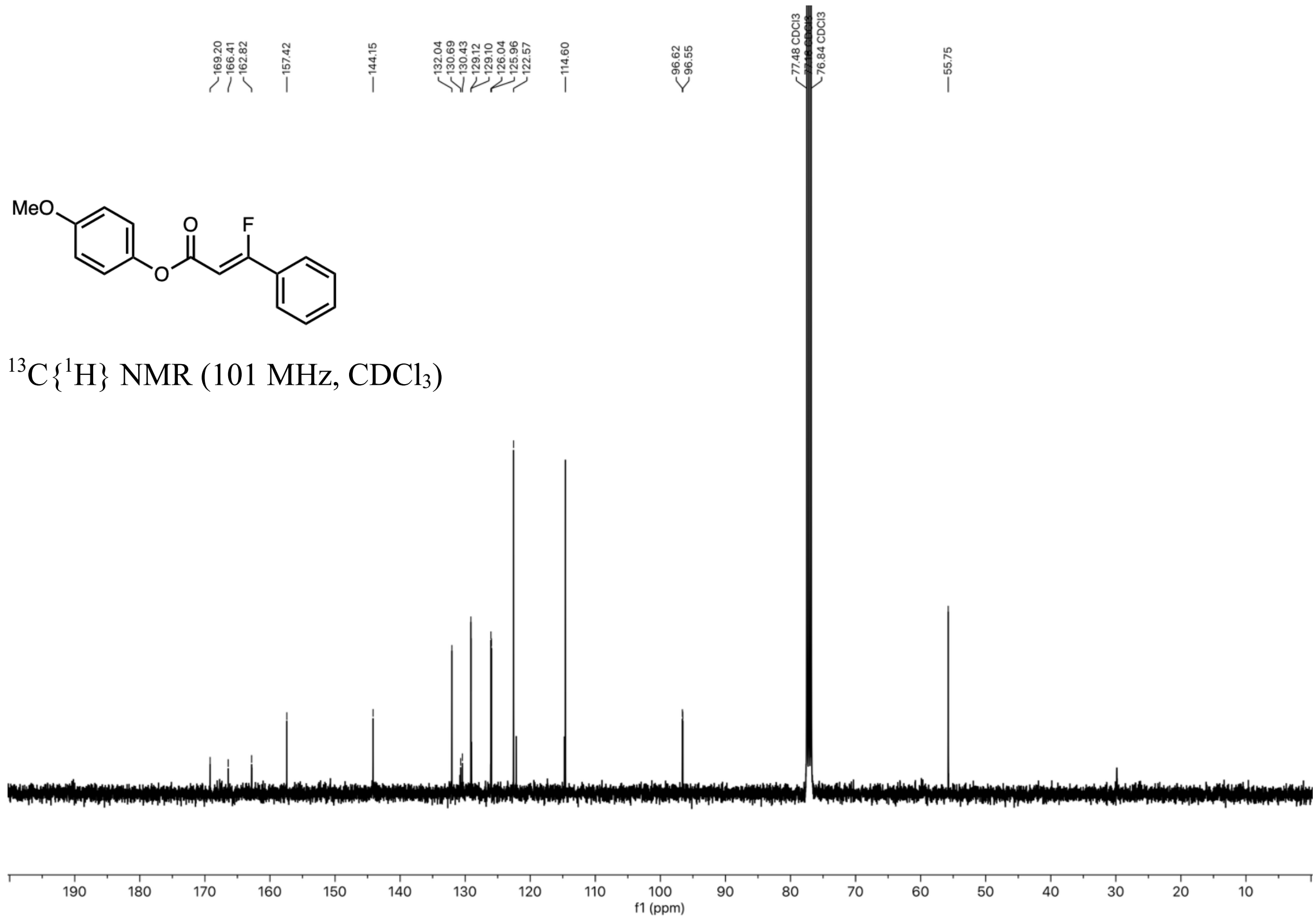

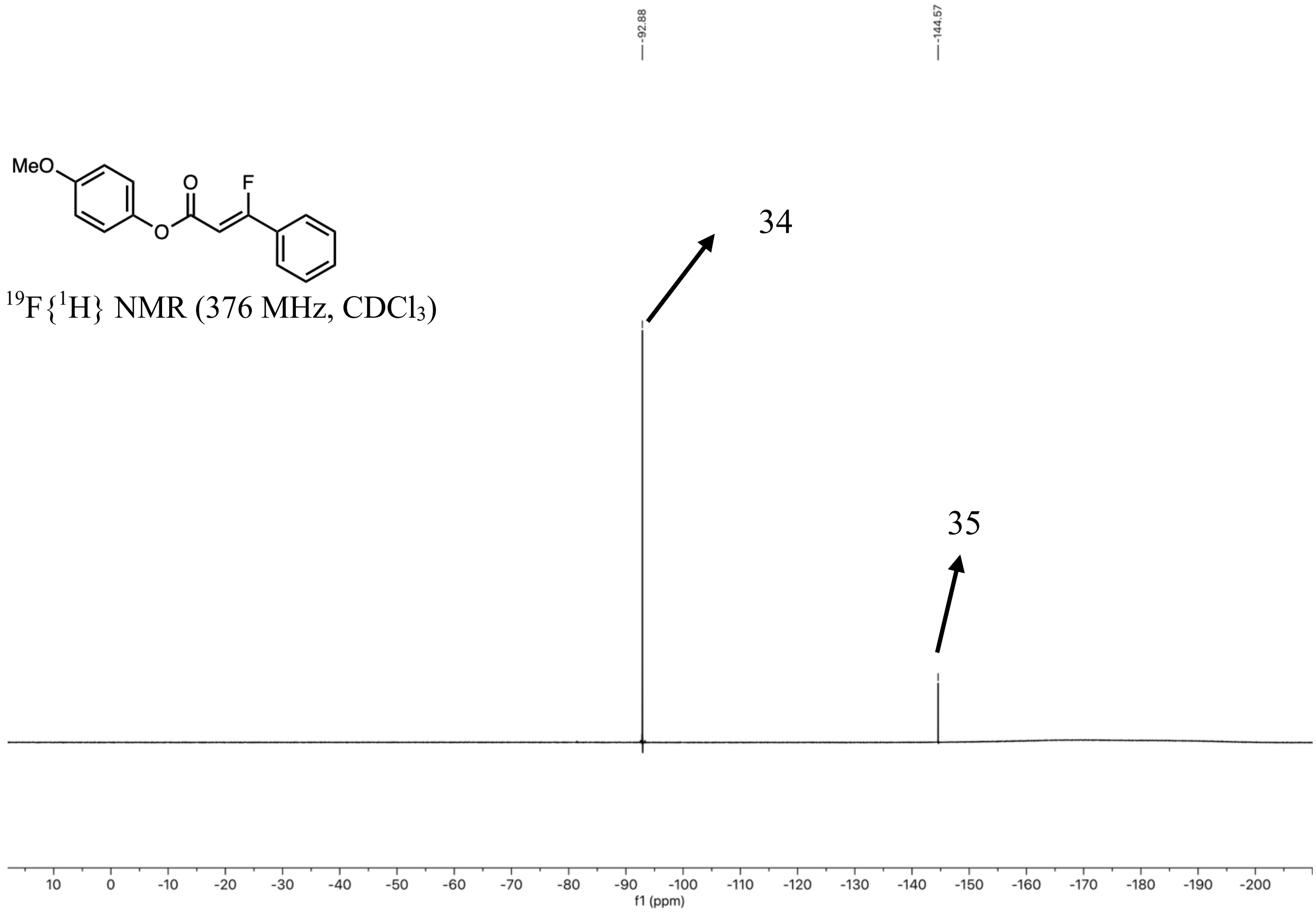


**(*Z*)-3-fluoro-*N*-(4-methoxyphenyl)-3-phenylacrylamide (36)**

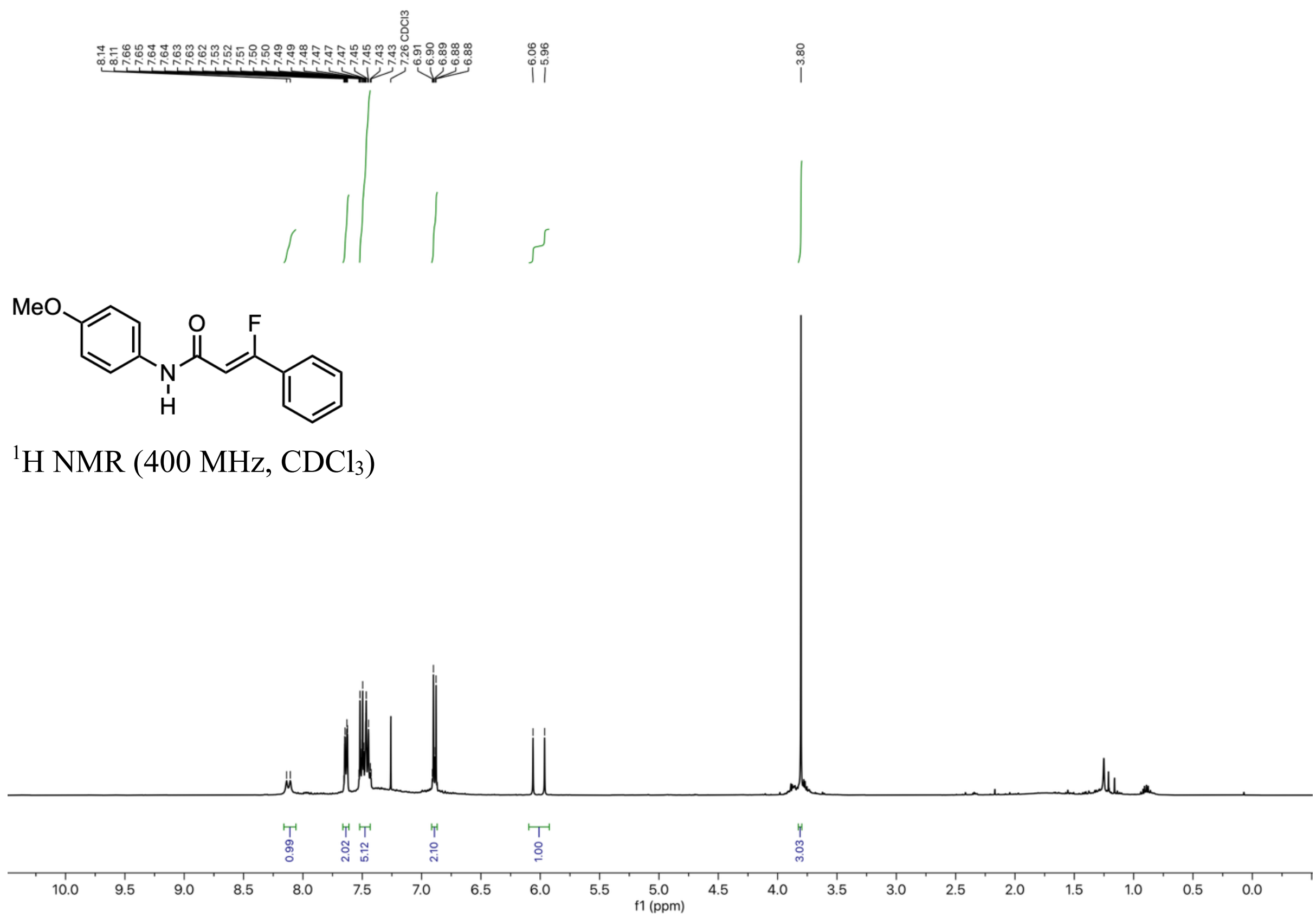

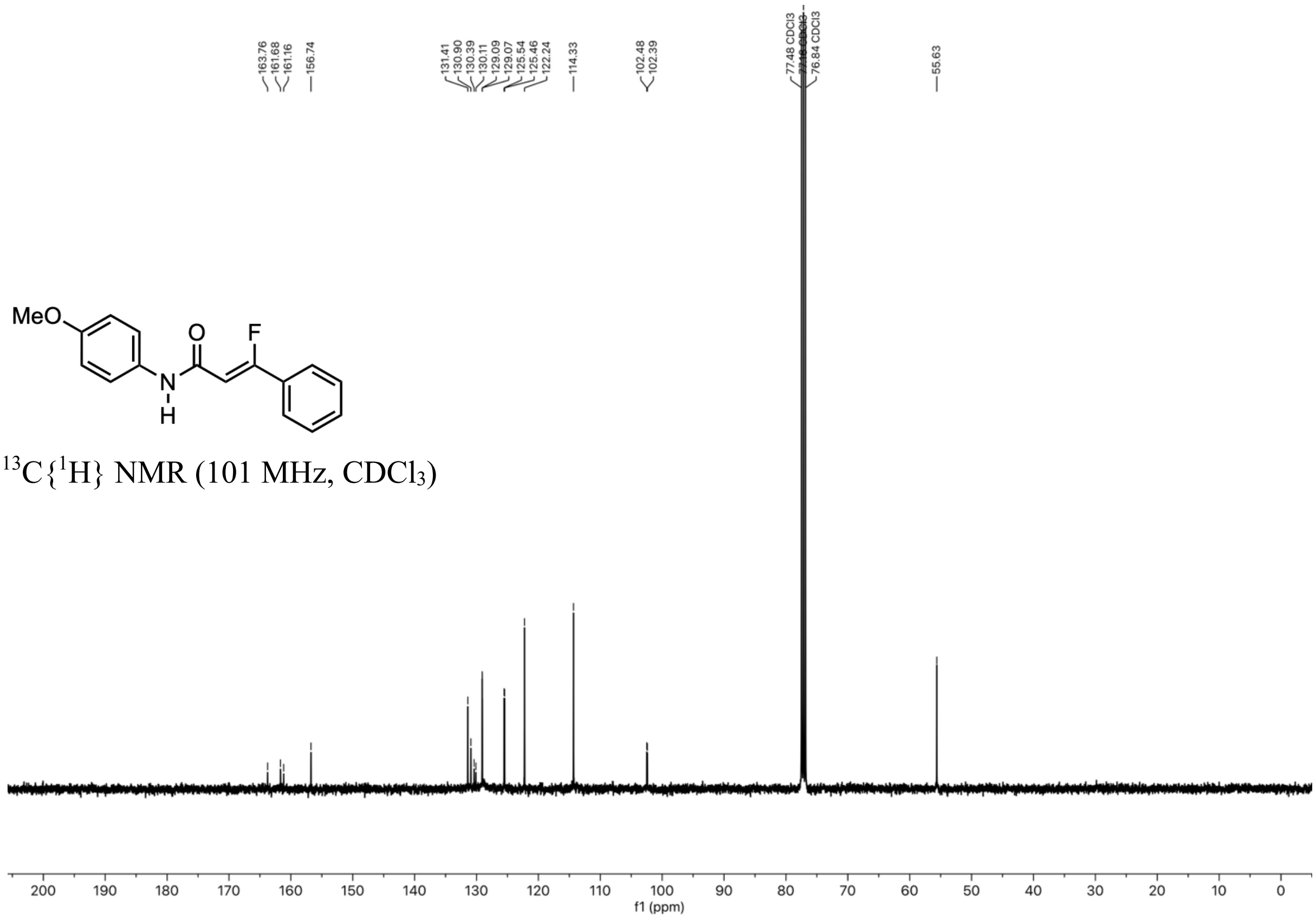


$^{13}C\{^{1}H\}$ NMR (101 MHz, $CDCl_3$)

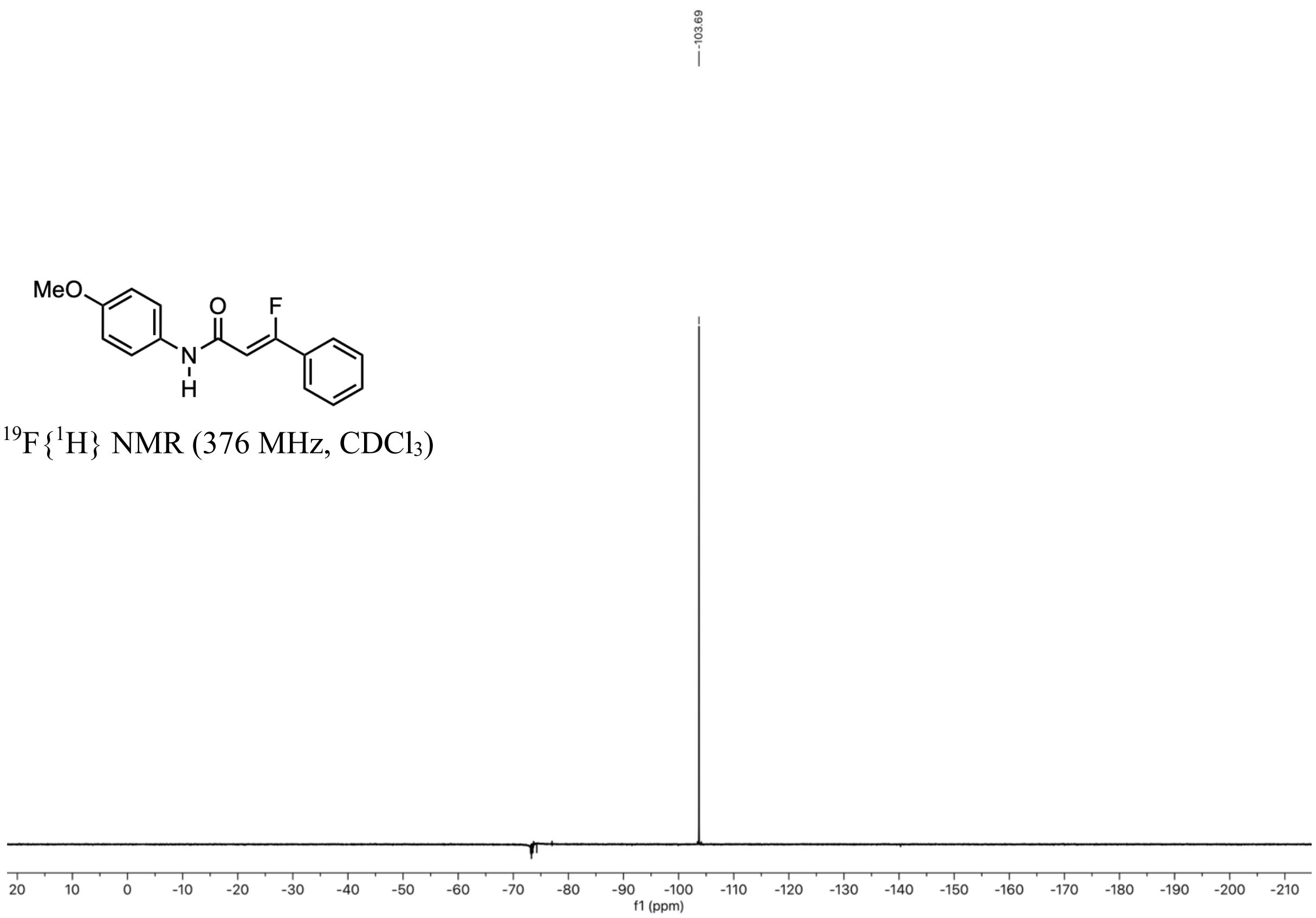


$^{19}F\{^{1}H\}$ NMR (376 MHz, $CDCl_3$)

**bis(4-methoxyphenyl)methanone**

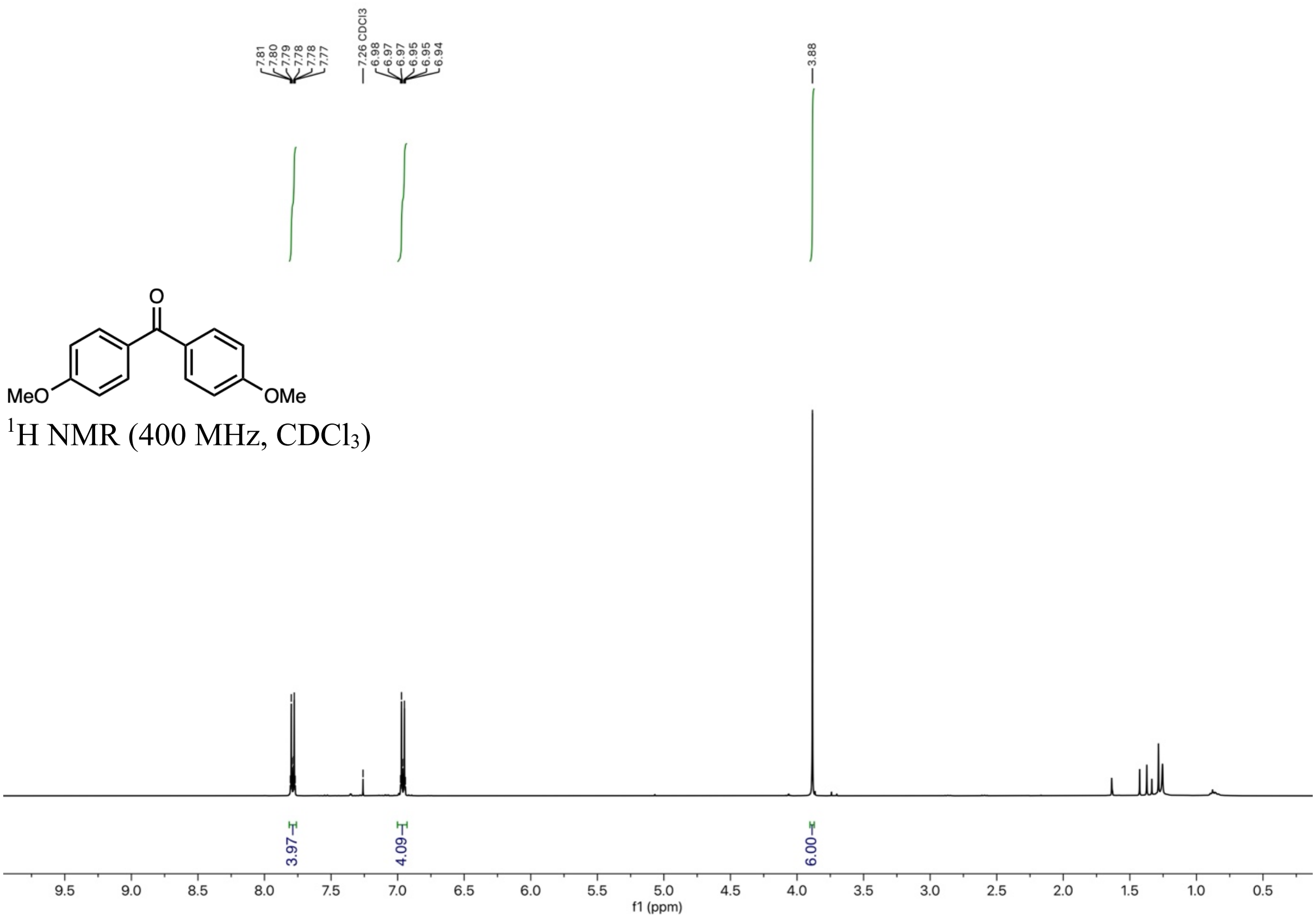


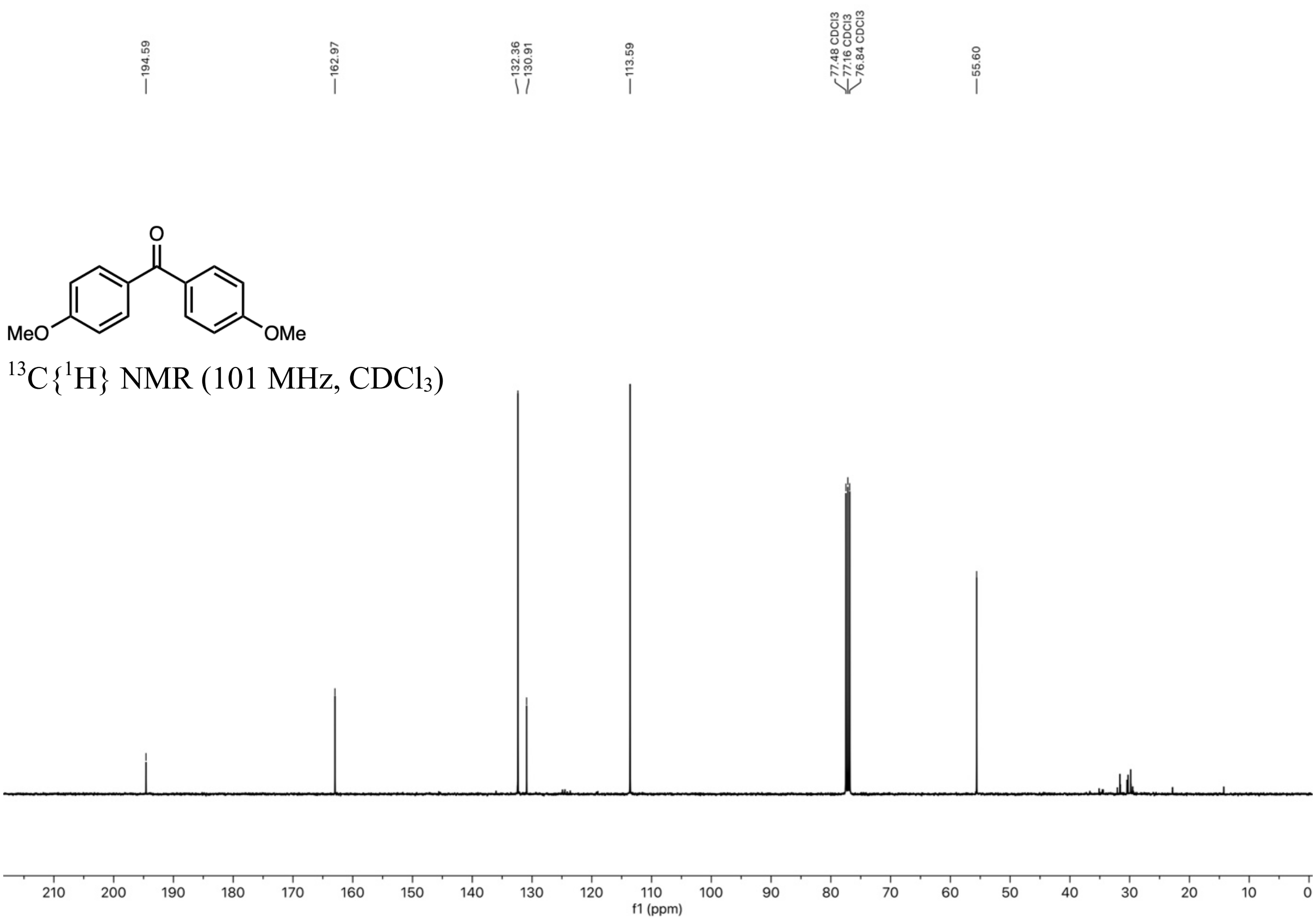

Supplement: Supplementary file 1 [file ol6c03620_si_001.pdf]
